# Artificial Intelligence-based database for prediction of protein structure and their alterations in ocular diseases

**DOI:** 10.1093/database/baad083

**Published:** 2023-12-18

**Authors:** Ling-Ping Cen, Tsz Kin Ng, Jie Ji, Jian-Wei Lin, Yao Yao, Rucui Yang, Geng Dong, Yingjie Cao, Chongbo Chen, Shi-Qi Yao, Wen-Ying Wang, Zijing Huang, Kunliang Qiu, Chi Pui Pang, Qingping Liu, Mingzhi Zhang

**Affiliations:** Joint Shantou International Eye Centre of Shantou University and The Chinese University of Hong Kong, North Dongxia Road (Guangxia New Town), Shantou, Guangdong 515041, China; Shantou University Medical College, 22 Xinling Road, Shantou, Guangdong 515041, China; Department of Ophthalmology and Visual Sciences, The Chinese University of Hong Kong, 147K Argyle Street, KLN, Hong Kong; Network & Information Centre, Shantou University, 243 Daxue Road, Shantou, Guangdong 515063, China; Joint Shantou International Eye Centre of Shantou University and The Chinese University of Hong Kong, North Dongxia Road (Guangxia New Town), Shantou, Guangdong 515041, China; Joint Shantou International Eye Centre of Shantou University and The Chinese University of Hong Kong, North Dongxia Road (Guangxia New Town), Shantou, Guangdong 515041, China; Shantou University Medical College, 22 Xinling Road, Shantou, Guangdong 515041, China; Joint Shantou International Eye Centre of Shantou University and The Chinese University of Hong Kong, North Dongxia Road (Guangxia New Town), Shantou, Guangdong 515041, China; Shantou University Medical College, 22 Xinling Road, Shantou, Guangdong 515041, China; Shantou University Medical College, 22 Xinling Road, Shantou, Guangdong 515041, China; Guangdong Provincial Key Laboratory of Infectious Diseases and Molecular Immunopathology, Shantou University Medical College, 22 Xinling Road, Shantou, Guangdong 515041, China; Joint Shantou International Eye Centre of Shantou University and The Chinese University of Hong Kong, North Dongxia Road (Guangxia New Town), Shantou, Guangdong 515041, China; Joint Shantou International Eye Centre of Shantou University and The Chinese University of Hong Kong, North Dongxia Road (Guangxia New Town), Shantou, Guangdong 515041, China; Joint Shantou International Eye Centre of Shantou University and The Chinese University of Hong Kong, North Dongxia Road (Guangxia New Town), Shantou, Guangdong 515041, China; Shantou University Medical College, 22 Xinling Road, Shantou, Guangdong 515041, China; Joint Shantou International Eye Centre of Shantou University and The Chinese University of Hong Kong, North Dongxia Road (Guangxia New Town), Shantou, Guangdong 515041, China; Shantou University Medical College, 22 Xinling Road, Shantou, Guangdong 515041, China; Joint Shantou International Eye Centre of Shantou University and The Chinese University of Hong Kong, North Dongxia Road (Guangxia New Town), Shantou, Guangdong 515041, China; Joint Shantou International Eye Centre of Shantou University and The Chinese University of Hong Kong, North Dongxia Road (Guangxia New Town), Shantou, Guangdong 515041, China; Joint Shantou International Eye Centre of Shantou University and The Chinese University of Hong Kong, North Dongxia Road (Guangxia New Town), Shantou, Guangdong 515041, China; Department of Ophthalmology and Visual Sciences, The Chinese University of Hong Kong, 147K Argyle Street, KLN, Hong Kong; Joint Shantou International Eye Centre of Shantou University and The Chinese University of Hong Kong, North Dongxia Road (Guangxia New Town), Shantou, Guangdong 515041, China; Shantou University Medical College, 22 Xinling Road, Shantou, Guangdong 515041, China; Joint Shantou International Eye Centre of Shantou University and The Chinese University of Hong Kong, North Dongxia Road (Guangxia New Town), Shantou, Guangdong 515041, China

## Abstract

The aim of the study is to establish an online database for predicting protein structures altered in ocular diseases by Alphafold2 and RoseTTAFold algorithms. Totally, 726 genes of multiple ocular diseases were collected for protein structure prediction. Both Alphafold2 and RoseTTAFold algorithms were built locally using the open-source codebases. A dataset with 48 protein structures from Protein Data Bank (PDB) was adopted for algorithm set-up validation. A website was built to match ocular genes with the corresponding predicted tertiary protein structures for each amino acid sequence. The predicted local distance difference test-Cα (pLDDT) and template modeling (TM) scores of the validation protein structure and the selected ocular genes were evaluated. Molecular dynamics and molecular docking simulations were performed to demonstrate the applications of the predicted structures. For the validation dataset, 70.8% of the predicted protein structures showed pLDDT greater than 90. Compared to the PDB structures, 100% of the AlphaFold2-predicted structures and 97.9% of the RoseTTAFold-predicted structure showed TM score greater than 0.5. Totally, 1329 amino acid sequences of 430 ocular disease-related genes have been predicted, of which 75.9% showed pLDDT greater than 70 for the wildtype sequences and 76.1% for the variant sequences. Small molecule docking and molecular dynamics simulations revealed that the predicted protein structures with higher confidence scores showed similar molecular characteristics with the structures from PDB. We have developed an ocular protein structure database (EyeProdb) for ocular disease, which is released for the public and will facilitate the biological investigations and structure-based drug development for ocular diseases.

**Database URL:**  http://eyeprodb.jsiec.org

Key messages

- The first study provides a protein structure database for ocular disease genes (EyeProdb).
- EyeProdb enables users searching on the web and submitting requests for specific amino acid sequences of genes related to ocular diseases, which in return expands the database of EyeProdb.
- EyeProdb can facilitate the biological investigations and structure-based drug development for ocular diseases.
- EyeProdb reduces requirement of human expert assistance.

## Introduction

Wildtype protein structures of various species can now be predicted by bioinformatics models, especially AlphaFold2 (1), which is freely accessed (https://alphafold.ebi.ac.uk/)(2). Protein prediction by AlphaFold2 programs is able to obtain comparable and even indistinguishable structures as compared to experimental proven structures (3). One recent advancement is the capability to accurately predict structures of G protein-coupled receptors (4). Similarly, RoseTTAFold, also released in 2021, has also been applied to predict different protein structures (5, 6). These algorithms could facilitate protein structural and functional studies in physiological conditions (7). Pathological situations, however, could be caused by mutant proteins resulted from gene mutations, which could be different from the wildtype proteins. AlphaFold2 and RoseTTAFold algorithms have been used to study genes and mutations in relation to the effects of structural changes to functions that affect biochemical pathways (8, 9). Protein structural databases/platforms for gene mutations should further speed up the investigations on disease mechanisms and drug development (10, 11).

Hundreds of genetic ocular diseases have so far been discovered with specific gene mutations, including congenital cataracts, retinitis pigmentosa (RP), congenital glaucoma, ocular coloboma, Leber’s congenital amaurosis, Stargardt disease, cone-rod dystrophies, choroideremia, inherited optic atrophy, corneal dystrophies and nystagmus. Congenital ocular diseases account for 60% of blindness among infants, and 1.4 million children under the age of 16 years worldwide are visually impaired (12). The etiology of most of these diseases remains to be fully understood. Understanding the changes in protein structures of the gene mutations could facilitate the investigation of ocular disease mechanisms and further drug development.

With the release of open source code, especially the inference code and trained models of AlphaFold2 and RoseTTAFold algorithms, the determination of protein structures of different ocular disease gene mutations by artificial intelligence-based prediction might open a new way in addition to the experimental methods that require sophisticated and expensive technologies, including X-ray crystallography, nuclear magnetic resonance (NMR) imaging and cryo-electron microscopy (1, 13). To facilitate the biological investigations and structure-based drug development for ocular diseases, we aimed to establish a protein structure database for ocular diseases. In this study, based on the source code and trained models of AlpbhaFold2 and RoseTTAFold, we assembled the AlphaFold2 and RoseTTAFold protein structure prediction platform for the protein structure prediction of wildtype and variant amino acid sequences for different ocular diseases. The protein structure prediction performance of the validation dataset and ocular genes was evaluated. In addition, molecular dynamics and molecular docking simulations were performed to determine the applications of the predicted protein structures.

## Methods

### Ocular disease gene collection and classification

More than 700 ocular genes with pathogenic mutations were collected from Uniprot (14) and the database of hereditary ocular diseases developed by The University of Arizona (https://disorders.eyes.arizona.edu/), which are summarized in Supplementary Table S1.

### Computational system setting and algorithms construction

#### Protein structure prediction

Source code and trained models of Alphafold2 (1) and RoseTTAFold (15) were downloaded and deployed in on-premises data center. A protein structure prediction platform, which combines the full functionality of Alphafold2 and RoseTTAFold, was built and can be accessed through the internet. Given one or a batch of sequences, their protein structures were predicted in parallel on multiple servers. The prediction results were recorded in the database. Besides protein structure prediction, the platform is a multi-layered architecture using the model view controller (MVC) design pattern, which includes database, web application and web browser.

#### Hardware and software environment

Four servers were used to predict protein structures. The following is hardware and software environment of these servers: CPU with Intel i9-10980XE, 256GB DDR4 Memory, Nvidia RTX3090 GPU * 2, 2 Samsung SSD 870 EVO 4TB hard disks. Ubutnu 18.04, Cuda11.1.

#### Alphafold2

DeepMind released its open source version of Alphafold2 (1) on 15 July 2021. The recommended operating environment is based on Docker. The codebase of Alphafold2 was downloaded to our servers on 10 August 2021. We made some changes to both the parameters and source code. In order to support CUDA11.1, ARG CUDA was set to 11.1 in ‘Dockerfile’ and then the docker image was rebuilt. Considering the performance of computer hardware and to improve computing speed, the n_cpu parameters in the data/tools/hhblits.py and data/tools/jackhammer.py files were set doubled. The following two lines were added into the pipeline.py script to fix a bug (http://alphafold.hegelab.org/).

uniref90_msa = uniref90_msa[:self.uniref_max_hits] # hege

uniref90_deletion_matrix = uniref90_deletion_matrix[:self.uniref_max_hits] # hege

Alphafold2 continues evolving, and the latest version is 2.1.1. Some application programming interfaces (APIs) may have been changed by the time this article is published. The above method may be only suitable for the specific version as used in this study.

#### The prediction pipeline

In the Alphafold2 source code repository, the prediction program file ‘run_docker.py’and a command line script were used to do prediction. To improve the efficiency of batch prediction, some minor modifications have been made to this program file. A custom program was developed to predict sequences in parallel using different GPUs and automatically using different parameters for different sequences. The parameter ‘preset’ was set to ‘reduced_dbs’ for sequences longer than 2000 and ‘full_dbs’ for others, and the parameter ‘max_template_date’ was set to ‘2021–08-14’. The ‘max_template_date’ was prior to the dates of determining protein structures of sequences that was used for validation using experimental methods. Five structures were obtained for each input sequence. After performing an amber (16) relaxation procedure on the unrelaxed structure prediction, these five models were ordered and ranked_0.pdb for the prediction. The one of the highest confidence was selected as the final model.

#### RoseTTAFold

According to the Baker Lab, the source code and trained models of RoseTTAFold (15) were publicly available almost as same as Alphafold2. The recommended running environment was Conda. The codebase of RoseTTAFold was downloaded to our servers on 12 August 2021. The Pyrosetta version was used instead of the end-to-end version, and model_1.pdb was chosen as the selected model. RoseTTAFold is equipped with a prediction shell script ‘run_pyrosetta_ver.sh’. The parameters ‘CPU’ and ‘MEM’ in this file were set tripled. The statically compiled version HH-suite (17) in the Conda environment ‘RoseTTAFold’ was replaced with that of building from source in order to fix a bug that displayed the message ‘segmentation fault(core dump)’ for a few sequences. For 23 sequences, which raised error ‘error in hhalignment.cpp:X: MergeMasterSlave:’ during hhblits, the sequence databases parameters were modified from DB = ‘$PIPEDIR/UniRef30_2020_06/UniRef30_2020_06’ and MYDB = ‘$PIPEDIR/bfd/bfd_metaclust_clu_complete_id30_c90_final_seq.sorted_opt’ to only the UniRef30 database. Except for this modification, other parameters are set by default. A custom python program that called this script was developed to predict sequences in parallel using different GPUs. Given one or a batch of sequences, their protein structures were predicted in parallel on multiple servers. As for every sequence, five pdb files with pLDDT (18) scores were generated by Alphafold2 and five pdb files with estimated CA rms error (19) values were generated by RoseTTAFold, respectively. After prediction, all the data, including the sequence, prediction type, parameters used in prediction, predicted pdb files and corresponding pLDDT or estimated CA rms error values, were recorded into the database.

#### Database

MySQL 5.7.37 was used as the database management system. There are three main tables: disease, sequence and protein structure prediction. The relationship between diseases and sequences is many to many, and the relationship between sequences and predictions is one to many. The relationship between genes and Online Mendelian Inheritance in Man (OMIM) IDs is one to one. Sequences were stored as both the TEXT field of the database and external fasta files. Likewise, protein structure data were stored as both the TEXT field of the database and external fasta files.

#### Web application

Python 3.6.10, Flask2.0.2 and mysql-connector-python 8.0.27 were used to develop the server-side web application. Flask was used to construct both the MVC application and Web APIs. Client-side web pages were built using HTML5, CSS and Javascript. The request library was used to call Web APIs. Importantly, the ngl (20) library was used to visualize protein structures in the web browser. The web site included two entry points, one for administrators and the other for public users. Administrators can add (or delete, update) diseases and sequences, and predict protein structures of one or multiple sequences. Public users can view the diseases, sequences and predicted protein structures. They can also upload their own sequences and receive their predicted protein structures after a period of time.

### Validation of prediction algorithms

These locally built AlphaFold2 and RoseTTAFold algorithms were validated with a dataset containing 48 experimental protein structures, which were newly released (after 1 September 2021) in PDB. These proteins with sequence length of 124–1332 have not been used for development of the algorithms. With the structure prediction, per-residue confidence score (pLDDT, 0–100), estimated CA rms error, TM-score (0–1), IDDT (0–1) and GDT_TS-score (0–1) of each prediction were also obtained for further analysis and comparison.

### Ligand docking and molecular dynamics simulation

#### Computational materials

Proteins were selected on the basis of their pLDDT scores of the AI platform. The PDB structure of the protein was retrieved from the protein data bank (https://www.rcsb.org) by the high-resolution X-ray. The Alphafold structure was from the AI platform. The ligand library used for high throughput virtual screening was from FDA-approved drugs (∼1500 compounds) in ZINC database (https://zinc.docking.org).

#### Protein preparation and ligand preparation

The structures of selected proteins from PDB database and our prediction by Alphafold2 were first optimized by module of *protein preparation wizard* in Schrödinger2021-1 package. The process of protein preparation utilized options such as assigning of bond orders, the addition of hydrogen atoms, the treatment of formal charges and the abstraction of water molecules. The optimized protein structure was used for the subsequent docking calculations (21). Ligands were prepared using the *LigPre*p module in Schrödinger. The protonation state and tautomeric states were determined at 7.0 ± 2.0 pH. Energy minimization was carried out with OPLS force field. The optimized ligands structures were then used for molecular docking.

#### Binding site prediction

Binding site identification is useful for drug discovery (22). Incorrect binding site would directly cause failure of molecular docking. Herein, binding sites were predicted by the *Receptor Grid Generation* panel in Schrödinger. For structures solved with ligands, this tool would directly use the ligand position as the binding site. For structures without ligands, the program would predict several binding sites. Once the binding site was determined, the grid box could be generated automatically. For the grid box generation, default parameters were used (21).

#### High throughput virtual screening (HTVs) and Ligand docking

Ligand docking was performed using the Grid-based Ligand Docking with Energetics (GLIDE) module. High throughput virtual screening of the selected compound (∼1500 compounds from FDA-approved drugs) was performed against the target protein with a flexible docking parameter for ligands.

The prepared glide grids were selected for molecular docking studies and each ligand was docked to PDB-protein individually and generated a best binding pose ligand–protein complex with the minimum dock score (D score) and glide energy (G energy). Then, the same ligand was docking to Alphafold-predicted protein by standard precision (SP). Default parameters were used, and no constraints were applied during the docking process.

#### Molecular dynamics (MD) simulations

MD simulation was carried out by the Desmond module of the Schrödinger suite. The ligand–protein complex obtained from molecular docking was used as the starting structure. Briefly, a model system was built using the *system builder* module in maestro, including determination of protonation states of residues after adding water box and counter ions (Na^+^ and Cl^−^). TIP3P model was used for water molecules and the shape water box was set as orthorhombic. For all simulations, OPLS_2005 force field was used (23). Finally, 100-ns simulation was carried out. The saved trajectories were analyzed using *Simulation Interaction Diagram*, including the root mean square deviation (RMSD) (24) and root mean square fluctuation (RMSF).

#### Cross correlation and normal mode calculation

The dynamic correlations between residues were calculated by Bio3d program. The NetCDF-formatted trajectory generated by Amber after production simulation was sampled and converted into a dcd format file, which was input into Bio3D along with the pdb-formatted simulation initial structure. All parameters used to analyze residue cross-correlation were default. The two-dimensional residue cross-correlation matrix was drawn by the igraph module of Bio3D. Normal modes calculation was performed with ProDy package. The motions of proteins were visualized by VMD program.

## Results

### Validation of the set-up of the protein structure prediction algorithm

**Figure 1.**
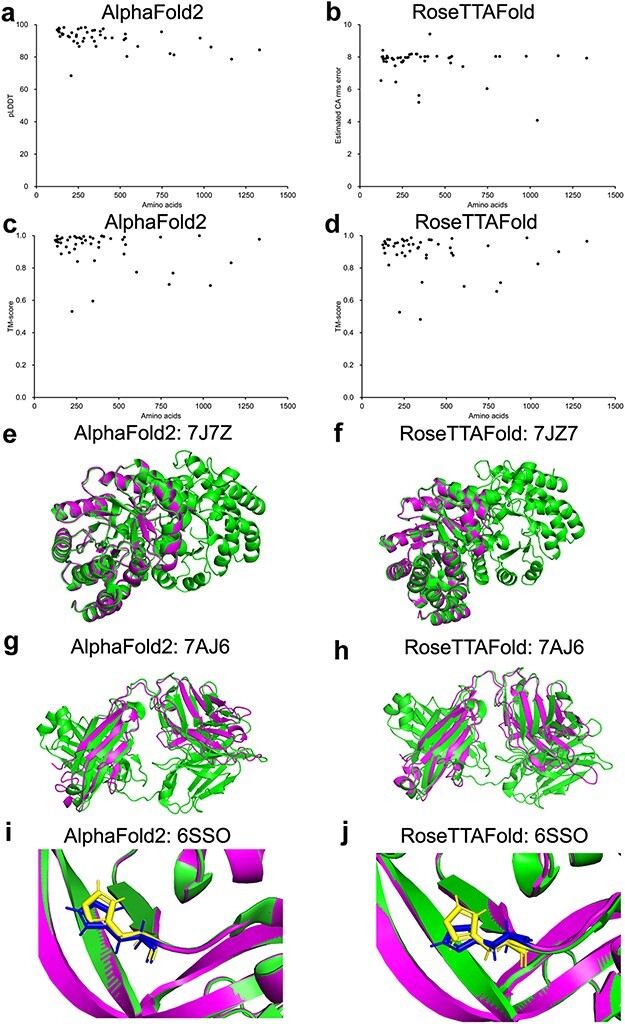
Model confidence profiling for validation genes. (a–d) The corresponding pLDDT (estimated CA rms error) and TM-score of each prediction generated by AlphaFold2 and RoseTTAFold were plotted. (e–h) Structural alignments of prediction of 7JZ7 and 7AJ6. (i–j) Similar geometry of the side chains of 6SSO.

Ten protein structures predicted by our platform were randomly selected and compared to the corresponding protein structures released in the AlphaFold website. The TM-score and lDDT of the comparison between the structures predicted by our platform and the corresponding structures released in the AlphaFold website ranged from 0.913 to 1.000 and from 0.930 to 0.990 (Supplementary Table S2), respectively, indicating that the structures predicted by our platform showed high similarity to that released in the AlphaFold website. To validate the set-up of the open-source code of the AlphaFold2 and RoseTTAFold algorithm, the amino acid sequences of 48 protein structures released from the RCSB protein data bank (PDB; https://www.rcsb.org/, after 1 September 2021) were taken for the prediction validation analysis (Supplementary Table S3). The corresponding TM-score and pLDDT of each prediction generated by AlphaFold2 and RoseTTAFold were plotted with the number of residues (124–1332 residues) in Figure 1a and b. Among the 48 selected structures, 97.9% (47/48) of the AlphaFold2 predictions showed pLDDT greater than 70 and 70.8% (34/48) greater than 90. For RoseTTAFold predictions, 89.6% (43/48) have estimated CA rms error greater than 0.7 and 66.7% (32/48) greater than 0.9. Compared to the PDB-released structures, 100% (48/48) of the AlphaFold2 predictions showed TM-score greater than 0.5, and 97.9% (47/48) of the RoseTTAFold predictions showed TM-score greater than 0.5. The structural alignments of 7JZ7 prediction (with both high pLDDT and TM-score) are shown in Figure 1c and d, and the alignments of 7AJ6 prediction (with high pLDDT but lowest TM-score) are shown in Figure 1e and f. Critically, 11 mutant protein structures were evaluated for the potential of mutant protein structure prediction by our AlphaFold2 and RoseTTAFold algorithms. Most of the predicted mutant protein structures showed good alignments with the corresponding PDB structures (TM-score >0.5) with notably similar geometry of the side chains (Figure 1i and j, Supplementary Figure S1). Collectively, our results indicated that our set-up of the AlphaFold2 and RoseTTAFold algorithms could be able to generate protein structures with the input of amino acid sequences, and should be useful for protein structure prediction.

### Interface of EyeProdb

**Figure 2.**
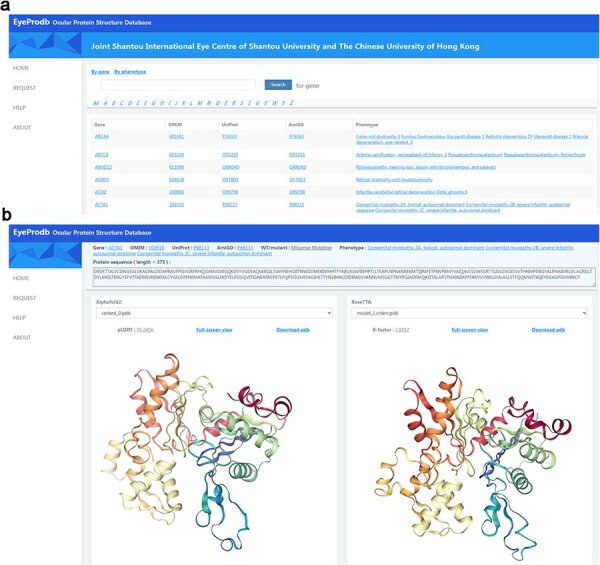
Searching EyeProdb. (a) EyeProdb provides a search engine to find ocular proteins of interest based on gene/protein name or phenotype. (b) Meta-information and 3D visualization of the EyeProdb structure predictions.

This newly established Ocular Protein Structure Database, Eyeprodb (http://eyeprodb.jsiec.org), can reliably provide a database for the predicted protein structures of the ocular disease genes. The predicted structures are listed in alphabetical orders according to the genes or the phenotypes (Figure 2). Users could search by the gene/protein name or phenotype. Each gene is linked to OMIM for genetic information in detail, UniProt for annotation of protein domains, structure and functional sites of the protein and AmiGO for GO terms and their annotations. Each protein has a prediction-list page that showed the sequence length, pLDDT score and estimated CA rms error for each of the listed wildtype and its mutants (Supplementary Figure S2a). Five predictions of the AlphaFold model and the RoseTTAFold model were listed by the pLDDT score and estimated CA rms error, respectively. Tertiary protein structures of each prediction could be visualized in a full-screen view with a built-in 3D Pdb viewer, with pLDDT or estimated CA rms error (Figure 2b, Supplementary Figure S2a). Customized service for protein structure prediction of specific genes could be obtained on a request page (Supplementary Figure S2b). By providing protein sequence with gene information and contact information, users could submit a service request to our platform and obtain a respond within a few days.

### Ocular disease genes and variant protein structure prediction analysis

**Figure 3.**
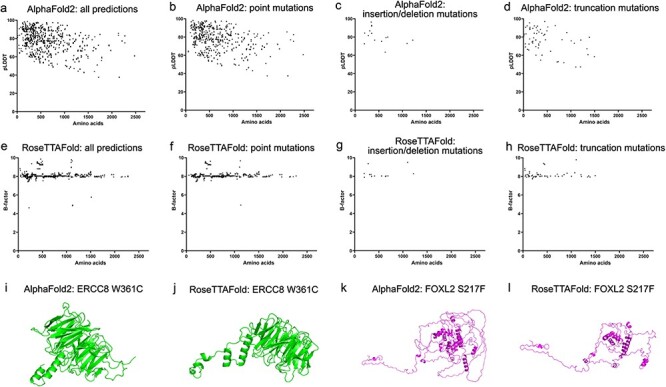
Model confidence profiling for ocular genes. (a and e) The pLDDT by AlphaFold2 and estimated CA rms error by RoseTTAFold for each prediction were plotted with the number of residues. (b–d and f–h) The pLDDT and estimated CA rms error were plotted according to different types of mutations.

In this study, 726 genes with phenotypes and OMIM numbers have been collected. Among them 1329 amino acid sequences (with the length of 11–2479 residues) of both wildtype and variants of 430 genes have been predicted by our set-up of Alphafold2 and RoseTTAFold algorithms These predicted genes were summarized according to the ocular tissues, the type of diseases, including strabismus, lens, vitreous, ocular deformity, eyelid, neuro-ophthalmology, choroid, iris, retina, cornea, myopia and glaucoma in Table 1. Top five predicted structures would be generated by each algorithm (Supplementary Table S4). The confident score pLDDT by AlphaFold2 and estimated CA rms error by RoseTTAFold for each prediction were plotted with the number of residues (Figure 3a and e). We found that 75.9% (315/415) of predictions showed pLDDT larger than 70 for the wildtype sequences and 76.1% (596/783) for the variant sequences. The plot also indicated that the prediction confidence for shorter sequences was higher than that for the longer ones. In contrast, no obvious trend was noticed in the plot of estimated CA rms error with the number of residues. Different types of mutations showed similar distributions of pLDDT and estimated CA rms error along the number of residues (Figure 3b**–****d and f****–**h).

**Table 1.** Summary of inheretary ocular genes and sequence for structure prediction

	No.				
Disease type	Gene	Sequence	Wild type	Mutant	Sequence length median (range)
Myopia	53	182	52	130	553 (99–2479)
Eyelid	16	44	16	28	458 (215–1661)
Cornea	63	209	62	147	542 (114–2479)
Glaucoma	34	139	33	106	543 (152–1786)
Iris	26	111	26	85	472 (100–1786)
Choroid	10	31	10	21	585 (220–1834)
Lens	107	346	107	239	422 (21–2479)
Vitreous	8	24	8	16	201 (109–1493)
Retina	276	823	274	549	555 (11–2479)
Neuro-ophthalmology	111	355	110	245	467 (37–2479)
Strabismus	24	72	24	48	513 (125–1935)
Ocular deformity	44	132	44	88	389 (109–2479)
**All^a^**	**430**	**1329**	**426**	**903**	**498 (11–2479)**

a Numbers of gene sequence wild type and mutant are less than the sum of different disease types, because some genes with multiple signs are repeatedly counted.

### Highly confident predictions and molecular simulations

**Figure 4.**
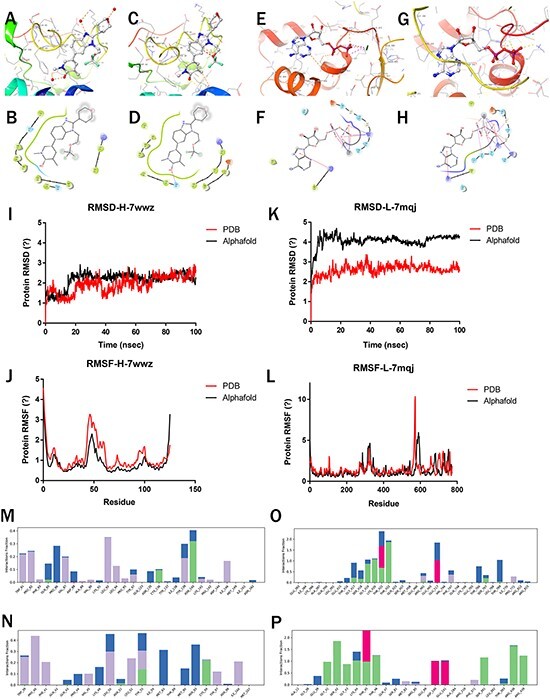
Molecular simulations of Highlighted predictions compared with PDB structures. (a–b) 7wwz-PDB-Protein docking with 7ow; (c–d) 7wwz-Alphafold-Protein docking with 7ow; (e–f) 7mqj-PDB-Protein docking with ADP; (g–h) 7mqj-Alphafold-Protein docking with ADP; (i–k) RMSD of 7wwz and 7mqj; (j–l) RMSF of 7wwz and 7mqj; (m–p) Complex interaction: (m) 7wwz-PDB-Complex, (n) 7wwz-Alphafold-Complex, (o) 7mqj-PDB-Complex, (p) 7mqj-Alphafold-Complex.

After the molecular docking of the selected ligand (Figure 4a**–**h), 100-ns MD simulations of the complexes were performed to compare the stability and dynamic behavior of PDB and predicted protein structure. The stability and structural flexibility of these complexes were studied through RMSD and RMSF, respectively.

The PDB (7wwz) protein’s RMSD for BRD4 co-crystallized complex remained stable, touched 2.65 Å at 100 ns and the predicted protein (pLDDT: 96.76) reached 2.19 Å at 100 ns (Figure 4i). For the DHR1 co-crystallized complex, the RMSD of PDB(7mqj) protein reached 2.53 Å at 100 ns while the predicted protein (pLDDT: 82.06) RMSD reached 4.31 Å (Figure 4k). The predicted protein with higher pLDDT is more stable.

For the BRD4 co-crystallized complex, the RMSF values of the residues around the active site did not exceed 3.2 Å in both PDB and predicted proteins (Figure 4j). This indicates that the flexibility of residues in the active site was not influenced upon binding of the compounds. Regardless of the nature of these residues within the active site of complex, they were structurally stable (17). For the DHR1 co-crystallized complex, the RMSF values of the residues did not exceed 3.0 Å in both PDB and predicted protein (Figure 4l).

Finally, the interactions between proteins and ligands were investigated, including hydrogen bond, hydrophobic interaction, water bridges and ionic interaction. For the BRD4 co-crystallized complex, the binding residues for the ligand were ASN140 in PDB and ASN93 in Alphafold2-predicted structure. In both the PDB and Alphafold complexes simulation trajectory, it formed hydrogen bonds with GLN85 and CYS136, with good overlap (Figure 4m–n). Similar interaction residues and hydrogen bonds were observed in the DHR1 PDB and Alphafold2-predicted structure (Figure 4o–p).

## Discussion

At present, around 200 million known proteins are documented in UniProt (14). Each protein has a unique 3D structure. In contrast, there are only 180 000 structures, resolved by X-ray crystallography, NMR imaging and cryo-electron microscopy, documented in PDB (25). With the development of neural network-based prediction algorithms, including AlphaFold2 and RoseTTAFold, the predicted protein structures could be dramatically increased (1, 13). The AlphaFold Protein Structure Database (AlphaFold DB), created by DeepMind in collaboration with emBL-EBI, contains a set of 360 000 predicted structures of wildtype proteins covering most of the human and 20 other organisms derived from UniProt. Important advancements in RoseTTAFold algorithm development have been made in predictions of proteins with specific function. One notable example is a powerful three-dimensional model building for antibodies (26). Currently (2), there is still a lag in the experimentally determined mutant protein structures for many different diseases, which makes the investigation of biochemical mechanism of diseases stochastic and ineffective. On a global perspective in the human proteome, metrics built on the AlphaFold model can be used for predictions of multi-domain proteins and their alternations that are related to signaling pathway disorders (27). RoseTTAFold and AlphaFold, in deep learning modes, have been explored that are capable to predict protein structures affected from mutations, showing a potential to investigate associations and putative effects of gene mutations to development of diseases (28). We, for the first time, develop a protein structure prediction database for ocular disease genes (EyeProdb). Tertiary protein structure of gene variants for specific ocular diseases could be visualized directly in our platform, EyeProdb. It enables users searching on the web and submitting requests for specific amino acid sequences of genes related to ocular diseases. To aid advancing scientific discovery in ophthalmic structural and functional biology, EyeProdb is freely accessible to the scientific community for reliable, cost-effective and readily accessible protein prediction. DeepMind and the emBL-EBI have released the predicted structures for many known proteins (2), especially for a specific discipline. EyeProdb is more general in applications and can be interactive. Users with specialized or private sequence sources will expand the database of EyeProdb.

To validate the efficiency of our set-up of AlphaFold2 and RoseTTAFold algorithms for protein structure prediction, 46 proteins with different length of amino acids released in PDB after 1 September 2021 were applied for structural prediction evaluation. Compared to the released structures in PDB, we showed that both algorithms are efficient for structure prediction of protein based on the pLDDT and TM scores. On the contrary, the prediction might not be precise between mass without substantial mutual interaction. Molecular simulation investigations also indicted that predicted protein structures with higher pLDDT scores could be more reliable and were comparable to PDB structures.

Some open cloud-based platforms and Colab notebooks (29) can be conveniently used to predict the structure of a protein. These platforms adopted a simplified version of Alphafold2, by which multiple sequence alignments (MSA) and templates were not used. In order to maximize the prediction accuracy, we utilized the source code and trained models of Alphafold2 (1) and RoseTTAFold (15) in our on-premises data center to build of EyeProdb. This protein structure prediction platform, which combines the full functionality provided by Alphafold2 and RoseTTAFold, can be accessed through the internet.

There can be limitations in the AI-based prediction of protein structure for AlphaFold2 and RoseTTAFold. For instance, confidence scores can provide assessment methods for the predictions, which have also been shown in our highlighted cases. However, AlphaFold predictions with high confidence can be different from the experimentally resolved structures. Besides, for proteins with multiple conformations, AI algorithms cannot readily cope with proteins that can adopt different structures in different conformations (30). Similarly, though Humphreys *et al.* have reported a multistep bioinformatics and deep learning pipeline for identifying pairs of proteins likely to interact and modeling the three-dimensional structures of the corresponding protein complexes (31), Alphafold2 currently can only predict the structures of monomeric proteins or one of the chains of oligomeric proteins. The structures of complex oligomeric proteins cannot currently be predicted and assembled by AlphaFold at a single time. Protein dynamics could not be captured for ligands, such as DNA, RNA, other molecules and minerals. On the other hand, the AI algorithms can serve as ‘hypothesis generator’ to provide new information for experimental design and validation for biological investigations on gene or proteins for specific diseases without the experimentally resolved structures. For the proteins that experimental structures cannot be resolved, AI prediction is essential promising approach for further functional investigations. With the expansion of AlphaFold DB and variant databases, structure interpretation will be the basic and indispensable step for further mechanism studies.

One of the limitations of our study is that we only predict the structures of monomeric proteins, while other studies have also included polymers and protein–protein interactions. For example, a recent paper by Wang *et al.* (2023) used coevolution analysis and deep-learning–based structure modeling to identify and build models of core eukaryotic protein complexes in yeast. These interactions are important for understanding the biological functions and mechanisms of many eukaryotic proteins, especially in complex cellular processes. However, we believe that predicting the structures of monomers is still important, as they are the building blocks of larger assemblies and can provide insights into their folding and stability.

However, predicting the structures of protein complexes is more challenging than predicting the structures of monomers, as it requires accurate modeling of both the individual subunits and their interfaces. Moreover, the available data on protein–protein interactions is incomplete and noisy, and there may be many undiscovered interactions in the yeast proteome. Therefore, our ophthalmic protein database may not capture the full structural diversity and functional potential of the proteins in our target domain. To address this limitation, we plan to improve our database in the future by incorporating protein–protein interaction data from various sources, such as coevolution analysis, experimental assays and literature mining. We will also use state-of-the-art deep learning methods, such as RoseTTAFold and AlphaFold, to predict the structures of protein complexes from their amino acid sequences. By doing so, we hope to provide a more comprehensive and accurate resource for ophthalmic protein structure prediction and analysis.

The first release of EyeProdb contains over 1329 predicted structures for different types of ocular diseases. These will facilitate the biological investigations and structure-based drug development for ocular diseases. We are to expand EyeProdb for continuous provision and update of more predicted protein structures and their alterations due to gene mutations in ocular diseases. This will persistently enable effective drug design and development based on specific disease mechanisms affected by alternations in protein structures. EyeProdb is accessible to the whole scientific community, which is in line with open accessibility of research tools especially for protein structure and their alterations as exemplified by the development of ColabFold (32).

## Supplementary Material

baad083_Supp

## Data Availability

Datasets of genetic ocular genes and predicted protein structures are available at EyeProdb (http://eyeprodb.jsiec.org).

## Code availability

The source codes for protein structure prediction program, web application and database structure are available on Github: https://github.com/linchundan88/protein_folding/.
