# Supplementary material for: Artificial Intelligence-based database for prediction of protein structure and their alterations in ocular diseases
Source: Database (Oxford). 2023 Dec 18;2023:baad083. doi: 10.1093/database/baad083 (PMC10727695; doi:10.1093/database/baad083)
Supplement: baad083_Supp [file baad083_supp.zip › suppl_data/Suppl Table 1-genes.docx]

| **Gene** | **OMIM** | **Phenosubtype** |
| --- | --- | --- |
| ABCA1 | 600046 | Tangier disease, Tangier disease |
| ABCA4 | 601691 | Retinitis pigmentosa, Retinitis pigmentosa |
| ABCB6 | 605452 | Microphthalmia with coloboma 5 |
| ABCC6 | 603234 | Pseudoxanthoma elasticum |
| ABCD1 | 300371 | Peroxisome biogenesis disorder 2B |
| ABHD12 | 613599 | Retinitis pigmentosa, Syndromic cataract |
| ACBD5 | 616618 | Cone-rod retinal dystrophy |
| ACO2 | 100850 | Optic atrophy, Retinal syndrome |
| ACTA1 | 102610 | Myopathy, congenital, with fiber-type disproportion |
| ACTB | 102630 | Blepharophimosis, epicanthus inversus, and ptosis, type 1 |
| ACTG1 | 102560 | Baraitser-Winter syndrome 2 |
| ADAM9 | 602713 | Cone-rod dystrophy, Cone-rod retinal dystrophy |
| ADAMTS10 | 608990 | Weill-Marchesani syndrome, Weill-Marchesani syndrome |
| ADAMTS17 | 607511 | Weill-Marchesani 4 syndrome, recessive, Weill-Marchesani 4 syndrome, recessive, Weill-Marchesani 4 syndrome, recessive, Weill-Marchesani 4 syndrome, recessive |
| ADAMTS18 | 607512 | Chorioretinopathy, Choroid retinal atrophy, Microcornea, myopic chorioretinal atrophy, and telecanthus |
| ADAMTSL4 | 610113 | Ectopia lentis et pupillae, Ectopia lentis et pupillae, Ectopia lentis et pupillae |
| ADAR | 146920 | Aicardi-Goutieres syndrome 6 |
| ADD3 | 601568 | Cerebral palsy, spastic quadriplegic, 3 |
| AGBL1 | 615496 | Dystrophia endothelialis corneae, Corneal dystrophy, Fuchs endothelial, 8 |
| AGBL5 | 615900 | Retinitis pigmentosa 75 |
| AGK | 610345 | Congenital cataract and developmental cataract, Sengers syndrome |
| AGPS | 603051 | Rhizomelic chondrodysplasia punctata, type 3 |
| AGXT | 604285 | Hyperoxaluria, primary, type 1 |
| AHI1 | 608894 | Joubert syndrome |
| AIPL1 | 604392 | Leber congenital amaurosis, Leber congenital amaurosis, Leber congenital amaurosis |
| ALDH18A1 | 138250 | Cutis laxa, autosomal dominant 3, Cutis laxa, autosomal dominant 3 |
| ALDH3A2 | 609523 | Sjogren-Larsson syndrome |
| ALDOB | 612724 | Fructose intolerance, hereditary |
| ALMS1 | 606844 | Alstrom syndrome |
| ANO5 | 608662 | Macular dystrophy |
| ANTXR1 | 606410 | Optic atrophy |
| AP3B1 | 603401 | Hermansky-Pudlak syndrome |
| AP3B2 | 602166 | Developmental and epileptic encephalopathy 48 |
| APC | 611731 | Gardner syndrome |
| APOE | 107741 | Macular degeneration, age-related |
| APTX | 606350 | Ataxia, early-onset, with oculomotor apraxia and hypoalbuminemia |
| ARHGEF18 | 616432 | Retinitis pigmentosa 78 |
| ARL13B | 608922 | Joubert syndrome |
| ARL3 | 604695 | Retinitis pigmentosa |
| ARL6 | 608845 | Retinitis pigmentosa |
| ARMS2 | 611313 | Macular degeneration, age-related |
| ARR3 | 301770 | Myopia 26, X-linked, female-limited |
| ARSB | 611542 | Mucopolysaccharidosis type VI (Maroteaux-Lamy) |
| ARSG | 610008 | Usher syndrome, type IV |
| ASB10 | 615054 | Open-angle glaucoma, Glaucoma |
| ASPA | 608034 | Canavan disease |
| ATF6 | 605537 | Achromatopsia |
| ATM | 607585 | Ataxia-telangiectasia |
| ATOH7 | 609875 | Persistent hyperplastic primary vitreous, autosomal recessive |
| ATP13A2 | 610513 | Spastic paraplegia 78, autosomal recessive |
| ATP1A3 | 182350 | Optic atrophy, Alternating hemiplegia of childhood 2 |
| ATP2C1 | 604384 | Hailey-Hailey disease |
| ATP7B | 606882 | Wilson disease, Wilson disease |
| ATXN1 | 601556 | Spinocerebellar ataxia 1 |
| ATXN10 | 611150 | Spinocerebellar ataxia 10 |
| ATXN3 | 607047 | Machado-Joseph disease |
| ATXN7 | 607640 | Spinocerebellar ataxia 7 |
| BBS1 | 209901 | Bardet-Biedl syndrome |
| BBS10 | 610148 | Bardet-Biedl syndrome |
| BBS12 | 610683 | Bardet-Biedl syndrome |
| BBS2 | 606151 | Bardet-Biedl syndrome, Retinitis pigmentosa |
| BBS4 | 600374 | Bardet-Biedl syndrome |
| BBS5 | 603650 | Bardet-Biedl syndrome |
| BBS7 | 607590 | Bardet-Biedl syndrome |
| BBS9 | 615986 | Bardet-Biedl syndrome |
| BCOR | 300485 | Microphthalmia, Microphthalmia, Microphthalmia, Microphthalmia |
| BEST1 | 607854 | Vitreoretinochoroidopathy, Vitreoretinochoroidopathy, Retinitis pigmentosa, Vitreoretinochoroidopathy |
| BFSP1 | 603307 | Congenital cataract and developmental cataract |
| BFSP2 | 603212 | Cataract, Congenital cataract and developmental cataract |
| BGN | 301870 | Meester-Loeys syndrome |
| BMP4 | 112262 | Microphthalmia, Microphthalmia |
| BRPF1 | 602410 | Intellectual developmental disorder with dysmorphic facies and ptosis |
| C12orf57 | 615140 | Temtamy syndrome, Temtamy syndrome, Temtamy syndrome |
| C2orf71 | 613425 | Retinitis pigmentosa |
| C3 | 120700 | Macular degeneration, age-related |
| C5AR2 | 609949 | Retinitis pigmentosa |
| C8orf37 | 614477 | Cone-rod dystrophy, Cone-rod retinal dystrophy |
| C9 | 120940 | Macular degeneration, age-related |
| CA4 | 114760 | Retinitis pigmentosa |
| CABP4 | 608965 | Congenital static night blindness, Congenital static night blindness, 2B |
| CACNA1A | 601011 | Spinocerebellar ataxia 6 |
| CACNA1F | 300110 | Cone-rod retinal dystrophy, Cone-rod dystrophy, X-linked, 3, Congenital static night blindness, Cone-rod dystrophy, Aland Island eye disease |
| CACNA1G | 604065 | Spinocerebellar ataxia 42 |
| CACNA2D4 | 608171 | Retinal cone dystrophy |
| CAMK2A | 114078 | Mental retardation, autosomal dominant 53 |
| CAPN3 | 114240 | Muscular dystrophy, limb-girdle |
| CASK | 300172 | Intellectual developmental disorder and microcephaly with pontine and cerebellar hypoplasia |
| CAV1 | 601047 | Lipodystrophy, familial partial, type 7 |
| CAV3 | 601253 | Muscular dystrophy, limb-girdle |
| CBS | 613381 | Homocystinuria, B6-responsive and nonresponsive types |
| CC2D2A | 612013 | COACH syndrome, Meckel syndrome, COACH syndrome |
| CCDC28B | 610162 | Bardet-Biedl syndrome 1 |
| CDC42 | 116952 | Takenouchi-Kosaki syndrome |
| CDCA7 | 609937 | Immunodeficiency-centromeric instability-facial anomalies syndrome 3, Immunodeficiency-centromeric instability-facial anomalies syndrome 3, Immunodeficiency-centromeric instability-facial anomalies syndrome 3 |
| CDH1 | 192090 | Blepharocheilodontic syndrome 1 |
| CDH11 | 600023 | Elsahy-Waters syndrome |
| CDH23 | 605516 | Usher syndrome, type 1D/F digenic |
| CDH3 | 114021 | Macular dystrophy |
| CDHR1 | 609502 | Cone-rod retinal dystrophy, Cone-rod dystrophy |
| CDK10 | 603464 | Al Kaissi syndrome |
| CDK13 | 603309 | Congenital heart defects, dysmorphic facial features, and intellectual developmental disorder |
| CENPF | 600236 | Stromme syndrome, Stromme syndrome, Stromme syndrome |
| CEP164 | 614848 | Nephronophthisis |
| CEP290 | 610142 | Meckel syndrome, Leber congenital amaurosis, Leber congenital amaurosis 10, Leber congenital amaurosis |
| CEP41 | 610523 | Joubert syndrome 15 |
| CERKL | 608381 | Retinitis pigmentosa |
| CFH | 134370 | Macular degeneration, age-related, Basal laminar drusen |
| CFI | 217030 | Macular degeneration, age-related |
| CFL2 | 601443 | Nemaline myopathy 7, autosomal recessive, Nemaline myopathy, autosomal recessive |
| CHD7 | 608892 | CHARGE syndrome, CHARGE syndrome, CHARGE syndrome |
| CHM | 300390 | Choroideremia, Choroideremia |
| CHMP4B | 610897 | Congenital cataract and developmental cataract |
| CHN1 | 118423 | Duane retraction syndrome 2, Congenital fibrosis of the extraocular muscles |
| CHST6 | 605294 | Macular corneal dystrophy, Corneal dystrophy |
| CIB2 | 605564 | Usher syndrome |
| CISD2 | 611507 | Wolfram syndrome |
| CKAP2L | 616174 | Filippi syndrome |
| CLDN10 | 617579 | HELIX syndrome |
| CLDN19 | 610036 | Hypomagnesemia 5, renal, with ocular involvement, Hypomagnesemia, renal, with ocular involvement |
| CLN3 | 607042 | Ceroid lipofuscinosis, neuronal |
| CLN5 | 608102 | Ceroid lipofuscinosis, neuronal |
| CLN6 | 606725 | Ceroid lipofuscinosis, neuronal |
| CLN8 | 607837 | Ceroid lipofuscinosis, neuronal |
| CLRN1 | 606397 | Usher syndrome |
| CNBP | 116955 | Myotonic dystrophy 2 |
| CNGA1 | 123825 | Retinitis pigmentosa |
| CNGA3 | 600053 | Achromatopsia |
| CNGB1 | 600724 | Retinitis pigmentosa |
| CNGB3 | 605080 | Achromatopsia |
| CNNM4 | 607805 | Jalili syndrome |
| COL11A1 | 120280 | Marshall syndrome, Stickler syndrome, type II |
| COL11A2 | 120290 | Stickler syndrome, type III |
| COL18A1 | 120328 | Knobloch syndrome, type 1, Knobloch syndrome, type 1, Knobloch syndrome, type 1 |
| COL2A1 | 120140 | Stickler syndrome, Kniest dysplasia |
| COL4A1 | 120130 | Angiopathy, hereditary, with nephropathy, aneurysms, and muscle cramps |
| COL6A1 | 120220 | Ullrich congenital muscular dystrophy, Ullrich congenital muscular dystrophy 1 |
| COL6A2 | 120240 | Ullrich congenital muscular dystrophy, Ullrich congenital muscular dystrophy 1 |
| COL6A3 | 120250 | Ullrich congenital muscular dystrophy 1, Ullrich congenital muscular dystrophy 1 |
| COL7A1 | 120120 | Epidermolysis bullosa dystrophica, AR |
| COL8A2 | 120252 | Corneal endothelial dystrophy, Corneal dystrophy, Corneal dystrophy, Fuchs endothelial, 1 |
| COL9A1 | 120210 | Stickler syndrome |
| COL9A2 | 120260 | Stickler syndrome |
| CPAMD8 | 608841 | Anterior segment dysgenesis 8 |
| CRB1 | 604210 | Choroidal retinopathy, Leber congenital amaurosis, Leber congenital amaurosis, Retinitis pigmentosa, Leber congenital amaurosis |
| CREBBP | 600140 | Rubinstein-Taybi syndrome 1, Rubinstein-Taybi syndrome 1, Rubinstein-Taybi syndrome 1 |
| CRTAP | 605497 | Osteogenesis imperfecta, type VII |
| CRX | 602225 | Leber congenital amaurosis, Leber congenital amaurosis, Leber congenital amaurosis |
| CRYAA | 123580 | Cataract 9, Cataract, Cataract |
| CRYAB | 123590 | Congenital cataract and developmental cataract |
| CRYBA1 | 123610 | Congenital cataract and developmental cataract |
| CRYBA4 | 123631 | Cataract 23, Congenital cataract and developmental cataract |
| CRYBB1 | 600929 | Congenital cataract and developmental cataract |
| CRYBB2 | 123620 | Congenital cataract and developmental cataract |
| CRYBB3 | 123630 | Congenital cataract and developmental cataract, Cataract |
| CRYGA | 123660 | Cataract |
| CRYGB | 123670 | Congenital cataract and developmental cataract |
| CRYGC | 123680 | Albinism, oculocutaneous, type III, Cataract, Congenital cataract and developmental cataract |
| CRYGD | 123690 | Congenital cataract and developmental cataract |
| CRYGS | 123730 | Congenital cataract and developmental cataract |
| CST3 | 604312 | Macular degeneration, age-related |
| CTC1 | 613129 | Cerebroretinal microangiopathy with calcifications and cysts |
| CTDP1 | 604927 | Congenital cataracts, facial dysmorphism, and neuropathy |
| CTNNA1 | 116805 | Macular dystrophy, butterfly-shaped pigmentary, 2 |
| CTNNB1 | 116806 | Exudative vitreoretinopathy 7 |
| CTNND1 | 601045 | Blepharocheilodontic syndrome 2 |
| CTNND2 | 604275 | high myopia |
| CTNS | 606272 | Cystinosis, nephropathic |
| CTSD | 116840 | Ceroid lipofuscinosis, neuronal |
| CWC27 | 617170 | Retinitis pigmentosa with or without skeletal anomalies |
| CX3CR1 | 601470 | Macular degeneration, age-related |
| CYP1B1 | 601771 | Congenital central leukoplakia of cornea |
| CYP27A1 | 606530 | Cerebrotendinous xanthomatosis |
| CYP4V2 | 608614 | Bietti crystalline corneoretinal dystrophy, Bietti crystalline corneoretinal dystrophy |
| CYP7B1 | 603711 | Spastic paraplegia 5A, autosomal recessive |
| DAB1 | 603448 | Spinocerebellar ataxia 37 |
| DCC | 120470 | Gaze palsy, familial horizontal, with progressive scoliosis, 2 |
| DCN | 125255 | Corneal dystrophy, congenital stromal, Corneal dystrophy, congenital stromal, Corneal dystrophy, congenital stromal |
| DDX58 | 609631 | Singleton-Merten syndrome 2 |
| DGUOK | 601465 | Mitochondrial DNA depletion syndrome 3 (hepatocerebral type) |
| DHCR7 | 602858 | Smith-Lemli-Opitz syndrome |
| DHDDS | 608172 | Retinitis pigmentosa |
| DIP2B | 611379 | Optic neuritis |
| DKC1 | 300126 | Dyskeratosis congenita, X-linked |
| DMD | 300377 | Duchenne muscular dystrophy |
| DMPK | 605377 | Myotonic dystrophy |
| DNAJC5 | 611203 | Ceroid lipofuscinosis, neuronal |
| DNM1L | 603850 | Encephalopathy, lethal, due to defective mitochondrial peroxisomal fission 1 |
| DPAGT1 | 191350 | Congenital disorder of glycosylation, type Ij |
| DPH1 | 603527 | Developmental delay with short stature, dysmorphic facial features, and sparse hair |
| DRD5 | 126453 | Blepharospasm, primary benign |
| DTNBP1 | 607145 | Hermansky-Pudlak syndrome 7 |
| DUX4 | 606009 | Fascioscapulohumeral muscular dystrophy 2, digenic, Fascioscapulohumeral muscular dystrophy 2, digenic |
| DYSF | 603009 | Muscular dystrophy, limb-girdle, autosomal recessive 2 |
| ECHS1 | 602292 | Mitochondrial short-chain enoyl-CoA hydratase 1 deficiency |
| EDNRA | 131243 | Mandibulofacial dysostosis with alopecia |
| EFEMP1 | 601548 | Retinal dystrophy |
| EIF2B | 606686 | Leukoencephalopathy with vanishing white matter |
| ELN | 130160 | Williams-Beuren syndrome |
| ELOVL4 | 605512 | Spinocerebellar ataxia 34, Stargardt disease |
| ELOVL5 | 611805 | Spinocerebellar ataxia 38 |
| EMC1 | 616846 | Cerebellar atrophy, visual impairment, and psychomotor retardation |
| EMD | 300384 | Emery-Dreifuss muscular dystrophy 1, X-linked, Emery-Dreifuss muscular dystrophy, X-linked |
| EP300 | 602700 | Rubinstein-Taybi syndrome 2 |
| EPG5 | 615068 | Vici syndrome |
| EPHA2 | 176946 | Cataract, Cataract |
| EPRS | 138295 | Leukodystrophy, hypomyelinating, 15 |
| ERBB3 | 190151 | DOOR syndrome |
| ERCC2 | 126340 | Cerebrooculofacioskeletal syndrome, Cockayne syndrome |
| ERCC3 | 133510 | Cockayne syndrome |
| ERCC4 | 133520 | Fanconi anemia, complementation group Q |
| ERCC5 | 133530 | Cockayne syndrome, Cerebrooculofacioskeletal syndrome |
| ERCC6 | 609413 | Cerebrooculofacioskeletal syndrome, Macular degeneration, age-related, Cockayne syndrome |
| ERCC8 | 609412 | Cockayne syndrome |
| ESCO2 | 609353 | Roberts-SC phocomelia syndrome |
| EXOSC2 | 602238 | Short stature, hearing loss, retinitis pigmentosa, and distinctive facies |
| EYA1 | 601653 | Branchiootorenal syndrome |
| EYS | 612424 | Retinitis pigmentosa |
| FAM111A | 615292 | Kenny-Caffey syndrome, type 2 |
| FAM126A | 610531 | Leukodystrophy, hypomyelinating, 5 |
| FAM161A | 613596 | Retinitis pigmentosa |
| FAR1 | 616107 | Peroxisomal fatty acyl-CoA reductase 1 disorder |
| FBLN5 | 604580 | Macular degeneration, age-related |
| FBN1 | 134797 | Ectopia lentis, familial |
| FBN2 | 612570 | Macular degeneration, early-onset |
| FGF12 | 601513 | Developmental and epileptic encephalopathy 47 |
| FGFR1 | 136350 | Encephalocraniocutaneous lipomatosis, somatic mosaic |
| FGFR2 | 176943 | Apert syndrome, Jackson-Weiss syndrome, |
| FGFR3 | 134934 | LADD syndrome, Crouzon syndrome with acanthosis nigricans |
| FHL1 | 300163 | Emery-Dreifuss muscular dystrophy 6, X-linked, Emery-Dreifuss muscular dystrophy 6, X-linked |
| FKRP | 606596 | Muscular dystrophy-dystroglycanopathy (congenital with brain and eye anomalies), type A, 5 |
| FKTN | 607440 | Walker Warburg syndrome |
| FLVCR1 | 609144 | Ataxia, posterior column, with retinitis pigmentosa |
| FOXC1 | 601090 | Axenfeld-Rieger syndrome |
| FOXD3 | 611539 | Aniridia |
| FOXE3 | 601094 | Peters, abnormal, Anterior segment dysgenesis 1, multiple subtypes, Coloboma lentis, Anterior segment dysgenesis, Cataract 34 |
| FOXL2 | 605597 | Palpebral fissure syndrome, Blepharophimosis, epicanthus inversus, and ptosis, type 1, Baraitser-Winter syndrome |
| FRAS1 | 607830 | Fraser syndrome 1 |
| FREM1 | 608944 | Manitoba oculotrichoanal syndrome |
| FREM2 | 608945 | Fraser syndrome 1 |
| FRMD7 | 300628 | Nystagmus |
| FSCN2 | 607643 | Retinitis pigmentosa |
| FTL | 134790 | Hyperferritinemia-cataract syndrome |
| FTO | 610966 | Growth retardation, developmental delay, facial dysmorphism |
| FUCA1 | 612280 | Fucosidosis |
| FXN | 606829 | Friedreich ataxia |
| FYCO1 | 607182 | Congenital cataract and developmental cataract |
| FZD4 | 604579 | Retinopathy of prematurity, Familial exudative vitreoretinopathy |
| GABRB2 | 600232 | Developmental and epileptic encephalopathy 92 |
| GALC | 606890 | Krabbe disease, atypical |
| GALK1 | 604313 | Galactokinase deficiency with cataracts |
| GALNS | 612222 | Morquio syndrome |
| GALT | 606999 | Galactosemia |
| GBA2 | 609471 | Spastic paraplegia 46, autosomal recessive |
| GCNT2 | 600429 | Congenital cataract and developmental cataract |
| GDF3 | 606522 | Microphthalmia, Microphthalmia, Microphthalmia, Microphthalmia, Microphthalmia |
| GDF6 | 601147 | Microphthalmia, Leber congenital amaurosis, Leber congenital amaurosis, Microphthalmia, Leber congenital amaurosis, Microphthalmia |
| GJA1 | 121014 | Hallermann-streiff syndrome |
| GJA3 | 121015 | Congenital cataract and developmental cataract |
| GJA8 | 600897 | Cataract 1 |
| GJB1 | 304040 | Charcot-Marie-Tooth neuropathy, X-linked dominant, 1 |
| GJB2 | 121011 | Keratitis-ichthyosis-deafness syndrome |
| GLA | 300644 | Fabry disease |
| GLB1 | 611458 | Morquio syndrome |
| GLI2 | 165230 | Holoprosencephaly 9 |
| GNAS | 139320 | Pseudohypoparathyroidism Ia, Pseudohypoparathyroidism Ia |
| GNAT1 | 139330 | Congenital static night blindness, Night blindness, congenital stationary, autosomal dominant 3 |
| GNAT2 | 139340 | Achromatopsia |
| GNB3 | 139130 | Congenital static night blindness |
| GNPAT | 602744 | Rhizomelic chondrodysplasia punctata |
| GNPTG | 607838 | Mucolipidosis, Mucopolysaccharidosis |
| GP1BA | 606672 | Nonarteritic anterior ischemic optic neuropathy, susceptibility to |
| GPR143 | 300808 | Ocular albinism, type I, Nettleship-Falls type |
| GPR179 | 614515 | Night blindness, congenital stationary (complete), 1E, autosomal recessive |
| GRHL2 | 608576 | Corneal dystrophy, posterior polymorphous, 4 |
| GRIA4 | 138246 | Neurodevelopmental disorder with or without seizures and gait abnormalities, Neurodevelopmental disorder with or without seizures and gait abnormalities |
| GRID2 | 602368 | Spinocerebellar ataxia, autosomal recessive 18 |
| GRIP1 | 604597 | Fraser syndrome 3 |
| GRK1 | 180381 | Oguchi disease-2, Congenital static night blindness |
| GRM6 | 604096 | Congenital static night blindness, Night blindness, congenital stationary (complete), 1B, autosomal recessive |
| GRN | 138945 | Ceroid lipofuscinosis, neuronal |
| GSN | 137350 | Amyloidosis, Finnish type |
| GUCA1A | 600364 | Cone-rod retinal dystrophy |
| GUCA1B | 602275 | Retinitis pigmentosa |
| GUCY2D | 600179 | Leber congenital amaurosis, Leber congenital amaurosis |
| GUSB | 611499 | Mucolipidosis, Mucopolysaccharidosis |
| GZF1 | 613842 | Joint laxity, short stature, and myopia |
| HACE1 | 610876 | Spastic paraplegia and psychomotor retardation with or without seizures, Spastic paraplegia and psychomotor retardation with or without seizures, Spastic paraplegia and psychomotor retardation with or without seizures |
| HARS | 142810 | Usher syndrome |
| HBB | 141900 | Sickle cell anemia |
| HCCS | 300056 | Microphthalmia |
| HESX1 | 601802 | Septooptic dysplasia |
| HEXA | 606869 | Tay-Sachs disease |
| HEXB | 606873 | Sandhoff disease, infantile, juvenile, and adult forms |
| HFE | 613609 | Cataract 47, juvenile, with microcornea |
| HGD | 607474 | Alkaptonuria |
| HGSNAT | 610453 | Mucolipidosis, Mucopolysaccharidosis, Retinitis pigmentosa |
| HK1 | 142600 | Retinitis pigmentosa 79 |
| HMX1 | 142992 | Oculoauricular syndrome, Kahrizi syndrome, Microcephaly with or without chorioretinopathy, lymphedema, or mental retardation |
| HPS1 | 604982 | Hermansky-Pudlak syndrome 1 |
| HPS3 | 606118 | Hermansky-Pudlak syndrome 3 |
| HPS4 | 606682 | Hermansky-Pudlak syndrome 4 |
| HPS5 | 607521 | Hermansky-Pudlak syndrome |
| HPS6 | 607522 | Hermansky-Pudlak syndrome 6 |
| HSF4 | 602438 | Congenital cataract and developmental cataract |
| HTRA1 | 602194 | Macular degeneration, age-related |
| IARS2 | 612801 | Cataracts, growth hormone deficiency, sensory neuropathy, sensorineural hearing loss, and skeletal dysplasia |
| IDH3B | 604526 | Retinitis pigmentosa |
| IDS | 300823 | Mucopolysaccharidosis II |
| IDUA | 252800 | Mucolipidosis, Mucopolysaccharidosis |
| IFIH1 | 606951 | Singleton-Merten syndrome 1 |
| IFNGR1 | 107470 | Cataract |
| IFT140 | 614620 | jeune syndrome, Retinitis pigmentosa 80 |
| IFT172 | 607386 | Retinitis pigmentosa |
| IFT43 | 614068 | Retinitis pigmentosa 81 |
| IGBP1 | 300139 | Papillorenal syndrome |
| IMPDH1 | 146690 | Leber congenital amaurosis, Leber congenital amaurosis, Retinitis pigmentosa, Leber congenital amaurosis |
| IMPG1 | 602870 | Macular dystrophy, vitelliform, 4 |
| IMPG2 | 607056 | Retinitis pigmentosa |
| INPP5E | 613037 | Joubert syndrome |
| INPP5K | 607875 | Muscular dystrophy, congenital, with cataracts and intellectual disability |
| INVS | 243305 | Nephronophthisis |
| IQCB1 | 609237 | Senior-Loken syndrome |
| IRX5 | 606195 | Microphthalmia, syndromic, Hamamy syndrome |
| ISCA2 | 615317 | Multiple mitochondrial dysfunctions syndrome 4 |
| ITGA2B | 607759 | Retina |
| ITGB3 | 173470 | Retina |
| ITM2B | 603904 | Retinal dystrophy, Retinitis pigmentosa, Lowe syndrome |
| JAG1 | 601920 | Alagille syndrome |
| JAM3 | 606871 | Hemorrhagic destruction of the brain, subependymal calcification, and cataracts |
| KANK2 | 614610 | Palmoplantar keratoderma and woolly hair |
| KCNJ13 | 603208 | Snowflake vitreoretinal degeneration |
| KCNV2 | 607604 | Retinal cone dystrophy, Cone-rod dystrophy, Retinal cone dystrophy 3B |
| KCTD7 | 611725 | Epilepsy, progressive myoclonic 3, with or without intracellular inclusions |
| KDM6A | 300128 | Jackson-Weiss syndrome |
| KERA | 603288 | Corneal abnormalities |
| KIDINS220 | 615759 | Spastic paraplegia, intellectual disability, nystagmus, and obesity |
| KIF11 | 148760 | Microcephaly with or without chorioretinopathy, lymphedema, or mental retardation, Microcephaly with or without chorioretinopathy, lymphedema, or mental retardation, Microcephaly with or without chorioretinopathy, lymphedema, or mental retardation |
| kif14 | 611279 | Microcephaly 20, primary, autosomal recessive |
| KIF1C | 603060 | Spastic ataxia 2, autosomal recessive |
| KIF21A | 608283 | Congenital fibrosis of the extraocular muscles |
| KIF7 | 611254 | Joubert syndrome |
| KLC2 | 611729 | Spastic paraplegia, optic atrophy, and neuropathy |
| KLHL7 | 611119 | Retinitis pigmentosa |
| KMT2D | 602113 | Kabuki syndrome 1 |
| KRT12 | 601687 | Meesmann corneal dystrophy 1 |
| KRT3 | 148043 | Corneal dystrophy |
| LAMA1 | 150320 | Poretti - Boltshauser syndrome, Poretti-Boltshauser syndrome |
| LAMA2 | 156225 | Muscular dystrophy, congenital, merosin deficient or partially deficient |
| LAMB2 | 150325 | Pierson syndrome |
| LAMP2 | 309060 | Danon disease |
| LCA5 | 611408 | Leber congenital amaurosis, Leber congenital amaurosis, Leber congenital amaurosis |
| LCAT | 606967 | Fish-eye disease |
| LCT | 603202 | Cataract |
| LEMD2 | 616312 | Cataract 46, juvenile-onset |
| LEPREL1 | 610341 | Myopia, high, with cataract and vitreoretinal degeneration |
| LIM2 | 154045 | Cataract, Congenital cataract and developmental cataract |
| LMNA | 150330 | Hutchinson-Gilford progeria |
| LMOD3 | 616112 | Nemaline myopathy 10 |
| LMX1B | 602575 | Nail-patella syndrome |
| LOXHD1 | 613072 | Fuchs corneal endothelial dystrophy, Fuchs endothelial corneal dystrophy |
| LOXL1 | 153456 | Exfoliative glaucoma |
| LRAT | 604863 | Leber congenital amaurosis, Leber congenital amaurosis, Leber congenital amaurosis |
| LRIT3 | 615004 | Congenital static night blindness, Night blindness, congenital stationary (complete), 1F, autosomal recessive |
| LRP2 | 600073 | Donnai-Barrow syndrome |
| LRP5 | 603506 | Familial exudative vitreoretinopathy |
| LTBP2 | 602091 | Weill-Marchesani syndrome |
| LYST | 606897 | Chediak-Higashi syndrome |
| LZTFL1 | 606568 | Bardet-Biedl syndrome |
| MAF | 177075 | Cataract, Cataract 21, Cataracts, congenital, deafness, short stature, developmental delay, Congenital cataract and developmental cataract, Ayme-Gripp syndrome |
| MAFB | 608968 | Duane retraction syndrome 3 |
| MAG | 159460 | Spastic paraplegia 75, autosomal recessive |
| MAK | 154235 | Retinitis pigmentosa |
| MAN2B1 | 609458 | Mannosidosis, alpha- |
| MANBA | 609489 | Nystagmus, Mannosidosis, beta |
| MAPT | 157140 | Supranuclear palsy, progressive |
| MBTPS2 | 300294 | IFAP syndrome with or without BRESHECK syndrome |
| MC1R | 155555 | Albinism, oculocutaneous, type II |
| MCOLN1 | 605248 | Mucolipidosis IV, Mucolipidosis, Mucopolysaccharidosis |
| MECR | 608205 | Dystonia, childhood-onset, with optic atrophy and basal ganglia abnormalities |
| MED25 | 610197 | Basel-Vanagait-Smirin-Yosef syndrome |
| MERTK | 604705 | Retinitis pigmentosa |
| MFF | 614785 | Encephalopathy due to defective mitochondrial and peroxisomal fission 2 |
| MFN2 | 608507 | Charcot-Marie-Tooth disease, axonal, type 2A |
| MFRP | 606227 | Microphthalmia, Microphthalmia, Microphthalmia, Microphthalmia, Microphthalmia, Microphthalmia |
| MFSD8 | 611124 | Macular dystrophy, Ceroid lipofuscinosis, neuronal, Macular degeneration, age-related |
| MIP | 154050 | Congenital cataract and developmental cataract |
| MIR184 | 613146 | EDICT syndrome |
| MITF | 156845 | Oculocutaneous albinism, type IB albinism, yellow mutant type ywllow albinism, Waardenburg syndrome without dystopia canthorum |
| MKKS | 604896 | Bardet-Biedl syndrome |
| MKS1 | 609883 | Meckel syndrome |
| MLPH | 606526 | Griscelli syndrome, type 3 |
| MPDZ | 603785 | Hydrocephalus, congenital, 2, with or without brain or eye anomalies |
| MPV17 | 137960 | Mitochondrial DNA depletion syndrome 6 (hepatocerebral type) |
| MRPS34 | 611994 | Combined oxidative phosphorylation deficiency 32 |
| MSTO1 | 617619 | Myopathy, mitochondrial, and ataxia, Myopathy, mitochondrial, and ataxia |
| MTATP6 | 516060 | Neuropathy, ataxia, and retinitis |
| MTHFR | 607093 | Homocystinuria due to MTHFR deficiency |
| MTM1 | 300415 | Myotubular myopathy, X-linked |
| MTTP | 157147 | Bassen Kornzweig syndrome, Syndromic retinitis pigmentosa |
| MVK | 251170 | Mevalonic aciduria, Mevalonic aciduria |
| MYCN | 164840 | Feingold syndrome 1 |
| MYH2 | 160740 | Proximal myopathy and ophthalmoplegia 3 |
| MYH7 | 160760 | Myopathy, myosin storage, autosomal dominant |
| MYH9 | 160775 | Fechtner Syndrome |
| MYMK | 615345 | Carey-Fineman-Ziter syndrome |
| MYO5A | 160777 | Griscelli syndrome, type 1 |
| MYO7A | 276903 | Usher syndrome |
| MYOC | 601652 | Primary open angle glaucoma, Townes-Brocks branchiootorenal-like syndrome, Cohen syndrome |
| MYOT | 604103 | Muscular dystrophy, limb-girdle |
| MYP1 | 310460 | Myopia-1 |
| NAA10 | 300013 | Microphthalmia, Microphthalmia |
| NALCN | 611549 | Hypotonia, infantile, with psychomotor retardation and characteristic facies 1 |
| NBAS | 608025 | Short stature, optic nerve atrophy, and Pelger-Huet anomaly |
| NDP | 300658 | Norrie disease |
| NEB | 161650 | Nemaline myopathy 2, autosomal recessive |
| NEU1 | 608272 | Sialidosis, type I |
| NF1 | 613113 | Neurofibromatosis, type 1 |
| NF2 | 607379 | Neurofibromatosis, type 2 |
| NHS | 300457 | Congenital cataract and developmental cataract |
| NLRP1 | 606636 | Dyskeratosis, hereditary benign intraepithelial |
| NLRP3 | 606416 | Keratoendothelitis fugax hereditaria |
| NMNAT1 | 608700 | Leber congenital amaurosis, Leber congenital amaurosis, Leber congenital amaurosis |
| NOD2 | 605956 | Rothmund-Thomson syndrome, type 2 |
| NOG | 602991 | Multiple synostoses syndrome 1 |
| NPC1 | 607623 | Niemann-Pick disease, type B |
| NPC2 | 601015 | Niemann-pick disease, type C2 |
| NPHP1 | 607100 | Senior-Loken syndrome, Joubert syndrome, Nephronophthisis |
| NPHP3 | 608002 | Meckel syndrome |
| NPHP4 | 607215 | Senior-Loken syndrome, Nephronophthisis |
| NR2E3 | 604485 | Enhanced S-cone syndrome, Retinitis pigmentosa |
| NR2F1 | 132890 | Optic atrophy |
| NRL | 162080 | Retinitis pigmentosa |
| NTF4 | 162662 | Glaucoma, Primary open angle glaucoma |
| NTRK2 | 600456 | Developmental and epileptic encephalopathy 58 |
| NYX | 300278 | Congenital static night blindness, Congenital static night blindness |
| OAT | 613349 | Gyrate atrophy of choroid and retina with or without ornithinemia, Gyrate atrophy of choroid and retina with or without ornithinemia |
| OCA2 | 611409 | Albinism, oculocutaneous, Albinism, oculocutaneous, Albinism, oculocutaneous |
| OCRL | 300535 | Itm2b-associated cerebral amyloid vascular disease type 2 |
| OFD1 | 300170 | Retinitis pigmentosa |
| OPA1 | 605290 | Optic atrophy, Behr syndrome, Optic atrophy plus syndrome, Normal tension glaucoma |
| OPA2 | 311050 | Optic atrophy 2, X-linked |
| OPA3 | 606580 | Optic atrophy, Optic atrophy 3 with cataract, Optic atrophy plus syndrome |
| OPN1SW | 613522 | Achromatopsia, Colorblindness, tritan |
| OPTN | 602432 | Glaucoma, Normal tension glaucoma, Primary open angle glaucoma |
| OTX2 | 600037 | Microphthalmia, Microphthalmia |
| P4HA2 | 600608 | Myopia 25, autosomal dominant |
| P4HB | 176790 | Cole-Carpenter syndrome 1 |
| PABPN1 | 602279 | Oculopharyngeal muscular dystrophy |
| PACS1 | 607492 | Schuurs-Hoeijmakers syndrome |
| PANK2 | 606157 | HARP syndrome, Pantothenate kinase-associated neurodegeneration |
| PAX2 | 167409 | Papillorenal syndrome |
| PAX3 | 606597 | Waardenburg syndrome |
| PAX6 | 607108 | Coloboma of optic nerve |
| PCDH15 | 605514 | Usher syndrome |
| PDE6A | 180071 | Retinitis pigmentosa |
| PDE6B | 180072 | Night blindness, congenital stationary, autosomal dominant 2, Congenital static night blindness, Retinitis pigmentosa |
| PDE6C | 600827 | Cone-rod retinal dystrophy |
| PDE6G | 180073 | Retinitis pigmentosa 57, Retinitis pigmentosa 57 |
| PDE6H | 601190 | Achromatopsia, Retinal cone dystrophy, Cone-rod dystrophy |
| PDZD7 | 612971 | Usher syndrome |
| PEX1 | 602136 | Heimler syndrome 1, Zellweger syndrome |
| PEX10 | 602859 | Zellweger syndrome |
| PEX11B | 603867 | Zellweger syndrome, Peroxisome biogenesis disorder |
| PEX12 | 601758 | Zellweger syndrome |
| PEX13 | 601789 | Zellweger syndrome |
| PEX14 | 601791 | Zellweger syndrome |
| PEX16 | 603360 | Peroxisome biogenesis disorder 8B |
| PEX19 | 600279 | Zellweger syndrome |
| PEX2 | 170993 | Zellweger syndrome |
| PEX26 | 608666 | Zellweger syndrome |
| PEX3 | 603164 | Zellweger syndrome |
| PEX5 | 600414 | Rhizomelic chondrodysplasia punctata, Zellweger syndrome |
| PEX6 | 601498 | Zellweger syndrome, Heimler syndrome 2 |
| PEX7 | 601757 | Zellweger syndrome, Rhizomelic chondrodysplasia punctata |
| PGK1 | 311800 | Phosphoglycerate kinase 1 deficiency |
| PHGDH | 606879 | Neu-Laxova syndrome 1 |
| PHOX2A | 602753 | Fibrosis of extraocular muscles, congenital, Congenital fibrosis of the extraocular muscles, Congenital fibrosis of the extraocular muscles |
| PHYH | 602026 | Refsum disease |
| PIGL | 605947 | Microphthalmia, isolated, with coloboma 10 |
| PIGY | 610662 | Hyperphosphatasia with mental retardation syndrome 6 |
| PIK3R1 | 171833 | SHORT syndrome |
| PIK3R5 | 611317 | Ataxia-oculomotor apraxia 3 |
| PIKFYVE | 609414 | Corneal fleck dystrophy |
| PITPNM3 | 608921 | Cone-rod retinal dystrophy, Cone-rod dystrophy |
| PITX2 | 601542 | Axenfeld-Rieger syndrome, Axenfeld-Rieger syndrome |
| PITX3 | 602669 | Cataract 11, Congenital cataract and developmental cataract, Anterior interstitial dysplasia, Peters, abnormal, Anterior segment dysgenesis |
| PLA2G5 | 601192 | Fleck retina, familial benign, Retinal spot |
| PLAA | 603873 | Neurodevelopmental disorder with progressive microcephaly, spasticity, and brain anomalies |
| PLP1 | 300401 | Spastic paraplegia 2, X-linked |
| PMM2 | 601785 | Congenital disorder of glycosylation, type Ia |
| PNKP | 605610 | Ataxia-oculomotor apraxia 4 |
| PNPLA6 | 603197 | Laurence-Moon syndrome, Boucher-Neuhauser syndrome |
| POLG | 174763 | Progressive external ophthalmoplegia, autosomal recessive 1, Progressive external ophthalmoplegia, autosomal dominant 1 |
| POLG2 | 604983 | Progressive external ophthalmoplegia with mitochondrial DNA deletions, autosomal dominant 4 |
| POLR1A | 616404 | Acrofacial dysostosis, Cincinnati type |
| POMGNT1 | 606822 | Limb-girdle muscular dystrophy, Muscular dystrophy-dystroglycanopathy (congenital with mental retardation), type B, 3, Walker Warburg syndrome, Retinitis pigmentosa, Muscular dystrophy-dystroglycanopathy (congenital with mental retardation) |
| POMT1 | 607423 | Muscular dystrophy-dystroglycanopathy (congenital with brain and eye anomalies), type A, 1 |
| POMT2 | 607439 | Walker Warburg syndrome |
| PORCN | 300651 | Focal dermal hypoplasia |
| PPT1 | 600722 | Ceroid lipofuscinosis, neuronal |
| PRCD | 610598 | Retinitis pigmentosa |
| PRDM5 | 614161 | Brittle cornea syndrome 2 |
| PRKCG | 176980 | Spinocerebellar ataxia |
| PROM1 | 604365 | Retinitis pigmentosa |
| PRPF3 | 607301 | Retinitis pigmentosa |
| PRPF31 | 606419 | Retinitis pigmentosa |
| PRPF6 | 613979 | Retinitis pigmentosa |
| PRPF8 | 607300 | Retinitis pigmentosa |
| PRPH2 | 179605 | Choroidal dystrophy |
| PRPS1 | 311850 | Arts syndrome |
| PRSS56 | 613858 | Microphthalmia, Microphthalmia, Microphthalmia, Microphthalmia, Microphthalmia |
| PSAT1 | 610936 | Neu-Laxova syndrome 2 |
| PTCH1 | 601309 | Basal cell nevus syndrome |
| PTPN11 | 176876 | LEOPARD syndrome 1 |
| PXDN | 605158 | Anterior segment dysgenesis 7, with sclerocornea, Anterior segment dysgenesis 7, with sclerocornea, Anterior segment dysgenesis 7, with sclerocornea |
| RAB18 | 602207 | Warburg micro syndrome 3, Warburg micro syndrome, Warburg micro syndrome |
| RAB23 | 606144 | Carpenter syndrome, Carpenter syndrome |
| RAB27A | 603868 | Choroideremia |
| RAB3GAP1 | 602536 | Warburg micro syndrome 1, Warburg micro syndrome 1, Warburg micro syndrome |
| RAB3GAP2 | 609275 | Warburg micro syndrome 2, Warburg micro syndrome 2 |
| RAB7A | 602298 | Charcot-Marie-Tooth disease, type 2B |
| RABGGTA | 601905 | Choroideremia, Choroideremia |
| RAI1 | 607642 | Smith-Magenis syndrome, Smith-Magenis syndrome, Smith-Magenis syndrome, Smith-Magenis syndrome |
| RAX | 601881 | Microphthalmia, Microphthalmia, Microphthalmia, Microphthalmia, Microphthalmia |
| RAX2 | 610362 | Macular degeneration, age-related, Cone-rod retinal dystrophy |
| RB1 | 614041 | Retinoblastoma |
| RBP3 | 180290 | Retinitis pigmentosa |
| RBP4 | 180250 | Microphthalmia, Microphthalmia, Microphthalmia |
| RCBTB1 | 607867 | Retinal dystrophy |
| RD3 | 180040 | Leber congenital amaurosis, Leber congenital amaurosis, Leber congenital amaurosis |
| RDH12 | 608830 | Leber congenital amaurosis, Leber congenital amaurosis, Leber congenital amaurosis |
| RDH5 | 601617 | White punctate fundus |
| RECQL4 | 603780 | Blau syndrome |
| REEP6 | 609346 | Retinitis pigmentosa 77 |
| RET | 164761 | Multiple endocrine neoplasia IIB |
| RGR | 600342 | Retinitis pigmentosa |
| RGS9 | 604067 | Bradyopsia, Bradyopsia |
| RGS9BP | 607814 | Bradyopsia, Bradyopsia |
| RHO | 180380 | Night blindness, congenital stationary, autosomal dominant 1, Retinitis pigmentosa, Retinitis punctata albescens, Congenital static night blindness |
| RIMS1 | 606629 | Cone-rod dystrophy, Cone-rod retinal dystrophy |
| RLBP1 | 180090 | Retinitis punctata albescens, Retinitis punctata albescens |
| RNF125 | 610432 | Tenorio syndrome |
| ROBO3 | 608630 | Gaze palsy, familial horizontal, with progressive scoliosis, 1 |
| ROM1 | 180721 | Retinitis pigmentosa |
| RP1 | 603937 | Retinitis pigmentosa |
| RP1L1 | 608581 | Occult macular dystrophy |
| RP2 | 300757 | Retinitis pigmentosa, Retinitis pigmentosa, Retinitis pigmentosa |
| RP9 | 607331 | Retinitis pigmentosa |
| RPE65 | 180069 | Leber congenital amaurosis, Retinitis pigmentosa, Leber congenital amaurosis |
| RPGR | 312610 | Retinitis pigmentosa, Retinitis pigmentosa |
| RPGRIP1 | 605446 | Leber congenital amaurosis, Leber congenital amaurosis, Leber congenital amaurosis, Cone-rod dystrophy |
| RPGRIP1L | 610937 | COACH syndrome 1, COACH syndrome 1, COACH syndrome 1 |
| RRM2B | 604712 | Progressive external ophthalmoplegia with mitochondrial DNA deletions, autosomal dominant 5 |
| RS1 | 300839 | Retinoschisis, Retinoschisis, Retinoschisis |
| RTN4IP1 | 610502 | Optic atrophy 10 |
| RYR1 | 180901 | Minicore myopathy with external ophthalmoplegia |
| SACS | 604490 | Spastic ataxia, Charlevoix-Saguenay type |
| SAG | 181031 | Oguchi disease-1, Oguchi disease-1 |
| SALL1 | 602218 | Glaucoma 1A, primary open angle |
| SALL4 | 607343 | Duane-radial ray syndrome |
| SAT1 | 313020 | Kaufman oculocerebrofacial syndrome |
| SBF2 | 607697 | Charcot-Marie-Tooth disease, type 4B2 |
| SC5D | 602286 | Lathosterolosis |
| SCO2 | 604272 | Myopia |
| SDCCAG8 | 613524 | Senior-Loken syndrome 7 |
| SDHA | 600857 | Leigh Syndrome |
| SEC23A | 610511 | Craniolenticulosutural dysplasia |
| SEC24D | 607186 | Cole-Carpenter syndrome 2 |
| SEMA4A | 607292 | Retinitis pigmentosa |
| SGCA | 600119 | Muscular dystrophy, limb-girdle |
| SGCB | 600900 | Limb-girdle muscular dystrophy |
| SGCD | 601411 | Macular degeneration |
| SGCG | 608896 | Muscular dystrophy, limb-girdle |
| SHH | 600725 | Microphthalmia, Microphthalmia, Microphthalmia, Microphthalmia |
| SIL1 | 608005 | Nail - patella syndrome |
| SIPA1L3 | 616655 | Cataract 45 |
| SIX5 | 600963 | Branchiootorenal syndrome |
| SIX6 | 606326 | Optic disc anomalies with retinal and/or macular dystrophy |
| SLC16A12 | 611910 | Diabetes, Corneal abnormality syndrome |
| SLC16A2 | 300095 | Allan-Herndon-Dudley syndrome |
| SLC24A1 | 603617 | Congenital static night blindness |
| SLC24A5 | 609802 | Albinism, oculocutaneous, type VI, Albinism, oculocutaneous, type VI, Albinism, oculocutaneous, type VI |
| SLC25A15 | 603861 | Hyperornithinemia-hyperammonemia-homocitrullinemia syndrome |
| SLC25A4 | 103220 | Progressive external ophthalmoplegia with mitochondrial DNA deletions, autosomal dominant 2 |
| SLC26A4 | 605646 | Retinitis pigmentosa |
| SLC2A1 | 138140 | Stomatin-deficient cryohydrocytosis with neurologic defects |
| SLC33A1 | 603690 | Syndromic cataract |
| SLC38A8 | 615585 | Foveal hypoplasia 2, with or without optic nerve misrouting and/or anterior segment dysgenesis |
| SLC45A2 | 606202 | Albinism, oculocutaneous, type IV |
| SLC4A11 | 610206 | Corneal endothelial dystrophy, Corneal endothelial dystrophy |
| SLC52A2 | 607882 | Brown-Vialetto-Van Laere syndrome 2 |
| SLC9A6 | 300231 | Intellectual developmental disorder, X-linked syndromic, Christianson type |
| SLITRK6 | 609681 | Myopic syndrome |
| SMCHD1 | 614982 | Bosma arhinia microphthalmia syndrome |
| SMG9 | 613176 | Heart and brain malformation syndrome |
| SMOC1 | 608488 | Microphthalmia with limb anomalies |
| SMPD1 | 607608 | Niemann-Pick disease, type B |
| SMS | 300105 | Myopia |
| SNAI2 | 602150 | Waardenburg syndrome, type 2D |
| SNRNP200 | 601664 | Retinitis pigmentosa |
| SNX3 | 605930 | Microphthalmia, syndromic 8 |
| SOD1 | 147450 | Macular degeneration |
| SON | 182465 | ZTTK syndrome, ZTTK syndrome |
| SOX10 | 602229 | Waardenburg syndrome |
| SOX18 | 601618 | Hypotrichosis-lymphedema-telangiectasia-renal defect syndrome |
| SOX2 | 184429 | Microphthalmia, Microphthalmia |
| SPATA7 | 609868 | Leber congenital amaurosis, Leber congenital amaurosis, Leber congenital amaurosis |
| SPG11 | 610844 | Spastic paraplegia 11, autosomal recessive, Spastic paraplegia 11, autosomal recessive |
| SPG7 | 602783 | Spastic paraplegia, autosomal recessive, Spastic paraplegia 7, autosomal recessive |
| SQSTM1 | 601530 | Neurodegeneration with ataxia, dystonia, and gaze palsy, childhood-onset |
| SRD5A3 | 611715 | Congenital disorder of glycosylation, type Iq |
| STRA6 | 610745 | Microphthalmia, Microphthalmia, Microphthalmia, Microphthalmia |
| SUOX | 606887 | Sulfite oxidase deficiency |
| SYNE1 | 608441 | Spinocerebellar ataxia, autosomal recessive 8 |
| SYNE2 | 608442 | Emery-Dreifuss muscular dystrophy 5, autosomal dominant, Emery-Dreifuss muscular dystrophy 5, autosomal dominant |
| SYT1 | 185605 | Baker-Gordon syndrome |
| TACSTD2 | 137290 | Corneal dystrophy, gelatinous drop-like |
| TAT | 613018 | Tyrosinemia, type II |
| TBC1D24 | 613577 | Hypomagnesemia 5, renal, with ocular involvement |
| TBC1D3 | 607741 | Pontocerebellar hypoplasia, type 11 |
| TBCD | 604649 | Encephalopathy, progressive, early-onset, with brain atrophy and thin corpus callosum |
| TBCE | 604934 | Encephalopathy, progressive, with amyotrophy and optic atrophy |
| TBCK | 616899 | Hypotonia, infantile, with psychomotor retardation and characteristic facies 3 |
| TBK1 | 604834 | Frontotemporal dementia and/or amyotrophic lateral sclerosis 4 |
| TBX1 | 602054 | DiGeorge syndrome |
| TCAP | 604488 | Muscular dystrophy, limb-girdle |
| TCF4 | 602272 | Corneal dystrophy, Fuchs endothelial, 3, Corneal endothelial dystrophy |
| TCOF1 | 606847 | Treacher Collins syndrome 1 |
| TCTN1 | 609863 | Joubert syndrome 13 |
| TCTN2 | 613846 | Joubert syndrome 24 |
| TCTN3 | 613847 | Joubert syndrome |
| TDRD7 | 611258 | Congenital cataract and developmental cataract, Cataract |
| TEAD1 | 189967 | Sveinsson chorioretinal atrophy, Sveinsson chorioretinal atrophy |
| TEK | 600221 | Congenital primary glaucoma |
| TFAP2A | 107580 | Branchiooculofacial syndrome, Branchiooculofacial syndrome, Branchiooculofacial syndrome |
| TGFB3 | 190230 | Loeys-Dietz syndrome 5 |
| TGFBI | 601692 | Corneal dystrophy, lattice, Corneal dystrophy, Reis-Bucklers type, Corneal dystrophy, Corneal dystrophy, Thiel-Behnke type, Corneal dystrophy, Groenouw |
| TGFBR1 | 190181 | Loeys-Dietz syndrome 1 |
| TGFBR2 | 190182 | Loeys-Dietz syndrome 2 |
| TIMM8A | 300356 | Mohr-Tranebjaerg syndrome |
| TIMP3 | 188826 | Sorsby fundus dystrophy |
| TINF2 | 604319 | Revesz syndrome |
| TK2 | 188250 | Progressive external ophthalmoplegia，autosomal recessive 3 |
| TLK2 | 608439 | Intellectual developmental disorder, autosomal dominant 57 |
| TLR3 | 603029 | Macular degeneration, age-related |
| TLR4 | 603030 | Macular degeneration, age-related |
| TMCO3 | 617134 | Cornea guttata with anterior polar cataracts |
| TMEM114 | 611579 | Cataract |
| TMEM126A | 612988 | Optic atrophy 7, Optic atrophy |
| TMEM138 | 614459 | Joubert syndrome |
| TMEM216 | 613277 | Meckel syndrome |
| TMEM231 | 614949 | Meckel syndrome |
| TMEM237 | 614423 | Joubert syndrome 14 |
| TMEM67 | 609884 | COACH syndrome, Meckel syndrome, COACH syndrome |
| TMEM70 | 612418 | Mitochondrial complex V (ATP synthase) deficiency, nuclear type 2 |
| TNFAIP3 | 191163 | Autoinflammatory syndrome, familial, Behcet-like |
| TNNT1 | 191041 | Nemaline myopathy 5, Amish type, Nemaline myopathy 5, Amish type |
| TOE1 | 613931 | Pontocerebellar hypoplasia, type 7 |
| TOPORS | 609507 | Retinitis pigmentosa |
| TPM2 | 190990 | Arthrogryposis, distal, type 2B1 |
| TPM3 | 191030 | Myopathy, congenital, with fiber-type disproportion |
| TPP1 | 607998 | Ceroid lipofuscinosis, neuronal |
| TRAPPC12 | 614139 | Encephalopathy, progressive, early-onset, with brain atrophy and spasticity |
| TRIM32 | 602290 | Bardet-Biedl syndrome |
| TRIM44 | 612298 | Aniridia 3 |
| TRNT1 | 612907 | Peeling skin syndrome 6 |
| TRPM1 | 603576 | Congenital static night blindness, Night blindness, congenital stationary (complete), 1C, autosomal recessive |
| TSC1 | 605284 | Tuberous sclerosis-1 |
| TSC2 | 191092 | Tuberous sclerosis-2 |
| TSPAN12 | 613138 | Spondylo-megaepiphyseal-metaphyseal dysplasia, Familial exudative vitreoretinopathy |
| TTC21B | 612014 | Nephronophthisis, jeune syndrome |
| TTC8 | 608132 | Retinitis pigmentosa |
| TTLL5 | 612268 | Cone-rod dystrophy 19 |
| TTN | 188840 | Salih myopathy |
| TTPA | 600415 | Ataxia with isolated vitamin E deficiency |
| TTR | 176300 | Other etiologies of vitreous lesions |
| TUBA3D | 617878 | Keratoconus 9 |
| TUBA8 | 605742 | Cerebral dysgenesis, neuropathy, ichthyosis, and palmoplantar keratoderma syndrome |
| TUBB3 | 602661 | Fibrosis of extraocular muscles, congenital |
| TUBB4B | 602660 | Leber congenital amaurosis with early-onset deafness |
| TUBGCP6 | 610053 | Chorioretinopathy |
| TULP1 | 602280 | Leber congenital amaurosis, Leber congenital amaurosis, Retinitis pigmentosa, Leber congenital amaurosis |
| TWIST1 | 601622 | Sweeney-Cox syndrome, Sweeney-Cox syndrome |
| TWIST2 | 607556 | Ablepharon-macrostomia syndrome |
| TXNL4A | 611595 | Burn-McKeown syndrome |
| TYMP | 131222 | Mitochondrial DNA depletion syndrome 1 (MNGIE type) |
| TYR | 606933 | Albinism, oculocutaneous, Albinism, oculocutaneous |
| TYRP1 | 115501 | Albinism, oculocutaneous |
| UBE3B | 608047 | Kabuki syndrome 2 |
| UBIAD1 | 611632 | Corneal dystrophy, Schnyder type, Corneal dystrophy |
| UCHL1 | 191342 | Spastic paraplegia 79, autosomal recessive, Optic atrophy |
| UNC119 | 604011 | Cone-rod retinal dystrophy |
| UNC45B | 611220 | Cataract 43 |
| UNC80 | 612636 | Hypotonia, infantile, with psychomotor retardation and characteristic facies 2 |
| USH1C | 605242 | Usher syndrome |
| USH1G | 607696 | Usher syndrome |
| USH2A | 608400 | Retinitis pigmentosa |
| USP9X | 300072 | Intellectual developmental disorder, X-linked 99, syndromic, female-restricted |
| VAX1 | 604294 | Microphthalmia, Microphthalmia |
| VCAN | 118661 | Achromatopsia 3 |
| VHL | 608537 | Pheochromocytoma, von Hippel-Lindau syndrome |
| VIM | 193060 | Congenital cataract and developmental cataract |
| VLDLR | 192977 | Cerebellar hypoplasia and mental retardation with or without quadrupedal locomotion 1 |
| VPS13B | 607817 | Cohen syndrome, Cohen syndrome |
| VSX1 | 605020 | Corneal dystrophy, posterior polymorphous, 1 |
| VSX2 | 142993 | Microphthalmia, Microphthalmia, Microphthalmia |
| WARS2 | 604733 | Neurodevelopmental disorder, mitochondrial, with abnormal movements and lactic acidosis, with or without seizures |
| WDPCP | 613580 | Bardet-Biedl syndrome |
| WDR19 | 608151 | jeune syndrome, Senior-Loken syndrome, Nephronophthisis |
| WDR36 | 609669 | Glaucoma, Primary open angle glaucoma |
| WDR73 | 616144 | Galloway-Mowat syndrome 1 |
| WFS1 | 606201 | Wolfram syndrome, Diabetes, Congenital cataract and developmental cataract |
| WRN | 277700 | Werner syndrome |
| WWOX | 605131 | Developmental and epileptic encephalopathy 28 |
| XYLT2 | 608125 | Spondyloocular syndrome |
| YME1L1 | 607472 | Optic atrophy 11 |
| YY1 | 600013 | Gabriele-de Vries syndrome |
| ZEB1 | 189909 | Corneal dystrophy, Fuchs endothelial, 6, Corneal dystrophy, Dystrophia endothelialis corneae |
| ZEB2 | 605802 | Mowat-Wilson syndrome |
| ZFYVE26 | 612012 | Spastic paraplegia 15, autosomal recessive |
| ZNF408 | 616454 | Retinitis pigmentosa, Exudative vitreoretinopathy 6 |
| ZNF423 | 604557 | Joubert syndrome, Nephronophthisis |
| ZNF469 | 612078 | Brittle cornea syndrome 1 |
| ZNF513 | 613598 | Retinitis pigmentosa |
| ZNF644 | 614159 | Myopia |
| ZNHIT3 | 604500 | PEHO syndrome |
