# Supplementary material for: Artificial Intelligence-based database for prediction of protein structure and their alterations in ocular diseases
Source: Database (Oxford). 2023 Dec 18;2023:baad083. doi: 10.1093/database/baad083 (PMC10727695; doi:10.1093/database/baad083)
Supplement: baad083_Supp [file baad083_supp.zip › suppl_data/Suppl Table 2-10 AlphaF.docx]

| Protein | Nature | TM-score | lDDT | GDT_TS score |
| --- | --- | --- | --- | --- |
| CISD2 | Wildtype | 0.91286 | 0.982197821 | 1.39 |
| TMEM216 | Wildtype | 0.95407 | 0.967840731 | 1.17 |
| SOD1 | Wildtype | 0.99943 | 0.989408076 | 0.11 |
| CFL2 | Wildtype | 0.9989 | 0.97527945 | 0.15 |
| LIM2 | Wildtype | 0.9962 | 0.980597079 | 0.35 |
| CRYGB | Wildtype | 0.99978 | 0.995129585 | 0.07 |
| ARL3 | Wildtype | 0.99888 | 0.97682941 | 0.17 |
| UCHL1 | Wildtype | 0.99115 | 0.930411458 | 0.55 |
| CLRN1 | Wildtype | 0.96838 | 0.957333624 | 1.11 |
| TPM3 | Wildtype | 0.95783 | 0.989558578 | 1.37 |
