# Supplementary material for: Artificial Intelligence-based database for prediction of protein structure and their alterations in ocular diseases
Source: Database (Oxford). 2023 Dec 18;2023:baad083. doi: 10.1093/database/baad083 (PMC10727695; doi:10.1093/database/baad083)
Supplement: baad083_Supp [file baad083_supp.zip › suppl_data/Suppl Table 3-48 detail.docx]

|  | **Organisms** | **Proteins** | **PDB ID** | **Size (amino acids)** | **TM-score (AlphaFold)** | **pLDDT (AlphaFold)** | **lDDT (AlphaFold)** | **GDT_TS-score (AlphaFold)** | **TM-score (RoseTTAFold)** | **lDDT (RoseTTAFold)** | **GDT_TS-score（Rose TTAFold）** |
| --- | --- | --- | --- | --- | --- | --- | --- | --- | --- | --- | --- |
| 1 | Saccharum hybrid cultivar SP80-3280 | Sugarwin | 7KSN | 124 | 0.971 | 96.788 | 0.473 | 0.93 | 0.943 | 0.412 | 1.22 |
| 2 | synthetic construct | RNASE 3/1 version3 | 6SSN | 130 | 0.964 | 96.063 | 0.445 | 0.88 | 0.924 | 0.393 | 1.35 |
| 3 | Homo sapiens | EDN mutant L45H | 6SSO | 135 | 0.997 | 97.805 | 0.956 | 0.24 | 0.964 | 0.851 | 0.89 |
| 4 | synthetic construct | RNASE 3/1 version2 | 6YBE | 136 | 0.934 | 95.411 | 0.843 | 1.27 | 0.891 | 0.773 | 1.63 |
| 5 | Homo sapiens | unliganded second bromodomain (BD2) of human TAF1 | 7K3O | 137 | 0.977 | 97.407 | 0.467 | 0.76 | 0.958 | 0.431 | 0.82 |
| 6 | Homo sapiens | BRD4 | 7DHS | 141 | 0.959 | 92.675 | 0.474 | 0.79 | 0.949 | 0.439 | 0.99 |
| 7 | Homo sapiens | ATAD2 bromodomain complexed with histone H4K5ac (res 1-10) ligand | 7M98 | 152 | 0.978 | 94.415 | 0.929 | 0.66 | 0.976 | 0.864 | 0.69 |
| 8 | Homo sapiens | ubiquitin-conjugating enzyme UBE2S L114E varaiant | 7AHF | 156 | 0.956 | 93.756 | 0.431 | 1.03 | 0.939 | 0.401 | 1.30 |
| 9 | Homo sapiens | ISG15 | 7S6P | 159 | 0.887 | 94.117 | 0.141 | 1.72 | 0.818 | 0.135 | 2.15 |
| 10 | Thermotoga maritima MSB8 | ferritin mutant-FLAL | 7DY9 | 164 | 0.986 | 98.241 | 0.442 | 0.52 | 0.975 | 0.403 | 0.77 |
| 11 | Homo sapiens | KRAS G12D Mutant in complex with GMPPCP and cyclic peptide MP-9903 | 7ROV | 189 | 0.930 | 92.855 | 0.429 | 1.23 | 0.934 | 0.406 | 1.40 |
| 12 | Influenza A virus (A/Luxembourg/43/2009(H1N1)) | PA endonuclease mutant I38T in complex with SJ001008025 | 7K77 | 197 | 0.972 | 92.373 | 0.915 | 0.49 | 0.952 | 0.814 | 0.99 |
| 13 | Homo sapiens | Retinoschisin | 3JD6 | 207 | 0.895 | 68.455 | 0.693 | 1.72 | 0.882 | 0.686 | 1.79 |
| 14 | Pseudomonas putida DOT-T1E | TtgR quadruple mutant (C137I I141W M167L F168Y) | 7K1A | 211 | 0.991 | 95.015 | 0.461 | 0.63 | 0.956 | 0.411 | 1.22 |
| 15 | Homo sapiens | LN02 Fab | 7AJ6 | 223 | 0.531 | 89.723 | 0.448 | 2.91 | 0.527 | 0.408 | 2.42 |
| 16 | Homo sapiens | Protocadherin-24 EC1-2 Form II | 7N86 | 238 | 0.917 | 92.650 | 0.229 | 1.27 | 0.894 | 0.198 | 1.66 |
| 17 | Aspergillus niger CBS 513.88 | chitin deacetylase AngCDA | 7BLY | 239 | 0.995 | 93.629 | 0.955 | 0.41 | 0.977 | 0.850 | 0.96 |
| 18 | Mycobacterium tuberculosis H37Rv | heterocyclic toxin methyltransferase (Rv0560c) | 7BGG | 245 | 0.946 | 88.484 | 0.877 | 1.01 | 0.937 | 0.798 | 1.47 |
| 19 | Pseudomonas aeruginosa PAO1 | SpuA native structure | 7D4R | 250 | 0.987 | 97.306 | 0.502 | 0.69 | 0.967 | 0.440 | 1.11 |
| 20 | Homo sapiens | truncated form of the KLC1-TPR domain ([A1-B5] fragment) | 7AIE | 255 | 0.840 | 86.503 | 0.200 | 2.05 | 0.874 | 0.200 | 1.83 |
| 21 | Mycobacterium tuberculosis | Beta-lactamase, Unmixed | 7K8L | 267 | 0.987 | 97.995 | 0.235 | 0.80 | 0.970 | 0.210 | 1.11 |
| 22 | Homo sapiens | SETD2 bound to Compound 2 | 7LZB | 278 | 0.980 | 89.683 | 0.911 | 0.92 | 0.945 | 0.777 | 1.45 |
| 23 | Mus musculus | 7-alpha-hydroxyl bile acid sulfotransferase (Sult2a8) | 7D1X | 284 | 0.954 | 91.554 | 0.416 | 1.41 | 0.904 | 0.364 | 1.93 |
| 24 | Escherichia coli | Dihydrodipicolinate synthase mutant S48F | 7JZ7 | 292 | 0.995 | 98.282 | 0.478 | 0.47 | 0.987 | 0.428 | 0.76 |
| 25 | Human coronavirus NL63 | 3C-like protease | 7E6M | 303 | 0.970 | 95.247 | 0.467 | 1.11 | 0.946 | 0.401 | 1.48 |
| 26 | Streptomyces lusitanus | SDR protein/resistance protein NapW | 7BTM | 331 | 0.991 | 92.151 | 0.116 | 0.62 | 0.922 | 0.095 | 1.65 |
| 27 | Chromohalobacter salexigens DSM 3043 | open conformation of the SBP TarP_Csal | 7NTE | 335 | 0.949 | 96.323 | 0.452 | 1.44 | 0.928 | 0.392 | 1.86 |
| 28 | Rattus norvegicus | Cannabinoid Receptor 1 Interacting Protein 1a (CRIP1a) | 6WSK | 346 | 0.595 | 89.761 | 0.848 | 1.32 | 0.482 | 0.579 | 1.09 |
| 29 | Homo sapiens | Dual specificity tyrosine-phosphorylation-regulated kinase 1A | 6LN1 | 347 | 0.983 | 96.564 | 0.469 | 0.90 | 0.958 | 0.420 | 1.43 |
| 30 | Homo sapiens | TMEM120A | 7N7P | 356 | 0.845 | 86.551 | 0.403 | 2.34 | 0.711 | 0.353 | 2.01 |
| 31 | Trypanosoma cruzi strain CL Brener | F337L mutation of Trypanosoma cruzi glucokinase in the apo form | 7S2N | 381 | 0.927 | 95.859 | 0.469 | 1.56 | 0.861 | 0.374 | 2.30 |
| 32 | Pseudomonas aeruginosa | siderophore reductase FoxB | 7ABW | 382 | 0.959 | 93.637 | 0.409 | 1.51 | 0.880 | 0.349 | 2.29 |
| 33 | Zoogloea ramigera | biosynthetic thiolase Y218E/delH221 mutant | 7LCA | 398 | 0.997 | 97.360 | 0.244 | 0.43 | 0.975 | 0.206 | 1.15 |
| 34 | Zoogloea ramigera | biosynthetic thiolase Q183Y mutant | 7LCL | 399 | 0.998 | 97.462 | 0.243 | 0.38 | 0.975 | 0.208 | 1.20 |
| 35 | Homo sapiens | indoleamine 2,3-dioxygenagse 1 (IDO1) complexed with IACS-8968 | 7M7D | 411 | 0.994 | 91.738 | 0.459 | 0.63 | 0.973 | 0.402 | 1.18 |
| 36 | Yersinia pestis | second heterocyclization domain of yersiniabactin synthetase | 7JTJ | 452 | 0.981 | 91.820 | 0.882 | 0.96 | 0.946 | 0.781 | 1.77 |
| 37 | Vibrio cholerae | L,D-transpeptidase LdtA | 7AJ9 | 523 | 0.975 | 90.661 | 0.440 | 1.28 | 0.924 | 0.384 | 1.97 |
| 38 | Bos taurus | RecQL | 7A8R | 531 | 0.886 | 94.174 | 0.428 | 2.34 | 0.892 | 0.382 | 2.61 |
| 39 | Bos taurus | RPE65 | 3KVC | 533 | 0.990 | 91.483 | 0.458 | 0.81 | 0.982 | 0.404 | 1.07 |
| 40 | Mus musculus | monomeric TTYH2 | 7RTV | 540 | 0.944 | 80.352 | 0.742 | 1.69 | 0.878 | 0.660 | 2.50 |
| 41 | Escherichia coli | MsbA in complex with G247 | 7MEW | 605 | 0.774 | 86.526 | 0.372 | 2.60 | 0.686 | 0.350 | 2.93 |
| 42 | Bacteroides clarus | PL6 alginate lyase BcAlyPL6 | 7DMK | 747 | 0.990 | 95.551 | 0.231 | 0.83 | 0.937 | 0.186 | 1.90 |
| 43 | Saccharomyces cerevisiae | Dhr1 Helicase Core | 7MQJ | 798 | 0.698 | 82.058 | 0.767 | 2.06 | 0.655 | 0.668 | 2.29 |
| 44 | Homo sapiens | p97-D592N mutant bound to ADP | 7RLG | 821 | 0.768 | 81.323 | 0.124 | 3.39 | 0.709 | 0.115 | 2.37 |
| 45 | Mus musculus | Isoform 2 of Neutral alpha-glucosidase AB | 7KB8 | 977 | 0.999 | 91.662 | 0.467 | 0.55 | 0.986 | 0.378 | 1.19 |
| 46 | Homo sapiens | WT transporter state1 | 7E7S | 1042 | 0.691 | 86.135 | 0.728 | 3.15 | 0.825 | 0.717 | 2.87 |
| 47 | Danio rerio | TRPM5 in the presence of 1 mM EDTA | 7MBP | 1165 | 0.832 | 78.668 | 0.209 | 2.01 | 0.900 | 0.189 | 2.61 |
| 48 | Homo sapiens | NPC1L1 mutant-W347R | 7N4X | 1332 | 0.977 | 84.414 | 0.856 | 1.37 | 0.965 | 0.774 | 1.78 |
