# Supplementary material for: Artificial Intelligence-based database for prediction of protein structure and their alterations in ocular diseases
Source: Database (Oxford). 2023 Dec 18;2023:baad083. doi: 10.1093/database/baad083 (PMC10727695; doi:10.1093/database/baad083)
Supplement: baad083_Supp [file baad083_supp.zip › suppl_data/Suppl Table 4-predict.docx]

| **Gene** | **OMIM** | **Phenosubtype** | **WT/ Mutant** | **seq_len** | **dir_name** | **alphafold_plddt** | **alphafold_plddt_0** | **rosetta_b_factor** | **rosetta_b_factor_0** |
| --- | --- | --- | --- | --- | --- | --- | --- | --- | --- |
| ABCA4 | 601691 | Stargardt disease, Cone-rod retinal dystrophy, Macular degeneration, age-related, Retinitis pigmentosa | WT | 2273 | ABCA4NM_000350WT | 75.1458_74.7284_73.8914_72.2906_71.2491 | 75.1458 | 7.9532_7.9532_7.9532_7.9532_7.9532 | 7.9532 |
| ABCA4 | 601691 | Stargardt disease, Cone-rod retinal dystrophy, Macular degeneration, age-related, Retinitis pigmentosa | Mutant | 2273 | ABCA4NM_000350C2133R | 75.2945_75.2695_73.0805_71.5932_70.7931 | 75.2945 | 7.9532_7.9532_7.9532_7.9532_7.9532 | 7.9532 |
| ABCA4 | 601691 | Stargardt disease, Cone-rod retinal dystrophy, Macular degeneration, age-related, Retinitis pigmentosa | Mutant | 2273 | ABCA4NM_000350D2102G | 75.4889_75.4022_73.7202_72.2046_70.7768 | 75.4889 | 7.9532_7.9532_7.9532_7.9532_7.9532 | 7.9532 |
| ABCA4 | 601691 | Stargardt disease, Cone-rod retinal dystrophy, Macular degeneration, age-related, Retinitis pigmentosa | Mutant | 2273 | ABCA4NM_000350N965S | 75.574_75.3345_73.3565_72.1078_70.0542 | 75.574 | 7.9532_7.9532_7.9532_7.9532_7.9532 | 7.9532 |
| ABCA4 | 601691 | Stargardt disease, Cone-rod retinal dystrophy, Macular degeneration, age-related, Retinitis pigmentosa | Mutant | 2039 | ABCA4NM_000350R2040X | 76.6304_75.93_73.6626_73.3129_71.8145 | 76.6304 | 8.0832_8.0835_8.0834_8.0836_8.0836 | 8.0832 |
| ABCA4 | 601691 | Stargardt disease, Cone-rod retinal dystrophy, Macular degeneration, age-related, Retinitis pigmentosa | Mutant | 2273 | ABCA4NM_000350R602W | 75.277_75.2761_73.7212_71.9519_70.2656 | 75.277 | 8.0366_8.0366_8.0366_8.0367_8.0366 | 8.0366 |
| ABCA4 | 601691 | Stargardt disease, Cone-rod retinal dystrophy, Macular degeneration, age-related, Retinitis pigmentosa | Mutant | 1524 | ABCA4NM_000350T1519fs | 76.8778_76.431_73.1709_72.6749_71.5212 | 76.8778 | 7.9402_7.9403_7.9403_7.9403_7.9403 | 7.9402 |
| ABCA4 | 601691 | Stargardt disease, Cone-rod retinal dystrophy, Macular degeneration, age-related, Retinitis pigmentosa | Mutant | 2273 | ABCA4NM_000350T1537M | 75.6792_74.7742_74.3784_72.3478_71.2126 | 75.6792 | 7.9531_7.9532_7.9532_7.9532_7.9532 | 7.9531 |
| ABCC6 | 603234 | Pseudoxanthoma elasticum | WT | 1503 | ABCC6WT | 81.2497_80.9646_80.5357_79.5837_79.4023 | 81.2497 | 8.4003_8.4004_8.4007_8.4011_8.4017 | 8.4003 |
| ABCC6 | 603234 | Pseudoxanthoma elasticum | Mutant | 1503 | ABCC6R1138Q | 80.9164_80.7438_79.9973_79.5319_79.329 | 80.9164 | 8.1583_8.1712_8.169_8.1706_8.187 | 8.1583 |
| ABCC6 | 603234 | Pseudoxanthoma elasticum | Mutant | 1503 | ABCC6R1339C | 81.1484_80.9055_80.4218_79.3017_78.9078 | 81.1484 | 8.5013_8.5014_8.5015_8.5014_8.5019 | 8.5013 |
| ABCC6 | 603234 | Pseudoxanthoma elasticum | Mutant | 1503 | ABCC6R518Q | 81.1878_80.5614_80.1339_79.3602_78.7381 | 81.1878 | 8.4001_8.4_8.4007_8.4004_8.4004 | 8.4001 |
| ABHD12 | 613599 | Retinitis pigmentosa, Syndromic cataract | WT | 404 | ABHD12WT | 83.1225_82.3215_82.2908_82.0968_81.9514 | 83.1225 | 8.0418_8.0419_8.042_8.0419_8.042 | 8.0418 |
| ABHD12 | 613599 | Retinitis pigmentosa, Syndromic cataract | Mutant | 349 | ABHD12298-352MISSING | 74.6381_73.5298_72.1247_71.0487_70.5904 | 74.6381 | 8.0015_8.0015_8.0015_8.0015_8.0015 | 8.0015 |
| ABHD12 | 613599 | Retinitis pigmentosa, Syndromic cataract | Mutant | 404 | ABHD12T202I | 83.0673_82.4624_82.003_81.9985_81.6309 | 83.0673 | 8.0419_8.042_8.042_8.0419_8.0419 | 8.0419 |
| ACBD5 | 616618 | Cone-rod retinal dystrophy | WT | 490 | ACBD5WT | 58.3738_56.5383_55.2213_55.1852_53.6084 | 58.3738 | 8.0312_8.0312_8.0311_8.0313_8.0312 | 8.0312 |
| ACBD5 | 616618 | Cone-rod retinal dystrophy | Mutant | 490 | ACBD5H8Y | 58.0321_56.6981_54.3699_54.1787_53.5844 | 58.0321 | 8.0313_8.0312_8.0313_8.0312_8.0312 | 8.0313 |
| ACO2 | 100850 | Optic atrophy, Retinal syndrome | WT | 753 | ACO2WT | 97.4717_97.4461_95.5494_95.4976_95.1101 | 97.4717 | 4.7859_8.3449_8.3452_8.3452_8.3453 | 4.7859 |
| ACO2 | 100850 | Optic atrophy, Retinal syndrome | Mutant | 753 | ACO2G259D | 97.4411_97.3921_95.7273_95.2587_95.1043 | 97.4411 | 4.753_7.9415_7.9417_7.9417_7.9415 | 4.753 |
| ACO2 | 100850 | Optic atrophy, Retinal syndrome | Mutant | 753 | ACO2G661R | 97.3722_97.2313_95.6523_95.4664_94.988 | 97.3722 | 4.7938_8.3448_8.3448_8.3448_8.3451 | 4.7938 |
| ACTA1 | 102610 | Myopathy, congenital, with fiber-type disproportion | WT | 375 | ACTA1WT | 95.0088_94.8777_94.0291_93.5753_93.4337 | 95.0088 | 7.8269_7.8273_7.8275_7.8276_7.8278 | 7.8269 |
| ACTA1 | 102610 | Myopathy, congenital, with fiber-type disproportion | Mutant | 375 | ACTA1R185G | 95.0423_95.0075_94.6593_93.83_93.817 | 95.0423 | 7.8261_7.8268_7.828_7.8271_7.8282 | 7.8261 |
| ACTA1 | 102610 | Myopathy, congenital, with fiber-type disproportion | Mutant | 375 | ACTA1V165M | 95.2456_95.2122_95.0673_94.8119_94.5482 | 95.2456 | 7.8157_7.818_7.8179_7.8184_7.8187 | 7.8157 |
| ADAM9 | 602713 | Cone-rod dystrophy, Cone-rod retinal dystrophy | WT | 791 | ADAM9WT | 77.6199_76.9404_76.5829_75.5709_75.0197 | 77.6199 | 8.0372_8.0372_8.0372_8.0372_8.0373 | 8.0372 |
| ADAM9 | 602713 | Cone-rod dystrophy, Cone-rod retinal dystrophy | Mutant | 135 | ADAM9R164X | 89.3554_89.2891_88.5674_88.1592_87.8839 | 89.3554 | 8.4205_8.4227_8.4454_8.4556_8.5061 | 8.4205 |
| ADAM9 | 602713 | Cone-rod dystrophy, Cone-rod retinal dystrophy | Mutant | 227 | ADAM9R256X | 84.1907_83.783_83.3948_82.7641_82.2279 | 84.1907 | 7.9468_7.9481_7.9479_7.9493_7.9506 | 7.9468 |
| ADAMTS10 | 608990 | Weill-Marchesani syndrome | WT | 870 | ADAMTS10WT | 83.6538_83.5636_83.1839_82.7802_81.5436 | 83.6538 | 8.1765_8.1764_8.1764_8.1766_8.1765 | 8.1765 |
| ADAMTS10 | 608990 | Weill-Marchesani syndrome | Mutant | 870 | ADAMTS10G518D | 84.0775_83.8485_83.4532_83.0979_82.3165 | 84.0775 | 8.1766_8.1765_8.1764_8.1765_8.1764 | 8.1766 |
| ADAMTS10 | 608990 | Weill-Marchesani syndrome | Mutant | 870 | ADAMTS10G700C | 84.2135_83.5767_83.5416_81.405_80.7543 | 84.2135 | 8.1765_8.1765_8.1766_8.1765_8.1767 | 8.1765 |
| ADAMTS18 | 607512 | Chorioretinopathy, Knobloch syndrome, type 1, Microcornea, myopic chorioretinal atrophy, and telecanthus, Knobloch syndrome 2, Choroid retinal atrophy | WT | 937 | ADAMTS18NM_199355WT | 81.9086_80.4117_79.2603_78.9508_78.1084 | 81.9086 | 5.5237_8.0449_8.045_8.045_8.0451 | 5.5237 |
| ADAMTS18 | 607512 | Chorioretinopathy, Knobloch syndrome, type 1, Microcornea, myopic chorioretinal atrophy, and telecanthus, Knobloch syndrome 2, Choroid retinal atrophy | Mutant | 937 | ADAMTS18NM_199355C577W | 82.1842_80.2905_79.1251_78.5731_78.4997 | 82.1842 | 5.5503_8.0448_8.045_8.045_8.0451 | 5.5503 |
| ADAMTSL4 | 610113 | Ectopia lentis et pupillae, Ectopia lentis, Albinism, oculocutaneous, type II | WT | 1050 | ADAMTSL4WT | 66.0737_64.9532_64.4789_64.0154_62.9143 | 66.0737 | 2.874_8.0425_8.0764_8.0772_8.0873 | 2.874 |
| ADAMTSL4 | 610113 | Ectopia lentis et pupillae, Ectopia lentis, Albinism, oculocutaneous, type II | Mutant | 570 | ADAMTSL4Y595X | 64.4786_62.1105_61.0186_60.8425_60.8033 | 64.4786 | 5.2455_8.0323_8.0324_8.0324_8.0323 | 5.2455 |
| AGBL1 | 615496 | Dystrophia endothelialis corneae, Corneal dystrophy, Fuchs endothelial, 8 | WT | 1112 | AGBL1WT | 78.4821_78.2455_78.2072_78.1344_77.9106 | 78.4821 | 7.93_7.93_7.93_7.93_7.93 | 7.93 |
| AGBL1 | 615496 | Dystrophia endothelialis corneae, Corneal dystrophy, Fuchs endothelial, 8 | Mutant | 1112 | AGBL1C1036S | 79.0234_78.5295_78.439_78.3619_78.1478 | 79.0234 | 7.93_7.93_7.93_7.93_7.93 | 7.93 |
| AGBL5 | 615900 | Retinitis pigmentosa 75 | WT | 886 | AGBL5NM_021831WT | 70.0268_68.9869_67.7182_67.5635_66.8038 | 70.0268 | 8.1735_8.1735_8.1736_8.1736_8.1737 | 8.1735 |
| AGBL5 | 615900 | Retinitis pigmentosa 75 | Mutant | 886 | AGBL5NM_021831R118C | 69.7853_68.7838_67.838_66.8684_66.7486 | 69.7853 | 8.1735_8.1736_8.1736_8.1737_8.1736 | 8.1735 |
| AGK | 610345 | Congenital cataract and developmental cataract, Sengers syndrome | WT | 422 | AGKWT | 87.2532_86.774_86.2925_85.4997_84.6655 | 87.2532 | 8.0332_8.0332_8.0332_8.033_8.0332 | 8.0332 |
| AGK | 610345 | Congenital cataract and developmental cataract, Sengers syndrome | Mutant | 136 | AGK137-422MISSING | 90.5554_89.5101_88.4858_87.2409_85.5231 | 90.5554 | 8.0132_8.0132_8.0132_8.0132_8.0132 | 8.0132 |
| AGK | 610345 | Congenital cataract and developmental cataract, Sengers syndrome | Mutant | 290 | AGK291-422MISSING | 87.3817_86.6057_86.0955_85.1482_82.7496 | 87.3817 | 8.3649_8.3634_8.3679_8.3708_8.37 | 8.3649 |
| AGK | 610345 | Congenital cataract and developmental cataract, Sengers syndrome | Mutant | 326 | AGK327-422MISSING | 85.5195_83.4801_83.4553_83.0173_82.8643 | 85.5195 | 8.1661_8.1662_8.1663_8.1663_8.1663 | 8.1661 |
| AHI1 | 608894 | Joubert syndrome | WT | 1196 | AHI1WT | 61.4608_60.9344_59.7657_59.5485_59.1003 | 61.4608 | 8.2622_8.2621_8.2622_8.2621_8.2624 | 8.2622 |
| AHI1 | 608894 | Joubert syndrome | Mutant | 1196 | AHI1R723Q | 61.4495_60.0544_59.8468_59.5607_59.3899 | 61.4495 | 8.2622_8.2624_8.2623_8.2625_8.2626 | 8.2622 |
| AHI1 | 608894 | Joubert syndrome | Mutant | 1196 | AHI1V443D | 61.2035_60.1727_59.4434_59.3761_59.2567 | 61.2035 | 8.1733_8.1736_8.1734_8.1737_8.1737 | 8.1733 |
| AIPL1 | 604392 | Leber congenital amaurosis 4, Leber congenital amaurosis | WT | 384 | AIPL1NM_014336WT | 82.6718_81.4024_81.0263_80.0899_79.4505 | 82.6718 | 6.6204_8.0392_8.0392_8.0392_8.0393 | 6.6204 |
| AIPL1 | 604392 | Leber congenital amaurosis 4, Leber congenital amaurosis | Mutant | 384 | AIPL1NM_014336C239R | 81.7398_81.0362_79.5454_78.9103_77.5728 | 81.7398 | 6.6602_8.0391_8.0391_8.0392_8.0392 | 6.6602 |
| AIPL1 | 604392 | Leber congenital amaurosis 4, Leber congenital amaurosis | Mutant | 384 | AIPL1NM_014336R270H | 82.7539_81.3627_81.3291_80.2867_79.6853 | 82.7539 | 6.7152_8.0392_8.0392_8.0392_8.0392 | 6.7152 |
| ANO5 | 608662 | Macular dystrophy | WT | 913 | ANO5WT | 82.2019_81.1203_80.6561_78.7517_78.7479 | 82.2019 | 6.7218_8.336_8.3393_8.3398_8.3392 | 6.7218 |
| ANO5 | 608662 | Macular dystrophy | Mutant | 913 | ANO5R58W | 82.1513_81.2189_80.482_78.9633_78.6792 | 82.1513 | 6.7685_8.3365_8.3361_8.3373_8.3391 | 6.7685 |
| ANO5 | 608662 | Macular dystrophy | Mutant | 913 | ANO5R758C | 81.9776_80.6272_80.2789_79.1306_78.8199 | 81.9776 | 6.7191_8.3379_8.3372_8.339_8.3396 | 6.7191 |
| ANTXR1 | 606410 | Optic atrophy | WT | 301 | ANTXR1WT | 92.2149_91.743_91.4105_87.2268_85.4669 | 92.2149 | 8.1865_8.1865_8.1865_8.1865_8.1865 | 8.1865 |
| ANTXR1 | 606410 | Optic atrophy | Mutant | 136 | ANTXR1R169X | 88.6926_88.6781_87.252_85.9043_85.3958 | 88.6926 | 8.0136_8.0136_8.0136_8.0136_8.0136 | 8.0136 |
| AP3B1 | 603401 | Hermansky-Pudlak syndrome | WT | 1094 | AP3B1WT | 75.0073_72.5259_71.3959_71.2005_70.7446 | 75.0073 | 8.0201_8.0201_8.0202_8.0202_8.0202 | 8.0201 |
| AP3B1 | 603401 | Hermansky-Pudlak syndrome | Mutant | 1073 | AP3B1390-410MISSING | 73.0917_70.4523_69.6628_68.6019_68.441 | 73.0917 | 7.835_8.378_8.3789_8.3789_8.3819 | 7.835 |
| AP3B1 | 603401 | Hermansky-Pudlak syndrome | Mutant | 1094 | AP3B1L580R | 74.4777_72.5244_70.8087_70.6838_69.5411 | 74.4777 | 8.0202_8.0201_8.0202_8.0202_8.0201 | 8.0202 |
| APOE | 107741 | Macular degeneration, age-related | WT | 299 | APOEWT | 75.2301_72.1235_68.3303_64.7507_62.3394 | 75.2301 | 8.1892_8.1892_8.1893_8.1892_8.1892 | 8.1892 |
| APOE | 107741 | Macular degeneration, age-related | Mutant | 299 | APOEC130R | 75.5107_71.7471_65.7449_65.6575_62.2864 | 75.5107 | 8.1892_8.1892_8.1893_8.1891_8.1892 | 8.1892 |
| APOE | 107741 | Macular degeneration, age-related | Mutant | 299 | APOER176C | 75.359_70.4694_65.0503_63.7917_61.7601 | 75.359 | 8.1891_8.1892_8.1892_8.1893_8.1892 | 8.1891 |
| ARHGEF18 | 616432 | Retinitis pigmentosa 78 | WT | 1361 | ARHGEF18WT | 59.4026_59.2524_58.3308_58.2447_58.2292 | 59.4026 | 7.9022_7.93_7.93_7.93_7.93 | 7.9022 |
| ARHGEF18 | 616432 | Retinitis pigmentosa 78 | Mutant | 1353 | ARHGEF181101-1108MISSING | 60.7353_59.5032_59.4395_59.2818_59.0339 | 60.7353 | 8.0449_8.045_8.0452_8.0451_8.0451 | 8.0449 |
| ARHGEF18 | 616432 | Retinitis pigmentosa 78 | Mutant | 1361 | ARHGEF18T458A | 61.1184_59.4751_59.4644_59.2015_59.1561 | 61.1184 | 8.0475_8.0477_8.0476_8.0477_8.0476 | 8.0475 |
| ARL13B | 608922 | Joubert syndrome | WT | 428 | ARL13BWT | 73.4071_71.4365_71.4329_70.817_70.6761 | 73.4071 | 8.039_8.039_8.0392_8.0391_8.0392 | 8.039 |
| ARL13B | 608922 | Joubert syndrome | Mutant | 428 | ARL13BR200C | 73.3422_71.3036_71.1776_71.1442_70.5692 | 73.3422 | 8.039_8.0391_8.0391_8.0391_8.039 | 8.039 |
| ARL13B | 608922 | Joubert syndrome | Mutant | 428 | ARL13BY86C | 73.5342_71.7276_71.4966_71.1662_71.0281 | 73.5342 | 8.81_8.81_8.81_8.81_8.81 | 8.81 |
| ARL3 | 604695 | Retinitis pigmentosa | WT | 181 | ARL3WT | 93.6506_92.5275_92.2349_90.9348_90.7544 | 93.6506 | 8.2144_8.2069_8.2157_8.2103_8.2174 | 8.2144 |
| ARL3 | 604695 | Retinitis pigmentosa | Mutant | 181 | ARL3R149C | 92.8211_92.7469_92.2859_92.1707_92.0514 | 92.8211 | 8.221_8.2214_8.2258_8.2226_8.2256 | 8.221 |
| ARL3 | 604695 | Retinitis pigmentosa | Mutant | 181 | ARL3Y90C | 93.3096_92.9459_92.736_92.6825_92.5842 | 93.3096 | 8.217_8.2242_8.2194_8.2224_8.2302 | 8.217 |
| ARL6 | 608845 | Retinitis pigmentosa, Bardet-Biedl syndrome | WT | 185 | ARL6WT | 94.9064_94.3589_94.1201_94.0553_93.6971 | 94.9064 | 5.5535_7.8525_7.85_7.8628_7.8637 | 5.5535 |
| ARL6 | 608845 | Retinitis pigmentosa, Bardet-Biedl syndrome | Mutant | 185 | ARL6G169A | 94.6126_94.3179_94.2734_94.0756_85.8763 | 94.6126 | 5.5935_8.1728_8.1723_8.1781_8.1761 | 5.5935 |
| ARL6 | 608845 | Retinitis pigmentosa, Bardet-Biedl syndrome | Mutant | 185 | ARL6L170W | 93.7828_93.5392_93.0326_92.673_91.9756 | 93.7828 | 5.5483_8.159_8.161_8.1684_8.1709 | 5.5483 |
| ARL6 | 608845 | Retinitis pigmentosa, Bardet-Biedl syndrome | Mutant | 185 | ARL6T31M | 94.476_94.3791_94.3257_93.9227_86.9137 | 94.476 | 5.6386_8.1678_8.1693_8.1742_8.1753 | 5.6386 |
| ARMS2 | 611313 | Macular degeneration, age-related | WT | 107 | ARMS2WT | 59.21_57.5538_56.2232_53.5015_52.2394 | 59.21 | 7.911_7.9149_7.9142_7.9168_7.9202 | 7.911 |
| ARMS2 | 611313 | Macular degeneration, age-related | Mutant | 107 | ARMS2A69S | 57.7116_56.0876_48.7863_47.2536_46.4194 | 57.7116 | 7.9151_7.918_7.917_7.9178_7.9205 | 7.9151 |
| ARMS2 | 611313 | Macular degeneration, age-related | Mutant | 37 | ARMS2R38X | 72.0778_68.2849_67.9179_66.0998_62.9482 | 72.0778 | 7.0113_8.1985_8.5077_7.9295_8.7426 | 7.0113 |
| ARR3 | 301770 | Myopia 26, X-linked, female-limited | WT | 388 | ARR3WT | 86.6682_85.4018_83.691_82.9566_81.9795 | 86.6682 | 6.3385_8.0407_8.0408_8.0408_8.0409 | 6.3385 |
| ARR3 | 301770 | Myopia 26, X-linked, female-limited | Mutant | 99 | ARR3100-388MISSING | 86.1063_86.0876_85.6985_85.5144_85.3743 | 86.1063 | 7.98_7.9804_7.9819_7.9828_7.9829 | 7.98 |
| ARR3 | 301770 | Myopia 26, X-linked, female-limited | Mutant | 388 | ARR3A298D | 85.6624_84.3989_83.2095_81.9671_80.5929 | 85.6624 | 6.3391_9.433_9.435_9.4327_9.4342 | 6.3391 |
| ARR3 | 301770 | Myopia 26, X-linked, female-limited | Mutant | 388 | ARR3L80P | 85.5112_85.4929_84.4565_82.4863_81.6804 | 85.5112 | 6.2925_9.4332_9.4342_9.434_9.4338 | 6.2925 |
| ASB10 | 615054 | Open-angle glaucoma, Glaucoma | WT | 467 | ASB10NM_080871WT | 78.9365_77.6898_76.3064_76.1844_74.3223 | 78.9365 | 9.306_9.308_9.3078_9.3079_9.3096 | 9.306 |
| ASB10 | 615054 | Open-angle glaucoma, Glaucoma | Mutant | 467 | ASB10A197V | 78.7139_78.702_77.225_76.4012_75.4114 | 78.7139 | 9.269_9.2701_9.2701_9.2698_9.2701 | 9.269 |
| ASB10 | 615054 | Open-angle glaucoma, Glaucoma | Mutant | 467 | ASB10D97E | 78.2004_77.7867_77.4667_76.3081_74.9968 | 78.2004 | 8.0415_8.0415_8.0416_8.0416_8.0416 | 8.0415 |
| ASB10 | 615054 | Open-angle glaucoma, Glaucoma | Mutant | 467 | ASB10G65E | 79.6038_78.5523_77.3355_75.4569_74.0792 | 79.6038 | 9.2694_9.2698_9.2692_9.2689_9.2697 | 9.2694 |
| ASB10 | 615054 | Open-angle glaucoma, Glaucoma | Mutant | 467 | ASB10H332Q | 79.9041_77.8854_76.0458_75.6575_74.7866 | 79.9041 | 8.0414_8.0416_8.0414_8.0415_8.0415 | 8.0414 |
| ASB10 | 615054 | Open-angle glaucoma, Glaucoma | Mutant | 467 | ASB10H356Y | 79.3012_79.2273_76.6669_75.9119_73.6772 | 79.3012 | 9.2671_9.268_9.2683_9.2683_9.2684 | 9.2671 |
| ASB10 | 615054 | Open-angle glaucoma, Glaucoma | Mutant | 467 | ASB10NM_080871Q295L | 78.1708_77.4096_76.3099_76.2078_75.3533 | 78.1708 | 8.0416_8.0415_8.0414_8.0415_8.0416 | 8.0416 |
| ASB10 | 615054 | Open-angle glaucoma, Glaucoma | Mutant | 467 | ASB10NM_080871R72H | 78.7919_78.2652_77.0428_77.0013_75.0962 | 78.7919 | 8.0415_8.0415_8.0415_8.0416_8.0415 | 8.0415 |
| ASB10 | 615054 | Open-angle glaucoma, Glaucoma | Mutant | 467 | ASB10NM_080871S440G | 79.9308_79.0905_77.3379_77.3326_75.434 | 79.9308 | 8.0415_8.0415_8.0415_8.0416_8.0416 | 8.0415 |
| ASB10 | 615054 | Open-angle glaucoma, Glaucoma | Mutant | 467 | ASB10R183C | 78.9608_78.8016_77.5629_77.4288_73.7137 | 78.9608 | 8.0417_8.0416_8.0415_8.0416_8.0417 | 8.0417 |
| ASB10 | 615054 | Open-angle glaucoma, Glaucoma | Mutant | 467 | ASB10R272H | 78.2546_76.8222_75.7203_75.3452_72.9178 | 78.2546 | 9.2358_9.2362_9.2366_9.2365_9.2362 | 9.2358 |
| ASB10 | 615054 | Open-angle glaucoma, Glaucoma | Mutant | 467 | ASB10R94Q | 81.2793_77.6254_76.5254_75.4001_74.9278 | 81.2793 | 9.2344_9.235_9.2355_9.2357_9.236 | 9.2344 |
| ASB10 | 615054 | Open-angle glaucoma, Glaucoma | Mutant | 467 | ASB10T48S | 78.5664_78.4111_75.9244_74.8035_73.506 | 78.5664 | 8.0416_8.0417_8.0416_8.0416_8.0417 | 8.0416 |
| ATF6 | 605537 | Achromatopsia | WT | 670 | ATF6WT | 55.8402_51.7989_51.1417_51.0302_50.9216 | 55.8402 | 8.035_8.035_8.035_8.035_8.035 | 8.035 |
| ATF6 | 605537 | Achromatopsia | Mutant | 670 | ATF6R324C | 55.3728_51.7318_51.3983_50.9889_50.9263 | 55.3728 | 7.9829_7.9837_7.9844_7.9868_7.9888 | 7.9829 |
| ATF6 | 605537 | Achromatopsia | Mutant | 670 | ATF6Y567N | 55.5554_51.407_51.0386_50.9196_50.8512 | 55.5554 | 8.0349_8.035_8.0351_8.035_8.035 | 8.0349 |
| ATOH7 | 609875 | Permanent primary vitreous hyperplasia, Aniridia, Persistent hyperplastic primary vitreous, autosomal recessive, Vitreoretinochoroidopathy | WT | 152 | ATOH7WT | 71.6929_71.6751_70.7996_69.5876_68.6145 | 71.6929 | 7.9492_7.9514_7.9594_7.9643_7.9736 | 7.9492 |
| ATOH7 | 609875 | Permanent primary vitreous hyperplasia, Aniridia, Persistent hyperplastic primary vitreous, autosomal recessive, Vitreoretinochoroidopathy | Mutant | 152 | ATOH7E49V | 71.5934_71.5656_70.9016_69.9947_69.0769 | 71.5934 | 7.956_7.9484_7.946_7.945_7.9606 | 7.956 |
| ATOH7 | 609875 | Permanent primary vitreous hyperplasia, Aniridia, Persistent hyperplastic primary vitreous, autosomal recessive, Vitreoretinochoroidopathy | Mutant | 152 | ATOH7N46H | 72.0638_71.5463_71.084_69.3067_68.69 | 72.0638 | 8.0223_8.035_8.0282_8.0276_8.0248 | 8.0223 |
| ATP13A2 | 610513 | Kufor-Rakeb syndrome, Spastic paraplegia 78, autosomal recessive | WT | 1180 | ATP13A2WT | 79.7624_79.1047_78.9679_78.0233_76.9358 | 79.7624 | 8.1736_8.1737_8.1741_8.1743_8.1742 | 8.1736 |
| ATP13A2 | 610513 | Kufor-Rakeb syndrome, Spastic paraplegia 78, autosomal recessive | Mutant | 1180 | ATP13A2F182L | 79.6046_78.9909_78.796_78.0075_76.6731 | 79.6046 | 8.124_8.1244_8.1244_8.1242_8.1244 | 8.124 |
| ATP13A2 | 610513 | Kufor-Rakeb syndrome, Spastic paraplegia 78, autosomal recessive | Mutant | 1180 | ATP13A2G533R | 79.6123_79.3429_78.787_78.1345_77.1993 | 79.6123 | 8.1737_8.1738_8.1737_8.1739_8.1741 | 8.1737 |
| ATP1A3 | 182350 | Optic atrophy, Alternating hemiplegia of childhood 2 | WT | 1013 | ATP1A3WT | 90.6324_90.1118_86.0546_85.1133_84.5717 | 90.6324 | 8.2428_8.2428_8.2428_8.2429_8.2429 | 8.2428 |
| ATP1A3 | 182350 | Optic atrophy, Alternating hemiplegia of childhood 2 | Mutant | 1013 | ATP1A3E818K | 90.6967_90.2012_86.5475_85.4799_85.4448 | 90.6967 | 8.2429_8.2428_8.2428_8.2428_8.2428 | 8.2429 |
| BBS1 | 209901 | Bardet-Biedl syndrome | WT | 592 | BBS1WT | 88.8643_88.5332_88.3188_87.4711_87.218 | 88.8643 | 8.0391_8.0393_8.0393_8.0393_8.0393 | 8.0391 |
| BBS1 | 209901 | Bardet-Biedl syndrome | Mutant | 592 | BBS1L518P | 88.9276_88.5759_88.1138_88.1061_87.2864 | 88.9276 | 8.0393_8.0392_8.0394_8.0393_8.0392 | 8.0393 |
| BBS1 | 209901 | Bardet-Biedl syndrome | Mutant | 592 | BBS1M390R | 88.5592_87.9882_87.8916_87.2584_87.127 | 88.5592 | 8.0392_8.0391_8.0392_8.0392_8.0394 | 8.0392 |
| BBS10 | 610148 | Bardet-Biedl syndrome | WT | 723 | BBS10WT | 72.2679_71.5883_71.2804_70.1017_69.8905 | 72.2679 | 7.9472_7.9475_7.9475_7.9476_7.9477 | 7.9472 |
| BBS10 | 610148 | Bardet-Biedl syndrome | Mutant | 723 | BBS10R49W | 71.9271_71.5024_71.0963_70.6781_69.1202 | 71.9271 | 7.9472_7.9476_7.9476_7.9475_7.9476 | 7.9472 |
| BBS10 | 610148 | Bardet-Biedl syndrome | Mutant | 723 | BBS10S311A | 72.4427_71.4466_71.054_70.2021_68.4392 | 72.4427 | 7.9474_7.9475_7.9475_7.9474_7.9477 | 7.9474 |
| BBS12 | 610683 | Bardet-Biedl syndrome | WT | 710 | BBS12WT | 74.8399_74.4436_73.9549_73.6735_73.4018 | 74.8399 | 8.239_8.2393_8.2394_8.2394_8.2393 | 8.239 |
| BBS12 | 610683 | Bardet-Biedl syndrome | Mutant | 710 | BBS12A289P | 74.3575_74.2613_74.01_73.6978_73.5364 | 74.3575 | 8.2391_8.2393_8.2394_8.2395_8.2393 | 8.2391 |
| BBS12 | 610683 | Bardet-Biedl syndrome | Mutant | 710 | BBS12T501M | 74.4425_74.114_73.9336_73.5886_73.2952 | 74.4425 | 8.2393_8.2393_8.2395_8.2396_8.2394 | 8.2393 |
| BBS2 | 606151 | Bardet-Biedl syndrome, Retinitis pigmentosa | WT | 721 | BBS2WT | 88.6749_88.4247_88.377_88.1511_86.6674 | 88.6749 | 7.5029_7.9435_7.9437_7.9438_7.9437 | 7.5029 |
| BBS2 | 606151 | Bardet-Biedl syndrome, Retinitis pigmentosa | Mutant | 721 | BBS2D104A | 88.4886_87.9869_87.9392_87.7886_86.5111 | 88.4886 | 7.4958_7.9436_7.9435_7.9437_7.9437 | 7.4958 |
| BBS2 | 606151 | Bardet-Biedl syndrome, Retinitis pigmentosa | Mutant | 721 | BBS2R632P | 88.7976_88.5339_87.9863_87.1894_86.2094 | 88.7976 | 7.956_7.9559_7.9563_7.9564_7.9568 | 7.956 |
| BBS4 | 600374 | Bardet-Biedl syndrome | WT | 519 | BBS4WT | 76.8266_76.4659_76.1224_75.3473_74.7381 | 76.8266 | 7.848_7.8486_7.8491_7.8521_7.8512 | 7.848 |
| BBS4 | 600374 | Bardet-Biedl syndrome | Mutant | 519 | BBS4M472V | 77.9353_76.9239_76.0668_75.3837_74.7053 | 77.9353 | 7.8473_7.8469_7.8494_7.8485_7.8515 | 7.8473 |
| BBS4 | 600374 | Bardet-Biedl syndrome | Mutant | 519 | BBS4N165H | 77.4696_76.8593_75.4541_75.3523_74.5819 | 77.4696 | 7.8465_7.8465_7.8499_7.8506_7.8514 | 7.8465 |
| BBS5 | 603650 | Bardet-Biedl syndrome | WT | 341 | BBS5WT | 88.3794_88.3388_88.1041_87.6233_87.4173 | 88.3794 | 8.1936_8.1936_8.1936_8.1936_8.1936 | 8.1936 |
| BBS5 | 603650 | Bardet-Biedl syndrome | Mutant | 341 | BBS5G72S | 88.87_88.4301_88.3063_87.8472_87.3321 | 88.87 | 8.1935_8.1936_8.1936_8.1936_8.1934 | 8.1935 |
| BBS5 | 603650 | Bardet-Biedl syndrome | Mutant | 341 | BBS5T183A | 88.4766_88.3883_87.6401_87.3734_85.8684 | 88.4766 | 8.1936_8.1936_8.1936_8.1936_8.1937 | 8.1936 |
| BBS7 | 607590 | Bardet-Biedl syndrome | WT | 715 | BBS7WT | 92.7882_92.5773_91.5928_91.3116_90.9197 | 92.7882 | 6.8676_8.2393_8.2394_8.2393_8.2394 | 6.8676 |
| BBS7 | 607590 | Bardet-Biedl syndrome | Mutant | 715 | BBS7I66F | 92.6556_92.415_91.2845_91.0883_90.6985 | 92.6556 | 6.8836_8.2356_8.2393_8.2393_8.2394 | 6.8836 |
| BBS7 | 607590 | Bardet-Biedl syndrome | Mutant | 715 | BBS7T211I | 92.7555_92.5604_91.6873_91.6832_91.2663 | 92.7555 | 6.9999_7.6815_7.9361_7.9376_7.9375 | 6.9999 |
| BBS9 | 615986 | Bardet-Biedl syndrome | WT | 887 | BBS9WT | 83.5673_83.3968_82.5323_81.2205_80.7933 | 83.5673 | 8.0457_8.0452_8.0465_8.0473_8.0485 | 8.0457 |
| BBS9 | 615986 | Bardet-Biedl syndrome | Mutant | 887 | BBS9G141R | 83.5008_82.577_82.234_81.4399_80.9966 | 83.5008 | 8.0459_8.0476_8.0478_8.0488_8.0495 | 8.0459 |
| BCOR | 300485 | Microphthalmia | WT | 1721 | BCORWT | 37.524_37.0286_36.905_36.729_35.6949 | 37.524 | 7.8467_7.8481_7.8487_7.8544_7.8573 | 7.8467 |
| BCOR | 300485 | Microphthalmia | Mutant | 1721 | BCORP85L | 37.5247_36.934_36.6208_36.253_36.1217 | 37.5247 | 7.9648_7.9649_7.9651_7.9653_7.9653 | 7.9648 |
| BEST1 | 607854 | Vitreoretinochoroidopathy, Microcornea, rod-cone dystrophy, cataract, and posterior staphyloma 2, Macular dystrophy, vitelliform, Macular dystrophy, Anterior segment dysgenesis 1, multiple subtypes, Retinitis pigmentosa | WT | 585 | BEST1NM_004183WT | 71.9922_71.8152_70.5525_70.082_69.6539 | 71.9922 | 5.578_8.0342_8.0343_8.0343_8.0343 | 5.578 |
| BEST1 | 607854 | Vitreoretinochoroidopathy, Microcornea, rod-cone dystrophy, cataract, and posterior staphyloma 2, Macular dystrophy, vitelliform, Macular dystrophy, Anterior segment dysgenesis 1, multiple subtypes, Retinitis pigmentosa | Mutant | 585 | BEST1NM_004183G121A | 71.8165_71.5845_70.9806_70.5209_69.8338 | 71.8165 | 5.6474_8.0342_8.0342_8.0342_8.0343 | 5.6474 |
| BEST1 | 607854 | Vitreoretinochoroidopathy, Microcornea, rod-cone dystrophy, cataract, and posterior staphyloma 2, Macular dystrophy, vitelliform, Macular dystrophy, Anterior segment dysgenesis 1, multiple subtypes, Retinitis pigmentosa | Mutant | 585 | BEST1NM_004183P346H | 71.7609_71.7142_71.0474_70.45_70.0346 | 71.7609 | 5.6045_8.0341_8.0343_8.0341_8.0342 | 5.6045 |
| BFSP1 | 603307 | Congenital cataract and developmental cataract | WT | 665 | BFSP1WT | 65.2228_64.176_63.5135_63.2085_62.2238 | 65.2228 | 8.0354_8.0354_8.0354_8.0354_8.0356 | 8.0354 |
| BFSP1 | 603307 | Congenital cataract and developmental cataract | Mutant | 665 | BFSP1D348N | 66.0518_63.6935_63.4187_62.5747_61.8664 | 66.0518 | 8.0351_8.0354_8.0353_8.0355_8.0354 | 8.0351 |
| BFSP2 | 603212 | Cataract, Congenital cataract and developmental cataract | WT | 414 | BFSP2WT | 76.8077_76.1233_75.947_75.2101_74.9712 | 76.8077 | 8.0325_8.0325_8.0324_8.0325_8.0326 | 8.0325 |
| BFSP2 | 603212 | Cataract, Congenital cataract and developmental cataract | Mutant | 413 | BFSP2233MISSING | 76.5919_75.3384_75.0085_74.7193_74.4645 | 76.5919 | 8.035_8.035_8.035_8.035_8.035 | 8.035 |
| BFSP2 | 603212 | Cataract, Congenital cataract and developmental cataract | Mutant | 414 | BFSP2R287W | 77.0142_75.7167_75.3931_75.0212_74.884 | 77.0142 | 8.0325_8.0324_8.0326_8.0326_8.0326 | 8.0325 |
| BMP4 | 112262 | Marfan lipodystrophy syndrome, Microphthalmia | WT | 389 | BMP4NM_001202WT | 81.6207_80.4454_79.3823_78.311_77.4286 | 81.6207 | 7.6668_8.0294_8.0293_8.0294_8.0292 | 7.6668 |
| BMP4 | 112262 | Marfan lipodystrophy syndrome, Microphthalmia | Mutant | 389 | BMP4NM_001202R287H | 80.2177_79.6666_79.1901_78.4894_78.0372 | 80.2177 | 7.6881_8.0293_8.0293_8.0293_8.0294 | 7.6881 |
| BMP4 | 112262 | Marfan lipodystrophy syndrome, Microphthalmia | Mutant | 389 | BMP4NM_001202S91C | 80.5403_79.9253_79.8307_78.4258_77.5993 | 80.5403 | 7.6761_8.0292_8.0292_8.0293_8.0294 | 7.6761 |
| C12orf57 | 615140 | Temtamy syndrome | WT | 125 | C12orf57WT | 79.3968_79.1319_78.2404_78.1309_76.59 | 79.3968 | 6.366_7.9924_7.9924_7.9924_7.9924 | 6.366 |
| C12orf57 | 615140 | Temtamy syndrome | Mutant | 125 | C12orf57L51Q | 81.0158_79.0273_78.5548_77.4355_76.6934 | 81.0158 | 6.3367_7.9923_7.9927_7.9927_7.9927 | 6.3367 |
| C2orf71 | 613425 | Retinitis pigmentosa | WT | 1287 | C2orf71NM_001029883WT | 44.2132_43.8582_42.7558_42.1719_42.0299 | 44.2132 | 8.0604_8.0685_8.0684_8.0686_8.0688 | 8.0604 |
| C2orf71 | 613425 | Retinitis pigmentosa | Mutant | 1287 | C2orf71NM_001029883A71P | 44.3347_43.9304_42.231_41.6084_41.1537 | 44.3347 | 7.911_7.9119_7.924_7.9248_7.927 | 7.911 |
| C2orf71 | 613425 | Retinitis pigmentosa | Mutant | 1104 | C2orf71NM_001029883P1060fs | 47.2043_46.7002_44.1421_43.9984_43.7514 | 47.2043 | 9.743_9.7471_9.7314_9.7334_9.7375 | 9.743 |
| C3 | 120700 | Macular degeneration, age-related | WT | 1641 | C3WT | 81.8642_80.7041_80.4637_80.4147_79.3091 | 81.8642 | 7.9409_7.941_7.941_7.941_7.941 | 7.9409 |
| C3 | 120700 | Macular degeneration, age-related | Mutant | 1641 | C3K155Q | 81.7993_80.775_80.381_80.3138_78.3017 | 81.7993 | 7.941_7.941_7.941_7.941_7.941 | 7.941 |
| C5AR2 | 609949 | Retinitis pigmentosa | WT | 337 | C5AR2WT | 82.4373_81.769_81.5767_80.3979_79.7657 | 82.4373 | 8.1973_8.1973_8.1975_8.1974_8.1973 | 8.1973 |
| C5AR2 | 609949 | Retinitis pigmentosa | Mutant | 337 | C5AR2T196N | 83.0002_81.9641_81.9426_81.9422_81.5565 | 83.0002 | 8.1975_8.1975_8.1975_8.1978_8.1979 | 8.1975 |
| C8orf37 | 614477 | Cone-rod dystrophy, Cone-rod retinal dystrophy | WT | 207 | C8orf37WT | 74.9422_72.8846_72.3093_70.5961_69.9544 | 74.9422 | 7.9024_7.9052_7.9042_7.906_7.906 | 7.9024 |
| C8orf37 | 614477 | Cone-rod dystrophy, Cone-rod retinal dystrophy | Mutant | 207 | C8orf37Q182R | 75.4026_72.6319_72.152_70.976_69.6581 | 75.4026 | 7.8984_7.9038_7.9052_7.9059_7.9071 | 7.8984 |
| C8orf37 | 614477 | Cone-rod dystrophy, Cone-rod retinal dystrophy | Mutant | 207 | C8orf37R177W | 74.411_72.0394_71.4257_68.8905_68.2559 | 74.411 | 7.9014_7.9057_7.9057_7.9069_7.9066 | 7.9014 |
| C9 | 120940 | Macular degeneration, age-related | WT | 538 | C9WT | 80.0804_79.1795_79.047_78.7438_78.596 | 80.0804 | 8.0318_8.0319_8.0317_8.0318_8.0319 | 8.0318 |
| C9 | 120940 | Macular degeneration, age-related | Mutant | 538 | C9P167S | 78.9649_78.168_77.6228_76.413_75.413 | 78.9649 | 7.9442_7.9444_7.9442_7.9448_7.945 | 7.9442 |
| CA4 | 114760 | Retinitis pigmentosa | WT | 266 | CA4NM_000717WT | 94.6392_94.3982_94.3759_94.3282_93.7716 | 94.6392 | 7.7293_7.7342_7.7346_7.7334_7.7349 | 7.7293 |
| CA4 | 114760 | Retinitis pigmentosa | Mutant | 266 | CA4NM_000717M139L | 96.6969_96.6378_94.5945_94.3414_94.1473 | 96.6969 | 7.9774_7.9774_7.9772_7.9781_7.9786 | 7.9774 |
| CABP4 | 608965 | Congenital static night blindness, Congenital static night blindness, 2B | WT | 275 | CABP4NM_145200WT | 64.9706_63.2137_61.86_61.5599_60.1633 | 64.9706 | 7.9842_7.985_7.9863_7.9868_7.9875 | 7.9842 |
| CABP4 | 608965 | Congenital static night blindness, Congenital static night blindness, 2B | Mutant | 275 | CABP4NM_145200R124C | 61.925_60.1554_59.8088_59.2792_57.5781 | 61.925 | 7.9831_7.9833_7.9853_7.9856_7.9864 | 7.9831 |
| CACNA1A | 601011 | Spinocerebellar ataxia 6 | Mutant | 1544 | CACNA1A1545-2506MISSING | 63.4406_62.2153_61.9506_60.9976_60.4794 | 63.4406 | 8.0262_8.0263_8.0262_8.0262_8.0263 | 8.0262 |
| CACNA1F | 300110 | Cone-rod retinal dystrophy, Cone-rod dystrophy, X-linked, 3, Congenital static night blindness, Cone-rod dystrophy, Aland Island eye disease | WT | 1977 | CACNA1FNM_00518WT | 66.7425_66.1122_64.6178_63.2545_63.0344 | 66.7425 | 7.93_7.93_7.93_7.93_7.93 | 7.93 |
| CACNA1F | 300110 | Cone-rod retinal dystrophy, Cone-rod dystrophy, X-linked, 3, Congenital static night blindness, Cone-rod dystrophy, Aland Island eye disease | Mutant | 1977 | CACNA1FNM_00518C1499R | 66.9223_66.1937_64.6489_63.8234_63.4416 | 66.9223 | 8.1811_8.181_8.1811_8.1811_8.1811 | 8.1811 |
| CACNA1F | 300110 | Cone-rod retinal dystrophy, Cone-rod dystrophy, X-linked, 3, Congenital static night blindness, Cone-rod dystrophy, Aland Island eye disease | Mutant | 1977 | CACNA1FNM_00518G369D | 67.273_66.3887_63.6149_63.0342_62.5552 | 67.273 | 7.93_7.93_7.93_7.93_7.93 | 7.93 |
| CACNA2D4 | 608171 | Retinal cone dystrophy | WT | 1118 | CACNA2D4NM_172364WT | 82.7939_82.6053_81.4974_80.4382_79.9082 | 82.7939 | 7.93_7.93_7.93_7.93_7.93 | 7.93 |
| CACNA2D4 | 608171 | Retinal cone dystrophy | Mutant | 1118 | CACNA2D4NM_172364R434T | 83.0288_82.7878_81.1546_80.7893_80.4555 | 83.0288 | 9.5981_9.5988_9.599_9.5991_9.5992 | 9.5981 |
| CAPN3 | 114240 | Muscular dystrophy, limb-girdle | WT | 821 | CAPN3WT | 77.807_77.5899_76.4877_73.6549_72.2567 | 77.807 | 8.0353_8.0354_8.0355_8.0355_8.0356 | 8.0353 |
| CAPN3 | 114240 | Muscular dystrophy, limb-girdle | Mutant | 814 | CAPN3215-221MISSING | 77.083_77.0281_74.3789_71.4271_70.0718 | 77.083 | 8.0371_8.0372_8.0371_8.037_8.0372 | 8.0371 |
| CAPN3 | 114240 | Muscular dystrophy, limb-girdle | Mutant | 821 | CAPN3R748Q | 77.6404_77.5602_77.2256_73.5158_71.014 | 77.6404 | 8.0355_8.0357_8.0356_8.0354_8.0355 | 8.0355 |
| CASK | 300172 | Intellectual developmental disorder and microcephaly with pontine and cerebellar hypoplasia | WT | 921 | CASKWT | 77.3886_75.6336_75.284_74.7986_74.6973 | 77.3886 |  |  |
| CASK | 300172 | Intellectual developmental disorder and microcephaly with pontine and cerebellar hypoplasia | Mutant | 921 | CASKD58E | 78.7976_76.9236_74.4576_73.9777_73.201 | 78.7976 | 5.3351_8.0357_8.0356_8.0356_8.0358 | 5.3351 |
| CASK | 300172 | Intellectual developmental disorder and microcephaly with pontine and cerebellar hypoplasia | Mutant | 921 | CASKD710G | 76.3515_75.8391_74.9134_73.3975_73.3427 | 76.3515 |  |  |
| CASK | 300172 | Intellectual developmental disorder and microcephaly with pontine and cerebellar hypoplasia | Mutant | 126 | CASKE127X | 92.0222_91.8028_91.6594_91.235_88.8185 | 92.0222 | 4.7654_7.9986_7.9986_7.9986_7.9986 | 4.7654 |
| CASK | 300172 | Intellectual developmental disorder and microcephaly with pontine and cerebellar hypoplasia | Mutant | 921 | CASKG659D | 76.323_74.6609_74.1261_73.1124_72.4087 | 76.323 | 5.3424_8.0353_8.0353_8.0354_8.0354 | 5.3424 |
| CASK | 300172 | Intellectual developmental disorder and microcephaly with pontine and cerebellar hypoplasia | Mutant | 921 | CASKL209P | 76.8089_76.3573_75.0907_74.5265_74.4936 | 76.8089 | 5.3682_8.0355_8.0357_8.0357_8.0357 | 5.3682 |
| CASK | 300172 | Intellectual developmental disorder and microcephaly with pontine and cerebellar hypoplasia | Mutant | 921 | CASKM519T | 77.4724_75.1857_73.615_73.6056_73.0524 | 77.4724 | 5.3566_8.0356_8.0357_8.0357_8.0357 | 5.3566 |
| CASK | 300172 | Intellectual developmental disorder and microcephaly with pontine and cerebellar hypoplasia | Mutant | 921 | CASKP396S | 77.4014_76.1172_75.9657_75.553_74.8159 | 77.4014 | 5.5841_8.0142_8.016_8.0164_8.0172 | 5.5841 |
| CASK | 300172 | Intellectual developmental disorder and microcephaly with pontine and cerebellar hypoplasia | Mutant | 691 | CASKQ692X | 75.5243_75.3425_73.7607_72.8348_72.0861 | 75.5243 | 6.6548_8.0373_8.0373_8.0373_8.0375 | 6.6548 |
| CASK | 300172 | Intellectual developmental disorder and microcephaly with pontine and cerebellar hypoplasia | Mutant | 921 | CASKR28L | 76.6739_76.0575_75.7036_74.6383_74.3445 | 76.6739 | 5.3505_8.0357_8.0358_8.0358_8.0358 | 5.3505 |
| CASK | 300172 | Intellectual developmental disorder and microcephaly with pontine and cerebellar hypoplasia | Mutant | 921 | CASKY268H | 76.5065_76.1741_74.7496_74.4533_73.577 | 76.5065 |  |  |
| CAV3 | 601253 | Muscular dystrophy, limb-girdle | WT | 151 | CAV3WT | 89.2337_88.7991_88.6816_88.1699_88.0354 | 89.2337 | 5.2064_7.972_7.97_7.9733_7.9756 | 5.2064 |
| CAV3 | 601253 | Muscular dystrophy, limb-girdle | Mutant | 151 | CAV3N33K | 87.8482_87.4984_86.9435_86.789_86.718 | 87.8482 | 5.1061_7.9663_7.9781_7.9777_7.9732 | 5.1061 |
| CAV3 | 601253 | Muscular dystrophy, limb-girdle | Mutant | 151 | CAV3R27Q | 88.5084_87.7343_87.4498_86.621_86.177 | 88.5084 | 5.125_7.9695_7.9872_7.9678_7.9798 | 5.125 |
| CC2D2A | 612013 | Meckel syndrome, COACH syndrome, Joubert syndrome | WT | 1620 | CC2D2AWT | 69.9616_69.269_69.0089_68.7847_68.2529 | 69.9616 | 7.9845_7.9847_7.9845_7.9849_7.9849 | 7.9845 |
| CC2D2A | 612013 | Meckel syndrome, COACH syndrome, Joubert syndrome | Mutant | 1620 | CC2D2AD1556V | 70.0322_69.1223_69.0277_68.9773_68.6231 | 70.0322 | 7.9475_7.9475_7.9475_7.9475_7.9475 | 7.9475 |
| CC2D2A | 612013 | Meckel syndrome, COACH syndrome, Joubert syndrome | Mutant | 1620 | CC2D2AE1126K | 69.906_68.5183_68.434_68.3074_68.2647 | 69.906 | 7.9475_7.9475_7.9475_7.9475_7.9475 | 7.9475 |
| CC2D2A | 612013 | Meckel syndrome, COACH syndrome, Joubert syndrome | Mutant | 1620 | CC2D2AR1528C | 70.3603_68.8835_68.8318_68.6728_68.1429 | 70.3603 | 7.9475_7.9475_7.9475_7.9475_7.9475 | 7.9475 |
| CDH3 | 114021 | Macular dystrophy | WT | 722 | CDH3WT | 83.8162_83.4675_83.0894_82.7961_82.5774 | 83.8162 | 8.0034_8.004_8.0055_8.0058_8.0063 | 8.0034 |
| CDH3 | 114021 | Macular dystrophy | Mutant | 722 | CDH3N322I | 83.0152_82.112_82.056_81.9762_81.8516 | 83.0152 | 7.9419_7.9421_7.942_7.9421_7.9421 | 7.9419 |
| CDH3 | 114021 | Macular dystrophy | Mutant | 722 | CDH3R503H | 83.9159_83.4199_83.1462_82.7233_82.3619 | 83.9159 | 7.9419_7.942_7.942_7.942_7.942 | 7.9419 |
| CDHR1 | 609502 | Cone-rod retinal dystrophy, Cone-rod dystrophy | WT | 840 | CDHR1NM_033100WT | 80.0811_80.0686_79.9428_79.6115_78.8318 | 80.0811 | 8.1777_8.1778_8.1779_8.1779_8.1778 | 8.1777 |
| CDHR1 | 609502 | Cone-rod retinal dystrophy, Cone-rod dystrophy | WT | 840 | CDHR1WT | 79.3087_79.1387_79.0758_78.741_77.861 | 79.3087 | 8.168_8.1692_8.1699_8.1698_8.171 | 8.168 |
| CDHR1 | 609502 | Cone-rod retinal dystrophy, Cone-rod dystrophy | Mutant | 840 | CDHR1NM_033100R191C | 80.2246_79.8223_79.4357_79.386_79.223 | 80.2246 | 7.9918_8.0322_8.0341_8.0344_8.0342 | 7.9918 |
| CDHR1 | 609502 | Cone-rod retinal dystrophy, Cone-rod dystrophy | Mutant | 840 | CDHR1P574A | 79.4718_78.8175_78.7808_78.7589_77.6855 | 79.4718 | 8.1689_8.1691_8.1689_8.1694_8.17 | 8.1689 |
| CEP164 | 614848 | Nephronophthisis | WT | 1460 | CEP164WT | 61.8447_60.7606_60.599_60.4225_60.109 | 61.8447 | 8.3313_8.3352_8.3364_8.3405_8.344 | 8.3313 |
| CEP164 | 614848 | Nephronophthisis | Mutant | 1460 | CEP164Q11P | 62.2594_60.6577_60.3716_60.1973_59.9379 | 62.2594 | 8.3016_8.3362_8.3366_8.3376_8.3423 | 8.3016 |
| CEP164 | 614848 | Nephronophthisis | Mutant | 1460 | CEP164R93W | 62.2067_60.8123_60.4661_60.2653_59.6881 | 62.2067 | 8.2468_8.249_8.2499_8.2511_8.2557 | 8.2468 |
| CEP290 | 610142 | Meckel syndrome, Leber congenital amaurosis, Senior-Loken syndrome 6, Joubert syndrome 5, Leber congenital amaurosis 10, Bardet-Biedl syndrome 14 | WT | 2479 | CEP290WT | 61.1894_60.6522_58.6525_57.2359_55.5515 | 61.1894 |  |  |
| CEP290 | 610142 | Meckel syndrome, Leber congenital amaurosis, Senior-Loken syndrome 6, Joubert syndrome 5, Leber congenital amaurosis 10, Bardet-Biedl syndrome 14 | Mutant | 2479 | CEP290W7C | 60.7603_60.6305_56.9997_55.0122_53.1251 | 60.7603 |  |  |
| CERKL | 608381 | Retinitis pigmentosa | WT | 532 | CERKLNM_201548WT | 82.6371_82.6172_82.1528_82.1357_82.1193 | 82.6371 | 7.821_7.8205_7.8272_7.8284_7.8329 | 7.821 |
| CERKL | 608381 | Retinitis pigmentosa | Mutant | 216 | CERKLNM_201548L215fs | 79.1446_77.9777_77.0103_76.7275_75.5612 | 79.1446 | 7.9198_7.9201_7.9204_7.9212_7.9206 | 7.9198 |
| CERKL | 608381 | Retinitis pigmentosa | Mutant | 532 | CERKLNM_201548Y522D | 82.7757_82.0354_81.9649_81.9394_81.5782 | 82.7757 | 7.8212_7.8294_7.8302_7.8313_7.8321 | 7.8212 |
| CFH | 134370 | Macular degeneration, age-related, Basal laminar drusen | Mutant | 1213 | CFHNM_00018662I-402H | 78.682_77.4968_76.9606_75.3019_74.1613 | 78.682 | 8.2718_8.2719_8.272_8.2721_8.2721 | 8.2718 |
| CFH | 134370 | Macular degeneration, age-related, Basal laminar drusen | Mutant | 1213 | CFHNM_00018662V-402H | 78.7108_76.6108_75.0951_74.2721_72.8835 | 78.7108 | 8.2717_8.2717_8.272_8.2722_8.2721 | 8.2717 |
| CFH | 134370 | Macular degeneration, age-related, Basal laminar drusen | Mutant | 1213 | CFHNM_00018662V-402Y | 79.2898_76.9596_76.0997_74.9409_73.5632 | 79.2898 | 8.1899_8.1898_8.1899_8.1898_8.1899 | 8.1899 |
| CFI | 217030 | Macular degeneration, age-related | WT | 565 | CFIWT | 85.0106_84.77_84.7341_84.0652_83.1848 | 85.0106 | 7.3792_7.947_7.9476_7.9474_7.9477 | 7.3792 |
| CFI | 217030 | Macular degeneration, age-related | Mutant | 565 | CFIG119R | 84.5138_84.4946_84.4239_84.3058_83.3714 | 84.5138 | 7.8782_8.0333_8.0333_8.0331_8.0334 | 7.8782 |
| CFL2 | 601443 | Nemaline myopathy 7, autosomal recessive, Nemaline myopathy, autosomal recessive | WT | 165 | CFL2WT | 91.1717_89.152_86.6351_85.9924_85.9434 | 91.1717 | 8.1743_8.179_8.1732_8.1757_8.1756 | 8.1743 |
| CFL2 | 601443 | Nemaline myopathy 7, autosomal recessive, Nemaline myopathy, autosomal recessive | Mutant | 165 | CFL2A35T | 91.2023_88.9043_86.7534_86.5184_85.0038 | 91.2023 | 7.9494_8.177_8.1716_8.1751_8.1764 | 7.9494 |
| CFL2 | 601443 | Nemaline myopathy 7, autosomal recessive, Nemaline myopathy, autosomal recessive | Mutant | 165 | CFL2V7M | 91.4833_89.5569_86.7956_86.6867_86.2317 | 91.4833 | 7.9455_7.9485_7.9474_7.9428_7.9516 | 7.9455 |
| CHM | 300390 | Choroideremia | WT | 653 | CHMWT | 80.3526_79.8761_78.6651_78.2539_78.0081 | 80.3526 | 8.032_8.0321_8.0322_8.0321_8.0321 | 8.032 |
| CHM | 300390 | Choroideremia | Mutant | 653 | CHMH507R | 80.162_80.0637_78.4959_78.2096_77.2424 | 80.162 | 8.0321_8.0321_8.032_8.0322_8.032 | 8.0321 |
| CHM | 300390 | Choroideremia | Mutant | 653 | CHMQ471L | 79.804_79.6826_78.7853_78.3741_77.9376 | 79.804 | 8.0321_8.032_8.0321_8.0321_8.0322 | 8.0321 |
| CHMP4B | 610897 | Congenital cataract and developmental cataract | WT | 223 | CHMP4BWT | 79.4767_76.1666_76.1464_71.6689_71.4561 | 79.4767 | 7.9104_7.9126_7.9132_7.913_7.9139 | 7.9104 |
| CHMP4B | 610897 | Congenital cataract and developmental cataract | Mutant | 223 | CHMP4BD129V | 79.9786_77.086_76.4439_72.8251_69.744 | 79.9786 | 7.8859_7.8865_7.8861_7.886_7.8864 | 7.8859 |
| CHMP4B | 610897 | Congenital cataract and developmental cataract | Mutant | 223 | CHMP4BE161K | 78.5115_76.894_75.9688_74.2102_72.0579 | 78.5115 | 7.8823_7.8824_7.8844_7.8848_7.8856 | 7.8823 |
| CHN1 | 118423 | Duane retraction syndrome 2, Congenital fibrosis of the extraocular muscles | WT | 458 | CHN1WT | 84.9122_84.0905_83.4454_82.1414_81.9723 | 84.9122 | 8.0304_8.0306_8.0305_8.0305_8.0306 | 8.0304 |
| CHN1 | 118423 | Duane retraction syndrome 2, Congenital fibrosis of the extraocular muscles | Mutant | 458 | CHN1P252Q | 84.7313_83.7099_82.7773_82.2059_80.3847 | 84.7313 | 8.8249_9.2673_9.2664_9.2672_9.2679 | 8.8249 |
| CHN1 | 118423 | Duane retraction syndrome 2, Congenital fibrosis of the extraocular muscles | Mutant | 458 | CHN1Y143H | 85.4681_85.0273_82.4327_81.5142_80.511 | 85.4681 | 8.0304_8.0304_8.0305_8.0305_8.0305 | 8.0304 |
| CHST6 | 605294 | Macular corneal dystrophy, Corneal dystrophy | WT | 395 | CHST6WT | 90.8111_90.2606_89.8101_89.7648_89.6963 | 90.8111 | 8.0326_8.0327_8.0326_8.0326_8.0328 | 8.0326 |
| CHST6 | 605294 | Macular corneal dystrophy, Corneal dystrophy | Mutant | 395 | CHST6E274K | 90.829_90.4258_90.1221_89.9407_89.6299 | 90.829 | 8.0323_8.0324_8.0324_8.0323_8.0325 | 8.0323 |
| CHST6 | 605294 | Macular corneal dystrophy, Corneal dystrophy | Mutant | 395 | CHST6L200R | 90.8501_90.4836_90.2524_89.9041_89.4754 | 90.8501 | 9.4184_9.4179_9.4181_9.4181_9.4183 | 9.4184 |
| CIB2 | 605564 | Usher syndrome | WT | 187 | CIB2WT | 87.1874_86.5146_85.8292_85.7133_83.9578 | 87.1874 | 7.9082_7.926_7.9208_7.9148_7.9232 | 7.9082 |
| CIB2 | 605564 | Usher syndrome | Mutant | 187 | CIB2E64D | 87.7766_86.3628_86.2618_86.0934_85.2815 | 87.7766 | 7.918_7.9196_7.9216_7.9226_7.9266 | 7.918 |
| CIB2 | 605564 | Usher syndrome | Mutant | 187 | CIB2F91S | 89.339_87.9058_87.0561_87.0297_86.1452 | 89.339 | 7.9264_8.2195_8.2207_8.2197_8.2256 | 7.9264 |
| CISD2 | 611507 | Wolfram syndrome | WT | 134 | CISD2WT | 91.2327_91.2199_90.7032_90.019_89.7501 | 91.2327 | 8.0138_8.0138_8.0138_8.0138_8.0138 | 8.0138 |
| CISD2 | 611507 | Wolfram syndrome | Mutant | 134 | CISD2E37Q | 90.8151_90.7575_90.1681_90.0329_89.4739 | 90.8151 | 8.0139_8.0139_8.0139_8.0139_8.0139 | 8.0139 |
| CLDN19 | 610036 | Hypomagnesemia, renal, with ocular involvement, Weill-marchesani-like syndrome, Hypomagnesemia 5, renal, with ocular involvement | WT | 224 | CLDN19NM_148960WT | 81.0101_80.9077_80.1254_80.0753_79.858 | 81.0101 | 7.7295_7.9678_7.9688_7.9693_7.968 | 7.7295 |
| CLDN19 | 610036 | Hypomagnesemia, renal, with ocular involvement, Weill-marchesani-like syndrome, Hypomagnesemia 5, renal, with ocular involvement | Mutant | 224 | CLDN19NM_148960L90P | 81.22_80.6988_80.1707_80.1689_80.1103 | 81.22 | 7.8715_7.9672_7.9675_7.9686_7.9692 | 7.8715 |
| CLDN19 | 610036 | Hypomagnesemia, renal, with ocular involvement, Weill-marchesani-like syndrome, Hypomagnesemia 5, renal, with ocular involvement | Mutant | 224 | CLDN19NM_148960Q57E | 80.764_80.6908_80.2164_79.9949_79.6178 | 80.764 | 7.7487_7.9685_7.9694_7.9697_7.9698 | 7.7487 |
| CLN3 | 607042 | Ceroid lipofuscinosis, neuronal | WT | 435 | CLN3WT | 81.36_80.1452_78.8185_77.8438_77.2213 | 81.36 | 8.0367_8.0367_8.0369_8.0366_8.0367 | 8.0367 |
| CLN3 | 607042 | Ceroid lipofuscinosis, neuronal | Mutant | 435 | CLN3E295K | 80.7769_79.3588_78.4359_77.459_77.0168 | 80.7769 | 8.0366_8.0367_8.0368_8.0367_8.0368 | 8.0366 |
| CLN3 | 607042 | Ceroid lipofuscinosis, neuronal | Mutant | 435 | CLN3L170P | 80.7661_80.1942_79.3628_78.4813_78.2766 | 80.7661 | 8.0371_8.0368_8.0369_8.0372_8.0369 | 8.0371 |
| CLN5 | 608102 | Ceroid lipofuscinosis, neuronal | WT | 358 | CLN5WT | 80.882_80.8505_80.7382_80.4046_80.3698 | 80.882 | 8.0028_8.0029_8.0028_8.0028_8.0029 | 8.0028 |
| CLN5 | 608102 | Ceroid lipofuscinosis, neuronal | Mutant | 358 | CLN5D230N | 81.7022_81.2934_81.2545_81.1197_81.1083 | 81.7022 | 8.0029_8.0028_8.0028_8.0029_8.0028 | 8.0029 |
| CLN5 | 608102 | Ceroid lipofuscinosis, neuronal | Mutant | 358 | CLN5R63H | 81.1666_81.0_80.7051_80.5326_78.9801 | 81.1666 | 8.0029_8.0028_8.0027_8.0029_8.0028 | 8.0029 |
| CLN6 | 606725 | Ceroid lipofuscinosis, neuronal | WT | 311 | CLN6WT | 86.6898_86.0893_85.6498_85.2589_84.0741 | 86.6898 | 8.1901_8.1902_8.1902_8.1903_8.1903 | 8.1901 |
| CLN6 | 606725 | Ceroid lipofuscinosis, neuronal | Mutant | 310 | CLN6265MISSING | 86.7547_86.0755_85.622_85.1517_83.9364 | 86.7547 | 8.1921_8.1923_8.1925_8.1923_8.1925 | 8.1921 |
| CLN6 | 606725 | Ceroid lipofuscinosis, neuronal | Mutant | 311 | CLN6F234L | 86.7637_85.7423_85.7276_85.3344_84.1984 | 86.7637 | 8.1901_8.1901_8.1901_8.1902_8.1902 | 8.1901 |
| CLN6 | 606725 | Ceroid lipofuscinosis, neuronal | Mutant | 311 | CLN6M241T | 86.5069_86.2107_85.8061_85.7843_84.8843 | 86.5069 | 8.1902_8.1901_8.1901_8.1903_8.1903 | 8.1902 |
| CLN8 | 607837 | Ceroid lipofuscinosis, neuronal | WT | 286 | CLN8WT | 90.0978_89.7514_89.6845_89.6569_89.6104 | 90.0978 | 8.0431_8.0461_8.0468_8.0457_8.0471 | 8.0431 |
| CLN8 | 607837 | Ceroid lipofuscinosis, neuronal | Mutant | 286 | CLN8G237R | 90.3311_90.1641_89.9928_89.733_89.6045 | 90.3311 | 8.0448_8.0442_8.0455_8.0442_8.0462 | 8.0448 |
| CLN8 | 607837 | Ceroid lipofuscinosis, neuronal | Mutant | 286 | CLN8Y158C | 90.1055_89.7999_89.7482_89.6613_88.7807 | 90.1055 | 8.0444_8.0449_8.0444_8.0456_8.0457 | 8.0444 |
| CLRN1 | 606397 | Usher syndrome, Retinitis pigmentosa | WT | 232 | CLRN1WT | 90.6821_89.9211_89.665_89.5673_88.3484 | 90.6821 | 7.9845_7.985_7.9861_7.9865_7.9874 | 7.9845 |
| CLRN1 | 606397 | Usher syndrome, Retinitis pigmentosa | Mutant | 232 | CLRN1L154W | 90.6334_90.119_89.0714_88.9906_88.9413 | 90.6334 | 7.983_7.9847_7.9854_7.9848_7.987 | 7.983 |
| CLRN1 | 606397 | Usher syndrome, Retinitis pigmentosa | Mutant | 232 | CLRN1N48K | 89.7444_89.4405_89.0786_88.9984_87.9412 | 89.7444 | 7.9831_7.9846_7.9855_7.9854_7.9861 | 7.9831 |
| CNGA1 | 123825 | Retinitis pigmentosa | WT | 686 | CNGA1WT | 76.3045_76.2243_75.7684_75.3919_73.742 | 76.3045 | 8.0303_8.0301_8.0303_8.0303_8.0303 | 8.0303 |
| CNGA1 | 123825 | Retinitis pigmentosa | Mutant | 686 | CNGA1S316F | 76.2604_75.5712_75.3431_75.2729_73.7808 | 76.2604 | 8.143_8.0303_8.0303_8.0303_8.0304 | 8.143 |
| CNGA3 | 600053 | Achromatopsia | WT | 694 | CNGA3WT | 74.3725_74.2488_73.7562_73.7232_73.0704 | 74.3725 | 7.269_8.0373_8.0374_8.0373_8.0374 | 7.269 |
| CNGA3 | 600053 | Achromatopsia | Mutant | 694 | CNGA3R377C | 74.3482_74.2188_74.1926_73.6566_73.3009 | 74.3482 | 7.2321_8.0373_8.0374_8.0375_8.0375 | 7.2321 |
| CNGA3 | 600053 | Achromatopsia | Mutant | 694 | CNGA3V529M | 74.7688_74.4555_74.0974_73.7655_73.5243 | 74.7688 | 7.1204_8.0372_8.0373_8.0375_8.0374 | 7.1204 |
| CNGB1 | 600724 | Retinitis pigmentosa | WT | 1251 | CNGB1NM_001297WT | 58.0252_55.8181_55.4448_55.1137_54.8268 | 58.0252 | 8.0973_8.0973_8.0975_8.0977_8.0975 | 8.0973 |
| CNGB1 | 600724 | Retinitis pigmentosa | Mutant | 1251 | CNGB1NM_001297P296L | 57.0898_55.8606_54.7672_54.5812_54.36 | 57.0898 | 8.0971_8.0973_8.0975_8.0976_8.0976 | 8.0971 |
| CNGB3 | 605080 | Wagner syndrome 1, Achromatopsia | WT | 809 | CNGB3NM_019098WT | 68.3495_66.9325_66.4217_66.277_65.8233 | 68.3495 | 8.0367_8.0366_8.0367_8.0367_8.037 | 8.0367 |
| CNGB3 | 605080 | Wagner syndrome 1, Achromatopsia | Mutant | 809 | CNGB3NM_019098G558C | 68.0976_67.1192_67.0193_66.5349_66.3324 | 68.0976 | 8.0368_8.0369_8.0368_8.0369_8.0368 | 8.0368 |
| CNGB3 | 605080 | Wagner syndrome 1, Achromatopsia | Mutant | 809 | CNGB3NM_019098S435F | 67.9307_66.8221_66.1788_65.8847_65.6356 | 67.9307 | 8.0368_8.0369_8.0368_8.0369_8.0368 | 8.0368 |
| CNNM4 | 607805 | Jalili syndrome | WT | 775 | CNNM4WT | 76.9707_76.4718_75.627_75.4271_75.227 | 76.9707 | 7.6881_7.6885_7.6889_7.6889_7.689 | 7.6881 |
| CNNM4 | 607805 | Jalili syndrome | Mutant | 775 | CNNM4L324P | 76.6386_76.1791_75.8614_75.7012_75.3856 | 76.6386 | 7.0674_7.94_7.94_7.94_7.94 | 7.0674 |
| CNNM4 | 607805 | Jalili syndrome | Mutant | 775 | CNNM4S196P | 76.9119_76.0663_75.7856_75.6146_75.2379 | 76.9119 | 6.9908_7.94_7.94_7.94_7.94 | 6.9908 |
| COL11A1 | 120280 | Stickler syndrome, Stickler syndrome, type II, Marshall syndrome | WT | 1052 | COL11A1NM_001854WT | 49.4737_47.6716_46.1506_43.8242_38.7889 | 49.4737 | 2.8803_7.6987_7.7001_7.7011_7.7019 | 2.8803 |
| COL11A1 | 120280 | Stickler syndrome, Stickler syndrome, type II, Marshall syndrome | Mutant | 1052 | COL11A1NM_001854G1516V | 49.3792_47.3632_44.7208_42.9566_39.3732 | 49.3792 | 3.0169_7.6997_7.701_7.7011_7.7014 | 3.0169 |
| COL11A1 | 120280 | Stickler syndrome, Stickler syndrome, type II, Marshall syndrome | Mutant | 1052 | COL11A1NM_001854G625V | 49.3778_47.8324_45.363_41.0911_38.1334 | 49.3778 | 2.8396_7.6975_7.6979_7.698_7.699 | 2.8396 |
| COL11A2 | 120290 | Stickler syndrome, Stickler syndrome, type III, Otospondylomegaepiphyseal dysplasia, autosomal dominant | WT | 1473 | COL11A2NM_080680WT | 48.8256_46.984_46.3582_44.8027_42.9844 | 48.8256 | 8.063_8.0803_8.0914_8.1_8.1013 | 8.063 |
| COL11A2 | 120290 | Stickler syndrome, Stickler syndrome, type III, Otospondylomegaepiphyseal dysplasia, autosomal dominant | Mutant | 1473 | COL11A2NM_080680G808E | 48.9245_47.2356_47.1782_45.4288_43.029 | 48.9245 | 8.2528_8.2707_8.2898_8.2987_8.2994 | 8.2528 |
| COL11A2 | 120290 | Stickler syndrome, Stickler syndrome, type III, Otospondylomegaepiphyseal dysplasia, autosomal dominant | Mutant | 1473 | COL11A2NM_080680P888T | 49.8425_46.8144_46.1065_44.7527_42.5751 | 49.8425 | 8.0608_8.0684_8.0778_8.0816_8.0824 | 8.0608 |
| COL18A1 | 120328 | Knobloch syndrome, type 1 | WT | 1493 | COL18A1NM_030582WT | 54.0305_52.9009_51.9666_51.6692_51.5619 | 54.0305 | 8.2718_8.2775_8.3128_8.3144_8.3173 | 8.2718 |
| COL18A1 | 120328 | Knobloch syndrome, type 1 | Mutant | 1493 | COL18A1NM_030582E184K | 54.7036_53.4623_52.549_52.1834_51.3719 | 54.7036 | 8.2914_8.3007_8.3055_8.3056_8.3232 | 8.2914 |
| COL2A1 | 120140 | Syndromic retinal disease, Myopic syndrome, Stickler syndrome, Kniest dysplasia | WT | 1306 | COL2A1NM_001844WT | 55.1191_52.2164_51.0323_51.0038_45.8841 | 55.1191 | 6.1319_7.8969_7.8973_7.9_7.903 | 6.1319 |
| COL2A1 | 120140 | Syndromic retinal disease, Myopic syndrome, Stickler syndrome, Kniest dysplasia | Mutant | 1306 | COL2A1NM_001844G909C | 55.3167_52.555_51.9718_51.8797_46.5288 | 55.3167 | 6.1421_7.9014_7.904_7.9045_7.9053 | 6.1421 |
| COL2A1 | 120140 | Syndromic retinal disease, Myopic syndrome, Stickler syndrome, Kniest dysplasia | Mutant | 1306 | COL2A1NM_001844L667F | 54.4565_52.6274_52.2855_51.1801_46.1946 | 54.4565 | 6.1203_8.1827_8.1828_8.1828_8.1828 | 6.1203 |
| COL2A1 | 120140 | Syndromic retinal disease, Myopic syndrome, Stickler syndrome, Kniest dysplasia | Mutant | 1306 | COL2A1NM_001844R719C | 54.5729_51.5478_51.4687_50.7518_44.9226 | 54.5729 | 6.1227_7.8915_7.8963_7.9005_7.9013 | 6.1227 |
| COL2A1 | 120140 | Syndromic retinal disease, Myopic syndrome, Stickler syndrome, Kniest dysplasia | Mutant | 1306 | COL2A1NM_001844R904C | 55.0679_52.3755_51.1967_50.5202_45.93 | 55.0679 | 6.1316_8.1826_8.1828_8.1827_8.1827 | 6.1316 |
| COL6A1 | 120220 | Ullrich congenital muscular dystrophy, Ullrich congenital muscular dystrophy 1 | WT | 1009 | COL6A1WT | 71.9051_69.9058_69.902_68.6714_68.5652 | 71.9051 | 8.2437_8.2438_8.2437_8.2438_8.2437 | 8.2437 |
| COL6A1 | 120220 | Ullrich congenital muscular dystrophy, Ullrich congenital muscular dystrophy 1 | Mutant | 1009 | COL6A1G284R | 71.6421_70.2043_69.9246_68.9692_68.474 | 71.6421 | 8.2437_8.2437_8.2437_8.2437_8.2438 | 8.2437 |
| COL6A1 | 120220 | Ullrich congenital muscular dystrophy, Ullrich congenital muscular dystrophy 1 | Mutant | 1009 | COL6A1G290R | 70.174_69.8261_69.4133_68.5606_67.4434 | 70.174 | 8.2437_8.2437_8.2437_8.2438_8.2437 | 8.2437 |
| COL6A2 | 120240 | Ullrich congenital muscular dystrophy, Ullrich congenital muscular dystrophy 1 | WT | 999 | COL6A2WT | 68.877_67.4631_67.2539_66.1642_65.4001 | 68.877 | 7.7881_7.9714_7.9984_7.9992_7.9993 | 7.7881 |
| COL6A2 | 120240 | Ullrich congenital muscular dystrophy, Ullrich congenital muscular dystrophy 1 | Mutant | 998 | COL6A2897MISSING | 67.7618_67.0372_66.5652_65.6544_65.2204 | 67.7618 | 8.041_8.041_8.041_8.0411_8.0411 | 8.041 |
| COL6A2 | 120240 | Ullrich congenital muscular dystrophy, Ullrich congenital muscular dystrophy 1 | Mutant | 999 | COL6A2R498H | 68.382_67.9428_67.5743_66.3811_65.9068 | 68.382 | 8.0372_8.0373_8.0373_8.0373_8.0373 | 8.0372 |
| COL8A2 | 120252 | Corneal endothelial dystrophy, Corneal dystrophy, Corneal dystrophy, Fuchs endothelial, 1 | WT | 675 | COL8A2WT | 61.312_60.2538_60.1288_58.4811_56.9241 | 61.312 | 8.038_8.038_8.038_8.0381_8.0382 | 8.038 |
| COL8A2 | 120252 | Corneal endothelial dystrophy, Corneal dystrophy, Corneal dystrophy, Fuchs endothelial, 1 | Mutant | 675 | COL8A2Q455K | 61.7692_61.137_59.3186_58.3484_55.9633 | 61.7692 | 8.038_8.038_8.0381_8.038_8.038 | 8.038 |
| COL8A2 | 120252 | Corneal endothelial dystrophy, Corneal dystrophy, Corneal dystrophy, Fuchs endothelial, 1 | Mutant | 675 | COL8A2R304Q | 61.683_60.5745_59.5682_59.1445_55.7912 | 61.683 | 8.038_8.0379_8.038_8.0381_8.0381 | 8.038 |
| COL9A1 | 120210 | Stickler syndrome, type IV, Stickler syndrome, Stickler syndrome | WT | 898 | COL9A1WT | 63.0799_60.6875_60.5861_58.4827_56.1728 | 63.0799 | 8.0395_8.042_8.0454_8.0459_8.0474 | 8.0395 |
| COL9A1 | 120210 | Stickler syndrome, type IV, Stickler syndrome, Stickler syndrome | Mutant | 271 | COL9A1R295X | 87.2163_87.1156_87.1046_87.0218_86.8466 | 87.2163 | 7.8825_7.8969_7.8986_7.8996_7.9007 | 7.8825 |
| COL9A2 | 120260 | Stickler syndrome, Stickler syndrome, Stickler syndrome, type IV, Stickler syndrome | WT | 666 | COL9A2NM_001852WT | 55.8443_54.6053_54.3083_49.6602_47.1135 | 55.8443 | 8.0335_8.0336_8.0335_8.0336_8.0335 | 8.0335 |
| COL9A2 | 120260 | Stickler syndrome, Stickler syndrome, Stickler syndrome, type IV, Stickler syndrome | Mutant | 666 | COL9A2NM_001852Q326W | 56.9937_55.3737_54.774_52.519_49.8586 | 56.9937 | 8.0335_8.0335_8.0333_8.0336_8.0336 | 8.0335 |
| CRB1 | 604210 | Retinitis pigmentosa, Leber congenital amaurosis, Pigmented paravenous chorioretinal atrophy, Choroidal retinopathy, Leber congenital amaurosis 8 | WT | 1381 | CRB1NM_201253WT | 76.61_75.6167_75.2626_73.3091_73.226 | 76.61 | 7.9286_7.93_7.93_7.93_7.93 | 7.9286 |
| CRB1 | 604210 | Retinitis pigmentosa, Leber congenital amaurosis, Pigmented paravenous chorioretinal atrophy, Choroidal retinopathy, Leber congenital amaurosis 8 | Mutant | 1381 | CRB1NM_201253C1163F | 77.0875_75.7232_75.1418_74.1363_74.0793 | 77.0875 | 7.9285_7.93_7.93_7.93_7.93 | 7.9285 |
| CRB1 | 604210 | Retinitis pigmentosa, Leber congenital amaurosis, Pigmented paravenous chorioretinal atrophy, Choroidal retinopathy, Leber congenital amaurosis 8 | Mutant | 1381 | CRB1NM_201253C948Y | 75.3348_74.705_73.8884_73.2685_72.6553 | 75.3348 | 7.9285_7.93_7.93_7.93_7.93 | 7.9285 |
| CRB1 | 604210 | Retinitis pigmentosa, Leber congenital amaurosis, Pigmented paravenous chorioretinal atrophy, Choroidal retinopathy, Leber congenital amaurosis 8 | Mutant | 1381 | CRB1NM_201253R764C | 76.8544_75.9194_74.0261_73.3076_73.2087 | 76.8544 | 7.9285_7.93_7.93_7.93_7.93 | 7.9285 |
| CRB1 | 604210 | Retinitis pigmentosa, Leber congenital amaurosis, Pigmented paravenous chorioretinal atrophy, Choroidal retinopathy, Leber congenital amaurosis 8 | Mutant | 1381 | CRB1NM_201253T745M | 76.8728_76.4856_75.2875_74.0222_72.6729 | 76.8728 | 7.9286_7.93_7.93_7.93_7.93 | 7.9286 |
| CRX | 602225 | Cone-rod retinal dystrophy, Leber congenital amaurosis 7, Leber congenital amaurosis, Cone-rod dystrophy, Cone-rod retinal dystrophy-2 | WT | 299 | CRXNM_000554WT | 61.4047_56.9639_56.8371_56.68_55.7272 | 61.4047 | 8.1891_8.1891_8.1891_8.1892_8.1891 | 8.1891 |
| CRX | 602225 | Cone-rod retinal dystrophy, Leber congenital amaurosis 7, Leber congenital amaurosis, Cone-rod dystrophy, Cone-rod retinal dystrophy-2 | Mutant | 299 | CRXNM_000554R41Q | 61.1742_56.2195_56.1415_56.0725_56.0329 | 61.1742 | 8.1891_8.1891_8.1891_8.1892_8.1891 | 8.1891 |
| CRX | 602225 | Cone-rod retinal dystrophy, Leber congenital amaurosis 7, Leber congenital amaurosis, Cone-rod dystrophy, Cone-rod retinal dystrophy-2 | Mutant | 299 | CRXNM_000554R90W | 61.2122_56.273_55.9356_55.8565_55.293 | 61.2122 | 8.1892_8.1892_8.1892_8.1893_8.1891 | 8.1892 |
| CRYAA | 123580 | Congenital cataract and developmental cataract, Cataract, Cataract 9 | WT | 173 | CRYAANM_000394WT | 75.6314_74.9393_74.7527_73.5404_71.8959 | 75.6314 | 7.8618_8.1014_8.5272_8.5275_8.5323 | 7.8618 |
| CRYAA | 123580 | Congenital cataract and developmental cataract, Cataract, Cataract 9 | Mutant | 173 | CRYAANM_000394R116C | 73.9284_73.7952_72.3611_71.5943_70.9121 | 73.9284 | 8.157_8.1625_8.1593_8.163_8.1748 | 8.157 |
| CRYAA | 123580 | Congenital cataract and developmental cataract, Cataract, Cataract 9 | Mutant | 173 | CRYAANM_000394R116H | 74.9345_72.7937_72.2635_72.0174_71.6929 | 74.9345 | 8.1641_8.1642_8.1695_8.1554_8.1737 | 8.1641 |
| CRYAA | 123580 | Congenital cataract and developmental cataract, Cataract, Cataract 9 | Mutant | 173 | CRYAANM_000394R12C | 74.2256_74.1356_73.7074_72.797_71.2654 | 74.2256 | 8.1588_8.1629_8.1694_8.1637_8.169 | 8.1588 |
| CRYAA | 123580 | Congenital cataract and developmental cataract, Cataract, Cataract 9 | Mutant | 173 | CRYAANM_000394R21W | 74.7007_73.4046_72.8331_72.5627_72.5451 | 74.7007 | 8.163_8.1637_8.1633_8.1687_8.1728 | 8.163 |
| CRYAB | 123590 | Congenital cataract and developmental cataract | WT | 175 | CRYABWT | 73.917_73.166_72.4663_72.323_71.9226 | 73.917 | 8.1257_8.1259_8.1307_8.1239_8.1302 | 8.1257 |
| CRYAB | 123590 | Congenital cataract and developmental cataract | Mutant | 175 | CRYABR120G | 72.1009_70.9976_70.7092_70.6097_69.9161 | 72.1009 | 8.1095_8.1334_8.1469_8.1427_8.1574 | 8.1095 |
| CRYBA1 | 123610 | Congenital cataract and developmental cataract | WT | 215 | CRYBA1WT | 87.5281_87.4058_87.0399_86.9622_86.8134 | 87.5281 | 7.9267_7.9268_7.9274_7.9288_7.929 | 7.9267 |
| CRYBA1 | 123610 | Congenital cataract and developmental cataract | Mutant | 216 | CRYBA1C185EfsX33 | 74.1358_73.1312_72.9871_72.7084_71.9908 | 74.1358 | 7.9233_7.9229_7.923_7.9234_7.9242 | 7.9233 |
| CRYBA1 | 123610 | Congenital cataract and developmental cataract | Mutant | 217 | CRYBA1E197VfsX22 | 78.2635_77.7497_77.2482_77.2061_77.0345 | 78.2635 | 7.9304_7.929_7.9306_7.931_7.9319 | 7.9304 |
| CRYBA1 | 123610 | Congenital cataract and developmental cataract | Mutant | 214 | CRYBA1G91del | 87.7142_87.1423_86.7137_86.5712_86.4833 | 87.7142 | 7.4808_7.4847_7.4876_7.4861_7.4865 | 7.4808 |
| CRYBA1 | 123610 | Congenital cataract and developmental cataract | Mutant | 217 | CRYBA1Q203DfsX16 | 80.6792_80.1321_80.1132_79.2292_78.2633 | 80.6792 | 7.5208_7.5191_7.5227_7.5234_7.5345 | 7.5208 |
| CRYBA1 | 123610 | Congenital cataract and developmental cataract | Mutant | 202 | CRYBA1Q203X | 85.7096_84.9987_84.269_84.1931_84.1326 | 85.7096 | 7.8879_7.8891_7.8888_7.889_7.8893 | 7.8879 |
| CRYBA1 | 123610 | Congenital cataract and developmental cataract | Mutant | 215 | CRYBA1R114C | 87.7714_87.3871_87.3579_87.0819_86.9428 | 87.7714 | 7.926_7.927_7.927_7.9268_7.9275 | 7.926 |
| CRYBA1 | 123610 | Congenital cataract and developmental cataract | Mutant | 215 | CRYBA1S209W | 86.3081_85.8783_84.9054_84.36_84.3219 | 86.3081 | 7.926_7.9265_7.9264_7.9264_7.9269 | 7.926 |
| CRYBA4 | 123631 | Cataract 23, Congenital cataract and developmental cataract | WT | 195 | CRYBA4WT | 91.2038_90.6165_90.4725_89.3723_88.7572 | 91.2038 | 7.5244_7.7593_7.7665_7.7762_7.7621 | 7.5244 |
| CRYBA4 | 123631 | Cataract 23, Congenital cataract and developmental cataract | Mutant | 195 | CRYBA4F94S | 92.017_91.2787_90.2132_89.6663_89.2744 | 92.017 | 7.4542_8.2601_8.2664_8.2646_8.2689 | 7.4542 |
| CRYBA4 | 123631 | Cataract 23, Congenital cataract and developmental cataract | Mutant | 195 | CRYBA4G64W | 91.5906_91.2546_90.7246_89.8719_88.908 | 91.5906 | 7.5131_8.2621_8.2626_8.2632_8.2667 | 7.5131 |
| CRYBA4 | 123631 | Cataract 23, Congenital cataract and developmental cataract | Mutant | 195 | CRYBA4L69P | 91.5304_91.0396_90.5722_90.461_89.2803 | 91.5304 | 7.4973_8.2664_8.2718_8.273_8.2783 | 7.4973 |
| CRYBB1 | 600929 | Congenital cataract and developmental cataract | WT | 251 | CRYBB1WT | 80.5503_79.3762_78.1458_77.3589_77.1841 | 80.5503 | 7.9455_7.9635_7.9647_7.9645_7.9654 | 7.9455 |
| CRYBB1 | 600929 | Congenital cataract and developmental cataract | Mutant | 251 | CRYBB1V96F | 79.0127_78.1574_78.0246_77.3965_77.2823 | 79.0127 | 7.77_7.8103_7.832_7.8492_7.8568 | 7.77 |
| CRYBB2 | 123620 | Congenital cataract and developmental cataract | WT | 204 | CRYBB2NM_000496WT | 89.2516_89.0939_88.2737_88.2197_87.9266 | 89.2516 | 7.4589_7.4584_7.4646_7.4655_7.4648 | 7.4589 |
| CRYBB2 | 123620 | Congenital cataract and developmental cataract | Mutant | 204 | CRYBB2NM_000496R81P | 90.2063_88.745_88.456_88.0029_87.8852 | 90.2063 | 7.8945_7.8947_7.8953_7.8958_7.8969 | 7.8945 |
| CRYBB2 | 123620 | Congenital cataract and developmental cataract | Mutant | 204 | CRYBB2S31W | 88.8992_88.8787_88.6138_88.5941_88.5536 | 88.8992 | 7.4563_7.4576_7.4618_7.461_7.4698 | 7.4563 |
| CRYBB3 | 123630 | Congenital cataract and developmental cataract, Cataract | WT | 211 | CRYBB3WT | 87.3064_86.7004_86.0904_85.9313_85.5329 | 87.3064 | 6.7848_7.4454_7.4462_7.451_7.4552 | 6.7848 |
| CRYBB3 | 123630 | Congenital cataract and developmental cataract, Cataract | Mutant | 211 | CRYBB3G165R | 87.2206_86.6451_86.3991_85.7442_85.4558 | 87.2206 | 6.7781_7.902_7.9025_7.9029_7.9057 | 6.7781 |
| CRYBB3 | 123630 | Congenital cataract and developmental cataract, Cataract | Mutant | 211 | CRYBB3V194E | 87.2105_86.3882_86.3432_86.2005_85.2004 | 87.2105 | 6.8209_7.4572_7.4591_7.463_7.4647 | 6.8209 |
| CRYGA | 123660 | Cataract | WT | 174 | CRYGAWT | 96.1404_96.0971_95.9094_95.4601_95.3561 | 96.1404 | 7.7675_7.7392_7.7444_7.7546_7.749 | 7.7675 |
| CRYGA | 123660 | Cataract | Mutant | 174 | CRYGAR48H | 96.2879_96.2029_95.6924_95.6384_94.702 | 96.2879 | 8.1665_8.1763_8.1756_8.1715_8.1769 | 8.1665 |
| CRYGB | 123670 | Congenital cataract and developmental cataract | WT | 175 | CRYGBWT | 96.828_96.6561_96.6361_96.4255_96.0839 | 96.828 | 7.7488_7.7457_7.7286_7.7427_7.7439 | 7.7488 |
| CRYGB | 123670 | Congenital cataract and developmental cataract | Mutant | 43 | CRYGBP24PfsX19 | 69.1418_68.8054_64.9322_61.5902_58.7525 | 69.1418 | 8.5783_8.5939_8.6568_8.617_8.6336 | 8.5783 |
| CRYGC | 123680 | Albinism, oculocutaneous, type III, Cataract, Congenital cataract and developmental cataract | WT | 173 | CRYGCWT | 96.7814_96.5648_96.4812_96.3656_96.0267 | 96.7814 | 8.1738_8.1736_8.1754_8.1737_8.1758 | 8.1738 |
| CRYGC | 123680 | Albinism, oculocutaneous, type III, Cataract, Congenital cataract and developmental cataract | Mutant | 142 | CRYGC144-174MISSING | 96.1045_95.9513_95.283_94.0762_93.8913 | 96.1045 | 6.5158_7.7923_7.7931_7.794_7.7936 | 6.5158 |
| CRYGC | 123680 | Albinism, oculocutaneous, type III, Cataract, Congenital cataract and developmental cataract | Mutant | 100 | CRYGCD65TfsX38 | 76.1698_73.6993_71.7958_71.6154_68.1109 | 76.1698 | 8.0646_8.0659_8.0669_8.068_8.0677 | 8.0646 |
| CRYGC | 123680 | Albinism, oculocutaneous, type III, Cataract, Congenital cataract and developmental cataract | Mutant | 173 | CRYGCG129C | 96.0526_95.7712_95.6931_95.384_94.955 | 96.0526 | 8.1738_8.1735_8.1743_8.1744_8.1746 | 8.1738 |
| CRYGC | 123680 | Albinism, oculocutaneous, type III, Cataract, Congenital cataract and developmental cataract | Mutant | 161 | CRYGCR142AfsX22 | 88.485_88.4469_85.9745_85.251_83.1685 | 88.485 | 8.0846_8.087_8.0901_8.0857_8.0841 | 8.0846 |
| CRYGC | 123680 | Albinism, oculocutaneous, type III, Cataract, Congenital cataract and developmental cataract | Mutant | 173 | CRYGCR168W | 96.5917_96.4088_96.2183_95.8958_95.7113 | 96.5917 | 7.741_7.7454_7.7349_7.7325_7.7267 | 7.741 |
| CRYGC | 123680 | Albinism, oculocutaneous, type III, Cataract, Congenital cataract and developmental cataract | Mutant | 173 | CRYGCR48H | 96.8242_96.5532_96.4152_96.1558_95.8346 | 96.8242 | 7.7459_8.1699_8.1734_8.17_8.1731 | 7.7459 |
| CRYGC | 123680 | Albinism, oculocutaneous, type III, Cataract, Congenital cataract and developmental cataract | Mutant | 173 | CRYGCS166F | 95.248_95.2042_94.4288_94.358_94.2421 | 95.248 | 7.7496_7.7442_7.7482_7.7456_7.7454 | 7.7496 |
| CRYGC | 123680 | Albinism, oculocutaneous, type III, Cataract, Congenital cataract and developmental cataract | Mutant | 173 | CRYGCS78F | 95.4401_95.0691_94.6533_94.6239_94.5053 | 95.4401 | 7.7371_7.732_7.7369_7.7417_7.7468 | 7.7371 |
| CRYGC | 123680 | Albinism, oculocutaneous, type III, Cataract, Congenital cataract and developmental cataract | Mutant | 173 | CRYGCT5P | 96.8578_96.4878_96.4722_96.2145_96.1287 | 96.8578 | 7.7458_7.7438_7.7486_7.7382_7.746 | 7.7458 |
| CRYGC | 123680 | Albinism, oculocutaneous, type III, Cataract, Congenital cataract and developmental cataract | Mutant | 142 | CRYGCY144X | 96.1733_96.0879_95.2051_94.0748_93.5908 | 96.1733 | 6.5185_7.7929_7.7927_7.7947_7.7927 | 6.5185 |
| CRYGC | 123680 | Albinism, oculocutaneous, type III, Cataract, Congenital cataract and developmental cataract | Mutant | 173 | CRYGCY46D | 96.8154_96.4966_96.343_96.3291_95.9138 | 96.8154 | 8.1763_8.1797_8.1795_8.1793_8.1763 | 8.1763 |
| CRYGD | 123690 | Congenital cataract and developmental cataract | WT | 173 | CRYGDWT | 97.5688_97.5119_96.5608_96.2148_96.2076 | 97.5688 | 8.1761_8.1828_8.1814_8.1697_8.1818 | 8.1761 |
| CRYGD | 123690 | Congenital cataract and developmental cataract | Mutant | 138 | CRYGD140-174MISSING | 97.3229_96.8549_90.9803_90.6945_88.9377 | 97.3229 | 8.3864_8.3509_8.4156_8.3901_8.4314 | 8.3864 |
| CRYGD | 123690 | Congenital cataract and developmental cataract | Mutant | 155 | CRYGD157-174MISSING | 96.7824_96.2362_94.6541_93.8543_93.4592 | 96.7824 | 6.5802_7.8651_7.8697_7.8663_7.8732 | 6.5802 |
| CRYGD | 123690 | Congenital cataract and developmental cataract | Mutant | 54 | CRYGD56-174MISSING | 97.3439_97.0352_93.2874_92.7565_90.9855 | 97.3439 | 7.9638_8.0023_7.9749_7.9891_8.0525 | 7.9638 |
| CRYGD | 123690 | Congenital cataract and developmental cataract | Mutant | 173 | CRYGDC111M | 97.4883_97.4258_96.625_96.1991_96.1567 | 97.4883 | 7.7314_7.7391_7.7342_7.7412_7.7377 | 7.7314 |
| CRYGD | 123690 | Congenital cataract and developmental cataract | Mutant | 173 | CRYGDE107A | 97.5101_97.4174_96.42_96.0761_96.0245 | 97.5101 | 8.1746_8.1776_8.1859_8.1806_8.1827 | 8.1746 |
| CRYGD | 123690 | Congenital cataract and developmental cataract | Mutant | 173 | CRYGDN125D | 97.5794_97.4997_96.6588_96.3707_96.2407 | 97.5794 | 8.179_8.1755_8.1805_8.1793_8.1824 | 8.179 |
| CRYGD | 123690 | Congenital cataract and developmental cataract | Mutant | 173 | CRYGDN161D | 97.5296_97.462_96.6426_96.3841_96.3526 | 97.5296 | 8.1676_8.1775_8.1726_8.1753_8.178 | 8.1676 |
| CRYGD | 123690 | Congenital cataract and developmental cataract | Mutant | 173 | CRYGDP24S | 97.5817_97.4887_96.5564_96.2989_96.1304 | 97.5817 | 8.1751_8.1778_8.176_8.1785_8.1758 | 8.1751 |
| CRYGD | 123690 | Congenital cataract and developmental cataract | Mutant | 173 | CRYGDP24T | 97.5791_97.5249_96.4976_96.3252_96.1461 | 97.5791 | 7.7329_7.7585_7.758_7.7513_7.7581 | 7.7329 |
| CRYGD | 123690 | Congenital cataract and developmental cataract | Mutant | 173 | CRYGDP24T-R37S | 97.3001_97.1839_96.3842_96.1416_96.0442 | 97.3001 | 8.1765_8.1781_8.1842_8.1826_8.1784 | 8.1765 |
| CRYGD | 123690 | Congenital cataract and developmental cataract | Mutant | 173 | CRYGDR15C | 97.5243_97.483_96.6444_96.3738_96.2334 | 97.5243 | 8.178_8.1787_8.176_8.1797_8.1806 | 8.178 |
| CRYGD | 123690 | Congenital cataract and developmental cataract | Mutant | 173 | CRYGDR37S | 97.5159_97.4473_96.4685_96.2441_96.1381 | 97.5159 | 8.178_8.1749_8.1762_8.1793_8.1785 | 8.178 |
| CRYGD | 123690 | Congenital cataract and developmental cataract | Mutant | 173 | CRYGDR59H | 97.1425_97.1278_96.5658_96.2457_96.1785 | 97.1425 | 8.1688_8.1768_8.1742_8.1781_8.1763 | 8.1688 |
| CRYGD | 123690 | Congenital cataract and developmental cataract | Mutant | 173 | CRYGDW43R | 97.269_97.1438_96.3598_96.1706_95.9024 | 97.269 | 8.1804_8.1754_8.1747_8.1813_8.1777 | 8.1804 |
| CRYGS | 123730 | Congenital cataract and developmental cataract | WT | 177 | CRYGSWT | 95.6243_95.1536_94.7311_94.6849_94.3862 | 95.6243 | 8.1246_8.1635_8.1625_8.1593_8.1613 | 8.1246 |
| CRYGS | 123730 | Congenital cataract and developmental cataract | Mutant | 177 | CRYGSG18V | 95.5294_95.4262_94.98_94.9077_94.9023 | 95.5294 | 8.1288_8.1604_8.1602_8.1552_8.1595 | 8.1288 |
| CST3 | 604312 | Macular degeneration, age-related | WT | 120 | CST3WT | 92.9229_92.4009_91.3914_90.7714_90.1918 | 92.9229 | 7.7809_8.1303_8.1304_8.1303_8.1305 | 7.7809 |
| CST3 | 604312 | Macular degeneration, age-related | Mutant | 120 | CST3L94Q | 92.8462_92.4877_90.3848_90.3371_89.601 | 92.8462 | 7.8822_8.1302_8.1303_8.1302_8.1302 | 7.8822 |
| CTC1 | 613129 | Cerebroretinal microangiopathy with calcifications and cysts | WT | 1217 | CTC1WT | 77.1477_76.9987_76.9357_76.7953_75.9848 | 77.1477 | 8.25_8.2501_8.2503_8.2502_8.2502 | 8.25 |
| CTC1 | 613129 | Cerebroretinal microangiopathy with calcifications and cysts | Mutant | 1210 | CTC11196-1202Missing | 76.7019_76.0712_75.9158_75.8759_75.7274 | 76.7019 | 7.9972_7.9975_7.9976_7.9975_7.9976 | 7.9972 |
| CTC1 | 613129 | Cerebroretinal microangiopathy with calcifications and cysts | Mutant | 1216 | CTC1985Missing | 76.6181_76.268_76.0536_75.8751_75.3878 | 76.6181 | 8.2767_8.2769_8.2772_8.2771_8.277 | 8.2767 |
| CTC1 | 613129 | Cerebroretinal microangiopathy with calcifications and cysts | Mutant | 1217 | CTC1R975G | 76.9947_76.7973_76.7248_76.6883_76.4464 | 76.9947 | 8.25_8.2499_8.25_8.25_8.2501 | 8.25 |
| CTNNB1 | 116806 | Exudative vitreoretinopathy 7 | WT | 780 | CTNNB1WT | 80.4425_80.0464_79.4402_79.1619_79.0279 | 80.4425 | 6.6349_7.94_7.94_7.94_7.94 | 6.6349 |
| CTNNB1 | 116806 | Exudative vitreoretinopathy 7 | Mutant | 780 | CTNNB1L388P | 80.0094_79.8125_78.9199_78.8222_78.7976 | 80.0094 | 6.5881_7.94_7.94_7.94_7.94 | 6.5881 |
| CTNNB1 | 116806 | Exudative vitreoretinopathy 7 | Mutant | 780 | CTNNB1R710C | 81.0321_80.9231_80.3684_80.213_79.3153 | 81.0321 | 6.5217_7.94_7.94_7.94_7.94 | 6.5217 |
| CTSD | 116840 | Ceroid lipofuscinosis, neuronal | WT | 348 | CTSDWT | 93.8675_93.4847_93.0971_93.033_92.714 | 93.8675 | 6.2804_8.008_8.008_8.008_8.008 | 6.2804 |
| CTSD | 116840 | Ceroid lipofuscinosis, neuronal | Mutant | 348 | CTSDW383C | 93.8449_93.1553_93.0758_92.8371_92.5979 | 93.8449 | 6.2846_8.008_8.008_8.008_8.008 | 6.2846 |
| CX3CR1 | 601470 | Macular degeneration, age-related | WT | 355 | CX3CR1WT | 79.9752_79.2743_78.8158_78.0716_76.0837 | 79.9752 | 8.0029_8.0029_8.0029_8.0029_8.003 | 8.0029 |
| CX3CR1 | 601470 | Macular degeneration, age-related | Mutant | 355 | CX3CR1T280M | 79.243_79.0292_78.2476_77.3365_76.6983 | 79.243 | 8.0028_8.0028_8.0029_8.0029_8.003 | 8.0028 |
| CX3CR1 | 601470 | Macular degeneration, age-related | Mutant | 355 | CX3CR1V249I | 79.9355_78.4604_78.4334_78.1251_77.0128 | 79.9355 | 8.0029_8.0029_8.0029_8.0029_8.0029 | 8.0029 |
| CYP1B1 | 601771 | Peters, abnormal, Primary open angle glaucoma, Congenital central leukoplakia of cornea | WT | 543 | CYP1B1NM_000104WT | 92.6383_92.3775_91.8109_91.5785_91.1738 | 92.6383 | 8.0319_8.0319_8.0319_8.0319_8.0318 | 8.0319 |
| CYP1B1 | 601771 | Peters, abnormal, Primary open angle glaucoma, Congenital central leukoplakia of cornea | Mutant | 543 | CYP1B1A443G | 92.422_92.2839_91.9697_91.5859_91.2736 | 92.422 | 8.0319_8.0318_8.032_8.032_8.032 | 8.0319 |
| CYP1B1 | 601771 | Peters, abnormal, Primary open angle glaucoma, Congenital central leukoplakia of cornea | Mutant | 543 | CYP1B1E387K | 92.5625_92.4243_91.8055_91.4582_90.8442 | 92.5625 | 7.9416_7.9418_7.9417_7.9417_7.9417 | 7.9416 |
| CYP1B1 | 601771 | Peters, abnormal, Primary open angle glaucoma, Congenital central leukoplakia of cornea | Mutant | 543 | CYP1B1NM_000104E229K | 92.5749_92.3686_91.9609_91.6003_91.316 | 92.5749 | 8.0319_8.0319_8.0319_8.0319_8.032 | 8.0319 |
| CYP1B1 | 601771 | Peters, abnormal, Primary open angle glaucoma, Congenital central leukoplakia of cornea | Mutant | 543 | CYP1B1NM_000104G61E | 92.0162_91.8213_91.0216_90.75_90.6944 | 92.0162 | 8.0319_8.0319_8.0319_8.0319_8.032 | 8.0319 |
| CYP1B1 | 601771 | Peters, abnormal, Primary open angle glaucoma, Congenital central leukoplakia of cornea | Mutant | 543 | CYP1B1NM_000104I471S | 92.427_92.3076_91.8475_91.3761_91.2344 | 92.427 | 8.0319_8.0318_8.032_8.0319_8.032 | 8.0319 |
| CYP1B1 | 601771 | Peters, abnormal, Primary open angle glaucoma, Congenital central leukoplakia of cornea | Mutant | 543 | CYP1B1NM_000104L107V | 92.5444_92.4937_92.2007_91.7657_90.4259 | 92.5444 | 8.0319_8.0319_8.0319_8.0319_8.0319 | 8.0319 |
| CYP1B1 | 601771 | Peters, abnormal, Primary open angle glaucoma, Congenital central leukoplakia of cornea | Mutant | 543 | CYP1B1NM_000104R368H | 92.4836_92.3385_92.1073_91.5734_91.5242 | 92.4836 | 7.9366_7.9364_7.9364_7.9369_7.9367 | 7.9366 |
| CYP1B1 | 601771 | Peters, abnormal, Primary open angle glaucoma, Congenital central leukoplakia of cornea | Mutant | 543 | CYP1B1NM_000104R390H | 92.7693_92.5596_91.8345_91.5783_91.5192 | 92.7693 | 8.0318_8.0319_8.032_8.0319_8.032 | 8.0318 |
| CYP1B1 | 601771 | Peters, abnormal, Primary open angle glaucoma, Congenital central leukoplakia of cornea | Mutant | 543 | CYP1B1R469W | 92.4624_92.278_91.7822_91.4851_91.4423 | 92.4624 | 8.0319_8.0319_8.032_8.032_8.0319 | 8.0319 |
| CYP1B1 | 601771 | Peters, abnormal, Primary open angle glaucoma, Congenital central leukoplakia of cornea | Mutant | 543 | CYP1B1Y81N | 92.4855_92.3681_91.893_91.6632_91.2697 | 92.4855 | 8.0319_8.0318_8.0319_8.0319_8.0319 | 8.0319 |
| CYP4V2 | 608614 | Bietti crystalline corneoretinal dystrophy, Retinal dystrophy | WT | 525 | CYP4V2WT | 90.866_90.7373_90.6447_90.3416_90.1662 | 90.866 | 7.9369_7.9372_7.9373_7.9373_7.9375 | 7.9369 |
| CYP4V2 | 608614 | Bietti crystalline corneoretinal dystrophy, Retinal dystrophy | Mutant | 525 | CYP4V2D324V | 90.0145_89.8601_89.6791_89.5542_88.863 | 90.0145 | 7.8493_7.8505_7.8512_7.8524_7.8523 | 7.8493 |
| CYP4V2 | 608614 | Bietti crystalline corneoretinal dystrophy, Retinal dystrophy | Mutant | 525 | CYP4V2E79D | 90.4149_90.1267_90.0949_89.9125_89.874 | 90.4149 | 7.8508_7.8519_7.8528_7.8523_7.8533 | 7.8508 |
| CYP4V2 | 608614 | Bietti crystalline corneoretinal dystrophy, Retinal dystrophy | Mutant | 525 | CYP4V2G61S | 90.4338_89.7845_89.5169_89.0957_88.4701 | 90.4338 | 7.937_7.9372_7.9374_7.9373_7.9376 | 7.937 |
| CYP4V2 | 608614 | Bietti crystalline corneoretinal dystrophy, Retinal dystrophy | Mutant | 525 | CYP4V2H331P | 90.1131_89.9377_89.8176_89.4507_88.6246 | 90.1131 | 7.8521_7.8536_7.8536_7.8543_7.8551 | 7.8521 |
| CYP4V2 | 608614 | Bietti crystalline corneoretinal dystrophy, Retinal dystrophy | Mutant | 525 | CYP4V2I111T | 91.377_90.9276_90.5662_90.4955_89.9625 | 91.377 | 7.851_7.8511_7.851_7.854_7.854 | 7.851 |
| CYP4V2 | 608614 | Bietti crystalline corneoretinal dystrophy, Retinal dystrophy | Mutant | 525 | CYP4V2M123V | 89.8646_89.8408_89.8206_89.5115_89.1546 | 89.8646 | 7.8521_7.8529_7.8534_7.8536_7.8542 | 7.8521 |
| CYP4V2 | 608614 | Bietti crystalline corneoretinal dystrophy, Retinal dystrophy | Mutant | 525 | CYP4V2P396L | 90.9505_90.6814_90.343_90.051_89.7388 | 90.9505 | 7.8529_7.853_7.8533_7.8535_7.8546 | 7.8529 |
| CYP4V2 | 608614 | Bietti crystalline corneoretinal dystrophy, Retinal dystrophy | Mutant | 525 | CYP4V2R400C | 90.6043_90.5086_89.9567_89.9267_89.5946 | 90.6043 | 7.9357_7.9362_7.9362_7.9362_7.9361 | 7.9357 |
| CYP4V2 | 608614 | Bietti crystalline corneoretinal dystrophy, Retinal dystrophy | Mutant | 525 | CYP4V2R400H | 90.5996_90.3647_90.0941_90.037_89.8562 | 90.5996 | 7.8482_7.8513_7.8518_7.8528_7.8525 | 7.8482 |
| CYP4V2 | 608614 | Bietti crystalline corneoretinal dystrophy, Retinal dystrophy | Mutant | 525 | CYP4V2W44R | 90.3014_90.2453_90.0263_89.604_89.2531 | 90.3014 | 7.8486_7.8489_7.8517_7.8533_7.8539 | 7.8486 |
| CYP4V2 | 608614 | Bietti crystalline corneoretinal dystrophy, Retinal dystrophy | Mutant | 525 | CYP4V2Y219H | 90.8518_89.9661_89.8326_89.5845_89.3388 | 90.8518 | 7.9368_7.9372_7.9372_7.9371_7.9374 | 7.9368 |
| DCN | 125255 | Corneal dystrophy, congenital stromal | WT | 329 | DCNWT | 93.9511_93.6195_92.1585_92.104_91.6487 | 93.9511 | 8.191_8.1911_8.1911_8.191_8.1911 | 8.191 |
| DCN | 125255 | Corneal dystrophy, congenital stromal | Mutant | 296 | DCNS323fsX5 | 91.9766_91.4333_91.3441_91.2185_91.0658 | 91.9766 | 8.1774_8.1774_8.1774_8.1774_8.1775 | 8.1774 |
| DHCR7 | 602858 | Smith-Lemli-Opitz syndrome | WT | 475 | DHCR7WT | 91.2912_91.2128_91.097_90.4652_90.0906 | 91.2912 | 6.3485_9.2684_9.269_9.2686_9.2687 | 6.3485 |
| DHCR7 | 602858 | Smith-Lemli-Opitz syndrome | Mutant | 475 | DHCR7R404C | 91.4755_91.4174_90.9523_90.5453_90.3121 | 91.4755 | 5.7806_8.0339_8.0339_8.0338_8.034 | 5.7806 |
| DHCR7 | 602858 | Smith-Lemli-Opitz syndrome | Mutant | 475 | DHCR7T93M | 91.4125_90.9366_90.8715_90.1709_89.7402 | 91.4125 | 5.8312_8.0338_8.0338_8.0339_8.0339 | 5.8312 |
| DHDDS | 608172 | Retinitis pigmentosa | WT | 333 | DHDDSWT | 94.4452_93.7815_93.5424_93.5189_93.174 | 94.4452 | 8.1843_8.1842_8.1843_8.1843_8.1842 | 8.1843 |
| DHDDS | 608172 | Retinitis pigmentosa | Mutant | 333 | DHDDSK42E | 94.1862_93.5718_93.5707_93.2661_92.5833 | 94.1862 | 8.1843_8.1843_8.1842_8.1842_8.1842 | 8.1843 |
| DHDDS | 608172 | Retinitis pigmentosa | Mutant | 333 | DHDDSR211Q | 94.3337_93.7944_93.5357_93.3618_93.0293 | 94.3337 | 8.1842_8.1841_8.1842_8.1842_8.1843 | 8.1842 |
| DIP2B | 611379 | Optic neuritis | WT | 1576 | DIP2BWT | 78.38_78.2124_78.0149_77.6174_77.2209 | 78.38 | 8.0281_8.0281_8.0282_8.0283_8.0282 | 8.0281 |
| DMPK | 605377 | Myotonic dystrophy | WT | 629 | DMPKWT | 77.9608_77.8573_75.1131_74.7018_74.0994 | 77.9608 | 6.3738_8.0342_8.0342_8.0342_8.0342 | 6.3738 |
| DMPK | 605377 | Myotonic dystrophy | Mutant | 629 | DMPKK100A | 77.5747_76.6336_74.5994_73.9235_70.9214 | 77.5747 | 5.6937_7.9492_7.9546_7.9555_7.9564 | 5.6937 |
| DNAJC5 | 611203 | Ceroid lipofuscinosis, neuronal | WT | 198 | DNAJC5WT | 74.4903_73.6226_73.2262_71.8725_71.6398 | 74.4903 | 7.8337_7.8368_7.8388_7.8435_7.8449 | 7.8337 |
| DNAJC5 | 611203 | Ceroid lipofuscinosis, neuronal | Mutant | 197 | DNAJC5116MISSING | 72.6196_72.1086_71.882_70.3763_70.0607 | 72.6196 | 8.2786_8.2786_8.2834_8.2896_8.2906 | 8.2786 |
| DNAJC5 | 611203 | Ceroid lipofuscinosis, neuronal | Mutant | 198 | DNAJC5L115R | 73.502_72.1886_70.7713_70.0053_69.1775 | 73.502 | 7.8897_7.8888_7.8889_7.8901_7.8905 | 7.8897 |
| DNM1L | 603850 | Encephalopathy, lethal, due to defective mitochondrial peroxisomal fission 1 | WT | 736 | DNM1LWT | 77.2715_76.8569_76.7277_76.0172_74.7796 | 77.2715 | 7.9694_7.9755_7.9758_7.9768_7.9772 | 7.9694 |
| DNM1L | 603850 | Encephalopathy, lethal, due to defective mitochondrial peroxisomal fission 1 | Mutant | 736 | DNM1LA395D | 77.2763_77.1974_76.2241_75.9449_74.8514 | 77.2763 | 7.8606_7.8615_7.8623_7.8625_7.8623 | 7.8606 |
| DNM1L | 603850 | Encephalopathy, lethal, due to defective mitochondrial peroxisomal fission 1 | Mutant | 736 | DNM1LG362D | 76.9584_76.7666_76.0531_75.5393_74.5526 | 76.9584 | 7.9798_7.988_7.9882_7.9887_7.9888 | 7.9798 |
| DRD5 | 126453 | Blepharospasm, primary benign | WT | 477 | DRD5WT | 69.7733_69.4707_69.4306_68.3579_68.2057 | 69.7733 | 6.1036_8.0332_8.0333_8.0333_8.0333 | 6.1036 |
| DRD5 | 126453 | Blepharospasm, primary benign | Mutant | 477 | DRD5N7Q | 69.4972_68.9547_68.6325_68.1704_67.9878 | 69.4972 | 6.0738_9.2645_9.2653_9.2661_9.2663 | 6.0738 |
| EFEMP1 | 601548 | Retinal dystrophy | WT | 476 | EFEMP1NM_001039348WT | 79.0745_77.9744_77.8035_76.6526_76.4257 | 79.0745 | 5.306_8.0405_8.0405_8.0406_8.0406 | 5.306 |
| EFEMP1 | 601548 | Retinal dystrophy | Mutant | 476 | EFEMP1NM_001039348M59L | 78.8588_77.7796_77.6546_77.3248_76.4935 | 78.8588 | 5.4261_8.0407_8.0406_8.0407_8.0406 | 5.4261 |
| ELOVL4 | 605512 | Spinocerebellar ataxia 34, Stargardt disease | WT | 314 | ELOVL4WT | 83.661_83.2644_83.1137_82.8635_81.4429 | 83.661 | 8.1894_8.1893_8.1894_8.1894_8.1893 | 8.1894 |
| ELOVL4 | 605512 | Spinocerebellar ataxia 34, Stargardt disease | Mutant | 271 | ELOVL4797-801-del | 90.2414_89.2084_87.8321_85.8968_85.0662 | 90.2414 | 8.0084_8.0091_8.0089_8.0091_8.01 | 8.0084 |
| EMD | 300384 | Emery-Dreifuss muscular dystrophy 1, X-linked, Emery-Dreifuss muscular dystrophy, X-linked | WT | 254 | EMDWT | 60.6564_57.7607_56.7239_56.6925_55.8832 | 60.6564 | 7.9657_7.9667_7.9666_7.9662_7.9669 | 7.9657 |
| EMD | 300384 | Emery-Dreifuss muscular dystrophy 1, X-linked, Emery-Dreifuss muscular dystrophy, X-linked | Mutant | 254 | EMDP183H | 60.0483_57.3428_56.4527_55.5312_55.2536 | 60.0483 | 7.9604_7.9631_7.9628_7.9637_7.9658 | 7.9604 |
| EMD | 300384 | Emery-Dreifuss muscular dystrophy 1, X-linked, Emery-Dreifuss muscular dystrophy, X-linked | Mutant | 254 | EMDQ133H | 60.1375_57.3016_56.2061_56.016_55.1212 | 60.1375 | 7.9658_7.9648_7.9684_7.9687_7.9698 | 7.9658 |
| EPHA2 | 176946 | Congenital cataract and developmental cataract, Cataract | WT | 953 | EPHA2WT | 84.2333_81.6679_81.1078_81.0329_80.1859 | 84.2333 |  |  |
| EPHA2 | 176946 | Congenital cataract and developmental cataract, Cataract | Mutant | 953 | EPHA2G948W | 84.2532_82.1152_81.2857_80.7227_80.2698 | 84.2532 |  |  |
| EPHA2 | 176946 | Congenital cataract and developmental cataract, Cataract | Mutant | 953 | EPHA2T940I | 84.3829_81.7904_81.4356_81.2253_80.564 | 84.3829 |  |  |
| ERBB3 | 190151 | DOOR syndrome | WT | 1323 | ERBB3NM_001982WT | 71.0102_69.6833_67.304_67.1827_66.9503 | 71.0102 | 6.7387_8.1791_8.1791_8.1793_8.1793 | 6.7387 |
| ERBB3 | 190151 | DOOR syndrome | Mutant | 1323 | ERBB3NM_001982A1337T | 71.2721_69.9966_67.7399_66.8116_66.4678 | 71.2721 | 6.7403_8.1792_8.1793_8.1793_8.1793 | 6.7403 |
| ERCC2 | 126340 | Cerebrooculofacioskeletal syndrome, Cockayne syndrome | WT | 760 | ERCC2WT | 88.2778_87.3569_84.2046_82.3562_82.3274 | 88.2778 | 4.3576_8.0172_8.0173_8.018_8.018 | 4.3576 |
| ERCC2 | 126340 | Cerebrooculofacioskeletal syndrome, Cockayne syndrome | Mutant | 760 | ERCC2L461V | 88.7275_87.5615_84.4545_83.4088_81.8858 | 88.7275 | 4.2944_8.0179_8.0181_8.0181_8.0184 | 4.2944 |
| ERCC2 | 126340 | Cerebrooculofacioskeletal syndrome, Cockayne syndrome | Mutant | 760 | ERCC2R112H | 88.5527_87.0667_83.2487_81.7589_81.617 | 88.5527 | 4.3383_8.0176_8.0181_8.0186_8.0187 | 4.3383 |
| ERCC3 | 133510 | Cockayne syndrome | WT | 782 | ERCC3WT | 76.4084_74.6796_71.2099_70.4667_65.1393 | 76.4084 | 6.6163_7.94_7.94_7.94_7.94 | 6.6163 |
| ERCC3 | 133510 | Cockayne syndrome | Mutant | 782 | ERCC3F99S | 76.2463_74.2531_71.8963_70.1157_65.8725 | 76.2463 | 6.5598_7.94_7.94_7.94_7.94 | 6.5598 |
| ERCC3 | 133510 | Cockayne syndrome | Mutant | 782 | ERCC3T119P | 76.0296_73.92_70.7408_69.9902_66.3524 | 76.0296 | 6.5662_7.94_7.94_7.94_7.94 | 6.5662 |
| ERCC4 | 133520 | Fanconi anemia, complementation group Q | WT | 916 | ERCC4WT | 74.7071_74.2474_72.9968_72.3321_71.1823 | 74.7071 | 5.646_8.0456_8.0456_8.0456_8.0456 | 5.646 |
| ERCC4 | 133520 | Fanconi anemia, complementation group Q | Mutant | 916 | ERCC4R689S | 76.2907_74.3809_74.1185_72.3042_70.6667 | 76.2907 | 6.7669_8.0102_8.0106_8.0112_8.0112 | 6.7669 |
| ERCC4 | 133520 | Fanconi anemia, complementation group Q | Mutant | 916 | ERCC4R799W | 76.364_74.1961_73.0856_72.8428_70.325 | 76.364 | 6.7625_8.0101_8.0109_8.0111_8.0111 | 6.7625 |
| ERCC5 | 133530 | Cockayne syndrome, Cerebrooculofacioskeletal syndrome | WT | 1186 | ERCC5WT | 56.5622_56.5315_55.0076_54.8758_54.8378 | 56.5622 | 8.23_8.2302_8.2303_8.2304_8.2306 | 8.23 |
| ERCC5 | 133530 | Cockayne syndrome, Cerebrooculofacioskeletal syndrome | Mutant | 1186 | ERCC5A28D | 56.6502_56.6392_55.2647_55.1716_55.0776 | 56.6502 | 8.1935_8.1936_8.1936_8.1937_8.1938 | 8.1935 |
| ERCC5 | 133530 | Cockayne syndrome, Cerebrooculofacioskeletal syndrome | Mutant | 1186 | ERCC5A792V | 57.1088_56.1825_55.1491_55.0322_54.373 | 57.1088 | 8.2303_8.2304_8.2305_8.2304_8.2305 | 8.2303 |
| ERCC6 | 609413 | Cerebrooculofacioskeletal syndrome, Macular degeneration, age-related, Cockayne syndrome | WT | 1493 | ERCC6WT | 60.6065_58.7469_57.8858_57.7582_56.0792 | 60.6065 | 8.3123_8.3209_8.3748_8.3783_8.3827 | 8.3123 |
| ERCC6 | 609413 | Cerebrooculofacioskeletal syndrome, Macular degeneration, age-related, Cockayne syndrome | Mutant | 1493 | ERCC6L871P | 60.3099_59.0147_58.4917_57.9784_56.7019 | 60.3099 | 8.3294_8.3522_8.3698_8.3929_8.4052 | 8.3294 |
| ERCC6 | 609413 | Cerebrooculofacioskeletal syndrome, Macular degeneration, age-related, Cockayne syndrome | Mutant | 1493 | ERCC6L987P | 60.2142_58.8864_58.1533_57.7189_55.3745 | 60.2142 | 8.1776_8.1883_8.2024_8.2076_8.2379 | 8.1776 |
| ERCC8 | 609412 | Cockayne syndrome | WT | 396 | ERCC8WT | 87.9994_87.8269_87.7647_87.0489_86.4255 | 87.9994 | nan_8.81_8.81_8.81_8.81 | nan |
| ERCC8 | 609412 | Cockayne syndrome | Mutant | 396 | ERCC8A160V | 92.354_91.5291_88.0469_85.2774_83.4583 | 92.354 | 8.0387_8.0387_8.0388_8.0388_8.0387 | 8.0387 |
| ERCC8 | 609412 | Cockayne syndrome | Mutant | 396 | ERCC8W361C | 92.1931_91.7993_87.723_87.0935_86.1878 | 92.1931 | 9.4215_9.4226_9.4217_nan_9.4195 | 9.4215 |
| EYA1 | 601653 | Branchiootorenal syndrome | WT | 592 | EYA1WT | 65.4598_63.0279_61.6151_61.552_61.201 | 65.4598 | 8.0389_8.039_8.0391_8.039_8.0392 | 8.0389 |
| EYA1 | 601653 | Branchiootorenal syndrome | Mutant | 592 | EYA1R440Q | 65.5318_63.318_61.7015_61.3758_61.185 | 65.5318 | 8.0388_8.0391_8.0391_8.0389_8.0393 | 8.0388 |
| EYA1 | 601653 | Branchiootorenal syndrome | Mutant | 592 | EYA1S242G | 65.7372_62.8616_61.7757_61.6611_61.5646 | 65.7372 | 8.0391_8.039_8.039_8.039_8.0391 | 8.0391 |
| EYS | 612424 | Retinitis pigmentosa | Mutant | 1656 | EYSNM_001142800A1636fs | 52.3844_50.014_49.065_47.8673_47.7496 | 52.3844 | 7.938_7.9381_7.9381_7.9381_7.9381 | 7.938 |
| FAM161A | 613596 | Retinitis pigmentosa | WT | 660 | FAM161AWT | 66.8377_65.2688_65.0655_64.7366_62.5356 | 66.8377 | 8.0466_8.0466_8.0466_8.0465_8.0467 | 8.0466 |
| FAM161A | 613596 | Retinitis pigmentosa | Mutant | 314 | FAM161A315-660missing | 68.143_67.7122_67.3185_66.6253_65.0515 | 68.143 | 8.1891_8.189_8.189_8.189_8.1891 | 8.1891 |
| FBLN5 | 604580 | Macular degeneration, age-related | WT | 425 | FBLN5WT | 85.8059_85.5082_84.5754_84.2887_83.054 | 85.8059 | nan_nan_nan_nan_nan | nan |
| FBLN5 | 604580 | Macular degeneration, age-related | Mutant | 425 | FBLN5G267S | 85.3795_85.2708_83.9197_83.8416_81.9072 | 85.3795 | nan_nan_nan_nan_nan | nan |
| FGFR3 | 134934 | LADD syndrome, Crouzon syndrome with acanthosis nigricans | WT | 784 | FGFR3WT | 74.6733_73.6806_73.6722_73.1358_72.4666 | 74.6733 | 6.609_8.0424_8.0425_8.0425_8.0425 | 6.609 |
| FGFR3 | 134934 | LADD syndrome, Crouzon syndrome with acanthosis nigricans | Mutant | 784 | FGFR3A391E | 74.8543_73.8582_72.7549_72.7233_72.0604 | 74.8543 | 6.5754_8.0425_8.0425_8.0424_8.0426 | 6.5754 |
| FGFR3 | 134934 | LADD syndrome, Crouzon syndrome with acanthosis nigricans | Mutant | 784 | FGFR3D513N | 74.6957_73.8394_73.6941_71.3942_71.1861 | 74.6957 | 6.642_8.0426_8.0426_8.0424_8.0424 | 6.642 |
| FKTN | 607440 | Walker Warburg syndrome | WT | 461 | FKTNWT | 92.0472_91.8106_91.5277_91.4111_91.3847 | 92.0472 | 8.0295_8.0295_8.0296_8.0295_8.0296 | 8.0295 |
| FKTN | 607440 | Walker Warburg syndrome | Mutant | 460 | FKTNA170E | 91.9145_91.3902_91.3097_91.2526_91.1929 | 91.9145 | 8.0361_8.0362_8.0362_8.0363_8.0362 | 8.0361 |
| FKTN | 607440 | Walker Warburg syndrome | Mutant | 461 | FKTNC250G | 92.0663_91.8555_91.4524_91.4292_91.1496 | 92.0663 | 8.0295_8.0295_8.0296_8.0295_8.0296 | 8.0295 |
| FKTN | 607440 | Walker Warburg syndrome | Mutant | 461 | FKTNY371C | 91.8255_91.6011_91.4504_91.3785_91.3038 | 91.8255 | 8.029_8.0295_8.0295_8.0295_8.0295 | 8.029 |
| FLVCR1 | 609144 | Ataxia, posterior column, with retinitis pigmentosa | WT | 555 | FLVCR1WT | 77.7985_76.3079_76.1305_75.1891_74.6116 | 77.7985 | 7.5091_8.0323_8.0324_8.0342_8.0343 | 7.5091 |
| FLVCR1 | 609144 | Ataxia, posterior column, with retinitis pigmentosa | Mutant | 555 | FLVCR1C192R | 76.7612_76.437_75.6659_74.7278_73.9851 | 76.7612 | 7.9347_7.9345_7.9388_7.9395_7.9395 | 7.9347 |
| FLVCR1 | 609144 | Ataxia, posterior column, with retinitis pigmentosa | Mutant | 555 | FLVCR1N121D | 77.4878_75.6243_75.3828_74.7471_74.0644 | 77.4878 | 7.9336_7.9338_7.9339_7.9341_7.934 | 7.9336 |
| FOXC1 | 601090 | Iris hypoplasia 1, Axenfeld-Rieger syndrome, Axenfeld-Rieger syndrome 3 | WT | 553 | FOXC1WT | 53.7076_49.1708_48.5555_47.9421_46.9589 | 53.7076 | 5.7565_8.0321_8.0321_8.0321_8.032 | 5.7565 |
| FOXC1 | 601090 | Iris hypoplasia 1, Axenfeld-Rieger syndrome, Axenfeld-Rieger syndrome 3 | Mutant | 553 | FOXC1F112S | 53.1945_49.1167_48.2196_47.1658_47.1378 | 53.1945 | 5.7783_8.032_8.0322_8.0322_8.0321 | 5.7783 |
| FOXC1 | 601090 | Iris hypoplasia 1, Axenfeld-Rieger syndrome, Axenfeld-Rieger syndrome 3 | Mutant | 553 | FOXC1M161K | 53.6493_50.0255_49.052_47.8719_47.6722 | 53.6493 | 6.2342_7.9776_7.9952_7.9979_8.0041 | 6.2342 |
| FOXC1 | 601090 | Iris hypoplasia 1, Axenfeld-Rieger syndrome, Axenfeld-Rieger syndrome 3 | Mutant | 553 | FOXC1S131L | 54.0553_48.8061_47.9824_47.7999_47.2425 | 54.0553 | 5.7723_8.0321_8.0321_8.0322_8.0321 | 5.7723 |
| FOXD3 | 611539 | Aniridia | WT | 478 | FOXD3WT | 55.1674_51.91_51.1792_50.875_49.6388 | 55.1674 | 7.4652_9.383_9.3842_9.4435_9.4476 | 7.4652 |
| FOXD3 | 611539 | Aniridia | Mutant | 478 | FOXD3N173H | 54.8554_51.6521_51.188_51.1261_49.4821 | 54.8554 | 6.966_8.0346_8.0344_8.0345_8.0346 | 6.966 |
| FOXD3 | 611539 | Aniridia | Mutant | 482 | FOXD3R273_G276dup | 55.0752_51.8166_51.3269_50.6077_49.6981 | 55.0752 | 8.0316_8.0316_8.0316_8.0315_8.0317 | 8.0316 |
| FOXE3 | 601094 | Peters, abnormal, Anterior segment dysgenesis 1, multiple subtypes, Coloboma lentis, Anterior segment dysgenesis, Cataract 34 | WT | 319 | FOXE3WT | 65.4726_59.5326_59.3393_59.0971_57.0306 | 65.4726 | 8.1837_8.184_8.1839_8.1841_8.184 | 8.1837 |
| FOXE3 | 601094 | Peters, abnormal, Anterior segment dysgenesis 1, multiple subtypes, Coloboma lentis, Anterior segment dysgenesis, Cataract 34 | Mutant | 319 | FOXE3E103K | 64.977_59.6011_59.2317_59.0187_57.3058 | 64.977 | 8.1838_8.1839_8.1839_8.1839_8.1839 | 8.1838 |
| FOXE3 | 601094 | Peters, abnormal, Anterior segment dysgenesis 1, multiple subtypes, Coloboma lentis, Anterior segment dysgenesis, Cataract 34 | Mutant | 319 | FOXE3N117K | 64.5344_59.4191_59.1769_58.5543_56.8523 | 64.5344 | 8.184_8.1839_8.184_8.1839_8.1839 | 8.184 |
| FOXE3 | 601094 | Peters, abnormal, Anterior segment dysgenesis 1, multiple subtypes, Coloboma lentis, Anterior segment dysgenesis, Cataract 34 | Mutant | 319 | FOXE3R90L | 65.0138_59.9896_59.3427_59.2588_58.1458 | 65.0138 | 8.1838_8.1839_8.1839_8.1839_8.1839 | 8.1838 |
| FOXL2 | 605597 | Palpebral fissure syndrome, Blepharophimosis, epicanthus inversus, and ptosis, type 1, Baraitser-Winter syndrome | WT | 376 | FOXL2WT | 59.7456_55.1348_54.8278_54.3714_52.9655 | 59.7456 | 7.8257_7.8269_7.8302_7.8281_7.8313 | 7.8257 |
| FOXL2 | 605597 | Palpebral fissure syndrome, Blepharophimosis, epicanthus inversus, and ptosis, type 1, Baraitser-Winter syndrome | Mutant | 376 | FOXL2R103C | 60.3614_55.0974_54.7241_54.0664_52.9455 | 60.3614 | 7.8301_7.8275_7.8262_7.8308_7.8331 | 7.8301 |
| FOXL2 | 605597 | Palpebral fissure syndrome, Blepharophimosis, epicanthus inversus, and ptosis, type 1, Baraitser-Winter syndrome | Mutant | 376 | FOXL2S217F | 59.5449_55.0386_54.6452_54.0533_53.464 | 59.5449 | 7.818_7.8257_7.8326_7.833_7.8378 | 7.818 |
| FRMD7 | 300628 | Nystagmus | WT | 714 | FRMD7WT | 63.2347_59.9896_59.5097_59.451_58.7449 | 63.2347 | 8.0259_8.2397_8.2398_8.2398_8.2397 | 8.0259 |
| FRMD7 | 300628 | Nystagmus | Mutant | 714 | FRMD7C271Y | 62.0025_59.8128_59.7999_59.7154_58.5481 | 62.0025 | 8.0474_8.2398_8.2398_8.24_8.2398 | 8.0474 |
| FRMD7 | 300628 | Nystagmus | Mutant | 714 | FRMD7G24R | 62.3788_60.3733_60.1536_60.0126_60.0028 | 62.3788 | 7.9554_8.238_8.2398_8.2398_8.2398 | 7.9554 |
| FSCN2 | 607643 | Retinitis pigmentosa | WT | 492 | FSCN2NM_012418WT | 93.6496_93.6418_93.2439_93.1391_92.7617 | 93.6496 | 9.2398_9.2399_9.2403_9.2406_9.2406 | 9.2398 |
| FSCN2 | 607643 | Retinitis pigmentosa | Mutant | 492 | FSCN2NM_012418G350R | 93.7994_93.5122_93.3504_92.7812_92.7425 | 93.7994 | 8.0421_8.0421_8.0423_8.0422_8.0421 | 8.0421 |
| FSCN2 | 607643 | Retinitis pigmentosa | Mutant | 492 | FSCN2NM_012418S70L | 93.62_93.3851_93.0872_92.7684_92.3214 | 93.62 | 9.2328_9.2327_9.2331_9.233_9.2328 | 9.2328 |
| FTO | 610966 | Growth retardation, developmental delay, facial dysmorphism | WT | 505 | FTOWT | 91.1468_91.1265_89.9295_89.1497_88.9211 | 91.1468 | 9.2411_9.2416_9.2414_9.2419_9.2411 | 9.2411 |
| FTO | 610966 | Growth retardation, developmental delay, facial dysmorphism | Mutant | 505 | FTOR316Q | 91.2144_91.0842_89.9678_89.4401_89.3656 | 91.2144 | 8.2155_8.2153_8.2155_8.2157_8.2306 | 8.2155 |
| FTO | 610966 | Growth retardation, developmental delay, facial dysmorphism | Mutant | 505 | FTOS319F | 91.205_91.073_89.6311_88.7935_88.7066 | 91.205 | 9.2415_9.2416_9.2408_9.2413_9.2419 | 9.2415 |
| FXN | 606829 | Friedreich ataxia | WT | 130 | FXNWT | 93.4309_93.4077_93.3684_93.1001_93.0002 | 93.4309 | 8.0079_8.0079_8.0079_8.0086_8.0159 | 8.0079 |
| FXN | 606829 | Friedreich ataxia | Mutant | 130 | FXND122Y | 92.9674_92.6334_92.6217_92.6022_92.5734 | 92.9674 | 8.0075_8.0075_8.0075_8.0079_8.012 | 8.0075 |
| FXN | 606829 | Friedreich ataxia | Mutant | 130 | FXNI154F | 93.3909_93.3704_93.1854_93.0156_92.8211 | 93.3909 | 8.0075_8.0075_8.0075_8.0083_8.0081 | 8.0075 |
| FYCO1 | 607182 | Congenital cataract and developmental cataract | WT | 1477 | FYCO1WT | 72.7776_71.6518_71.3449_71.1996_68.3465 | 72.7776 | 8.2073_8.2083_8.211_8.2133_8.2147 | 8.2073 |
| FYCO1 | 607182 | Congenital cataract and developmental cataract | Mutant | 1477 | FYCO1L1376P | 72.069_71.3398_70.1131_70.0823_69.655 | 72.069 | 8.2057_8.2135_8.2169_8.2248_8.2276 | 8.2057 |
| FZD4 | 604579 | Retinopathy of prematurity, Familial exudative vitreoretinopathy | WT | 501 | FZD4WT | 86.9309_86.88_85.6959_85.3379_84.5574 | 86.9309 | 8.2365_8.2365_8.2365_8.2365_8.2365 | 8.2365 |
| FZD4 | 604579 | Retinopathy of prematurity, Familial exudative vitreoretinopathy | Mutant | 501 | FZD4H69Y | 86.4783_86.2054_85.3571_85.1129_84.789 | 86.4783 | 9.2382_9.238_9.2387_9.2387_9.2392 | 9.2382 |
| FZD4 | 604579 | Retinopathy of prematurity, Familial exudative vitreoretinopathy | Mutant | 501 | FZD4M105V | 86.863_86.8325_85.8887_84.966_84.4841 | 86.863 | 8.2364_8.2363_8.2364_8.2364_8.2364 | 8.2364 |
| GALT | 606999 | Galactosemia | WT | 379 | GALTWT | 91.8001_91.4635_90.6624_90.4194_90.3715 | 91.8001 | 4.3166_7.8122_7.8121_7.8127_7.8131 | 4.3166 |
| GALT | 606999 | Galactosemia | Mutant | 379 | GALTN314D | 91.9268_91.47_90.9151_90.5618_90.3919 | 91.9268 | 4.2787_7.8045_7.8049_7.806_7.8063 | 4.2787 |
| GALT | 606999 | Galactosemia | Mutant | 379 | GALTQ188R | 92.0385_91.8321_91.3612_91.1909_90.9251 | 92.0385 | 4.2813_7.8094_7.8103_7.8111_7.8114 | 4.2813 |
| GCNT2 | 600429 | Congenital cataract and developmental cataract | WT | 402 | GCNT2WT | 91.862_91.5663_91.55_91.5055_91.2797 | 91.862 | 9.4193_9.419_9.4197_9.4206_9.4192 | 9.4193 |
| GCNT2 | 600429 | Congenital cataract and developmental cataract | Mutant | 402 | GCNT2G350E | 92.1046_91.4859_91.2583_91.1719_91.0278 | 92.1046 | 9.4207_9.4197_9.4195_9.4203_9.4205 | 9.4207 |
| GCNT2 | 600429 | Congenital cataract and developmental cataract | Mutant | 402 | GCNT2R385H | 91.7501_91.5784_91.3641_91.2238_91.1755 | 91.7501 | 8.0347_8.0346_8.0347_8.0347_8.0346 | 8.0347 |
| GDF3 | 606522 | Microphthalmia | WT | 114 | GDF3WT | 89.7182_89.6385_89.0734_88.4867_88.4636 | 89.7182 | 8.0099_8.0104_8.0115_8.0107_8.012 | 8.0099 |
| GDF3 | 606522 | Microphthalmia | Mutant | 114 | GDF3R266C | 89.6967_89.4847_88.8829_88.7486_88.4983 | 89.6967 | 8.0114_8.012_8.012_8.0138_8.0141 | 8.0114 |
| GDF6 | 601147 | Microphthalmia, Leber congenital amaurosis | WT | 120 | GDF6NM_001001557WT | 89.6734_89.5001_89.419_88.9577_88.4892 | 89.6734 | 7.5514_8.1294_8.1297_8.1297_8.1296 | 7.5514 |
| GDF6 | 601147 | Microphthalmia, Leber congenital amaurosis | Mutant | 120 | GDF6NM_001001557K424R | 89.9817_89.9207_89.7279_89.389_88.5865 | 89.9817 | 7.757_8.1316_8.1316_8.1316_8.1316 | 7.757 |
| GDF6 | 601147 | Microphthalmia, Leber congenital amaurosis | Mutant | 120 | GDF6NM_001001557Y444N | 90.0449_89.9757_89.8776_89.4746_88.9722 | 90.0449 | 7.5935_8.1299_8.1297_8.1301_8.1299 | 7.5935 |
| GJA3 | 121015 | Congenital cataract and developmental cataract | WT | 434 | GJA3WT | 66.9947_63.1879_62.432_61.342_59.4852 | 66.9947 | 8.0396_8.0397_8.0398_8.0398_8.04 | 8.0396 |
| GJA3 | 121015 | Congenital cataract and developmental cataract | Mutant | 434 | GJA3N188I | 67.1405_63.5298_62.505_61.0439_59.4421 | 67.1405 | 8.81_9.346_9.346_9.3434_9.3454 | 8.81 |
| GJA3 | 121015 | Congenital cataract and developmental cataract | Mutant | 434 | GJA3R76H | 67.2394_63.2565_62.4717_60.9636_59.5667 | 67.2394 | 8.81_9.347_9.3453_9.3453_9.3468 | 8.81 |
| GJA8 | 600897 | Congenital cataract and developmental cataract, Cataract 1, Cataract | WT | 432 | GJA8WT | 63.4678_60.01_59.9991_59.7962_59.1074 | 63.4678 | 8.035_8.035_8.0351_8.0351_8.0351 | 8.035 |
| GJA8 | 600897 | Congenital cataract and developmental cataract, Cataract 1, Cataract | Mutant | 432 | GJA8D47N | 62.964_60.4386_59.9699_59.475_59.1613 | 62.964 | 8.035_8.035_8.0351_8.0349_8.035 | 8.035 |
| GJA8 | 600897 | Congenital cataract and developmental cataract, Cataract 1, Cataract | Mutant | 432 | GJA8V64G | 63.1501_60.1838_60.1499_59.994_58.7995 | 63.1501 | 8.81_8.81_8.81_9.3528_9.3536 | 8.81 |
| GJB1 | 304040 | Charcot-Marie-Tooth neuropathy, X-linked dominant, 1 | WT | 283 | GJB1WT | 78.7783_76.4599_76.3402_74.5957_73.7665 | 78.7783 | 7.9343_7.9343_7.9347_7.9345_7.9345 | 7.9343 |
| GJB1 | 304040 | Charcot-Marie-Tooth neuropathy, X-linked dominant, 1 | Mutant | 283 | GJB1R142W | 77.0067_75.2903_74.562_73.782_72.6681 | 77.0067 | 7.9346_7.9346_7.9345_7.9345_7.9345 | 7.9346 |
| GJB1 | 304040 | Charcot-Marie-Tooth neuropathy, X-linked dominant, 1 | Mutant | 283 | GJB1R215W | 77.5518_75.94_75.6658_74.4383_74.2752 | 77.5518 | 7.9345_7.9344_7.9345_7.9345_7.9346 | 7.9345 |
| GJB1 | 304040 | Charcot-Marie-Tooth neuropathy, X-linked dominant, 1 | Mutant | 283 | GJB1R22Q | 78.5279_76.7023_76.1921_74.6446_74.081 | 78.5279 | 8.1492_8.1491_8.1487_8.1498_8.1492 | 8.1492 |
| GNAT1 | 139330 | Congenital static night blindness, Night blindness, congenital stationary, autosomal dominant 3 | WT | 349 | GNAT1NM_000172WT | 94.5562_94.0437_93.6544_93.4917_93.2849 | 94.5562 | 8.0016_8.0016_8.0016_8.0016_8.0016 | 8.0016 |
| GNAT1 | 139330 | Congenital static night blindness, Night blindness, congenital stationary, autosomal dominant 3 | Mutant | 349 | GNAT1NM_000172D129G | 94.4064_93.7134_93.5709_93.4672_93.2099 | 94.4064 | 8.0016_8.0016_8.0016_8.0016_8.0016 | 8.0016 |
| GNAT1 | 139330 | Congenital static night blindness, Night blindness, congenital stationary, autosomal dominant 3 | Mutant | 349 | GNAT1NM_000172Q200E | 94.2561_93.8447_93.6573_93.0269_92.6941 | 94.2561 | 8.0016_8.0016_8.0016_8.0016_8.0016 | 8.0016 |
| GNAT2 | 139340 | Achromatopsia | WT | 353 | GNAT2WT | 94.4033_93.8197_93.7912_93.1135_92.9301 | 94.4033 | 8.0071_8.0069_8.0073_8.0073_8.0073 | 8.0071 |
| GNAT2 | 139340 | Achromatopsia | Mutant | 77 | GNAT2Q79X | 92.2841_91.6752_91.4263_89.171_89.0751 | 92.2841 | 7.9063_7.9049_7.9097_7.9127_7.9136 | 7.9063 |
| GNB3 | 139130 | Congenital static night blindness | WT | 340 | GNB3WT | 96.6613_96.6323_93.9628_93.5822_90.8981 | 96.6613 | 8.1931_8.1931_8.1932_8.1931_8.1931 | 8.1931 |
| GNB3 | 139130 | Congenital static night blindness | Mutant | 339 | GNB357missing | 96.377_96.3513_94.1068_93.077_90.4346 | 96.377 | 8.1943_8.1942_8.1944_8.1943_8.1943 | 8.1943 |
| GNB3 | 139130 | Congenital static night blindness | Mutant | 340 | GNB3S67F | 96.5754_96.5567_93.8675_92.8535_90.0548 | 96.5754 | 8.1929_8.1929_8.1929_8.1929_8.1929 | 8.1929 |
| GNPAT | 602744 | Rhizomelic chondrodysplasia punctata | WT | 680 | GNPATWT | 88.8144_88.532_88.3233_88.157_88.0651 | 88.8144 | 8.0466_8.0465_8.0466_8.0466_8.0466 | 8.0466 |
| GNPAT | 602744 | Rhizomelic chondrodysplasia punctata | Mutant | 680 | GNPATD519G | 88.587_88.5664_88.5324_88.3076_88.0844 | 88.587 | 8.0466_8.0467_8.0467_8.0467_8.0467 | 8.0466 |
| GNPAT | 602744 | Rhizomelic chondrodysplasia punctata | Mutant | 680 | GNPATR211H | 88.6652_88.2907_88.2337_87.8531_87.7036 | 88.6652 | 8.0466_8.0466_8.0466_8.0465_8.0466 | 8.0466 |
| GNPTG | 607838 | Mucolipidosis, Mucopolysaccharidosis | WT | 281 | GNPTGWT | 81.5423_80.3208_80.1793_80.0296_79.8324 | 81.5423 | 7.9345_7.9345_7.9345_7.9345_7.9345 | 7.9345 |
| GNPTG | 607838 | Mucolipidosis, Mucopolysaccharidosis | Mutant | 280 | GNPTG115MISSING | 81.5197_80.1908_80.1847_79.7625_79.5912 | 81.5197 | 9.0897_9.0765_9.0793_9.0849_9.0829 | 9.0897 |
| GNPTG | 607838 | Mucolipidosis, Mucopolysaccharidosis | Mutant | 281 | GNPTGG106S | 81.7103_80.4864_80.4631_80.0707_79.8388 | 81.7103 | 7.9346_7.9346_7.9346_7.9346_7.9346 | 7.9346 |
| GP1BA | 606672 | Nonarteritic anterior ischemic optic neuropathy, susceptibility to | WT | 636 | GP1BAWT | 63.2474_61.9818_59.2146_59.1174_59.035 | 63.2474 | 7.5601_7.9975_7.9989_8.001_8.0033 | 7.5601 |
| GP1BA | 606672 | Nonarteritic anterior ischemic optic neuropathy, susceptibility to | Mutant | 635 | GP1BA195missing | 63.4639_61.2609_58.8147_58.3171_57.4052 | 63.4639 | 7.4509_7.9931_7.9956_7.9989_7.9989 | 7.4509 |
| GP1BA | 606672 | Nonarteritic anterior ischemic optic neuropathy, susceptibility to | Mutant | 636 | GP1BAG249V | 63.6294_61.348_58.9544_58.5033_58.4265 | 63.6294 | 7.5807_8.0002_8.0037_8.0037_8.0039 | 7.5807 |
| GPR143 | 300808 | Nystagmus, Ocular albinism, Ocular albinism, type I, Nettleship-Falls type | WT | 404 | GPR143WT | 73.9935_72.6957_71.9501_71.7711_68.9098 | 73.9935 | 8.0423_8.0423_8.0423_8.0423_8.0424 | 8.0423 |
| GPR143 | 300808 | Nystagmus, Ocular albinism, Ocular albinism, type I, Nettleship-Falls type | Mutant | 404 | GPR143G118E | 74.7155_72.4001_72.215_71.2261_69.562 | 74.7155 | 9.423_9.4228_9.4226_9.422_9.4223 | 9.423 |
| GPR143 | 300808 | Nystagmus, Ocular albinism, Ocular albinism, type I, Nettleship-Falls type | Mutant | 404 | GPR143T232K | 74.4502_72.612_72.1151_72.0902_69.1014 | 74.4502 | 9.798_9.4217_9.4227_9.4215_9.4228 | 9.798 |
| GRK1 | 180381 | Oguchi disease-2, Congenital static night blindness | WT | 560 | GRK1NM_002929WT | 90.2325_90.058_88.5244_88.0735_87.2283 | 90.2325 | 7.9412_7.9411_7.9412_7.9416_7.9413 | 7.9412 |
| GRK1 | 180381 | Oguchi disease-2, Congenital static night blindness | Mutant | 560 | GRK1NM_002929P391H | 90.1414_89.7345_89.6337_88.2861_87.9588 | 90.1414 | 8.0398_8.0398_8.0398_8.0399_8.0399 | 8.0398 |
| GRK1 | 180381 | Oguchi disease-2, Congenital static night blindness | Mutant | 560 | GRK1NM_002929V380D | 90.4895_90.3068_89.0026_87.7802_87.0433 | 90.4895 | 8.0398_8.0399_8.0398_8.0399_8.04 | 8.0398 |
| GRM6 | 604096 | Congenital static night blindness, Night blindness, congenital stationary (complete), 1B, autosomal recessive | WT | 853 | GRM6NM_000843WT | 86.9753_86.9171_86.3386_86.1818_85.3305 | 86.9753 | 4.8014_8.1843_8.1843_8.1843_8.1842 | 4.8014 |
| GRM6 | 604096 | Congenital static night blindness, Night blindness, congenital stationary (complete), 1B, autosomal recessive | Mutant | 853 | GRM6NM_000843C522Y | 86.9919_86.9794_86.4945_85.718_85.5782 | 86.9919 | 4.832_8.1842_8.1843_8.1842_8.1842 | 4.832 |
| GRM6 | 604096 | Congenital static night blindness, Night blindness, congenital stationary (complete), 1B, autosomal recessive | Mutant | 853 | GRM6NM_000843G58R | 86.6257_86.6134_86.2788_86.1041_85.4261 | 86.6257 | 4.8241_8.1843_8.1842_8.1842_8.1843 | 4.8241 |
| GRN | 138945 | Ceroid lipofuscinosis, neuronal | WT | 576 | GRNWT | 76.214_75.3851_74.4806_72.4528_72.1607 | 76.214 | 7.9076_7.956_7.9595_7.9623_7.963 | 7.9076 |
| GRN | 138945 | Ceroid lipofuscinosis, neuronal | Mutant | 263 | GRNp_T272Sfs_10 | 76.7658_75.8067_75.0517_74.648_74.0711 | 76.7658 | 7.9707_7.9707_7.9784_7.9772_7.9799 | 7.9707 |
| GUCA1A | 600364 | Cone-rod retinal dystrophy | WT | 200 | GUCA1AWT | 72.0765_71.8591_69.8544_67.2796_66.0262 | 72.0765 | 7.0286_7.8901_7.8917_7.8911_7.893 | 7.0286 |
| GUCA1A | 600364 | Cone-rod retinal dystrophy | Mutant | 200 | GUCA1AL151F | 73.1133_72.4266_71.3664_68.1209_67.1932 | 73.1133 | 7.0064_7.8889_7.8912_7.8916_7.8917 | 7.0064 |
| GUCA1A | 600364 | Cone-rod retinal dystrophy | Mutant | 200 | GUCA1AY99C | 75.6781_74.8245_72.9368_68.8328_67.7194 | 75.6781 | 6.9614_7.8892_7.8888_7.89_7.8908 | 6.9614 |
| GUCA1B | 602275 | Retinitis pigmentosa | WT | 199 | GUCA1BWT | 75.0734_70.283_69.7916_68.3565_67.7465 | 75.0734 | 7.4358_7.443_7.4481_7.445_7.4475 | 7.4358 |
| GUCA1B | 602275 | Retinitis pigmentosa | Mutant | 199 | GUCA1BR44C | 72.6602_69.6476_68.897_67.6136_67.0582 | 72.6602 | 7.7929_7.883_7.8847_7.8839_7.8847 | 7.7929 |
| GUCY2D | 600179 | Cone-rod dystrophy, Leber congenital amaurosis, Leber congenital amaurosis 1, Cone-rod retinal dystrophy | WT | 1052 | GUCY2DNM_000180WT | 83.7949_83.7645_83.2682_82.9004_82.7464 | 83.7949 | 2.9332_7.9786_7.9807_7.9823_7.9829 | 2.9332 |
| GUCY2D | 600179 | Cone-rod dystrophy, Leber congenital amaurosis, Leber congenital amaurosis 1, Cone-rod retinal dystrophy | Mutant | 1052 | GUCY2DNM_000180R768W | 83.598_83.3166_83.1048_82.5512_82.1932 | 83.598 | 2.9572_7.9772_7.9791_7.9827_7.9845 | 2.9572 |
| GUCY2D | 600179 | Cone-rod dystrophy, Leber congenital amaurosis, Leber congenital amaurosis 1, Cone-rod retinal dystrophy | Mutant | 1052 | GUCY2DNM_000180R838C | 83.6014_83.3375_83.0755_83.0108_82.3848 | 83.6014 | 3.0327_7.9798_7.9834_7.9853_7.9861 | 3.0327 |
| GUSB | 611499 | Mucolipidosis, Mucopolysaccharidosis | WT | 629 | GUSBWT | 95.2396_95.1933_95.0188_94.9908_94.9401 | 95.2396 | 5.8548_7.9399_7.9401_7.9404_7.9408 | 5.8548 |
| GUSB | 611499 | Mucolipidosis, Mucopolysaccharidosis | Mutant | 629 | GUSBL176F | 95.9929_95.991_95.0097_95.0001_94.9298 | 95.9929 | 6.5405_8.0343_8.0344_8.0344_8.0344 | 6.5405 |
| GUSB | 611499 | Mucolipidosis, Mucopolysaccharidosis | Mutant | 629 | GUSBW627C | 96.1796_96.1352_95.0608_94.9981_94.9759 | 96.1796 | 6.4683_7.7137_7.9779_7.9808_7.9822 | 6.4683 |
| HARS | 142810 | Usher syndrome | WT | 508 | HARSWT | 92.1194_91.872_91.5274_91.0769_90.8245 | 92.1194 | 8.2891_8.2892_8.2893_8.2891_8.2892 | 8.2891 |
| HARS | 142810 | Usher syndrome | Mutant | 508 | HARSY454S | 92.1534_91.7191_91.4412_90.6852_90.5545 | 92.1534 | 9.2328_9.233_9.2333_9.2336_9.2339 | 9.2328 |
| HCCS | 300056 | Microphthalmia | WT | 267 | HCCSWT | 77.6367_74.4093_74.3837_73.9315_73.6097 | 77.6367 | 7.8871_7.9026_7.9032_7.9051_7.905 | 7.8871 |
| HCCS | 300056 | Microphthalmia | Mutant | 195 | HCCS197-268MISSING | 73.448_71.9047_71.1928_71.1222_70.6889 | 73.448 | 7.7679_7.8263_7.8533_8.1259_8.1269 | 7.7679 |
| HCCS | 300056 | Microphthalmia | Mutant | 267 | HCCSR217C | 77.7282_75.0029_74.9617_74.4401_73.4383 | 77.7282 | 7.9057_7.9086_7.9157_7.9196_7.9198 | 7.9057 |
| HESX1 | 601802 | Septooptic dysplasia | WT | 185 | HESX1WT | 71.3985_70.2046_68.8644_68.2903_67.9586 | 71.3985 | 6.0107_8.0345_8.0407_8.0394_8.06 | 6.0107 |
| HESX1 | 601802 | Septooptic dysplasia | Mutant | 185 | HESX1R109Q | 70.9284_68.787_68.1633_68.007_67.7985 | 70.9284 | 5.8958_8.248_8.2577_8.2398_8.2596 | 5.8958 |
| HEXA | 606869 | Tay-Sachs disease | WT | 441 | HEXAWT | 97.2579_97.0565_95.5376_95.4571_95.2106 | 97.2579 | 9.3469_9.3475_9.3475_9.3472_9.3477 | 9.3469 |
| HEXA | 606869 | Tay-Sachs disease | Mutant | 441 | HEXAE482K | 97.2934_97.0478_95.7657_95.4307_95.1261 | 97.2934 | 8.0342_8.0342_8.0343_8.0342_8.0342 | 8.0342 |
| HEXA | 606869 | Tay-Sachs disease | Mutant | 441 | HEXAR170W | 97.2585_97.1156_95.5601_95.5014_94.6911 | 97.2585 | 8.0341_8.0341_8.0341_8.0343_8.0343 | 8.0341 |
| HEXB | 606873 | Sandhoff disease, infantile, juvenile, and adult forms | WT | 435 | HEXBWT | 97.5236_97.3801_95.9836_95.8873_95.7958 | 97.5236 | 8.037_8.0372_8.037_8.0371_8.0371 | 8.037 |
| HEXB | 606873 | Sandhoff disease, infantile, juvenile, and adult forms | Mutant | 435 | HEXBP417L | 97.6799_97.589_95.6843_95.642_95.5075 | 97.6799 | 8.0369_8.037_8.0371_8.037_8.0371 | 8.0369 |
| HEXB | 606873 | Sandhoff disease, infantile, juvenile, and adult forms | Mutant | 435 | HEXBR505Q | 97.8081_97.7059_95.8637_95.7291_95.6724 | 97.8081 | 8.0374_8.0374_8.0373_8.0375_8.0374 | 8.0374 |
| HGSNAT | 610453 | Mucolipidosis, Mucopolysaccharidosis, Retinitis pigmentosa | WT | 664 | HGSNATWT | 77.1569_75.4972_75.3259_75.2906_74.9205 | 77.1569 | 7.9987_8.0005_8.0015_8.0021_8.0027 | 7.9987 |
| HGSNAT | 610453 | Mucolipidosis, Mucopolysaccharidosis, Retinitis pigmentosa | Mutant | 664 | HGSNATA644T | 77.2556_76.0643_75.4575_75.3906_75.1307 | 77.2556 | 8.0441_8.0442_8.0443_8.0441_8.0443 | 8.0441 |
| HGSNAT | 610453 | Mucolipidosis, Mucopolysaccharidosis, Retinitis pigmentosa | Mutant | 664 | HGSNATR124W | 77.2003_75.697_75.398_75.3634_75.0334 | 77.2003 | 8.0441_8.044_8.0437_8.044_8.0443 | 8.0441 |
| HK1 | 142600 | Retinitis pigmentosa 79 | WT | 917 | HK1WT | 94.4995_94.239_92.0933_90.7556_90.2445 | 94.4995 | 5.5432_8.0386_8.0388_8.0388_8.0386 | 5.5432 |
| HK1 | 142600 | Retinitis pigmentosa 79 | Mutant | 917 | HK1E847K | 94.601_94.1942_92.2355_91.0748_90.4079 | 94.601 | 6.4545_7.9836_7.9836_7.9836_7.9837 | 6.4545 |
| HK1 | 142600 | Retinitis pigmentosa 79 | Mutant | 917 | HK1K418E | 94.5885_94.2321_92.1418_91.2517_90.753 | 94.5885 | 5.5565_8.039_8.039_8.039_8.039 | 5.5565 |
| HMX1 | 142992 | Oculoauricular syndrome, Kahrizi syndrome, Microcephaly with or without chorioretinopathy, lymphedema, or mental retardation | WT | 348 | HMX1WT | 62.7561_58.0991_57.9996_57.596_56.2969 | 62.7561 | 6.1624_8.0078_8.0078_8.0079_8.0079 | 6.1624 |
| HMX1 | 142992 | Oculoauricular syndrome, Kahrizi syndrome, Microcephaly with or without chorioretinopathy, lymphedema, or mental retardation | Mutant | 348 | HMX1Q217P | 61.5218_58.6207_57.5698_56.945_56.7665 | 61.5218 | 6.2696_8.0079_8.0079_8.0079_8.0079 | 6.2696 |
| HPS5 | 607521 | Hermansky-Pudlak syndrome | WT | 1129 | HPS5WT | 71.1666_71.1048_70.7054_70.4006_69.5441 | 71.1666 | 7.6819_7.93_7.93_7.93_7.93 | 7.6819 |
| HPS5 | 607521 | Hermansky-Pudlak syndrome | Mutant | 1129 | HPS5L624R | 71.343_70.9917_70.5906_70.4929_69.6806 | 71.343 | 4.5734_4.6071_4.6508_4.7583_4.9094 | 4.5734 |
| HPS5 | 607521 | Hermansky-Pudlak syndrome | Mutant | 1129 | HPS5T1098I | 70.8664_70.7235_70.6126_70.5891_70.1383 | 70.8664 | 7.6807_7.93_7.93_7.93_7.93 | 7.6807 |
| HSF4 | 602438 | Congenital cataract and developmental cataract | WT | 462 | HSF4WT | 59.7079_57.6564_57.042_56.5368_56.2714 | 59.7079 | 8.0308_8.0309_8.0309_8.0308_8.0309 | 8.0308 |
| HSF4 | 602438 | Congenital cataract and developmental cataract | Mutant | 462 | HSF4R119C | 59.4628_57.7012_56.55_55.6223_55.0681 | 59.4628 | 8.0308_8.0309_8.0309_8.031_8.0308 | 8.0308 |
| HSF4 | 602438 | Congenital cataract and developmental cataract | Mutant | 462 | HSF4R73H | 59.6926_57.927_56.7941_55.6537_55.4304 | 59.6926 | 8.0309_8.0308_8.0309_8.0309_8.031 | 8.0309 |
| HTRA1 | 602194 | Macular degeneration, age-related | WT | 458 | HTRA1WT | 84.9032_84.7502_84.6375_84.5154_84.0123 | 84.9032 | 8.0302_8.0302_8.0302_8.0302_8.0302 | 8.0302 |
| HTRA1 | 602194 | Macular degeneration, age-related | Mutant | 458 | HTRA1A252T | 84.8244_84.474_84.241_84.0835_83.7877 | 84.8244 | 8.8159_9.2743_9.2749_9.2748_9.2751 | 8.8159 |
| HTRA1 | 602194 | Macular degeneration, age-related | Mutant | 458 | HTRA1V297M | 84.7778_84.7695_84.3933_84.2866_83.4751 | 84.7778 | 8.6551_9.3393_9.3404_9.3398_9.3397 | 8.6551 |
| IDH3B | 604526 | Retinitis pigmentosa | WT | 351 | IDH3BWT | 92.9793_92.1565_91.8574_91.7778_91.3846 | 92.9793 | 8.0012_8.0013_8.0013_8.0012_8.0013 | 8.0012 |
| IDH3B | 604526 | Retinitis pigmentosa | Mutant | 351 | IDH3BL132P | 92.8521_92.3551_92.105_91.7653_90.8462 | 92.8521 | 8.0013_8.0013_8.0013_8.0013_8.0013 | 8.0013 |
| IDUA | 252800 | Mucolipidosis, Mucopolysaccharidosis | WT | 626 | IDUAWT | 96.5113_96.3753_95.3593_94.9266_94.591 | 96.5113 | 8.0384_8.0384_8.0383_8.0384_8.0384 | 8.0384 |
| IDUA | 252800 | Mucolipidosis, Mucopolysaccharidosis | Mutant | 626 | IDUAA327P | 96.603_96.4532_95.1509_94.932_94.8604 | 96.603 | 8.0384_8.0384_8.0383_8.0384_8.0386 | 8.0384 |
| IDUA | 252800 | Mucolipidosis, Mucopolysaccharidosis | Mutant | 626 | IDUAP533R | 96.5475_96.5134_94.8973_94.4027_94.3559 | 96.5475 | 7.9393_7.9398_7.9396_7.9397_7.9399 | 7.9393 |
| IFNGR1 | 107470 | Cataract | WT | 472 | IFNGR1WT | 67.1643_64.548_64.2694_64.1894_64.0349 | 67.1643 | 7.5476_8.0438_8.0435_8.0437_8.0436 | 7.5476 |
| IFNGR1 | 107470 | Cataract | Mutant | 472 | IFNGR1C77Y | 67.1029_64.5571_64.5515_64.092_64.0623 | 67.1029 | 8.2891_9.2603_9.2629_9.2631_9.2637 | 8.2891 |
| IFNGR1 | 107470 | Cataract | Mutant | 472 | IFNGR1I87T | 67.3011_64.6167_64.1267_63.9962_63.9673 | 67.3011 | 7.5639_8.0437_8.0437_8.0439_8.0439 | 7.5639 |
| IFT140 | 614620 | jeune syndrome, Retinitis pigmentosa 80 | WT | 1462 | IFT140WT | 79.838_78.1385_77.698_77.3155_76.2479 | 79.838 | 7.9663_7.9687_7.9699_7.971_7.9755 | 7.9663 |
| IFT140 | 614620 | jeune syndrome, Retinitis pigmentosa 80 | Mutant | 1462 | IFT140E664K | 79.518_78.0034_77.8615_77.6292_76.7104 | 79.518 | 7.9742_7.9743_7.9772_7.9801_7.9805 | 7.9742 |
| IFT140 | 614620 | jeune syndrome, Retinitis pigmentosa 80 | Mutant | 1462 | IFT140G212R | 79.5497_78.0177_77.8104_77.1853_76.4067 | 79.5497 | 7.9734_7.9753_7.9792_7.98_7.9805 | 7.9734 |
| IFT172 | 607386 | jeune syndrome, Retinitis pigmentosa | WT | 1749 | IFT172WT | 84.419_83.0339_81.3996_80.883_80.1762 | 84.419 | 8.0816_8.0898_8.0912_8.0911_8.0964 | 8.0816 |
| IFT172 | 607386 | jeune syndrome, Retinitis pigmentosa | Mutant | 1749 | IFT172D1605E | 84.5357_82.6171_81.191_80.5529_80.5032 | 84.5357 | 8.0869_8.0933_8.0935_8.0945_8.0943 | 8.0869 |
| IFT172 | 607386 | jeune syndrome, Retinitis pigmentosa | Mutant | 1749 | IFT172H1567Q | 84.6276_82.8802_82.095_81.0818_80.4716 | 84.6276 | 8.0862_8.0901_8.0913_8.0969_8.0979 | 8.0862 |
| IFT172 | 607386 | jeune syndrome, Retinitis pigmentosa | Mutant | 1749 | IFT172L257P | 84.6634_83.253_81.7654_81.2163_80.2551 | 84.6634 | 8.0848_8.0902_8.0908_8.092_8.0941 | 8.0848 |
| IFT43 | 614068 | Retinitis pigmentosa 81 | WT | 208 | IFT43WT | 63.3471_62.4889_62.4229_61.6598_61.3414 | 63.3471 | 7.9008_7.9013_7.9043_7.9026_7.904 | 7.9008 |
| IFT43 | 614068 | Retinitis pigmentosa 81 | Mutant | 208 | IFT43E34K | 63.0265_62.2133_61.8511_61.0233_60.9587 | 63.0265 | 7.9018_7.9037_7.9048_7.9059_7.907 | 7.9018 |
| IMPDH1 | 146690 | Retinitis pigmentosa, Leber congenital amaurosis, Leber congenital amaurosis 11 | WT | 562 | IMPDH1NM_183243WT | 89.6208_87.7572_86.7993_86.6363_86.5617 | 89.6208 | 8.0316_8.0317_8.0318_8.0318_8.0318 | 8.0316 |
| IMPDH1 | 146690 | Retinitis pigmentosa, Leber congenital amaurosis, Leber congenital amaurosis 11 | Mutant | 598 | IMPDH1NM_183243D214Y | 86.0001_84.7573_83.3641_82.9704_82.8436 | 86.0001 | 8.0333_8.0332_8.0332_8.0334_8.0333 | 8.0333 |
| IMPDH1 | 146690 | Retinitis pigmentosa, Leber congenital amaurosis, Leber congenital amaurosis 11 | Mutant | 598 | IMPDH1NM_183243K293E | 85.3682_83.1908_82.6966_82.6926_81.9844 | 85.3682 | 8.0332_8.0333_8.0334_8.0333_8.0333 | 8.0332 |
| IMPDH1 | 146690 | Retinitis pigmentosa, Leber congenital amaurosis, Leber congenital amaurosis 11 | Mutant | 562 | IMPDH1NM_183243V268I | 89.3369_88.5633_85.9492_85.5765_85.2979 | 89.3369 | 8.0318_8.0317_8.0316_8.0317_8.0318 | 8.0318 |
| IMPG2 | 607056 | Retinitis pigmentosa, Macular dystrophy, vitelliform | WT | 1219 | IMPG2WT | 54.2602_51.78_51.5303_51.3557_51.2602 | 54.2602 | 8.13_8.1302_8.1303_8.1302_8.1308 | 8.13 |
| IMPG2 | 607056 | Retinitis pigmentosa, Macular dystrophy, vitelliform | Mutant | 1219 | IMPG2C1077F | 54.2202_51.9543_51.4456_51.223_50.9515 | 54.2202 | 8.13_8.13_8.1303_8.1302_8.1304 | 8.13 |
| IMPG2 | 607056 | Retinitis pigmentosa, Macular dystrophy, vitelliform | Mutant | 1219 | IMPG2F124L | 54.3913_51.6319_51.57_51.4221_51.1915 | 54.3913 | 8.1298_8.13_8.1303_8.1304_8.1304 | 8.1298 |
| INPP5E | 613037 | Joubert syndrome | WT | 641 | INPP5EWT | 72.6061_70.4313_69.5503_69.0049_67.9551 | 72.6061 | 8.0383_8.0382_8.0383_8.0383_8.0383 | 8.0383 |
| INPP5E | 613037 | Joubert syndrome | Mutant | 641 | INPP5ER435Q | 71.458_70.5277_69.0726_69.0578_68.9523 | 71.458 | 8.0383_8.0382_8.0384_8.0385_8.0384 | 8.0383 |
| INPP5E | 613037 | Joubert syndrome | Mutant | 641 | INPP5ER563H | 71.6744_69.9476_69.4797_68.6553_68.1816 | 71.6744 | 7.9511_7.9555_7.9566_7.9624_7.9648 | 7.9511 |
| INVS | 243305 | Nephronophthisis | WT | 1065 | INVSWT | 67.8106_67.4445_67.3671_66.7862_65.5049 | 67.8106 | 3.6348_8.2537_8.2633_8.2702_8.2709 | 3.6348 |
| INVS | 243305 | Nephronophthisis | Mutant | 1065 | INVSL493S | 68.8407_68.0458_67.6713_66.4408_66.1497 | 68.8407 | 3.8597_8.2544_8.2581_8.2601_8.2608 | 3.8597 |
| INVS | 243305 | Nephronophthisis | Mutant | 1065 | INVSP482R | 68.4039_68.3076_67.7668_66.5916_66.1144 | 68.4039 | 3.8244_8.2616_8.2662_8.2689_8.2712 | 3.8244 |
| IQCB1 | 609237 | Senior-Loken syndrome | WT | 598 | IQCB1WT | 83.7118_82.7886_82.0198_81.7391_80.9818 | 83.7118 | 8.0329_8.0329_8.0329_8.033_8.0329 | 8.0329 |
| IQCB1 | 609237 | Senior-Loken syndrome | Mutant | 598 | IQCB1A549K | 82.6007_82.2931_81.8777_81.5622_81.5443 | 82.6007 | 7.9568_7.9593_7.9597_7.9596_7.9603 | 7.9568 |
| IRX5 | 606195 | Microphthalmia, syndromic, Hamamy syndrome | WT | 483 | IRX5NM_005853WT | 53.2353_48.173_47.5703_47.383_47.0796 | 53.2353 | 8.0337_8.0335_8.0336_8.0337_8.0335 | 8.0337 |
| IRX5 | 606195 | Microphthalmia, syndromic, Hamamy syndrome | Mutant | 483 | IRX5NM_005853A150P | 53.7856_48.6631_48.1201_47.5139_47.5077 | 53.7856 | 8.0334_8.0336_8.0336_8.0337_8.0337 | 8.0334 |
| IRX5 | 606195 | Microphthalmia, syndromic, Hamamy syndrome | Mutant | 483 | IRX5NM_005853N166K | 53.5317_48.3964_47.9694_47.7461_47.1674 | 53.5317 | 8.0337_8.0337_8.0337_8.0338_8.0337 | 8.0337 |
| ITGA2B | 607759 | Retina | WT | 1008 | ITGA2BWT | 90.1147_89.549_86.7693_86.3931_86.0651 | 90.1147 | 8.0514_8.0514_8.0514_8.0514_8.0514 | 8.0514 |
| ITGA2B | 607759 | Retina | Mutant | 1008 | ITGA2BI405T | 90.0201_89.4119_86.9628_86.2253_86.0586 | 90.0201 | 8.0514_8.0515_8.0514_8.0515_8.0514 | 8.0514 |
| ITGA2B | 607759 | Retina | Mutant | 1008 | ITGA2BQ778P | 89.7092_88.9961_87.0849_86.3741_85.6849 | 89.7092 | 8.0514_8.0514_8.0514_8.0514_8.0514 | 8.0514 |
| ITGB3 | 173470 | Retina | WT | 762 | ITGB3WT | 88.6959_88.0504_85.0072_84.6095_83.5701 | 88.6959 | 7.1797_8.1831_8.1845_8.1845_8.1845 | 7.1797 |
| ITGB3 | 173470 | Retina | Mutant | 762 | ITGB3D749H | 88.3532_87.5662_84.4097_84.3567_82.5482 | 88.3532 | 7.1077_8.1845_8.1845_8.1845_8.1845 | 7.1077 |
| ITGB3 | 173470 | Retina | Mutant | 762 | ITGB3L222P | 88.3886_87.6433_84.5429_84.4655_83.2986 | 88.3886 | 6.9394_8.1828_8.1845_8.1845_8.1845 | 6.9394 |
| ITM2B | 603904 | Retinal dystrophy, Retinitis pigmentosa, Lowe syndrome | WT | 266 | ITM2BWT | 76.2431_75.9636_75.4774_75.4563_75.0497 | 76.2431 | 7.9824_7.9817_7.9843_7.9842_7.9867 | 7.9824 |
| ITM2B | 603904 | Retinal dystrophy, Retinitis pigmentosa, Lowe syndrome | Mutant | 277 | ITM2B266S-FNLFLNSQEKHY | 74.3176_73.6976_73.6773_73.2364_72.482 | 74.3176 | 7.9385_7.9387_7.9383_7.9384_7.9386 | 7.9385 |
| ITM2B | 603904 | Retinal dystrophy, Retinitis pigmentosa, Lowe syndrome | Mutant | 277 | ITM2B266S-SRTVKKNIIEEN | 74.7257_74.7055_74.103_74.0293_73.1558 | 74.7257 | 7.9385_7.9386_7.9384_7.9384_7.9386 | 7.9385 |
| JAG1 | 601920 | Alagille syndrome | WT | 1185 | JAG1WT | 75.063_73.3771_73.1329_71.7935_70.4384 | 75.063 | 8.0807_8.0804_8.0807_8.0808_8.0808 | 8.0807 |
| JAG1 | 601920 | Alagille syndrome | Mutant | 1185 | JAG1G274D | 74.5525_73.4441_73.0761_71.325_69.8679 | 74.5525 | 8.0257_8.0259_8.026_8.0261_8.0262 | 8.0257 |
| JAG1 | 601920 | Alagille syndrome | Mutant | 1185 | JAG1L37S | 75.6793_73.3503_72.471_71.5032_69.1075 | 75.6793 | 8.0805_8.0805_8.0806_8.0807_8.0807 | 8.0805 |
| KCNV2 | 607604 | Retinal cone dystrophy, Cone-rod dystrophy, Retinal cone dystrophy 3B | WT | 545 | KCNV2WT | 76.1952_75.5549_75.1069_75.0489_74.4346 | 76.1952 | 8.0327_8.0329_8.0328_8.0329_8.0328 | 8.0327 |
| KCNV2 | 607604 | Retinal cone dystrophy, Cone-rod dystrophy, Retinal cone dystrophy 3B | Mutant | 545 | KCNV2A331T | 76.151_75.9947_75.2582_75.1089_74.8536 | 76.151 | 8.0328_8.0329_8.0328_8.0328_8.0328 | 8.0328 |
| KCNV2 | 607604 | Retinal cone dystrophy, Cone-rod dystrophy, Retinal cone dystrophy 3B | Mutant | 545 | KCNV2A505S | 75.9012_75.6612_75.0085_74.9351_74.616 | 75.9012 | 8.0328_8.0328_8.0329_8.0329_8.0329 | 8.0328 |
| KCTD7 | 611725 | Epilepsy, progressive myoclonic 3, with or without intracellular inclusions | WT | 289 | KCTD7WT | 81.1202_80.7356_80.4752_80.3471_79.9733 | 81.1202 | 8.3024_8.305_8.3052_8.3058_8.3137 | 8.3024 |
| KCTD7 | 611725 | Epilepsy, progressive myoclonic 3, with or without intracellular inclusions | Mutant | 289 | KCTD7L108M | 81.311_80.3314_80.0799_80.009_79.0943 | 81.311 | 8.2964_8.3019_8.3056_8.3043_8.3075 | 8.2964 |
| KCTD7 | 611725 | Epilepsy, progressive myoclonic 3, with or without intracellular inclusions | Mutant | 289 | KCTD7R94W | 81.7767_80.8869_80.4991_80.1014_79.1626 | 81.7767 | 8.04_8.0404_8.0405_8.0406_8.0412 | 8.04 |
| KERA | 603288 | Corneal abnormalities | WT | 332 | KERAWT | 89.3662_88.9332_88.638_88.4828_88.4312 | 89.3662 | 8.1865_8.1865_8.1865_8.1866_8.1865 | 8.1865 |
| KERA | 603288 | Corneal abnormalities | Mutant | 332 | KERAN247S | 90.6727_89.0846_88.9233_88.7466_88.3011 | 90.6727 | 8.1864_8.1864_8.1866_8.1866_8.1865 | 8.1864 |
| KERA | 603288 | Corneal abnormalities | Mutant | 332 | KERAT215K | 88.5017_88.3494_88.2273_88.1936_87.7756 | 88.5017 | 8.1865_8.1864_8.1866_8.1865_8.1866 | 8.1865 |
| KIF11 | 148760 | Familial exudative vitreoretinopathy, Optic disc anomalies with retinal and/or macular dystrophy, Microcephaly with or without chorioretinopathy, lymphedema, or mental retardation | WT | 1056 | KIF11NM_004523WT | 75.0109_73.7047_72.8321_72.4939_65.8701 | 75.0109 | 2.8634_8.0088_8.0131_8.0201_8.0211 | 2.8634 |
| KIF11 | 148760 | Familial exudative vitreoretinopathy, Optic disc anomalies with retinal and/or macular dystrophy, Microcephaly with or without chorioretinopathy, lymphedema, or mental retardation | Mutant | 1056 | KIF11NM_004523F144L | 74.4511_74.42_73.3659_71.479_70.0367 | 74.4511 | 3.0066_8.0335_8.0355_8.0385_8.0398 | 3.0066 |
| KIF11 | 148760 | Familial exudative vitreoretinopathy, Optic disc anomalies with retinal and/or macular dystrophy, Microcephaly with or without chorioretinopathy, lymphedema, or mental retardation | Mutant | 1056 | KIF11NM_004523S235C | 74.5262_74.3984_74.1342_74.1101_69.6285 | 74.5262 | 2.747_8.035_8.0358_8.0384_8.0389 | 2.747 |
| KIF21A | 608283 | Congenital fibrosis of the extraocular muscles, Congenital fibrosis of the extraocular muscles, Fibrosis of extraocular muscles, congenital, 1, Fibrosis of extraocular muscles, congenital, 3B, Duane retraction syndrome | WT | 1661 | KIF21AWT | 68.5027_68.3647_67.97_67.2248_67.0383 | 68.5027 | 7.9502_7.9502_7.9502_7.9502_7.9502 | 7.9502 |
| KIF21A | 608283 | Congenital fibrosis of the extraocular muscles, Congenital fibrosis of the extraocular muscles, Fibrosis of extraocular muscles, congenital, 1, Fibrosis of extraocular muscles, congenital, 3B, Duane retraction syndrome | Mutant | 1661 | KIF21AM356T | 68.98_67.6864_66.9519_66.8732_66.8172 | 68.98 | 8.0244_8.0245_8.0245_8.0245_8.0245 | 8.0244 |
| KIF21A | 608283 | Congenital fibrosis of the extraocular muscles, Congenital fibrosis of the extraocular muscles, Fibrosis of extraocular muscles, congenital, 1, Fibrosis of extraocular muscles, congenital, 3B, Duane retraction syndrome | Mutant | 1661 | KIF21AR954Q | 68.612_68.0985_67.4248_67.0159_66.8125 | 68.612 | 7.9502_7.9502_7.9502_7.9502_7.9502 | 7.9502 |
| KIF21A | 608283 | Congenital fibrosis of the extraocular muscles, Congenital fibrosis of the extraocular muscles, Fibrosis of extraocular muscles, congenital, 1, Fibrosis of extraocular muscles, congenital, 3B, Duane retraction syndrome | Mutant | 1661 | KIF21AR954W | 68.9635_68.7136_68.2684_67.5582_67.5311 | 68.9635 | 7.9502_7.9502_7.9502_7.9502_7.9502 | 7.9502 |
| KIF7 | 611254 | Joubert syndrome | WT | 1343 | KIF7WT | 66.1296_66.0071_65.9222_64.1225_63.3652 | 66.1296 | 7.9264_7.93_7.93_7.93_7.93 | 7.9264 |
| KIF7 | 611254 | Joubert syndrome | Mutant | 1339 | KIF71329-1332MISSING | 67.152_66.6997_66.2011_65.3885_64.7193 | 67.152 | 7.9251_7.93_7.93_7.93_7.93 | 7.9251 |
| KIF7 | 611254 | Joubert syndrome | Mutant | 1343 | KIF7R641G | 66.3283_66.3171_66.2696_63.8972_63.8821 | 66.3283 | 7.9262_7.93_7.93_7.93_7.93 | 7.9262 |
| KLHL7 | 611119 | Retinitis pigmentosa | WT | 586 | KLHL7WT | 91.031_90.8985_90.6444_89.2505_89.0979 | 91.031 | 5.6619_8.0339_8.0338_8.0339_8.0338 | 5.6619 |
| KLHL7 | 611119 | Retinitis pigmentosa | Mutant | 586 | KLHL7A153T | 90.734_90.3998_90.3196_87.9237_87.6246 | 90.734 | 5.6882_8.0339_8.0339_8.0339_8.0339 | 5.6882 |
| KLHL7 | 611119 | Retinitis pigmentosa | Mutant | 586 | KLHL7A153V | 90.1926_89.8386_89.8178_86.9655_86.43 | 90.1926 | 5.6916_7.9399_7.9398_7.9399_7.9404 | 5.6916 |
| KRT3 | 148043 | Corneal dystrophy | WT | 628 | KRT3WT | 63.907_63.1103_61.7469_61.7302_61.5324 | 63.907 | 7.0045_8.0448_8.0449_8.0449_8.045 | 7.0045 |
| KRT3 | 148043 | Corneal dystrophy | Mutant | 628 | KRT3E509K | 63.2132_62.9116_62.2119_62.2028_61.707 | 63.2132 | 7.1633_8.0447_8.0449_8.0449_8.045 | 7.1633 |
| KRT3 | 148043 | Corneal dystrophy | Mutant | 628 | KRT3R503P | 63.2173_62.9428_61.927_61.9099_61.6069 | 63.2173 | 7.1757_8.0448_8.0449_8.0449_8.0448 | 7.1757 |
| LAMA1 | 150320 | Poretti - Boltshauser syndrome, Poretti-Boltshauser syndrome | Mutant | 167 | LAMA1Y185X | 90.8804_90.7844_90.6386_90.4112_90.0961 | 90.8804 | 7.7867_8.1473_8.1535_8.1441_8.1547 | 7.7867 |
| LCA5 | 611408 | Leber congenital amaurosis, Leber congenital amaurosis 5 | WT | 697 | LCA5NM_181714WT | 62.6658_60.6568_59.9003_59.7904_58.8964 | 62.6658 | 7.7358_7.9254_7.9294_7.9346_7.9356 | 7.7358 |
| LCA5 | 611408 | Leber congenital amaurosis, Leber congenital amaurosis 5 | Mutant | 697 | LCA5NM_181714R218G | 63.3261_60.4425_60.0099_59.4522_59.2453 | 63.3261 | 8.033_8.0331_8.033_8.0331_8.0331 | 8.033 |
| LCT | 603202 | Cataract | WT | 1059 | LCTWT | 94.2601_93.8689_93.7962_93.7471_93.608 | 94.2601 | 3.0881_8.2258_8.2259_8.2261_8.226 | 3.0881 |
| LCT | 603202 | Cataract | Mutant | 1059 | LCTG1363S | 94.026_93.6791_93.6514_93.556_93.388 | 94.026 | 3.1103_8.2247_8.225_8.2249_8.226 | 3.1103 |
| LEMD2 | 616312 | Cataract 46, juvenile-onset | WT | 502 | LEMD2WT | 70.3026_69.8866_68.4119_66.8368_66.0698 | 70.3026 | 9.3238_9.3244_9.324_9.3254_9.328 | 9.3238 |
| LEMD2 | 616312 | Cataract 46, juvenile-onset | Mutant | 502 | LEMD2L13R | 69.4943_68.2856_67.4642_66.0787_65.1071 | 69.4943 | 8.2372_8.2372_8.2372_8.2372_8.2372 | 8.2372 |
| LEPREL1 | 610341 | Myopia, high, with cataract and vitreoretinal degeneration | WT | 684 | LEPREL1NM_018192WT | 83.1743_82.625_82.2849_82.188_81.4247 | 83.1743 |  |  |
| LEPREL1 | 610341 | Myopia, high, with cataract and vitreoretinal degeneration | Mutant | 684 | LEPREL1NM_018192G508V | 83.5073_82.896_82.8284_82.541_82.4797 | 83.5073 | 8.0426_8.0427_8.0428_8.0426_8.0428 | 8.0426 |
| LEPREL1 | 610341 | Myopia, high, with cataract and vitreoretinal degeneration | Mutant | 684 | LEPREL1NM_018192K661R | 83.7403_83.2464_82.9718_82.5537_81.9463 | 83.7403 |  |  |
| LIM2 | 154045 | Cataract, Congenital cataract and developmental cataract | WT | 173 | LIM2WT | 90.6267_89.1124_88.1695_87.7999_87.6315 | 90.6267 | 7.7857_7.7709_7.7844_7.7739_7.7937 | 7.7857 |
| LIM2 | 154045 | Cataract, Congenital cataract and developmental cataract | Mutant | 173 | LIM2F105V | 90.3525_89.7611_88.1529_88.1033_87.9304 | 90.3525 | 8.1539_8.1645_8.1637_8.1664_8.1683 | 8.1539 |
| LMNA | 150330 | Hutchinson-Gilford progeria | WT | 646 | LMNAWT | 76.5844_76.4994_76.4913_74.8157_74.4065 | 76.5844 | nan_nan_nan_nan_nan | nan |
| LMNA | 150330 | Hutchinson-Gilford progeria | Mutant | 646 | LMNAR249Q | 77.7474_77.6027_77.5921_74.5124_74.0675 | 77.7474 | 7.1804_8.0364_8.0365_8.0364_8.0364 | 7.1804 |
| LMNA | 150330 | Hutchinson-Gilford progeria | Mutant | 646 | LMNAR527P | 77.6875_77.5309_77.3645_74.3881_73.8444 | 77.6875 | nan_nan_nan_nan_nan | nan |
| LOXHD1 | 613072 | Fuchs corneal endothelial dystrophy, Fuchs endothelial corneal dystrophy | Mutant | 669 | LOXHD1R670X | 92.4478_91.289_91.1096_90.8896_90.8637 | 92.4478 | 8.0343_8.0342_8.0344_8.0343_8.0343 | 8.0343 |
| LOXL1 | 153456 | Exfoliative glaucoma | WT | 480 | LOXL1WT | 62.9902_61.3821_61.3615_61.1229_60.3686 | 62.9902 | 7.3842_8.0381_8.0381_8.0381_8.0381 | 7.3842 |
| LOXL1 | 153456 | Exfoliative glaucoma | Mutant | 480 | LOXL1G153D | 62.1633_61.4638_61.3262_60.3555_60.3285 | 62.1633 | 7.319_8.0381_8.0381_8.0381_8.0381 | 7.319 |
| LOXL1 | 153456 | Exfoliative glaucoma | Mutant | 480 | LOXL1R141L | 62.5076_60.9658_60.9236_60.2837_60.2052 | 62.5076 | 7.8274_9.2318_9.2318_9.2334_9.2331 | 7.8274 |
| LRAT | 604863 | Leber congenital amaurosis 14, Leber congenital amaurosis | WT | 230 | LRATWT | 84.6718_84.5812_84.0295_82.4261_82.0948 | 84.6718 | 7.6771_7.6912_7.6972_7.7004_7.7089 | 7.6771 |
| LRAT | 604863 | Leber congenital amaurosis 14, Leber congenital amaurosis | Mutant | 230 | LRATS175R | 79.4799_78.9373_78.2226_76.6273_74.7561 | 79.4799 | 7.959_7.9611_7.9624_7.9635_7.9632 | 7.959 |
| LRIT3 | 615004 | Congenital static night blindness, Night blindness, congenital stationary (complete), 1F, autosomal recessive | WT | 660 | LRIT3NM_198506WT | 70.3593_69.973_69.3451_68.943_67.7454 | 70.3593 | 8.0466_8.0467_8.0467_8.0467_8.0469 | 8.0466 |
| LRIT3 | 615004 | Congenital static night blindness, Night blindness, congenital stationary (complete), 1F, autosomal recessive | Mutant | 660 | LRIT3NM_198506C328Y | 70.0393_69.3183_68.4673_68.3975_67.5649 | 70.0393 | 8.0467_8.0467_8.0467_8.0469_8.0468 | 8.0467 |
| LRIT3 | 615004 | Congenital static night blindness, Night blindness, congenital stationary (complete), 1F, autosomal recessive | Mutant | 660 | LRIT3NM_198506W203L | 70.32_69.5105_68.9309_68.8847_68.8108 | 70.32 | 8.0467_8.0467_8.0468_8.0467_8.0468 | 8.0467 |
| LRP5 | 603506 | Familial exudative vitreoretinopathy | WT | 1584 | LRP5WT | 78.4973_78.3319_78.2898_78.115_75.0107 | 78.4973 | 8.0251_8.0253_8.0254_8.0255_8.0253 | 8.0251 |
| LRP5 | 603506 | Familial exudative vitreoretinopathy | Mutant | 1584 | LRP5C1361G | 78.7086_78.165_78.0084_77.4881_75.2871 | 78.7086 | 7.939_7.9391_7.9391_7.9392_7.9391 | 7.939 |
| LRP5 | 603506 | Familial exudative vitreoretinopathy | Mutant | 1584 | LRP5R570Q | 78.8221_78.5071_78.3164_78.1308_76.4957 | 78.8221 | 7.939_7.939_7.9391_7.9392_7.9391 | 7.939 |
| LRP5 | 603506 | Familial exudative vitreoretinopathy | Mutant | 1584 | LRP5T173M | 78.5953_78.3512_78.2219_77.7389_75.6438 | 78.5953 | 7.939_7.939_7.9391_7.9392_7.9392 | 7.939 |
| LTBP2 | 602091 | Primary open angle glaucoma, Corneal abnormality syndrome, Weill-Marchesani syndrome, Macrokeratosis, lens heterotopia and globular pharynx, Glaucoma 3, primary congenital, D | WT | 1786 | LTBP2NM_000428WT | 59.6258_57.584_56.8827_56.1509_55.8246 | 59.6258 | 7.987_8.0607_8.0702_8.0735_8.1241 | 7.987 |
| LTBP2 | 602091 | Primary open angle glaucoma, Corneal abnormality syndrome, Weill-Marchesani syndrome, Macrokeratosis, lens heterotopia and globular pharynx, Glaucoma 3, primary congenital, D | Mutant | 263 | LTBP2NM_000428R299X | 60.2209_56.821_56.7735_56.5012_55.8072 | 60.2209 | 7.9853_7.9858_7.9855_7.9858_7.9864 | 7.9853 |
| LTBP2 | 602091 | Primary open angle glaucoma, Corneal abnormality syndrome, Weill-Marchesani syndrome, Macrokeratosis, lens heterotopia and globular pharynx, Glaucoma 3, primary congenital, D | Mutant | 1786 | LTBP2NM_000428V1177M | 58.5947_57.4598_57.2717_56.3854_56.0338 | 58.5947 | 8.1192_8.1317_8.14_8.1421_8.1507 | 8.1192 |
| LZTFL1 | 606568 | Bardet-Biedl syndrome | WT | 299 | LZTFL1WT | 84.2418_84.1214_83.9746_83.3105_82.7633 | 84.2418 | 8.1891_8.1891_8.1891_8.1891_8.1891 | 8.1891 |
| LZTFL1 | 606568 | Bardet-Biedl syndrome | Mutant | 299 | LZTFL1L87P | 83.8082_82.9586_82.9506_82.6604_80.0585 | 83.8082 | 8.189_8.1891_8.189_8.189_8.1892 | 8.189 |
| MAF | 177075 | Cataract, Cataract 21, Cataracts, congenital, deafness, short stature, developmental delay, Congenital cataract and developmental cataract, Ayme-Gripp syndrome | WT | 403 | MAFWT | 57.2778_56.0604_55.1175_54.8239_53.7009 | 57.2778 | 8.0358_8.0358_8.0359_8.0359_8.036 | 8.0358 |
| MAF | 177075 | Cataract, Cataract 21, Cataracts, congenital, deafness, short stature, developmental delay, Congenital cataract and developmental cataract, Ayme-Gripp syndrome | Mutant | 403 | MAFK297R | 57.8309_56.0085_55.8864_55.2268_54.6354 | 57.8309 | 8.0358_8.0359_8.0359_8.0359_8.0359 | 8.0358 |
| MAF | 177075 | Cataract, Cataract 21, Cataracts, congenital, deafness, short stature, developmental delay, Congenital cataract and developmental cataract, Ayme-Gripp syndrome | Mutant | 403 | MAFQ303L | 57.8153_56.3749_55.6869_55.2694_53.8744 | 57.8153 | 8.036_8.036_8.036_8.0359_8.0359 | 8.036 |
| MAF | 177075 | Cataract, Cataract 21, Cataracts, congenital, deafness, short stature, developmental delay, Congenital cataract and developmental cataract, Ayme-Gripp syndrome | Mutant | 403 | MAFR288P | 57.5059_55.6341_55.4058_54.8035_54.6037 | 57.5059 | 8.036_8.0359_8.0359_8.036_8.0359 | 8.036 |
| MAK | 154235 | Retinitis pigmentosa | WT | 648 | MAKWT | 59.6089_58.9431_58.0624_57.3699_55.6481 | 59.6089 | 6.6941_8.0492_8.0493_8.0494_8.0494 | 6.6941 |
| MAK | 154235 | Retinitis pigmentosa | Mutant | 648 | MAKN130H | 60.1217_58.5982_57.5073_56.8783_55.5414 | 60.1217 | 6.7057_8.0492_8.0492_8.0495_8.0495 | 6.7057 |
| MAK | 154235 | Retinitis pigmentosa | Mutant | 648 | MAKR166H | 60.098_58.7983_57.4673_56.6076_55.3985 | 60.098 | 6.6921_8.0493_8.0494_8.0495_8.0494 | 6.6921 |
| MAN2B1 | 609458 | Mannosidosis, alpha- | WT | 962 | MAN2B1WT | 94.4615_94.3565_94.2064_94.1697_94.1203 | 94.4615 | 8.0502_8.0502_8.0502_8.0502_8.0502 | 8.0502 |
| MAN2B1 | 609458 | Mannosidosis, alpha- | Mutant | 962 | MAN2B1H72L | 94.4488_94.3908_94.3728_94.0653_94.0245 | 94.4488 | 8.0502_8.0503_8.0502_8.0502_8.0503 | 8.0502 |
| MAN2B1 | 609458 | Mannosidosis, alpha- | Mutant | 962 | MAN2B1S453Y | 94.4235_94.3137_94.224_94.2_94.1519 | 94.4235 | 8.05_8.0502_8.0502_8.0502_8.0503 | 8.05 |
| MANBA | 609489 | Nystagmus, Mannosidosis, beta | WT | 862 | MANBAWT | 97.2323_96.9453_96.9293_96.7252_96.5001 | 97.2323 | 5.0723_8.1822_8.1821_8.1822_8.1822 | 5.0723 |
| MANBA | 609489 | Nystagmus, Mannosidosis, beta | Mutant | 448 | MANBA466-879MISSING | 96.9338_96.9232_96.3265_96.2866_96.1677 | 96.9338 | 8.0377_8.0377_8.0378_8.0378_8.0377 | 8.0377 |
| MANBA | 609489 | Nystagmus, Mannosidosis, beta | Mutant | 862 | MANBAG392E | 96.8864_96.7996_96.7907_96.608_96.3989 | 96.8864 | 5.0971_8.182_8.1821_8.1822_8.1821 | 5.0971 |
| MANBA | 609489 | Nystagmus, Mannosidosis, beta | Mutant | 862 | MANBAR182W | 97.0566_97.036_96.6606_96.4258_96.3778 | 97.0566 | 5.076_8.1819_8.182_8.1821_8.1821 | 5.076 |
| MAPT | 157140 | Supranuclear palsy, progressive | WT | 757 | MAPTWT | 49.2879_45.4587_45.0016_44.181_44.0459 | 49.2879 | 3.6295_7.8453_7.8555_7.8627_7.8693 | 3.6295 |
| MAPT | 157140 | Supranuclear palsy, progressive | Mutant | 757 | MAPTG620V | 49.2469_44.8128_44.1981_43.8525_43.7376 | 49.2469 | 4.1951_7.8522_7.8532_7.8533_7.8532 | 4.1951 |
| MAPT | 157140 | Supranuclear palsy, progressive | Mutant | 757 | MAPTR5L | 48.5535_45.3362_44.933_44.2934_44.2699 | 48.5535 | 3.8552_7.8534_7.8551_7.8586_7.871 | 3.8552 |
| MCOLN1 | 605248 | Mucolipidosis IV, Mucolipidosis, Mucopolysaccharidosis | WT | 580 | MCOLN1WT | 81.2947_80.5309_80.0187_77.4957_76.6348 | 81.2947 | 8.0467_8.0466_8.0467_8.0467_8.0467 | 8.0467 |
| MCOLN1 | 605248 | Mucolipidosis IV, Mucolipidosis, Mucopolysaccharidosis | Mutant | 580 | MCOLN1D362Y | 82.1317_80.5857_79.655_76.7347_76.3069 | 82.1317 | 8.0466_8.0467_8.0467_8.0467_8.0467 | 8.0466 |
| MCOLN1 | 605248 | Mucolipidosis IV, Mucolipidosis, Mucopolysaccharidosis | Mutant | 580 | MCOLN1T232P | 82.4164_81.1627_80.4625_77.9138_77.7845 | 82.4164 | 8.0467_8.0468_8.0469_8.0467_8.0468 | 8.0467 |
| MERTK | 604705 | Retinitis pigmentosa | WT | 979 | MERTKWT | 72.5114_71.2256_69.6551_69.5238_69.0664 | 72.5114 | 8.047_8.047_8.047_8.0471_8.0471 | 8.047 |
| MERTK | 604705 | Retinitis pigmentosa | Mutant | 979 | MERTKP763S | 71.8036_70.5699_70.1682_69.083_69.0364 | 71.8036 | 8.0471_8.0471_8.0471_8.0471_8.0471 | 8.0471 |
| MERTK | 604705 | Retinitis pigmentosa | Mutant | 979 | MERTKR844S | 72.5143_70.2823_69.8202_69.0134_68.5352 | 72.5143 | 8.0471_8.047_8.0472_8.0471_8.0472 | 8.0471 |
| MERTK | 604705 | Retinitis pigmentosa | Mutant | 85 | MERTKT99fs | 66.4368_65.4064_65.0487_63.9742_61.1055 | 66.4368 | 8.1861_8.1872_8.1871_8.1874_8.1876 | 8.1861 |
| MFN2 | 608507 | Charcot-Marie-Tooth disease, axonal, type 2A | WT | 757 | MFN2WT | 81.7986_81.2705_77.5396_77.3053_76.9875 | 81.7986 | 3.8694_7.4308_7.92_7.92_8.0479 | 3.8694 |
| MFN2 | 608507 | Charcot-Marie-Tooth disease, axonal, type 2A | Mutant | 757 | MFN2R707W | 80.0243_80.0196_78.1052_77.3531_77.0564 | 80.0243 | 4.0057_8.0236_8.0242_8.0243_8.0249 | 4.0057 |
| MFN2 | 608507 | Charcot-Marie-Tooth disease, axonal, type 2A | Mutant | 757 | MFN2R94Q | 81.5208_81.4092_78.0577_77.8048_77.4576 | 81.5208 | 3.977_7.4513_8.049_8.0682_8.0692 | 3.977 |
| MFRP | 606227 | Nanophthalmos, Microphthalmia, Microphthalmia | WT | 579 | MFRPWT | 72.2852_71.1821_71.0883_70.4288_70.0197 | 72.2852 | 8.0391_8.0389_8.0391_8.0389_8.0391 | 8.0391 |
| MFRP | 606227 | Nanophthalmos, Microphthalmia, Microphthalmia | Mutant | 579 | MFRPI182T | 71.4748_70.718_69.9033_69.6961_68.4373 | 71.4748 | 8.039_8.039_8.0391_8.0389_8.039 | 8.039 |
| MFSD8 | 611124 | Macular dystrophy, Ceroid lipofuscinosis, neuronal, Macular degeneration, age-related | WT | 518 | MFSD8WT | 83.4408_83.2152_82.941_82.7048_82.5028 | 83.4408 | 5.3031_7.932_7.9319_7.9323_7.9325 | 5.3031 |
| MFSD8 | 611124 | Macular dystrophy, Ceroid lipofuscinosis, neuronal, Macular degeneration, age-related | Mutant | 518 | MFSD8G310D | 82.5927_82.5206_82.4117_82.3447_82.2081 | 82.5927 | 5.2704_7.9314_7.9319_7.9318_7.9323 | 5.2704 |
| MFSD8 | 611124 | Macular dystrophy, Ceroid lipofuscinosis, neuronal, Macular degeneration, age-related | Mutant | 518 | MFSD8T294K | 83.7525_82.4234_82.3365_82.2979_82.1956 | 83.7525 | 5.2694_7.9318_7.9319_7.9322_7.9317 | 5.2694 |
| MIP | 154050 | Congenital cataract and developmental cataract | WT | 263 | MIPWT | 91.2281_91.1503_91.1139_90.9602_90.7196 | 91.2281 | 7.9907_7.9918_7.9912_7.9912_7.9922 | 7.9907 |
| MIP | 154050 | Congenital cataract and developmental cataract | Mutant | 263 | MIPE134G | 91.1683_91.1334_91.1186_90.96_90.7117 | 91.1683 | 7.8345_7.8348_7.8352_7.8358_7.8377 | 7.8345 |
| MIP | 154050 | Congenital cataract and developmental cataract | Mutant | 263 | MIPR33C | 91.2582_91.1908_91.1733_91.1353_91.0228 | 91.2582 | 7.9909_7.9911_7.9911_7.992_7.9926 | 7.9909 |
| MIP | 154050 | Congenital cataract and developmental cataract | Mutant | 263 | MIPT138R | 90.8921_90.8612_90.6469_90.5701_89.7265 | 90.8921 | 7.9923_7.9915_7.9919_7.992_7.9916 | 7.9923 |
| MKKS | 604896 | Bardet-Biedl syndrome | WT | 570 | MKKSWT | 89.1315_88.3314_88.2848_88.1637_87.3669 | 89.1315 | 5.3444_8.0322_8.0322_8.0324_8.0323 | 5.3444 |
| MKKS | 604896 | Bardet-Biedl syndrome | Mutant | 570 | MKKSA242S | 89.27_88.7287_88.1238_88.1013_88.0122 | 89.27 | 5.3191_8.0323_8.0324_8.0323_8.0323 | 5.3191 |
| MKKS | 604896 | Bardet-Biedl syndrome | Mutant | 570 | MKKSY37C | 89.0678_88.5524_88.4522_88.3736_87.5922 | 89.0678 | 5.3054_8.0323_8.0324_8.0324_8.0324 | 5.3054 |
| MKS1 | 609883 | Bardet-Biedl syndrome, Meckel syndrome | WT | 559 | MKS1WT | 75.9523_74.4926_73.3813_72.3301_71.7174 | 75.9523 | 8.0314_8.0313_8.0315_8.0314_8.0314 | 8.0314 |
| MKS1 | 609883 | Bardet-Biedl syndrome, Meckel syndrome | Mutant | 558 | MKS1372MISSING | 75.2335_73.2761_73.2572_73.254_71.2915 | 75.2335 | 8.0341_8.0342_8.0342_8.0341_8.0342 | 8.0341 |
| MKS1 | 609883 | Bardet-Biedl syndrome, Meckel syndrome | Mutant | 559 | MKS1R166W | 74.7523_74.5547_72.6293_72.1419_71.3829 | 74.7523 | 8.0313_8.0313_8.0313_8.0314_8.0315 | 8.0313 |
| MPDZ | 603785 | Hydrocephalus, congenital, 2, with or without brain or eye anomalies | Mutant | 209 | MPDZ210-2070MISSING | 75.0409_73.8689_73.6483_72.7177_72.0975 | 75.0409 | 7.9044_7.905_7.9045_7.9072_7.9059 | 7.9044 |
| MTHFR | 607093 | Homocystinuria due to MTHFR deficiency, Homocysteinuria | WT | 656 | MTHFRWT | 88.4953_87.8792_85.9634_85.4024_84.04 | 88.4953 | 7.5984_8.0433_8.0435_8.0434_8.0434 | 7.5984 |
| MTHFR | 607093 | Homocystinuria due to MTHFR deficiency, Homocysteinuria | Mutant | 656 | MTHFRP572L | 88.1947_87.841_85.3316_84.5821_82.6323 | 88.1947 | 7.56_8.0432_8.0433_8.0434_8.0435 | 7.56 |
| MTHFR | 607093 | Homocystinuria due to MTHFR deficiency, Homocysteinuria | Mutant | 656 | MTHFRR157Q | 88.5483_87.7196_85.6103_85.5563_84.0735 | 88.5483 | 7.6949_8.0433_8.0435_8.0434_8.0432 | 7.6949 |
| MTTP | 157147 | Bassen Kornzweig syndrome, Syndromic retinitis pigmentosa | WT | 876 | MTTPWT | 88.4383_88.3536_87.618_87.1791_85.8521 | 88.4383 | 8.1755_8.1756_8.1757_8.1756_8.1757 | 8.1755 |
| MTTP | 157147 | Bassen Kornzweig syndrome, Syndromic retinitis pigmentosa | Mutant | 876 | MTTPN780Y | 93.2033_92.7429_88.0316_87.8493_85.4234 | 93.2033 | 8.1757_8.1757_8.1757_8.1757_8.1758 | 8.1757 |
| MTTP | 157147 | Bassen Kornzweig syndrome, Syndromic retinitis pigmentosa | Mutant | 876 | MTTPR540H | 93.0621_92.8106_88.2797_87.4357_86.9347 | 93.0621 | 8.1757_8.1756_8.1756_8.1757_8.1756 | 8.1757 |
| MYH2 | 160740 | Proximal myopathy and ophthalmoplegia 3 | WT | 1941 | MYH2WT | 72.8872_71.9175_71.0227_68.4141_62.6622 | 72.8872 | 8.1811_8.1811_8.1812_8.1812_8.1811 | 8.1811 |
| MYH2 | 160740 | Proximal myopathy and ophthalmoplegia 3 | Mutant | 1941 | MYH2E706K | 71.9536_71.4995_70.8476_68.5603_62.7148 | 71.9536 | 8.1812_8.1812_8.1813_8.1813_8.1813 | 8.1812 |
| MYH7 | 160760 | Myopathy, myosin storage, autosomal dominant | WT | 1935 | MYH7WT | 72.6163_72.0889_71.0965_70.6011_63.5445 | 72.6163 | 8.1803_8.1815_8.1815_8.1815_8.1815 | 8.1803 |
| MYH7 | 160760 | Myopathy, myosin storage, autosomal dominant | Mutant | 1935 | MYH7H1901L | 72.3108_71.1885_70.9453_69.6185_63.4398 | 72.3108 | 8.1815_8.1815_8.1815_8.1815_8.1815 | 8.1815 |
| MYH7 | 160760 | Myopathy, myosin storage, autosomal dominant | Mutant | 1935 | MYH7R1845W | 72.2041_71.045_69.7825_69.6947_63.8661 | 72.2041 | 8.1813_8.1813_8.1813_8.1814_8.1815 | 8.1813 |
| MYO7A | 276903 | Usher syndrome | WT | 2215 | MYO7AWT | 77.5772_76.977_76.7137_76.4544_75.7779 | 77.5772 | 7.9537_7.9537_7.9537_7.9537_7.9537 | 7.9537 |
| MYO7A | 276903 | Usher syndrome | Mutant | 2215 | MYO7AE1170K | 77.4965_76.6753_76.5869_76.4881_76.1117 | 77.4965 | 7.9537_7.9537_7.9537_7.9538_7.9537 | 7.9537 |
| MYO7A | 276903 | Usher syndrome | Mutant | 2215 | MYO7AT165M | 77.0037_76.9983_76.9642_75.7977_75.176 | 77.0037 | 7.9537_7.9537_7.9537_7.9537_7.9537 | 7.9537 |
| MYOC | 601652 | Primary open angle glaucoma, Townes-Brocks branchiootorenal-like syndrome, Cohen syndrome | WT | 472 | MYOCNM_000261WT | 82.0835_81.9914_81.8099_81.5915_80.6136 | 82.0835 | 8.9486_9.2314_9.2324_9.2321_9.2327 | 8.9486 |
| MYOC | 601652 | Primary open angle glaucoma, Townes-Brocks branchiootorenal-like syndrome, Cohen syndrome | Mutant | 472 | MYOCC245Y | 82.0859_81.797_81.7775_80.3967_79.009 | 82.0859 | 7.0728_8.044_8.0441_8.0441_8.044 | 7.0728 |
| MYOC | 601652 | Primary open angle glaucoma, Townes-Brocks branchiootorenal-like syndrome, Cohen syndrome | Mutant | 472 | MYOCD208E | 81.9635_81.8598_81.0896_80.6717_80.1893 | 81.9635 | 7.0244_8.044_8.0441_8.044_8.0441 | 7.0244 |
| MYOC | 601652 | Primary open angle glaucoma, Townes-Brocks branchiootorenal-like syndrome, Cohen syndrome | Mutant | 472 | MYOCD380A-D478S | 82.9864_82.5485_82.0945_80.966_80.781 | 82.9864 | 7.0932_8.044_8.0442_8.0441_8.0441 | 7.0932 |
| MYOC | 601652 | Primary open angle glaucoma, Townes-Brocks branchiootorenal-like syndrome, Cohen syndrome | Mutant | 472 | MYOCD478N | 81.8991_81.702_80.8933_80.4642_77.9427 | 81.8991 | 7.0657_8.044_8.044_8.0439_8.0441 | 7.0657 |
| MYOC | 601652 | Primary open angle glaucoma, Townes-Brocks branchiootorenal-like syndrome, Cohen syndrome | Mutant | 472 | MYOCD478S | 81.9882_81.8208_81.7125_80.949_78.5793 | 81.9882 | 7.0922_8.0441_8.0441_8.044_8.044 | 7.0922 |
| MYOC | 601652 | Primary open angle glaucoma, Townes-Brocks branchiootorenal-like syndrome, Cohen syndrome | Mutant | 472 | MYOCE323K | 81.7901_81.7157_81.49_81.1879_79.4783 | 81.7901 | 6.9447_8.0441_8.0441_8.044_8.044 | 6.9447 |
| MYOC | 601652 | Primary open angle glaucoma, Townes-Brocks branchiootorenal-like syndrome, Cohen syndrome | Mutant | 472 | MYOCE396D | 82.1518_82.1482_81.8769_80.7313_78.7951 | 82.1518 | 8.3638_9.2601_9.2618_9.261_9.2618 | 8.3638 |
| MYOC | 601652 | Primary open angle glaucoma, Townes-Brocks branchiootorenal-like syndrome, Cohen syndrome | Mutant | 472 | MYOCG252R | 82.9742_81.7495_81.6688_80.5663_79.64 | 82.9742 | 7.0626_8.044_8.0439_8.044_8.044 | 7.0626 |
| MYOC | 601652 | Primary open angle glaucoma, Townes-Brocks branchiootorenal-like syndrome, Cohen syndrome | Mutant | 335 | MYOCGln368X | 79.6524_79.1221_78.7714_78.0615_77.721 | 79.6524 | 10.474_10.2824_10.2121_9.315_10.5152 | 10.474 |
| MYOC | 601652 | Primary open angle glaucoma, Townes-Brocks branchiootorenal-like syndrome, Cohen syndrome | Mutant | 472 | MYOCN428D-D478H | 82.6459_82.2097_81.9916_81.0541_79.6501 | 82.6459 | 6.9358_8.0441_8.044_8.044_8.044 | 6.9358 |
| MYOC | 601652 | Primary open angle glaucoma, Townes-Brocks branchiootorenal-like syndrome, Cohen syndrome | Mutant | 472 | MYOCN428E-D478K | 82.5033_82.2816_81.796_80.5603_78.3221 | 82.5033 | 7.0708_8.0441_8.0441_8.0441_8.044 | 7.0708 |
| MYOC | 601652 | Primary open angle glaucoma, Townes-Brocks branchiootorenal-like syndrome, Cohen syndrome | Mutant | 472 | MYOCN428E-D478S | 82.4321_81.9767_81.6734_81.288_79.55 | 82.4321 | 7.0628_8.044_8.0441_8.0441_8.0441 | 7.0628 |
| MYOC | 601652 | Primary open angle glaucoma, Townes-Brocks branchiootorenal-like syndrome, Cohen syndrome | Mutant | 472 | MYOCNM_000261A488V | 82.2422_81.9321_81.536_81.4015_80.1137 | 82.2422 | 8.3379_9.2321_9.2321_9.2323_9.2327 | 8.3379 |
| MYOC | 601652 | Primary open angle glaucoma, Townes-Brocks branchiootorenal-like syndrome, Cohen syndrome | Mutant | 472 | MYOCNM_000261D384H | 82.7086_82.3573_82.2822_81.543_79.8712 | 82.7086 | 6.9525_8.044_8.044_8.044_8.0441 | 6.9525 |
| MYOC | 601652 | Primary open angle glaucoma, Townes-Brocks branchiootorenal-like syndrome, Cohen syndrome | Mutant | 472 | MYOCNM_000261G367R | 82.2948_82.1887_81.5708_81.0555_79.738 | 82.2948 | 7.0843_8.0441_8.0441_8.044_8.0441 | 7.0843 |
| MYOC | 601652 | Primary open angle glaucoma, Townes-Brocks branchiootorenal-like syndrome, Cohen syndrome | Mutant | 472 | MYOCNM_000261P370L | 82.0341_82.0043_81.5472_80.6987_80.3607 | 82.0341 | 8.3867_9.2317_9.232_9.2323_9.2327 | 8.3867 |
| MYOC | 601652 | Primary open angle glaucoma, Townes-Brocks branchiootorenal-like syndrome, Cohen syndrome | Mutant | 472 | MYOCNM_000261S313F | 82.4479_82.1183_81.6252_80.5504_79.4343 | 82.4479 | 7.0345_8.044_8.044_8.044_8.044 | 7.0345 |
| MYOC | 601652 | Primary open angle glaucoma, Townes-Brocks branchiootorenal-like syndrome, Cohen syndrome | Mutant | 472 | MYOCQ48H | 82.1179_82.0767_81.9419_81.2543_80.0837 | 82.1179 | 7.1348_8.0439_8.0439_8.0441_8.0441 | 7.1348 |
| MYOC | 601652 | Primary open angle glaucoma, Townes-Brocks branchiootorenal-like syndrome, Cohen syndrome | Mutant | 472 | MYOCT353I | 82.6368_82.3033_82.083_81.0284_80.4981 | 82.6368 | 7.0839_8.0439_8.0439_8.0441_8.0441 | 7.0839 |
| MYOT | 604103 | Muscular dystrophy, limb-girdle | WT | 498 | MYOTWT | 64.9665_62.4855_62.4077_62.1234_61.1438 | 64.9665 | 8.2318_8.2317_8.2317_8.2318_8.2318 | 8.2318 |
| MYOT | 604103 | Muscular dystrophy, limb-girdle | Mutant | 498 | MYOTS55F | 65.569_62.2619_62.1629_62.1139_61.1141 | 65.569 | 8.2317_8.2319_8.2318_8.2317_8.2319 | 8.2317 |
| MYOT | 604103 | Muscular dystrophy, limb-girdle | Mutant | 498 | MYOTS60F | 64.9385_63.0642_62.9606_61.4777_61.0917 | 64.9385 | 9.4974_9.5213_9.5283_9.6008_9.6125 | 9.4974 |
| NAA10 | 300013 | Microphthalmia, Ogden syndrome | WT | 235 | NAA10WT | 80.1549_78.6968_78.6871_78.6847_78.6806 | 80.1549 | 7.9703_7.9711_7.974_7.9736_7.9741 | 7.9703 |
| NAA10 | 300013 | Microphthalmia, Ogden syndrome | Mutant | 235 | NAA10S37P | 79.6442_78.5753_78.4913_78.1009_78.0497 | 79.6442 | 7.9698_7.9705_7.9725_7.9734_7.9745 | 7.9698 |
| NDP | 300658 | Norrie disease, Familial exudative vitreoretinopathy | WT | 109 | NDPWT | 90.6661_90.4089_89.1784_88.992_88.9746 | 90.6661 | 7.7_7.9682_7.9727_7.9756_7.9664 | 7.7 |
| NDP | 300658 | Norrie disease, Familial exudative vitreoretinopathy | Mutant | 109 | NDPR121C | 92.2043_91.9581_90.1768_89.986_89.0782 | 92.2043 | 7.6082_7.96_7.9596_7.9684_7.9625 | 7.6082 |
| NDP | 300658 | Norrie disease, Familial exudative vitreoretinopathy | Mutant | 109 | NDPR38C | 91.8687_91.033_89.7285_89.1799_88.1214 | 91.8687 | 7.6136_7.972_7.9712_7.9717_7.9709 | 7.6136 |
| NHS | 300457 | Congenital cataract and developmental cataract | WT | 1630 | NHSWT | 43.8382_40.4439_40.1649_39.9085_39.9031 | 43.8382 | 8.0249_8.025_8.025_8.025_8.025 | 8.0249 |
| NHS | 300457 | Congenital cataract and developmental cataract | Mutant | 1202 | NHSK1198fs | 47.3151_42.7662_42.5134_42.4602_42.2409 | 47.3151 | 8.1703_8.1704_8.1717_8.1718_8.1718 | 8.1703 |
| NHS | 300457 | Congenital cataract and developmental cataract | Mutant | 852 | NHSK850fsX852 | 51.9916_48.4278_47.7076_47.2815_47.046 | 51.9916 | 4.8422_8.1811_8.181_8.1811_8.1811 | 4.8422 |
| NMNAT1 | 608700 | Leber congenital amaurosis 9, Leber congenital amaurosis | WT | 279 | NMNAT1NM_022787.4WT | 89.1059_88.6602_88.5251_88.3541_88.1912 | 89.1059 | 8.0557_8.0567_8.0561_8.0564_8.0569 | 8.0557 |
| NMNAT1 | 608700 | Leber congenital amaurosis 9, Leber congenital amaurosis | Mutant | 279 | NMNAT1NM_022787.4E257K | 89.1203_88.7675_88.5168_88.4322_88.2632 | 89.1203 | 7.9365_7.9365_7.9366_7.9366_7.9366 | 7.9365 |
| NMNAT1 | 608700 | Leber congenital amaurosis 9, Leber congenital amaurosis | Mutant | 279 | NMNAT1NM_022787.4V98G | 89.2916_88.6993_88.5479_88.4184_88.1551 | 89.2916 | 7.8528_7.8841_7.946_7.9508_7.9541 | 7.8528 |
| NPHP1 | 607100 | Senior-Loken syndrome, Joubert syndrome, Nephronophthisis | WT | 732 | NPHP1WT | 76.1617_75.59_75.5063_74.8689_74.5805 | 76.1617 | 7.9527_7.961_7.9638_7.9651_7.9741 | 7.9527 |
| NPHP1 | 607100 | Senior-Loken syndrome, Joubert syndrome, Nephronophthisis | Mutant | 732 | NPHP1G342R | 75.6087_75.1636_74.7779_74.0491_72.5038 | 75.6087 | 7.9642_7.9665_7.9676_7.9779_7.9785 | 7.9642 |
| NPHP1 | 607100 | Senior-Loken syndrome, Joubert syndrome, Nephronophthisis | Mutant | 732 | NPHP1L180P | 76.5537_75.0165_74.857_74.3323_73.7375 | 76.5537 | 7.9703_7.9716_7.9721_7.9732_7.9857 | 7.9703 |
| NPHP3 | 608002 | Meckel syndrome, Nephronophthisis | WT | 1329 | NPHP3WT | 70.9612_70.8643_69.0734_68.2025_67.6996 | 70.9612 | 7.93_7.93_7.93_7.93_7.93 | 7.93 |
| NPHP3 | 608002 | Meckel syndrome, Nephronophthisis | Mutant | 1329 | NPHP3N386S | 72.0738_70.848_69.8981_68.2268_68.0448 | 72.0738 | 8.0504_8.0503_8.0504_8.0504_8.0504 | 8.0504 |
| NPHP3 | 608002 | Meckel syndrome, Nephronophthisis | Mutant | 1329 | NPHP3R397H | 71.9986_70.8224_69.9989_69.5745_67.8788 | 71.9986 | 7.93_7.93_7.93_7.93_7.93 | 7.93 |
| NPHP4 | 607215 | Senior-Loken syndrome, Nephronophthisis | WT | 1426 | NPHP4WT | 72.0491_71.8524_71.4906_70.94_70.3365 | 72.0491 | 7.3958_8.0543_8.0561_8.0562_8.0562 | 7.3958 |
| NPHP4 | 607215 | Senior-Loken syndrome, Nephronophthisis | Mutant | 1426 | NPHP4F91L | 72.4209_71.4275_71.407_71.2457_69.8738 | 72.4209 | 7.3462_8.0482_8.0484_8.0486_8.0486 | 7.3462 |
| NPHP4 | 607215 | Senior-Loken syndrome, Nephronophthisis | Mutant | 1426 | NPHP4G754R | 71.776_71.7381_71.2479_70.9421_69.3038 | 71.776 | 7.423_8.0558_8.0562_8.0562_8.0562 | 7.423 |
| NR2E3 | 604485 | Retinitis pigmentosa, Enhanced S-cone syndrome, Goldmann - Favre syndrome | WT | 410 | NR2E3WT | 74.7217_72.1931_71.4621_70.8003_70.7777 | 74.7217 | 8.0346_8.0346_8.0346_8.0346_8.0347 | 8.0346 |
| NR2E3 | 604485 | Retinitis pigmentosa, Enhanced S-cone syndrome, Goldmann - Favre syndrome | Mutant | 410 | NR2E3H361R | 75.1046_71.9846_71.3615_71.1112_70.8127 | 75.1046 | 9.3933_9.3935_9.3952_9.3941_9.3947 | 9.3933 |
| NR2E3 | 604485 | Retinitis pigmentosa, Enhanced S-cone syndrome, Goldmann - Favre syndrome | Mutant | 410 | NR2E3P376L | 75.0644_72.136_71.8046_71.4045_70.5476 | 75.0644 | 8.0345_8.0346_8.0346_8.0346_8.0346 | 8.0345 |
| NR2F1 | 132890 | Optic atrophy | WT | 423 | NR2F1WT | 75.6303_74.017_73.352_73.3341_72.5055 | 75.6303 | 9.3891_9.3904_9.3895_9.3908_9.389 | 9.3891 |
| NR2F1 | 132890 | Optic atrophy | Mutant | 423 | NR2F1L252P | 75.5352_74.0802_73.2228_73.1143_72.9742 | 75.5352 | 8.0323_8.0326_8.0325_8.0325_8.0326 | 8.0323 |
| NR2F1 | 132890 | Optic atrophy | Mutant | 423 | NR2F1R115P | 75.2073_73.6524_73.2099_73.1731_72.6639 | 75.2073 | 8.0324_8.0325_8.0326_8.0325_8.0325 | 8.0324 |
| NRL | 162080 | Retinitis pigmentosa | WT | 237 | NRLWT | 71.2621_70.6247_69.7939_69.4225_68.9058 | 71.2621 | 7.9108_7.911_7.9466_7.9477_7.9481 | 7.9108 |
| NRL | 162080 | Retinitis pigmentosa | Mutant | 237 | NRLG116W | 71.1236_70.0652_70.0573_69.8706_68.7237 | 71.1236 | 7.9119_7.9148_7.9152_7.9156_7.9154 | 7.9119 |
| NTF4 | 162662 | Glaucoma, Primary open angle glaucoma | WT | 186 | NTF4NM_006179WT | 79.6004_78.7179_75.2995_75.0345_74.5486 | 79.6004 | 5.9935_8.1346_8.1374_8.1636_8.1683 | 5.9935 |
| NTF4 | 162662 | Glaucoma, Primary open angle glaucoma | Mutant | 186 | NTF4NM_006179A182V | 79.6004_78.7646_75.4644_75.4236_74.0923 | 79.6004 | 6.0715_8.1478_8.1641_8.1475_8.163 | 6.0715 |
| NTF4 | 162662 | Glaucoma, Primary open angle glaucoma | Mutant | 186 | NTF4NM_006179G157A | 78.7161_78.4427_76.3848_76.1991_75.211 | 78.7161 | 6.0581_8.131_8.1403_8.1966_8.2104 | 6.0581 |
| NTF4 | 162662 | Glaucoma, Primary open angle glaucoma | Mutant | 186 | NTF4NM_006179R206W | 78.2681_78.2661_75.6167_74.733_74.0254 | 78.2681 | 6.089_8.1286_8.1697_8.1489_8.1686 | 6.089 |
| NYX | 300278 | Night blindness, congenital stationary (complete), 1A, X-linked, Congenital static night blindness | WT | 453 | NYXWT | 82.9482_82.6563_81.8353_81.7512_81.0212 | 82.9482 | 8.81_8.81_8.81_8.81_9.2722 | 8.81 |
| NYX | 300278 | Night blindness, congenital stationary (complete), 1A, X-linked, Congenital static night blindness | Mutant | 449 | NYX243-246missing | 83.095_81.8985_81.814_81.6895_81.3502 | 83.095 | 9.2736_9.2736_9.2741_9.2739_9.2749 | 9.2736 |
| NYX | 300278 | Night blindness, congenital stationary (complete), 1A, X-linked, Congenital static night blindness | Mutant | 445 | NYX29-36MISSING | 82.7741_82.0466_81.6732_81.3051_80.8322 | 82.7741 | 9.314_9.315_9.3144_9.3134_9.3127 | 9.314 |
| OAT | 613349 | Gyrate atrophy of choroid and retina with or without ornithinemia | WT | 404 | OATWT | 97.5692_97.4949_97.4856_97.4079_97.25 | 97.5692 | 9.4188_9.42_9.4203_9.4201_9.4205 | 9.4188 |
| OAT | 613349 | Gyrate atrophy of choroid and retina with or without ornithinemia | Mutant | 404 | OATR180T | 97.4662_97.4261_97.3596_97.3098_97.1955 | 97.4662 | 8.0422_8.0423_8.0422_8.0423_8.0422 | 8.0422 |
| OAT | 613349 | Gyrate atrophy of choroid and retina with or without ornithinemia | Mutant | 404 | OATV332M | 97.6593_97.5611_97.5178_97.4144_97.223 | 97.6593 | 8.0423_8.0425_8.0424_8.0424_8.0424 | 8.0423 |
| OCA2 | 611409 | Albinism, oculocutaneous, Albinism, oculocutaneous, Ectopia lentis et pupillae | WT | 838 | OCA2WT | 73.9123_73.848_73.81_73.4339_73.3151 | 73.9123 | 8.1023_8.175_8.1763_8.1764_8.1776 | 8.1023 |
| OCA2 | 611409 | Albinism, oculocutaneous, Albinism, oculocutaneous, Ectopia lentis et pupillae | Mutant | 838 | OCA2R290G | 73.8021_73.6273_73.4174_73.2633_71.3081 | 73.8021 | 8.2243_8.174_8.1741_8.174_8.1742 | 8.2243 |
| OCA2 | 611409 | Albinism, oculocutaneous, Albinism, oculocutaneous, Ectopia lentis et pupillae | Mutant | 838 | OCA2W679C | 73.5528_73.4832_73.2922_72.8559_72.558 | 73.5528 | 8.027_8.0565_8.0573_8.0569_8.057 | 8.027 |
| OCRL | 300535 | Itm2b-associated cerebral amyloid vascular disease type 2 | WT | 901 | OCRLWT | 82.8724_82.8242_81.5776_81.4676_81.4453 | 82.8724 | 8.0382_8.0435_8.0439_8.0447_8.045 | 8.0382 |
| OCRL | 300535 | Itm2b-associated cerebral amyloid vascular disease type 2 | Mutant | 901 | OCRLR318C | 83.1417_82.6969_81.4439_80.6816_80.3149 | 83.1417 | 8.039_8.0429_8.0454_8.0455_8.0469 | 8.039 |
| OCRL | 300535 | Itm2b-associated cerebral amyloid vascular disease type 2 | Mutant | 901 | OCRLR500Q | 82.7046_82.6993_81.3622_81.3565_80.8057 | 82.7046 | 8.0397_8.0427_8.0424_8.0439_8.0447 | 8.0397 |
| OFD1 | 300170 | Joubert syndrome, Retinitis pigmentosa | WT | 1012 | OFD1WT | 67.6434_67.0786_65.2589_64.3745_64.1902 | 67.6434 | 8.2776_8.2775_8.2777_8.2777_8.2778 | 8.2776 |
| OFD1 | 300170 | Joubert syndrome, Retinitis pigmentosa | Mutant | 1012 | OFD1T737A | 67.9049_67.58_65.4762_64.5292_62.5067 | 67.9049 | 8.2776_8.2776_8.2776_8.2778_8.2778 | 8.2776 |
| OPA1 | 605290 | Optic atrophy, Behr syndrome, Optic atrophy plus syndrome, Normal tension glaucoma | WT | 873 | OPA1WT | 77.3389_77.2236_76.9837_76.4422_75.3801 | 77.3389 | 8.1776_8.1775_8.1776_8.1777_8.1778 | 8.1776 |
| OPA1 | 605290 | Optic atrophy, Behr syndrome, Optic atrophy plus syndrome, Normal tension glaucoma | Mutant | 873 | OPA1R290Q | 77.5854_77.3602_76.9326_76.8637_74.1075 | 77.5854 | 8.1776_8.1776_8.1777_8.1778_8.1777 | 8.1776 |
| OPA1 | 605290 | Optic atrophy, Behr syndrome, Optic atrophy plus syndrome, Normal tension glaucoma | Mutant | 873 | OPA1R445H | 77.3305_76.6907_76.5836_76.4693_74.1024 | 77.3305 | 8.1776_8.1777_8.1778_8.1777_8.1778 | 8.1776 |
| OPA1 | 605290 | Optic atrophy, Behr syndrome, Optic atrophy plus syndrome, Normal tension glaucoma | Mutant | 873 | OPA1S545R | 77.731_77.563_77.3921_76.7924_75.5965 | 77.731 | 7.9814_7.9993_8.052_8.0534_8.0529 | 7.9814 |
| OPA3 | 606580 | Optic atrophy, Optic atrophy 3 with cataract, Optic atrophy plus syndrome | WT | 178 | OPA3WT | 82.8143_79.1084_79.0096_74.5926_73.5515 | 82.8143 | 8.1473_8.1553_8.1644_8.1603_8.1813 | 8.1473 |
| OPA3 | 606580 | Optic atrophy, Optic atrophy 3 with cataract, Optic atrophy plus syndrome | Mutant | 178 | OPA3G93S | 83.1931_81.1061_79.2661_77.9268_74.1242 | 83.1931 | 8.0946_8.0987_8.1007_8.097_8.0952 | 8.0946 |
| OPA3 | 606580 | Optic atrophy, Optic atrophy 3 with cataract, Optic atrophy plus syndrome | Mutant | 178 | OPA3Q105E | 83.552_80.9795_80.0827_77.2045_73.9356 | 83.552 | 8.1486_8.1535_8.154_8.1483_8.1542 | 8.1486 |
| OPN1SW | 613522 | Achromatopsia, Colorblindness, tritan | WT | 345 | OPN1SWT190I | 86.8138_85.4809_85.1811_84.506_81.7716 | 86.8138 | 7.7537_8.01_8.0101_8.01_8.0101 | 7.7537 |
| OPN1SW | 613522 | Achromatopsia, Colorblindness, tritan | WT | 345 | OPN1SWWT | 86.9782_86.2688_85.3073_84.4089_83.4851 | 86.9782 | 7.7555_8.01_8.01_8.01_8.0101 | 7.7555 |
| OPN1SW | 613522 | Achromatopsia, Colorblindness, tritan | Mutant | 345 | OPN1SWP261S | 87.0667_84.7946_84.6566_83.5787_81.4165 | 87.0667 | 7.7222_8.0101_8.0101_8.0101_8.0101 | 7.7222 |
| OPN1SW | 613522 | Achromatopsia, Colorblindness, tritan | Mutant | 345 | OPN1SWS211P | 87.9979_86.429_86.2081_85.4272_83.7178 | 87.9979 | 7.7602_8.01_8.01_8.01_8.01 | 7.7602 |
| OPTN | 602432 | Glaucoma, Normal tension glaucoma, Primary open angle glaucoma | WT | 577 | OPTNWT | 77.7099_77.5998_77.1367_76.8034_75.3606 | 77.7099 | 8.0384_8.0385_8.0385_8.0384_8.0384 | 8.0384 |
| OPTN | 602432 | Glaucoma, Normal tension glaucoma, Primary open angle glaucoma | Mutant | 577 | OPTNE50K | 77.7166_77.7117_77.3789_76.6277_75.8947 | 77.7166 | 8.0385_8.0385_8.0384_8.0385_8.0385 | 8.0385 |
| OPTN | 602432 | Glaucoma, Normal tension glaucoma, Primary open angle glaucoma | Mutant | 577 | OPTNH26D | 77.9013_77.3853_77.1062_76.6908_76.1689 | 77.9013 | 7.9379_7.9474_7.9624_7.9826_7.9837 | 7.9379 |
| OPTN | 602432 | Glaucoma, Normal tension glaucoma, Primary open angle glaucoma | Mutant | 577 | OPTNL494W | 77.9578_77.5228_77.1989_76.4551_76.2259 | 77.9578 | 8.0385_8.0384_8.0385_8.0386_8.0385 | 8.0385 |
| OPTN | 602432 | Glaucoma, Normal tension glaucoma, Primary open angle glaucoma | Mutant | 577 | OPTNR329G | 77.5685_77.4619_77.1675_76.7975_75.8804 | 77.5685 | 8.0384_8.0386_8.0385_8.0386_8.0385 | 8.0384 |
| OPTN | 602432 | Glaucoma, Normal tension glaucoma, Primary open angle glaucoma | Mutant | 577 | OPTNR545Q | 77.6647_77.2662_77.0944_76.6737_75.528 | 77.6647 | 8.0384_8.0385_8.0385_8.0385_8.0385 | 8.0384 |
| OTX2 | 600037 | Microphthalmia | WT | 289 | OTX2WT | 59.7451_56.2488_55.9984_55.8584_55.8087 | 59.7451 | 8.2821_8.2998_8.3034_8.3039_8.3097 | 8.2821 |
| OTX2 | 600037 | Microphthalmia | Mutant | 289 | OTX2P133T | 59.6783_55.8287_55.8265_55.5114_55.4443 | 59.6783 | 8.2862_8.2981_8.3058_8.3043_8.3065 | 8.2862 |
| OTX2 | 600037 | Microphthalmia | Mutant | 289 | OTX2R90S | 59.5761_56.5047_55.805_55.7676_55.2034 | 59.5761 | 8.2933_8.2977_8.2926_8.3068_8.3088 | 8.2933 |
| P4HA2 | 600608 | Myopia 25, autosomal dominant | WT | 514 | P4HA2NM_004199WT | 91.047_90.7367_90.1354_89.7731_89.6225 | 91.047 | 5.4651_7.5219_7.5227_7.5228_7.5231 | 5.4651 |
| P4HA2 | 600608 | Myopia 25, autosomal dominant | Mutant | 513 | P4HA2NM_004199184delH | 90.999_90.7112_90.3984_89.1773_88.8275 | 90.999 | 7.9047_7.9051_7.9053_7.9053_7.9053 | 7.9047 |
| P4HA2 | 600608 | Myopia 25, autosomal dominant | Mutant | 514 | P4HA2NM_004199K383E | 90.7872_90.2463_89.8779_89.6357_89.5372 | 90.7872 | 5.4823_7.5221_7.5223_7.5224_7.5225 | 5.4823 |
| PANK2 | 606157 | HARP syndrome, Pantothenate kinase-associated neurodegeneration | WT | 430 | PANK2WT | 87.1253_86.6879_83.1051_82.2605_80.9153 | 87.1253 | 9.3661_9.3671_9.3662_9.3666_9.3666 | 9.3661 |
| PANK2 | 606157 | HARP syndrome, Pantothenate kinase-associated neurodegeneration | Mutant | 430 | PANK2G521R | 87.1203_86.3086_82.1227_81.5643_80.786 | 87.1203 | 8.033_8.033_8.033_8.0329_8.0329 | 8.033 |
| PANK2 | 606157 | HARP syndrome, Pantothenate kinase-associated neurodegeneration | Mutant | 430 | PANK2T528M | 86.9757_86.2804_82.646_81.3008_81.1851 | 86.9757 | 9.3664_9.3669_9.3666_9.3669_9.3665 | 9.3664 |
| PAX2 | 167409 | Papillorenal syndrome, Coloboma | WT | 394 | PAX2WT | 63.076_59.0836_58.7102_57.4976_56.7025 | 63.076 | 8.0331_8.0331_8.0332_8.0331_8.0332 | 8.0331 |
| PAX2 | 167409 | Papillorenal syndrome, Coloboma | Mutant | 389 | PAX262-66MISSING | 60.2289_56.6132_55.9672_55.1249_53.3288 | 60.2289 | 7.7812_8.0293_8.0295_8.0294_8.0297 | 7.7812 |
| PAX2 | 167409 | Papillorenal syndrome, Coloboma | Mutant | 394 | PAX2T164N | 63.097_59.1461_58.6866_57.4777_56.8223 | 63.097 | 9.4064_9.4089_9.4086_9.5214_9.5862 | 9.4064 |
| PAX3 | 606597 | Waardenburg syndrome, Craniofacial-deafness-hand syndrome | WT | 215 | PAX3WT | 75.4354_73.3075_72.4837_71.4314_67.4979 | 75.4354 | 7.9224_7.9234_7.9238_7.9248_7.9253 | 7.9224 |
| PAX3 | 606597 | Waardenburg syndrome, Craniofacial-deafness-hand syndrome | Mutant | 215 | PAX3C70R | 75.8003_73.6466_72.9911_72.0224_69.9064 | 75.8003 | 7.9231_7.9258_7.9259_7.9268_7.9265 | 7.9231 |
| PAX6 | 607108 | Eye deficit disorder, Wilms tumor, aniridia, genitourinary anomalies and mental retardation syndrome, Peters, abnormal, Foveal hypoplasia, Cataract, Symptoms resembling Alport syndrome, Aniridia, Coloboma of optic nerve | WT | 422 | PAX6WT | 68.8099_65.1317_64.3771_64.1821_63.8572 | 68.8099 | 8.0335_8.0334_8.0335_8.0335_8.0335 | 8.0335 |
| PAX6 | 607108 | Eye deficit disorder, Wilms tumor, aniridia, genitourinary anomalies and mental retardation syndrome, Peters, abnormal, Foveal hypoplasia, Cataract, Symptoms resembling Alport syndrome, Aniridia, Coloboma of optic nerve | Mutant | 422 | PAX6F258S | 68.7133_65.2028_64.0601_63.7442_63.3377 | 68.7133 | 8.0336_8.0334_8.0334_8.0335_8.0336 | 8.0336 |
| PAX6 | 607108 | Eye deficit disorder, Wilms tumor, aniridia, genitourinary anomalies and mental retardation syndrome, Peters, abnormal, Foveal hypoplasia, Cataract, Symptoms resembling Alport syndrome, Aniridia, Coloboma of optic nerve | Mutant | 422 | PAX6Q422R | 68.7893_65.2879_64.3798_63.9262_63.8933 | 68.7893 | 8.0334_8.0334_8.0334_8.0336_8.0335 | 8.0334 |
| PAX6 | 607108 | Eye deficit disorder, Wilms tumor, aniridia, genitourinary anomalies and mental retardation syndrome, Peters, abnormal, Foveal hypoplasia, Cataract, Symptoms resembling Alport syndrome, Aniridia, Coloboma of optic nerve | Mutant | 422 | PAX6S119R | 69.0259_65.2394_64.211_64.1686_63.5938 | 69.0259 | 8.81_8.81_8.81_8.81_8.81 | 8.81 |
| PCDH15 | 605514 | Usher syndrome | WT | 1714 | PCDH15WT | 72.729_71.6373_71.5416_71.3602_70.6074 | 72.729 | 8.0684_8.0762_8.0773_8.079_8.0804 | 8.0684 |
| PCDH15 | 605514 | Usher syndrome | Mutant | 1714 | PCDH15Q1342K | 72.358_71.0662_71.045_71.0205_69.4258 | 72.358 | 8.0697_8.0772_8.0775_8.0796_8.0802 | 8.0697 |
| PCDH15 | 605514 | Usher syndrome | Mutant | 1714 | PCDH15R134G | 72.7713_72.557_72.3264_72.0747_71.4997 | 72.7713 | 8.0702_8.0723_8.0736_8.0773_8.0783 | 8.0702 |
| PDE6A | 180071 | Retinitis pigmentosa | WT | 856 | PDE6AWT | 92.1077_91.2889_88.1708_87.8032_87.4469 | 92.1077 | 4.3946_8.1773_8.1775_8.1776_8.1775 | 4.3946 |
| PDE6A | 180071 | Retinitis pigmentosa | Mutant | 856 | PDE6AS573Y | 92.1119_90.7687_88.1123_88.0236_87.7771 | 92.1119 | 4.3729_8.1774_8.1776_8.1775_8.1776 | 4.3729 |
| PDE6B | 180072 | Night blindness, congenital stationary, autosomal dominant 2, Congenital static night blindness, Retinitis pigmentosa | WT | 850 | PDE6BNM_000283WT | 91.0523_90.0676_88.7754_88.6424_88.1728 | 91.0523 | 6.4313_7.7978_7.9837_8.0554_8.0604 | 6.4313 |
| PDE6B | 180072 | Night blindness, congenital stationary, autosomal dominant 2, Congenital static night blindness, Retinitis pigmentosa | Mutant | 850 | PDE6BNM_000283G407R | 91.3281_90.3387_90.0111_89.2295_89.1417 | 91.3281 | 6.4127_7.7969_7.9824_8.0558_8.0603 | 6.4127 |
| PDE6B | 180072 | Night blindness, congenital stationary, autosomal dominant 2, Congenital static night blindness, Retinitis pigmentosa | Mutant | 850 | PDE6BNM_000283H557Y | 91.4827_90.3506_89.6331_89.5182_88.7877 | 91.4827 | 6.0072_8.1804_8.1805_8.1805_8.1805 | 6.0072 |
| PDE6B | 180072 | Night blindness, congenital stationary, autosomal dominant 2, Congenital static night blindness, Retinitis pigmentosa | Mutant | 850 | PDE6BNM_000283L228H | 91.3547_90.8518_89.3431_88.7776_87.9532 | 91.3547 | 6.0077_8.1805_8.1804_8.1804_8.1804 | 6.0077 |
| PDE6B | 180072 | Night blindness, congenital stationary, autosomal dominant 2, Congenital static night blindness, Retinitis pigmentosa | Mutant | 850 | PDE6BNM_000283L87P | 91.3384_90.42_88.25_87.7756_86.3294 | 91.3384 | 6.0332_8.1804_8.1804_8.1805_8.1804 | 6.0332 |
| PDE6B | 180072 | Night blindness, congenital stationary, autosomal dominant 2, Congenital static night blindness, Retinitis pigmentosa | Mutant | 850 | PDE6BNM_000283P536S | 91.2348_90.3583_89.4567_88.8293_88.5337 | 91.2348 | 6.0319_8.1804_8.1804_8.1804_8.1805 | 6.0319 |
| PDE6C | 600827 | Cone-rod retinal dystrophy | WT | 855 | PDE6CWT | 89.6097_89.3295_88.6526_87.6998_87.3442 | 89.6097 | 4.6396_8.1784_8.1784_8.1784_8.1784 | 4.6396 |
| PDE6C | 600827 | Cone-rod retinal dystrophy | Mutant | 855 | PDE6CR29W | 89.9757_88.7733_88.4322_88.4118_88.2669 | 89.9757 | 4.6652_8.1768_8.1784_8.1785_8.1785 | 4.6652 |
| PDE6C | 600827 | Cone-rod retinal dystrophy | Mutant | 855 | PDE6CY323N | 90.3677_88.828_88.4515_88.307_87.4115 | 90.3677 | 4.6594_8.1785_8.1785_8.1785_8.1786 | 4.6594 |
| PDE6H | 601190 | Achromatopsia, Retinal cone dystrophy, Cone-rod dystrophy | WT | 83 | PDE6HWT | 69.1101_67.1361_65.5608_61.3334_60.1917 | 69.1101 | 8.3344_8.3238_8.3299_8.3336_8.3441 | 8.3344 |
| PDE6H | 601190 | Achromatopsia, Retinal cone dystrophy, Cone-rod dystrophy | Mutant | 11 | PDE6HS12X | 81.2672_80.2173_78.1706_76.1493_74.7318 | 81.2672 | 24.67_24.67_24.67_24.67_24.67 | 24.67 |
| PDZD7 | 612971 | Usher syndrome | WT | 1033 | PDZD7WT | 56.2193_53.8569_53.8043_53.7892_53.4465 | 56.2193 | 7.9629_7.9786_7.979_7.999_8.0219 | 7.9629 |
| PDZD7 | 612971 | Usher syndrome | Mutant | 1033 | PDZD7G103R | 55.9415_54.4134_53.465_52.7791_52.076 | 55.9415 | 7.9595_7.968_7.992_7.996_8.0006 | 7.9595 |
| PDZD7 | 612971 | Usher syndrome | Mutant | 1033 | PDZD7M285R | 55.6387_54.3618_54.093_53.9708_53.3133 | 55.6387 | 7.921_7.9675_7.9847_7.9827_7.9849 | 7.921 |
| PEX1 | 602136 | Heimler syndrome 1, Zellweger syndrome | WT | 1283 | PEX1WT | 66.5835_65.7661_65.7316_63.6481_63.2143 | 66.5835 | 7.8859_7.9219_7.9277_7.9279_7.928 | 7.8859 |
| PEX1 | 602136 | Heimler syndrome 1, Zellweger syndrome | Mutant | 1283 | PEX1G843D | 66.0804_64.9866_64.6982_62.9337_62.6799 | 66.0804 | 8.0668_8.0694_8.0694_8.0694_8.0694 | 8.0668 |
| PEX1 | 602136 | Heimler syndrome 1, Zellweger syndrome | Mutant | 1283 | PEX1I989T | 66.2993_65.2602_63.8832_63.8457_61.8552 | 66.2993 | 8.0659_8.0694_8.0694_8.0694_8.0694 | 8.0659 |
| PEX10 | 602859 | Zellweger syndrome | WT | 326 | PEX10WT | 82.8972_82.4508_81.317_79.392_77.6232 | 82.8972 | 8.166_8.1662_8.1661_8.1662_8.1662 | 8.166 |
| PEX10 | 602859 | Zellweger syndrome | Mutant | 326 | PEX10H290Q | 82.8326_82.1257_81.0656_79.1101_76.0633 | 82.8326 | 8.1662_8.1663_8.1662_8.1661_8.1662 | 8.1662 |
| PEX11B | 603867 | Zellweger syndrome, Peroxisome biogenesis disorder | WT | 259 | PEX11BWT | 88.3899_87.5952_87.2754_84.9843_84.5794 | 88.3899 | 7.9859_7.9872_7.9885_7.9883_7.9892 | 7.9859 |
| PEX11B | 603867 | Zellweger syndrome, Peroxisome biogenesis disorder | Mutant | 21 | PEX11BQ22X | 89.3394_85.0694_84.0135_83.175_82.3937 | 89.3394 | 11.1583_11.1583_11.1583_11.1583_11.1583 | 11.1583 |
| PEX12 | 601758 | Zellweger syndrome | WT | 359 | PEX12WT | 81.1212_78.5651_78.5516_76.9541_76.7135 | 81.1212 | 8.0033_8.0047_8.0057_8.0053_8.0176 | 8.0033 |
| PEX12 | 601758 | Zellweger syndrome | Mutant | 359 | PEX12R34S | 81.6081_79.2445_78.1501_76.9828_76.8889 | 81.6081 | 8.0034_8.0045_8.0053_8.014_8.0158 | 8.0034 |
| PEX12 | 601758 | Zellweger syndrome | Mutant | 359 | PEX12S320F | 81.6471_78.4924_78.4363_77.675_77.5549 | 81.6471 | 8.0034_8.0047_8.006_8.0094_8.0164 | 8.0034 |
| PEX13 | 601789 | Zellweger syndrome | WT | 403 | PEX13WT | 64.6106_63.4374_62.0391_61.8052_61.2942 | 64.6106 | 8.0358_8.0357_8.0358_8.0358_8.0358 | 8.0358 |
| PEX13 | 601789 | Zellweger syndrome | Mutant | 403 | PEX13I326T | 65.051_63.3112_62.5118_62.4016_61.84 | 65.051 | 9.3951_9.3974_9.4017_9.5103_9.5094 | 9.3951 |
| PEX14 | 601791 | Zellweger syndrome | WT | 376 | PEX14WT | 67.2092_63.8722_63.0644_62.9256_62.5236 | 67.2092 | 7.9107_7.912_7.9137_7.914_7.9194 | 7.9107 |
| PEX14 | 601791 | Zellweger syndrome | Mutant | 183 | PEX14Q185X | 74.5936_74.5039_73.8735_73.5801_72.6732 | 74.5936 | 7.6241_8.0333_8.0339_8.0352_8.0406 | 7.6241 |
| PEX16 | 603360 | Peroxisome biogenesis disorder 8B, Zellweger syndrome | WT | 336 | PEX16WT | 82.4578_78.8621_77.4527_76.1293_74.7565 | 82.4578 | 8.1802_8.1803_8.1802_8.1803_8.1802 | 8.1802 |
| PEX16 | 603360 | Peroxisome biogenesis disorder 8B, Zellweger syndrome | Mutant | 336 | PEX16P289T | 82.668_78.3472_76.0806_75.618_73.5667 | 82.668 | 8.1801_8.1802_8.1802_8.1803_8.1802 | 8.1801 |
| PEX16 | 603360 | Peroxisome biogenesis disorder 8B, Zellweger syndrome | Mutant | 336 | PEX16Y331C | 81.8098_76.5594_76.1681_73.666_72.574 | 81.8098 | 8.1804_8.1802_8.1802_8.1803_8.1802 | 8.1804 |
| PEX19 | 600279 | Zellweger syndrome | WT | 295 | PEX19WT | 71.2926_69.8595_67.9937_67.126_63.0445 | 71.2926 | 8.0444_8.0448_8.0451_8.044_8.0451 | 8.0444 |
| PEX19 | 600279 | Zellweger syndrome | Mutant | 117 | PEX19320delA | 72.4078_71.2568_69.6724_69.372_64.0919 | 72.4078 | 8.094_8.0944_8.0939_8.0967_8.0949 | 8.094 |
| PEX2 | 170993 | Zellweger syndrome | WT | 305 | PEX2WT | 80.4529_79.7064_79.6511_79.3572_79.133 | 80.4529 | 8.1943_8.1942_8.1942_8.1943_8.1941 | 8.1943 |
| PEX2 | 170993 | Zellweger syndrome | Mutant | 305 | PEX2E55K | 80.1438_79.9309_79.7789_79.5455_79.222 | 80.1438 | 8.1942_8.1942_8.1942_8.1942_8.1942 | 8.1942 |
| PEX26 | 608666 | Zellweger syndrome | WT | 305 | PEX26WT | 79.5267_78.2121_77.3454_77.1875_77.0553 | 79.5267 | 8.0475_8.0479_8.0489_8.0482_8.0486 | 8.0475 |
| PEX26 | 608666 | Zellweger syndrome | Mutant | 305 | PEX26L45P | 79.8155_78.7105_78.2379_77.9898_77.0756 | 79.8155 | 8.1941_8.1941_8.1943_8.1943_8.1943 | 8.1941 |
| PEX26 | 608666 | Zellweger syndrome | Mutant | 305 | PEX26R98W | 80.1132_78.4714_77.8987_77.8981_77.0798 | 80.1132 | 8.1943_8.1942_8.1943_8.1943_8.1942 | 8.1943 |
| PEX3 | 603164 | Zellweger syndrome | WT | 373 | PEX3WT | 89.366_88.9515_87.8674_87.7343_87.2123 | 89.366 | 6.32_7.772_7.7725_7.7726_7.7723 | 6.32 |
| PEX3 | 603164 | Zellweger syndrome | Mutant | 373 | PEX3G138E | 88.5189_88.4709_86.9732_86.8689_86.328 | 88.5189 | 6.2621_7.7719_7.7716_7.7724_7.7728 | 6.2621 |
| PEX3 | 603164 | Zellweger syndrome | Mutant | 373 | PEX3G331R | 89.3463_89.264_87.7263_87.6266_87.0984 | 89.3463 | 6.2961_7.7673_7.7675_7.7675_7.7676 | 6.2961 |
| PEX5 | 600414 | Rhizomelic chondrodysplasia punctata, Zellweger syndrome | WT | 631 | PEX5WT | 68.553_68.2336_67.5294_67.3005_66.5885 | 68.553 | 6.586_8.0371_8.0373_8.0374_8.0374 | 6.586 |
| PEX5 | 600414 | Rhizomelic chondrodysplasia punctata, Zellweger syndrome | Mutant | 631 | PEX5N526K | 69.1372_67.5475_66.9421_66.8934_66.4472 | 69.1372 | 6.6531_8.0357_8.0377_8.0373_8.0373 | 6.6531 |
| PEX6 | 601498 | Zellweger syndrome, Heimler syndrome 2 | WT | 980 | PEX6WT | 65.449_63.2074_63.1635_62.7235_59.8124 | 65.449 | 8.05_8.05_8.05_8.0501_8.0501 | 8.05 |
| PEX6 | 601498 | Zellweger syndrome, Heimler syndrome 2 | Mutant | 980 | PEX6P274L | 64.9473_63.3135_62.3713_61.5986_61.0682 | 64.9473 | 8.0501_8.0501_8.05_8.0501_8.0501 | 8.0501 |
| PEX6 | 601498 | Zellweger syndrome, Heimler syndrome 2 | Mutant | 980 | PEX6R601Q | 66.8043_64.1585_59.5194_59.2105_58.2138 | 66.8043 | 8.0501_8.05_8.0501_8.0501_8.0501 | 8.0501 |
| PEX7 | 601757 | Zellweger syndrome, Rhizomelic chondrodysplasia punctata | WT | 323 | PEX7WT | 94.3147_94.1188_93.923_93.6054_93.393 | 94.3147 | 8.1695_8.1695_8.1695_8.1696_8.1696 | 8.1695 |
| PEX7 | 601757 | Zellweger syndrome, Rhizomelic chondrodysplasia punctata | Mutant | 323 | PEX7A218V | 94.0247_94.0046_93.8568_93.4861_93.447 | 94.0247 | 8.1695_8.1695_8.1695_8.1696_8.1696 | 8.1695 |
| PEX7 | 601757 | Zellweger syndrome, Rhizomelic chondrodysplasia punctata | Mutant | 323 | PEX7G217R | 94.2455_94.002_93.9721_93.7886_93.6631 | 94.2455 | 8.1695_8.1696_8.1695_8.1695_8.1695 | 8.1695 |
| PGK1 | 311800 | Phosphoglycerate kinase 1 deficiency | WT | 416 | PGK1WT | 96.7643_96.6221_96.6004_95.9315_95.7132 | 96.7643 | 8.0377_8.0377_8.0378_8.0379_8.0378 | 8.0377 |
| PGK1 | 311800 | Phosphoglycerate kinase 1 deficiency | Mutant | 416 | PGK1L88P | 96.7092_96.354_96.1875_96.0252_95.5655 | 96.7092 | 8.81_8.81_8.81_8.81_8.81 | 8.81 |
| PGK1 | 311800 | Phosphoglycerate kinase 1 deficiency | Mutant | 416 | PGK1V266M | 96.4686_96.3099_96.1246_95.6925_95.3774 | 96.4686 | 8.0378_8.0378_8.0377_8.0378_8.0378 | 8.0378 |
| PHOX2A | 602753 | Fibrosis of extraocular muscles, congenital, Congenital fibrosis of the extraocular muscles, Congenital fibrosis of the extraocular muscles | WT | 284 | PHOX2AWT | 63.065_58.3274_57.9657_57.3481_57.2445 | 63.065 | 7.976_7.9764_7.9775_7.9777_7.9803 | 7.976 |
| PHOX2A | 602753 | Fibrosis of extraocular muscles, congenital, Congenital fibrosis of the extraocular muscles, Congenital fibrosis of the extraocular muscles | Mutant | 284 | PHOX2AA72V | 62.385_58.5439_58.2246_58.0433_57.5167 | 62.385 | 7.9736_7.9713_7.9754_7.9763_7.9791 | 7.9736 |
| PHYH | 602026 | Refsum disease | WT | 308 | PHYHWT | 90.7826_89.6208_89.5175_88.8503_88.2947 | 90.7826 | 8.192_8.192_8.192_8.192_8.192 | 8.192 |
| PHYH | 602026 | Refsum disease | Mutant | 308 | PHYHN269H | 90.1058_89.3069_88.992_88.631_87.5532 | 90.1058 | 8.192_8.1921_8.192_8.192_8.1921 | 8.192 |
| PHYH | 602026 | Refsum disease | Mutant | 308 | PHYHR275W | 90.2371_89.2778_88.391_87.6127_86.6339 | 90.2371 | 8.1921_8.1921_8.1921_8.1921_8.1921 | 8.1921 |
| PITPNM3 | 608921 | Cone-rod retinal dystrophy, Cone-rod dystrophy | WT | 974 | PITPNM3WT | 66.3577_65.0862_64.8582_64.5031_64.2991 | 66.3577 | 8.0427_8.0426_8.0427_8.0427_8.0427 | 8.0427 |
| PITPNM3 | 608921 | Cone-rod retinal dystrophy, Cone-rod dystrophy | Mutant | 974 | PITPNM3Q626H | 66.2882_65.1801_64.8789_64.6472_64.0894 | 66.2882 | 8.0426_8.0427_8.0426_8.0426_8.0427 | 8.0426 |
| PITX2 | 601542 | Axenfeld-Rieger syndrome, Peters, abnormal, Anterior segment dysgenesis 5, multiple subtypes, Ring dermoid of cornea | WT | 317 | PITX2WT | 60.8447_57.0024_56.4082_55.7394_54.8407 | 60.8447 | 8.1882_8.1882_8.1883_8.1882_8.1883 | 8.1882 |
| PITX2 | 601542 | Axenfeld-Rieger syndrome, Peters, abnormal, Anterior segment dysgenesis 5, multiple subtypes, Ring dermoid of cornea | Mutant | 317 | PITX2P110L | 61.0663_57.1162_56.6084_56.2819_55.8382 | 61.0663 | 8.1883_8.1883_8.1882_8.1883_8.1883 | 8.1883 |
| PITX2 | 601542 | Axenfeld-Rieger syndrome, Peters, abnormal, Anterior segment dysgenesis 5, multiple subtypes, Ring dermoid of cornea | Mutant | 317 | PITX2R136C | 60.9601_57.1429_56.8654_56.3135_55.0977 | 60.9601 | 8.1883_8.1883_8.1882_8.1884_8.1883 | 8.1883 |
| PITX3 | 602669 | Cataract 11, Congenital cataract and developmental cataract, Anterior interstitial dysplasia, Peters, abnormal, Anterior segment dysgenesis | WT | 302 | PITX3WT | 61.9159_58.7105_58.319_57.5442_57.4696 | 61.9159 | 8.1847_8.1847_8.1847_8.1848_8.1848 | 8.1847 |
| PITX3 | 602669 | Cataract 11, Congenital cataract and developmental cataract, Anterior interstitial dysplasia, Peters, abnormal, Anterior segment dysgenesis | Mutant | 302 | PITX3S13N | 61.3431_57.9182_57.7765_57.6553_57.0341 | 61.3431 | 8.1848_8.1848_8.1848_8.1848_8.1848 | 8.1848 |
| PLA2G5 | 601192 | Fleck retina, familial benign, Retinal spot | WT | 118 | PLA2G5WT | 97.1354_96.9292_96.8602_96.7265_96.6589 | 97.1354 | 7.8363_7.942_7.9433_7.9443_7.9457 | 7.8363 |
| PLA2G5 | 601192 | Fleck retina, familial benign, Retinal spot | Mutant | 118 | PLA2G5G45C | 96.7883_95.7572_95.6349_95.536_95.4737 | 96.7883 | 7.898_7.9401_7.942_7.9469_7.9498 | 7.898 |
| PLA2G5 | 601192 | Fleck retina, familial benign, Retinal spot | Mutant | 118 | PLA2G5G49S | 96.5352_96.2898_96.2677_96.2046_94.7662 | 96.5352 | 7.9897_7.9362_7.9368_7.942_7.9407 | 7.9897 |
| PNPLA6 | 603197 | Laurence-Moon syndrome, Boucher-Neuhauser syndrome | WT | 1375 | PNPLA6WT | 69.3191_69.2167_68.6255_68.5696_66.9525 | 69.3191 | 6.4949_8.176_8.176_8.176_8.1762 | 6.4949 |
| PNPLA6 | 603197 | Laurence-Moon syndrome, Boucher-Neuhauser syndrome | Mutant | 1375 | PNPLA6G578W | 68.8881_68.8763_68.5233_68.3545_67.4281 | 68.8881 | 7.9285_7.93_7.93_7.93_7.93 | 7.9285 |
| PNPLA6 | 603197 | Laurence-Moon syndrome, Boucher-Neuhauser syndrome | Mutant | 1375 | PNPLA6R1099Q | 69.682_69.3487_68.4441_68.3712_66.9664 | 69.682 | 7.9282_7.93_7.93_7.93_7.93 | 7.9282 |
| POLG | 174763 | Progressive external ophthalmoplegia, autosomal recessive 1, Progressive external ophthalmoplegia, autosomal dominant 1 | WT | 1239 | POLGWT | 79.0551_78.11_77.8309_77.0205_75.8835 | 79.0551 | 8.1656_8.1655_8.1656_8.1656_8.1657 | 8.1656 |
| POLG | 174763 | Progressive external ophthalmoplegia, autosomal recessive 1, Progressive external ophthalmoplegia, autosomal dominant 1 | Mutant | 1239 | POLGA467T | 78.4482_77.7599_77.5049_76.7496_75.4121 | 78.4482 | 8.1654_8.1655_8.1656_8.1656_8.1657 | 8.1654 |
| POLG | 174763 | Progressive external ophthalmoplegia, autosomal recessive 1, Progressive external ophthalmoplegia, autosomal dominant 1 | Mutant | 1239 | POLGW748S | 78.2018_77.885_77.4358_76.1052_75.6167 | 78.2018 | 8.1655_8.1655_8.1655_8.1655_8.1655 | 8.1655 |
| POLG | 174763 | Progressive external ophthalmoplegia, autosomal recessive 1, Progressive external ophthalmoplegia, autosomal dominant 1 | Mutant | 1239 | POLGY955C | 79.2638_78.2313_78.2235_77.6275_76.6238 | 79.2638 | 8.1656_8.1656_8.1655_8.1658_8.1657 | 8.1656 |
| POLG2 | 604983 | Progressive external ophthalmoplegia with mitochondrial DNA deletions, autosomal dominant 4 | WT | 485 | POLG2WT | 80.8088_78.9663_77.4459_77.3714_74.2443 | 80.8088 | 7.1386_9.2378_9.2386_9.2386_9.2379 | 7.1386 |
| POLG2 | 604983 | Progressive external ophthalmoplegia with mitochondrial DNA deletions, autosomal dominant 4 | Mutant | 485 | POLG2G451E | 81.324_78.6743_77.5554_76.3464_72.9962 | 81.324 | 7.1345_9.2381_9.2382_9.238_9.2379 | 7.1345 |
| POLG2 | 604983 | Progressive external ophthalmoplegia with mitochondrial DNA deletions, autosomal dominant 4 | Mutant | 485 | POLG2R182W | 80.0224_78.1586_77.4287_77.1172_74.151 | 80.0224 | 9.2282_9.2289_9.2278_9.2285_9.228 | 9.2282 |
| POMGNT1 | 606822 | Limb-girdle muscular dystrophy, Muscular dystrophy-dystroglycanopathy (congenital with mental retardation), type B, 3, Walker Warburg syndrome, Retinitis pigmentosa, Muscular dystrophy-dystroglycanopathy (congenital with mental retardation) | WT | 660 | POMGNT1NM_017739WT | 90.3069_89.6187_88.1948_88.055_87.7439 | 90.3069 | 8.0471_8.0471_8.0472_8.0472_8.0472 | 8.0471 |
| POMGNT1 | 606822 | Limb-girdle muscular dystrophy, Muscular dystrophy-dystroglycanopathy (congenital with mental retardation), type B, 3, Walker Warburg syndrome, Retinitis pigmentosa, Muscular dystrophy-dystroglycanopathy (congenital with mental retardation) | Mutant | 660 | POMGNT1NM_017739C269Y | 89.9556_89.5872_87.4967_87.2828_87.2159 | 89.9556 | 8.0431_8.047_8.0471_8.047_8.047 | 8.0431 |
| POMGNT1 | 606822 | Limb-girdle muscular dystrophy, Muscular dystrophy-dystroglycanopathy (congenital with mental retardation), type B, 3, Walker Warburg syndrome, Retinitis pigmentosa, Muscular dystrophy-dystroglycanopathy (congenital with mental retardation) | Mutant | 660 | POMGNT1NM_017739C490Y | 90.098_89.7542_87.4718_87.3012_86.8737 | 90.098 | 8.0127_8.0132_8.0133_8.0138_8.0139 | 8.0127 |
| POMGNT1 | 606822 | Limb-girdle muscular dystrophy, Muscular dystrophy-dystroglycanopathy (congenital with mental retardation), type B, 3, Walker Warburg syndrome, Retinitis pigmentosa, Muscular dystrophy-dystroglycanopathy (congenital with mental retardation) | Mutant | 660 | POMGNT1NM_017739E223K | 90.1927_89.5039_87.4421_87.219_87.1822 | 90.1927 | 8.0472_8.0472_8.0471_8.0473_8.0471 | 8.0472 |
| POMT2 | 607439 | Walker Warburg syndrome | WT | 750 | POMT2WT | 88.0968_87.5766_87.0845_86.5456_86.1652 | 88.0968 | 5.226_8.1418_8.1417_8.1435_8.1446 | 5.226 |
| POMT2 | 607439 | Walker Warburg syndrome | Mutant | 750 | POMT2G726E | 88.2852_86.9707_86.8644_86.317_85.8743 | 88.2852 | 5.3401_8.1415_8.1435_8.1466_8.1475 | 5.3401 |
| POMT2 | 607439 | Walker Warburg syndrome | Mutant | 750 | POMT2Y666C | 88.2946_87.2222_87.0747_86.9879_86.8519 | 88.2946 | 5.4992_8.1434_8.1476_8.1496_8.15 | 5.4992 |
| PPT1 | 600722 | Ceroid lipofuscinosis, neuronal | WT | 279 | PPT1WT | 95.7155_95.173_94.7756_94.5195_94.0996 | 95.7155 | 7.9368_7.9366_7.9366_7.9367_7.9366 | 7.9368 |
| PPT1 | 600722 | Ceroid lipofuscinosis, neuronal | Mutant | 279 | PPT1G108R | 95.2251_95.1876_94.8521_94.6563_94.6194 | 95.2251 | 7.9367_7.9365_7.9368_7.9368_7.9369 | 7.9367 |
| PPT1 | 600722 | Ceroid lipofuscinosis, neuronal | Mutant | 279 | PPT1L219Q | 95.1765_94.8407_94.4019_94.2466_94.1911 | 95.1765 | 8.0524_8.0523_8.0535_8.0534_8.0537 | 8.0524 |
| PRCD | 610598 | Retinitis pigmentosa | WT | 54 | PRCDWT | 63.9748_61.7744_60.5533_60.4484_59.7587 | 63.9748 | 8.0944_8.0547_8.0963_8.1334_8.0923 | 8.0944 |
| PRCD | 610598 | Retinitis pigmentosa | Mutant | 54 | PRCDC2Y | 64.7102_62.4615_62.0357_61.911_61.2276 | 64.7102 | 8.0027_8.0702_8.1122_8.0102_8.0577 | 8.0027 |
| PRCD | 610598 | Retinitis pigmentosa | Mutant | 54 | PRCDV30M | 63.7527_61.4879_61.1195_60.6854_60.4313 | 63.7527 | 8.0567_8.0956_8.1005_8.0902_8.0835 | 8.0567 |
| PRKCG | 176980 | Spinocerebellar ataxia | WT | 697 | PRKCGWT | 77.8476_75.2208_75.0_74.3978_73.2873 | 77.8476 | 8.033_8.0329_8.0329_8.033_8.033 | 8.033 |
| PRKCG | 176980 | Spinocerebellar ataxia | Mutant | 697 | PRKCGG128D | 77.6198_75.7498_75.2208_75.2175_74.4291 | 77.6198 | 7.6613_7.9399_8.0006_8.0016_8.0077 | 7.6613 |
| PRKCG | 176980 | Spinocerebellar ataxia | Mutant | 697 | PRKCGG63V | 77.1948_76.3881_75.3236_75.0702_74.5747 | 77.1948 | 7.9479_7.9479_7.9481_7.9484_7.9505 | 7.9479 |
| PROM1 | 604365 | Macular dystrophy, Cone-rod retinal dystrophy, Retinitis pigmentosa, Stargardt disease | WT | 846 | PROM1WT | 87.0263_86.6092_86.5495_86.3273_86.0717 | 87.0263 | 8.158_8.1794_8.1795_8.1796_8.1796 | 8.158 |
| PROM1 | 604365 | Macular dystrophy, Cone-rod retinal dystrophy, Retinitis pigmentosa, Stargardt disease | Mutant | 846 | PROM1R399H | 86.7122_86.5205_86.5006_86.4991_86.4521 | 86.7122 | 8.1756_8.1795_8.1794_8.1794_8.1795 | 8.1756 |
| PROM1 | 604365 | Macular dystrophy, Cone-rod retinal dystrophy, Retinitis pigmentosa, Stargardt disease | Mutant | 846 | PROM1V413D | 86.7116_86.6261_86.6066_86.5893_86.5791 | 86.7116 | 8.171_8.1795_8.1795_8.1795_8.1796 | 8.171 |
| PRPF3 | 607301 | Retinitis pigmentosa | WT | 683 | PRPF3WT | 72.1514_70.3558_69.7288_68.7461_68.7184 | 72.1514 | 8.0333_8.0333_8.0334_8.0336_8.0335 | 8.0333 |
| PRPF3 | 607301 | Retinitis pigmentosa | Mutant | 683 | PRPF3P493S | 70.9783_70.2799_70.0576_69.9087_66.908 | 70.9783 | 8.0333_8.0334_8.0334_8.0336_8.0334 | 8.0333 |
| PRPF3 | 607301 | Retinitis pigmentosa | Mutant | 683 | PRPF3T494M | 74.5535_71.058_70.3712_70.1325_68.3484 | 74.5535 | 8.0333_8.0334_8.0333_8.0334_8.0336 | 8.0333 |
| PRPF31 | 606419 | Retinitis pigmentosa | WT | 499 | PRPF31WT | 77.3111_76.7289_76.6196_76.2459_74.2574 | 77.3111 | 8.2303_8.2304_8.2303_8.2304_8.2304 | 8.2303 |
| PRPF31 | 606419 | Retinitis pigmentosa | Mutant | 196 | PRPF31E183fs | 75.3412_75.2784_72.3437_71.9685_68.8874 | 75.3412 | 7.5063_7.706_7.7072_7.715_7.7189 | 7.5063 |
| PRPF31 | 606419 | Retinitis pigmentosa | Mutant | 319 | PRPF31E268fs | 72.4057_71.6699_70.5896_70.3025_70.0173 | 72.4057 | 8.1841_8.1841_8.1842_8.1842_8.1842 | 8.1841 |
| PRPF31 | 606419 | Retinitis pigmentosa | Mutant | 499 | PRPF31G261R | 76.5881_76.0371_75.8807_75.6504_74.3718 | 76.5881 | 8.2304_8.2304_8.2303_8.2303_8.2304 | 8.2304 |
| PRPF31 | 606419 | Retinitis pigmentosa | Mutant | 499 | PRPF31Q458L | 77.1699_76.8313_76.4584_76.0171_74.5589 | 77.1699 | 8.2303_8.2304_8.2304_8.2304_8.2304 | 8.2303 |
| PRPF31 | 606419 | Retinitis pigmentosa | Mutant | 353 | PRPF31R354X | 83.4291_81.5695_80.5027_80.0803_78.1383 | 83.4291 | 8.0071_8.007_8.0071_8.0071_8.0071 | 8.0071 |
| PRPF31 | 606419 | Retinitis pigmentosa | Mutant | 499 | PRPF31R408W | 77.5631_76.9895_76.9087_76.1352_75.5521 | 77.5631 | 9.224_9.2245_9.2246_9.2245_9.2253 | 9.224 |
| PRPF6 | 613979 | Retinitis pigmentosa | WT | 941 | PRPF6WT | 79.7717_79.3508_73.8465_72.4414_72.402 | 79.7717 | 6.2087_8.0425_8.0426_8.0425_8.0426 | 6.2087 |
| PRPF6 | 613979 | Retinitis pigmentosa | Mutant | 941 | PRPF6R729W | 81.4802_81.0626_73.528_72.5531_70.8448 | 81.4802 | 6.1271_7.787_8.0054_8.0165_8.0172 | 6.1271 |
| PRPF8 | 607300 | Retinitis pigmentosa | WT | 2334 | PRPF8WT | 83.2265_81.8962_81.6141_81.552_81.4366 | 83.2265 | 7.9611_7.9611_7.9611_7.9611_7.9611 | 7.9611 |
| PRPF8 | 607300 | Retinitis pigmentosa | Mutant | 2334 | PRPF8P1540L | 85.8293_82.974_81.5698_81.2031_80.8412 | 85.8293 | 7.9611_7.9611_7.9611_7.9611_7.9611 | 7.9611 |
| PRPF8 | 607300 | Retinitis pigmentosa | Mutant | 2334 | PRPF8R2330W | 85.8597_83.883_81.5742_81.2918_81.0525 | 85.8597 | 7.9611_7.9611_7.9611_7.9611_7.9611 | 7.9611 |
| PRPH2 | 179605 | Macular dystrophy, vitelliform, Patterned macular dystrophy, Retinitis punctata albescens, Retinitis pigmentosa, Choroidal dystrophy, Leber congenital amaurosis | WT | 346 | PRPH2WT | 86.0208_85.9295_85.8151_84.1578_84.1524 | 86.0208 | nan_nan_nan_nan_nan | nan |
| PRPH2 | 179605 | Macular dystrophy, vitelliform, Patterned macular dystrophy, Retinitis punctata albescens, Retinitis pigmentosa, Choroidal dystrophy, Leber congenital amaurosis | Mutant | 346 | PRPH2P210R | 86.2808_85.9593_85.4163_84.6617_82.6927 | 86.2808 | nan_nan_nan_nan_nan | nan |
| PRPH2 | 179605 | Macular dystrophy, vitelliform, Patterned macular dystrophy, Retinitis punctata albescens, Retinitis pigmentosa, Choroidal dystrophy, Leber congenital amaurosis | Mutant | 346 | PRPH2Q178R | 86.0354_85.9049_85.2749_84.2767_82.6256 | 86.0354 | nan_nan_nan_nan_nan | nan |
| PRPH2 | 179605 | Macular dystrophy, vitelliform, Patterned macular dystrophy, Retinitis punctata albescens, Retinitis pigmentosa, Choroidal dystrophy, Leber congenital amaurosis | Mutant | 346 | PRPH2R142W | 85.0979_85.0548_83.8773_83.8132_82.1846 | 85.0979 | nan_nan_nan_nan_nan | nan |
| PRPH2 | 179605 | Macular dystrophy, vitelliform, Patterned macular dystrophy, Retinitis punctata albescens, Retinitis pigmentosa, Choroidal dystrophy, Leber congenital amaurosis | Mutant | 346 | PRPH2Y141C | 85.3435_85.1111_84.918_82.5176_80.4371 | 85.3435 | nan_nan_nan_nan_nan | nan |
| PRPS1 | 311850 | Arts syndrome | WT | 317 | PRPS1WT | 95.3851_95.1345_94.994_94.8902_94.7268 | 95.3851 | 8.1881_8.188_8.1881_8.1881_8.1881 | 8.1881 |
| PRPS1 | 311850 | Arts syndrome | Mutant | 317 | PRPS1D183H | 95.3477_94.887_94.4878_94.4579_94.2397 | 95.3477 | 8.1881_8.1881_8.1882_8.1881_8.1881 | 8.1881 |
| PRPS1 | 311850 | Arts syndrome | Mutant | 317 | PRPS1N114S | 95.479_94.9758_94.9612_94.7814_94.5443 | 95.479 | 8.188_8.188_8.1881_8.188_8.1882 | 8.188 |
| PRPS1 | 311850 | Arts syndrome | Mutant | 317 | PRPS1Q133P | 95.3199_95.0688_94.9956_94.838_94.6209 | 95.3199 | 8.1884_8.1884_8.1884_8.1883_8.1883 | 8.1884 |
| PRSS56 | 613858 | Eye deficit disorder, Microphthalmia, Microphthalmia | WT | 584 | PRSS56WT | 74.4899_73.877_72.4959_72.1871_71.674 | 74.4899 | 5.7962_8.043_8.043_8.0431_8.0431 | 5.7962 |
| PRSS56 | 613858 | Eye deficit disorder, Microphthalmia, Microphthalmia | Mutant | 584 | PRSS56C395R | 74.1901_73.4396_72.1646_71.7808_70.4407 | 74.1901 | 5.8307_8.043_8.043_8.0432_8.0433 | 5.8307 |
| PRSS56 | 613858 | Eye deficit disorder, Microphthalmia, Microphthalmia | Mutant | 584 | PRSS56G237R | 74.4018_74.0921_71.8856_71.803_70.4336 | 74.4018 | 5.7461_8.043_8.0429_8.043_8.043 | 5.7461 |
| PRSS56 | 613858 | Eye deficit disorder, Microphthalmia, Microphthalmia | Mutant | 584 | PRSS56V302F | 74.1889_73.8919_72.6503_71.6119_69.9686 | 74.1889 | 5.6247_7.9366_7.9381_7.9381_7.9389 | 5.6247 |
| RAB18 | 602207 | Warburg micro syndrome 3, Warburg micro syndrome | WT | 203 | RAB18WT | 84.5814_84.2815_83.582_83.3977_82.1132 | 84.5814 | 7.426_7.4459_7.4477_7.4513_7.4575 | 7.426 |
| RAB18 | 602207 | Warburg micro syndrome 3, Warburg micro syndrome | Mutant | 202 | RAB1893MISSING | 84.3657_84.1208_84.0241_83.2125_82.3707 | 84.3657 | 7.8864_7.8891_7.8891_7.8915_7.8913 | 7.8864 |
| RAB18 | 602207 | Warburg micro syndrome 3, Warburg micro syndrome | Mutant | 203 | RAB18L24Q | 84.8481_83.9471_83.6841_83.6384_82.7579 | 84.8481 | 7.8897_7.8894_7.8923_7.8928_7.8931 | 7.8897 |
| RAB27A | 603868 | Griscelli syndrome, Choroideremia | WT | 220 | RAB27AWT | 84.2856_83.3817_82.9008_82.2572_80.5268 | 84.2856 | 6.9116_7.9456_7.9458_7.9479_7.9474 | 6.9116 |
| RAB27A | 603868 | Griscelli syndrome, Choroideremia | Mutant | 220 | RAB27AA152P | 84.2598_82.917_82.8941_81.7804_79.9784 | 84.2598 | 7.0152_7.5636_7.565_7.5654_7.5796 | 7.0152 |
| RAB27A | 603868 | Griscelli syndrome, Choroideremia | Mutant | 220 | RAB27AW73G | 84.5572_83.9241_83.1344_81.4577_80.6399 | 84.5572 | 6.8723_7.945_7.9468_7.9462_7.9473 | 6.8723 |
| RAB3GAP1 | 602536 | Warburg micro syndrome, Warburg micro syndrome 1 | WT | 981 | RAB3GAP1WT | 77.8522_77.5209_77.2351_76.4088_75.2446 | 77.8522 | 8.0407_8.0408_8.0409_8.0408_8.0409 | 8.0407 |
| RAB3GAP1 | 602536 | Warburg micro syndrome, Warburg micro syndrome 1 | Mutant | 981 | RAB3GAP1R728A | 77.6009_77.355_76.782_76.6778_76.0306 | 77.6009 | 8.0408_8.0408_8.0408_8.0408_8.0408 | 8.0408 |
| RAB3GAP2 | 609275 | Marshall syndrome, Warburg micro syndrome 2, Warburg micro syndrome | WT | 1393 | RAB3GAP2WT | 80.3193_79.866_79.6989_79.4262_79.3949 | 80.3193 | 7.93_7.93_7.93_7.93_7.93 | 7.93 |
| RAB3GAP2 | 609275 | Marshall syndrome, Warburg micro syndrome 2, Warburg micro syndrome | Mutant | 1390 | RAB3GAP2167-169MISSING | 79.7102_79.5354_79.4208_79.1601_79.0591 | 79.7102 | 7.93_7.93_7.93_7.93_7.93 | 7.93 |
| RAB7A | 602298 | Charcot-Marie-Tooth disease, type 2B | WT | 206 | RAB7AWT | 87.751_87.6884_87.5423_86.9377_85.4765 | 87.751 | 7.9009_7.9014_7.9021_7.9022_7.903 | 7.9009 |
| RAB7A | 602298 | Charcot-Marie-Tooth disease, type 2B | Mutant | 206 | RAB7AL129F | 87.9876_87.9178_87.3448_86.7286_86.5362 | 87.9876 | 7.8998_7.9011_7.9017_7.9019_7.9019 | 7.8998 |
| RAB7A | 602298 | Charcot-Marie-Tooth disease, type 2B | Mutant | 206 | RAB7AV162M | 87.909_87.8951_87.8469_87.7518_87.1283 | 87.909 | 7.8999_7.8994_7.9007_7.902_7.9039 | 7.8999 |
| RAX | 601881 | Microphthalmia | WT | 346 | RAXWT | 62.0425_57.8935_57.2184_57.0091_56.1755 | 62.0425 | 4.8705_8.0093_8.0093_8.0093_8.0093 | 4.8705 |
| RAX | 601881 | Microphthalmia | Mutant | 346 | RAXR187Q | 62.9911_57.9924_57.3963_57.1275_57.0017 | 62.9911 | 4.8604_8.0093_8.0093_8.0093_8.0093 | 4.8604 |
| RAX | 601881 | Microphthalmia | Mutant | 346 | RAXR188Q | 63.4687_58.5123_58.0685_56.9462_56.8424 | 63.4687 | 4.9438_8.0093_8.0093_8.0093_8.0093 | 4.9438 |
| RAX | 601881 | Microphthalmia | Mutant | 346 | RAXR192Q | 62.9102_58.5246_57.5459_57.4266_57.1951 | 62.9102 | 4.9291_8.0093_8.0093_8.0093_8.0093 | 4.9291 |
| RAX | 601881 | Microphthalmia | Mutant | 346 | RAXY160H | 62.0799_58.6632_57.5613_56.9701_56.6936 | 62.0799 | 4.9189_8.0093_8.0093_8.0093_8.0093 | 4.9189 |
| RAX2 | 610362 | Macular degeneration, age-related, Cone-rod retinal dystrophy | WT | 184 | RAX2WT | 68.6043_67.5432_66.3594_65.5101_65.3221 | 68.6043 | 5.9766_8.2282_8.2309_8.2586_8.2578 | 5.9766 |
| RAX2 | 610362 | Macular degeneration, age-related, Cone-rod retinal dystrophy | Mutant | 184 | RAX2G137R | 68.9294_67.9433_66.2953_65.9603_65.574 | 68.9294 | 5.9768_8.0211_8.019_8.0251_8.0236 | 5.9768 |
| RAX2 | 610362 | Macular degeneration, age-related, Cone-rod retinal dystrophy | Mutant | 184 | RAX2R87Q | 68.7686_67.5181_66.708_66.2021_65.5811 | 68.7686 | 5.9839_8.2446_8.2475_8.2604_8.2539 | 5.9839 |
| RB1 | 614041 | Retinoblastoma | WT | 927 | RB1WT | 76.5241_75.7896_75.6793_75.6155_75.2267 | 76.5241 | 5.3614_8.0362_8.0362_8.0362_8.0362 | 5.3614 |
| RB1 | 614041 | Retinoblastoma | Mutant | 927 | RB1R661W | 76.2973_75.7918_75.6967_75.6837_75.6765 | 76.2973 | 5.5445_7.6984_8.003_8.0165_8.0169 | 5.5445 |
| RB1 | 614041 | Retinoblastoma | Mutant | 927 | RB1S567L | 76.2766_75.7255_75.3792_75.2711_74.6013 | 76.2766 | 5.4556_8.0362_8.0362_8.0362_8.0362 | 5.4556 |
| RBP3 | 180290 | Retinitis pigmentosa | WT | 1230 | RBP3WT | 85.5611_85.4029_84.6655_84.1612_83.9275 | 85.5611 | 8.1714_8.1714_8.1714_8.1714_8.1714 | 8.1714 |
| RBP3 | 180290 | Retinitis pigmentosa | Mutant | 1230 | RBP3D1080N | 84.8183_84.5375_84.3002_83.9947_83.3886 | 84.8183 | 8.1714_8.1713_8.1713_8.1714_8.1714 | 8.1714 |
| RBP4 | 180250 | Microphthalmia, Retinal dystrophy, Coloboma iridis, Coloboma, Retinitis pigmentosa | WT | 183 | RBP4WT | 94.133_93.9615_93.6723_93.3371_92.849 | 94.133 | 7.4851_8.1714_8.1736_8.1724_8.1698 | 7.4851 |
| RBP4 | 180250 | Microphthalmia, Retinal dystrophy, Coloboma iridis, Coloboma, Retinitis pigmentosa | Mutant | 183 | RBP4G93D | 93.8571_93.679_93.5966_93.2576_93.2434 | 93.8571 | 7.4288_7.8346_7.8493_7.8394_7.8388 | 7.4288 |
| RBP4 | 180250 | Microphthalmia, Retinal dystrophy, Coloboma iridis, Coloboma, Retinitis pigmentosa | Mutant | 183 | RBP4I59N | 93.7239_93.5882_93.4992_93.1666_93.0046 | 93.7239 | 7.5644_8.1653_8.1704_8.1727_8.176 | 7.5644 |
| RCBTB1 | 607867 | Retinal dystrophy | WT | 531 | RCBTB1WT | 92.4705_92.0144_91.5741_90.806_89.5795 | 92.4705 | 7.9575_7.9616_7.9601_7.9636_7.9659 | 7.9575 |
| RCBTB1 | 607867 | Retinal dystrophy | Mutant | 531 | RCBTB1L388F | 91.704_91.5797_91.2485_90.1851_90.176 | 91.704 | 7.9421_7.9425_7.9424_7.9425_7.9425 | 7.9421 |
| RCBTB1 | 607867 | Retinal dystrophy | Mutant | 531 | RCBTB1W310C | 91.9765_91.5812_91.2607_91.1704_89.4332 | 91.9765 | 7.9419_7.9419_7.9421_7.9425_7.9424 | 7.9419 |
| RD3 | 180040 | Leber congenital amaurosis 12, Leber congenital amaurosis | WT | 195 | RD3NM_183059WT | 80.6026_80.4565_80.2566_80.1849_78.6992 | 80.6026 | 8.105_8.188_8.2527_8.2494_8.2549 | 8.105 |
| RD3 | 180040 | Leber congenital amaurosis 12, Leber congenital amaurosis | Mutant | 195 | RD3NM_183059C93P | 80.5668_80.4948_80.4189_79.9094_78.658 | 80.5668 | 8.0696_8.2594_8.2611_8.2664_8.2696 | 8.0696 |
| RD3 | 180040 | Leber congenital amaurosis 12, Leber congenital amaurosis | Mutant | 195 | RD3NM_183059E32K | 79.8516_79.8426_79.6841_79.1439_78.7064 | 79.8516 | 7.8552_7.9349_7.9399_7.9429_8.2315 | 7.8552 |
| RDH12 | 608830 | Leber congenital amaurosis 13, Leber congenital amaurosis | WT | 316 | RDH12NM_152443WT | 91.1441_90.7914_90.7736_90.7557_89.8148 | 91.1441 | 8.1874_8.1874_8.1875_8.1875_8.1876 | 8.1874 |
| RDH12 | 608830 | Leber congenital amaurosis 13, Leber congenital amaurosis | Mutant | 316 | RDH12NM_152443A269G | 91.5802_91.0629_90.9169_90.1046_89.724 | 91.5802 | 8.1874_8.1874_8.1874_8.1874_8.1874 | 8.1874 |
| RDH12 | 608830 | Leber congenital amaurosis 13, Leber congenital amaurosis | Mutant | 316 | RDH12NM_152443H151D | 91.6719_91.1269_91.0471_90.4418_89.4733 | 91.6719 | 8.1874_8.1874_8.1874_8.1873_8.1874 | 8.1874 |
| RDH12 | 608830 | Leber congenital amaurosis 13, Leber congenital amaurosis | Mutant | 277 | RDH12NM_152443Q263fs | 83.7753_83.6424_82.254_82.0721_80.8084 | 83.7753 | 7.98_7.9809_7.981_7.9807_7.9815 | 7.98 |
| RDH12 | 608830 | Leber congenital amaurosis 13, Leber congenital amaurosis | Mutant | 316 | RDH12NM_152443R169G | 90.4637_90.4102_90.2635_90.15_88.4434 | 90.4637 | 8.1874_8.1874_8.1874_8.1874_8.1874 | 8.1874 |
| RDH12 | 608830 | Leber congenital amaurosis 13, Leber congenital amaurosis | Mutant | 316 | RDH12NM_152443Y226C | 91.0318_90.981_90.8865_90.564_89.2806 | 91.0318 | 8.1875_8.1876_8.1875_8.1875_8.1876 | 8.1875 |
| RDH5 | 601617 | White punctate fundus | WT | 318 | RDH5WT | 94.5185_93.5216_92.4986_92.0172_91.9374 | 94.5185 | 8.1864_8.1864_8.1864_8.1864_8.1864 | 8.1864 |
| RDH5 | 601617 | White punctate fundus | Mutant | 318 | RDH5G238W | 93.5367_93.0877_92.7576_92.7347_91.1475 | 93.5367 | 8.1866_8.1867_8.1867_8.1867_8.1866 | 8.1866 |
| RDH5 | 601617 | White punctate fundus | Mutant | 318 | RDH5R280H | 94.2821_94.2015_93.5843_93.5563_91.9998 | 94.2821 | 8.1864_8.1864_8.1864_8.1864_8.1864 | 8.1864 |
| REEP6 | 609346 | Retinitis pigmentosa 77 | WT | 211 | REEP6WT | 72.8481_72.2984_69.6753_67.5117_65.7824 | 72.8481 | 6.29_8.2333_8.2336_8.2346_8.2353 | 6.29 |
| REEP6 | 609346 | Retinitis pigmentosa 77 | Mutant | 211 | REEP6L135P | 71.7339_71.0204_70.7056_66.4765_66.0326 | 71.7339 | 6.5002_7.8988_7.901_7.9036_7.9039 | 6.5002 |
| REEP6 | 609346 | Retinitis pigmentosa 77 | Mutant | 211 | REEP6P128L | 72.2116_71.628_71.1879_68.9809_66.7089 | 72.2116 | 6.3398_8.2344_8.2356_8.2371_8.2408 | 6.3398 |
| RGR | 600342 | Retinitis pigmentosa | WT | 295 | RGRWT | 88.8321_87.8779_87.3449_87.0145_87.0003 | 88.8321 | 8.0448_8.0452_8.0451_8.0457_8.0468 | 8.0448 |
| RGR | 600342 | Retinitis pigmentosa | Mutant | 295 | RGRS66R | 88.3489_87.7441_87.4739_87.4397_86.6326 | 88.3489 | 8.0274_8.0273_8.0281_8.028_8.0282 | 8.0274 |
| RHO | 180380 | Night blindness, congenital stationary, autosomal dominant 1, Retinitis pigmentosa, Retinitis punctata albescens, Congenital static night blindness | WT | 348 | RHOWT | 89.9886_87.7759_87.6772_87.3412_86.1751 | 89.9886 | 6.249_8.0079_8.0079_8.0079_8.0079 | 6.249 |
| RHO | 180380 | Night blindness, congenital stationary, autosomal dominant 1, Retinitis pigmentosa, Retinitis punctata albescens, Congenital static night blindness | Mutant | 359 | RHOP327Hfs32X | 88.1864_86.352_86.2222_86.1447_85.0429 | 88.1864 | 8.0029_8.003_8.003_8.003_8.003 | 8.0029 |
| RHO | 180380 | Night blindness, congenital stationary, autosomal dominant 1, Retinitis pigmentosa, Retinitis punctata albescens, Congenital static night blindness | Mutant | 348 | RHOP347L | 90.169_88.3197_88.0939_87.908_86.2265 | 90.169 | 6.2555_8.0078_8.0078_8.0078_8.0078 | 6.2555 |
| RHO | 180380 | Night blindness, congenital stationary, autosomal dominant 1, Retinitis pigmentosa, Retinitis punctata albescens, Congenital static night blindness | Mutant | 348 | RHOR135W | 89.4675_87.3799_86.6984_85.3944_84.9222 | 89.4675 | 6.2564_8.0079_8.0079_8.0079_8.0079 | 6.2564 |
| RIMS1 | 606629 | Cone-rod dystrophy, Cone-rod retinal dystrophy | WT | 1692 | RIMS1WT | 50.9733_49.5838_49.1208_48.7901_48.3276 | 50.9733 | 7.9375_7.9385_7.9465_7.9497_7.9511 | 7.9375 |
| RIMS1 | 606629 | Cone-rod dystrophy, Cone-rod retinal dystrophy | Mutant | 1692 | RIMS1R820H | 51.0164_49.4026_49.1185_48.767_48.293 | 51.0164 | 7.967_7.9672_7.9672_7.9675_7.9675 | 7.967 |
| RLBP1 | 180090 | Cone-rod retinal dystrophy, Bothnia retinal dystrophy, Retinitis punctata albescens | WT | 316 | RLBP1WT | 91.6184_91.2933_91.2506_90.7769_90.4374 | 91.6184 | 8.1872_8.1873_8.1874_8.1873_8.1874 | 8.1872 |
| RLBP1 | 180090 | Cone-rod retinal dystrophy, Bothnia retinal dystrophy, Retinitis punctata albescens | Mutant | 116 | RLBP1G94fs | 72.2855_71.6159_68.9629_65.0278_60.534 | 72.2855 | 7.9483_7.9491_7.9522_7.9524_7.9505 | 7.9483 |
| ROBO3 | 608630 | Gaze palsy, familial horizontal, with progressive scoliosis, 1 | WT | 1366 | ROBO3WT | 66.6528_64.9163_63.1997_63.173_63.0862 | 66.6528 | 7.9133_7.93_7.93_7.93_7.93 | 7.9133 |
| ROBO3 | 608630 | Gaze palsy, familial horizontal, with progressive scoliosis, 1 | Mutant | 1366 | ROBO3I66L | 66.5674_64.902_63.5181_63.2573_63.2529 | 66.5674 | 7.9186_7.93_7.93_7.93_7.93 | 7.9186 |
| ROBO3 | 608630 | Gaze palsy, familial horizontal, with progressive scoliosis, 1 | Mutant | 1366 | ROBO3S705P | 66.2056_64.791_63.4141_63.0844_63.0227 | 66.2056 | 7.9126_7.93_7.93_7.93_7.93 | 7.9126 |
| ROM1 | 180721 | Retinitis pigmentosa | WT | 351 | ROM1WT | 85.1874_83.6743_82.7775_81.7555_80.2154 | 85.1874 | 8.0012_8.0012_8.0012_8.0012_8.0012 | 8.0012 |
| ROM1 | 180721 | Retinitis pigmentosa | Mutant | 351 | ROM1R229H | 85.1955_83.9668_82.9184_82.0812_79.6762 | 85.1955 | 8.0012_8.0012_8.0012_8.0013_8.0012 | 8.0012 |
| RP1 | 603937 | Retinitis pigmentosa | WT | 2156 | RP1WT | 37.5437_36.6808_36.5456_36.2311_34.6462 | 37.5437 | 7.9561_7.9561_7.9561_7.956_7.9561 | 7.9561 |
| RP1 | 603937 | Retinitis pigmentosa | Mutant | 735 | RP1D735fs | 53.0117_48.7336_48.3014_47.8852_47.16 | 53.0117 | 7.9641_7.9794_7.995_7.9964_7.9963 | 7.9641 |
| RP1 | 603937 | Retinitis pigmentosa | Mutant | 711 | RP1G706fs | 52.7894_49.145_48.5899_48.429_47.4253 | 52.7894 | 8.2348_8.2348_8.235_8.2348_8.2347 | 8.2348 |
| RP1 | 603937 | Retinitis pigmentosa | Mutant | 2156 | RP1P86T | 37.4749_36.2298_35.9437_35.7154_34.5309 | 37.4749 | 7.956_7.9561_7.9561_7.9561_7.9561 | 7.956 |
| RP1 | 603937 | Retinitis pigmentosa | Mutant | 2156 | RP1S1589F | 37.6445_36.2379_36.127_35.6557_34.4075 | 37.6445 | 7.956_7.956_7.9561_7.9561_7.9561 | 7.956 |
| RP2 | 300757 | Retinitis pigmentosa | WT | 349 | RP2WT | 91.5196_91.1625_91.1211_91.1204_90.8709 | 91.5196 | 8.0014_8.0014_8.0014_8.0014_8.0014 | 8.0014 |
| RP2 | 300757 | Retinitis pigmentosa | Mutant | 348 | RP2137missing | 92.005_91.835_90.5505_90.3769_90.2863 | 92.005 | 6.2463_8.0078_8.0078_8.0078_8.0078 | 6.2463 |
| RP2 | 300757 | Retinitis pigmentosa | Mutant | 348 | RP26missing | 92.6117_92.2233_91.4219_91.3342_91.1739 | 92.6117 | 6.3237_8.0078_8.0078_8.0078_8.0078 | 6.3237 |
| RP2 | 300757 | Retinitis pigmentosa | Mutant | 349 | RP2E138G | 92.4564_92.3576_91.1535_91.1243_90.8702 | 92.4564 | 8.0013_8.0013_8.0013_8.0013_8.0013 | 8.0013 |
| RP2 | 300757 | Retinitis pigmentosa | Mutant | 349 | RP2R118H | 92.4112_92.1644_91.2048_91.0263_90.9603 | 92.4112 | 8.0013_8.0013_8.0013_8.0013_8.0014 | 8.0013 |
| RP9 | 607331 | Retinitis pigmentosa | WT | 221 | RP9WT | 76.4222_76.0575_75.9309_73.5333_72.673 | 76.4222 | 6.8948_7.9015_7.9029_7.905_8.2489 | 6.8948 |
| RP9 | 607331 | Retinitis pigmentosa | Mutant | 221 | RP9D170G | 76.3833_76.0277_75.7278_73.6231_72.7852 | 76.3833 | 6.6882_7.9419_7.9424_7.9423_7.9438 | 6.6882 |
| RP9 | 607331 | Retinitis pigmentosa | Mutant | 221 | RP9E138K | 76.3306_76.0952_75.8573_73.5838_73.5619 | 76.3306 | 6.5071_8.2497_8.2517_8.2528_8.2537 | 6.5071 |
| RP9 | 607331 | Retinitis pigmentosa | Mutant | 221 | RP9H137L | 76.5253_75.9319_75.5397_73.2914_72.7832 | 76.5253 | 6.9296_7.8822_7.9403_7.9415_7.9424 | 6.9296 |
| RPE65 | 180069 | Retinitis pigmentosa, Leber congenital amaurosis, Leber congenital amaurosis 2 | WT | 532 | RPE65NM_000329WT | 95.5105_95.3802_91.4629_90.7065_90.6455 | 95.5105 | 7.8875_7.888_7.8886_7.8884_7.8889 | 7.8875 |
| RPE65 | 180069 | Retinitis pigmentosa, Leber congenital amaurosis, Leber congenital amaurosis 2 | Mutant | 532 | RPE65NM_000329D477G | 95.4598_95.3511_91.9217_91.3424_90.8301 | 95.4598 | 7.8883_7.8888_7.8886_7.8887_7.8887 | 7.8883 |
| RPE65 | 180069 | Retinitis pigmentosa, Leber congenital amaurosis, Leber congenital amaurosis 2 | Mutant | 532 | RPE65NM_000329R91W | 95.3648_95.1792_91.1207_89.8191_89.2034 | 95.3648 | 7.8876_7.8881_7.8881_7.8881_7.8885 | 7.8876 |
| RPE65 | 180069 | Retinitis pigmentosa, Leber congenital amaurosis, Leber congenital amaurosis 2 | Mutant | 532 | RPE65NM_000329T385M | 95.3726_95.3427_91.3723_91.2363_90.7108 | 95.3726 | 7.8876_7.8878_7.8887_7.8885_7.8888 | 7.8876 |
| RPGR | 312610 | Cone-rod dystrophy, X-linked, 1, Macular degeneration, Cone-rod retinal dystrophy, Retinitis pigmentosa | WT | 815 | RPGRNM_00328WT | 63.2712_61.556_58.836_57.893_57.7697 | 63.2712 | 8.0344_8.0346_8.0346_8.0346_8.0346 | 8.0344 |
| RPGR | 312610 | Cone-rod dystrophy, X-linked, 1, Macular degeneration, Cone-rod retinal dystrophy, Retinitis pigmentosa | Mutant | 815 | RPGRNM_00328A110V | 62.5431_61.4006_59.1139_59.0934_57.8755 | 62.5431 | 7.9224_7.9242_7.9248_7.925_7.9254 | 7.9224 |
| RPGR | 312610 | Cone-rod dystrophy, X-linked, 1, Macular degeneration, Cone-rod retinal dystrophy, Retinitis pigmentosa | Mutant | 130 | RPGRNM_00328E125fs | 95.5005_94.4903_90.1544_89.9414_89.9128 | 95.5005 | 8.0036_8.0071_8.0071_8.0071_8.0145 | 8.0036 |
| RPGR | 312610 | Cone-rod dystrophy, X-linked, 1, Macular degeneration, Cone-rod retinal dystrophy, Retinitis pigmentosa | Mutant | 815 | RPGRNM_00328G436D | 63.0876_61.9377_59.5227_58.2505_57.4309 | 63.0876 | 8.0344_8.0345_8.0345_8.0346_8.0345 | 8.0344 |
| RPGR | 312610 | Cone-rod dystrophy, X-linked, 1, Macular degeneration, Cone-rod retinal dystrophy, Retinitis pigmentosa | Mutant | 815 | RPGRNM_00328H98Q | 62.9883_62.165_59.6407_58.6192_58.6054 | 62.9883 | 8.0345_8.0347_8.0346_8.0347_8.0346 | 8.0345 |
| RPGRIP1 | 605446 | Cone-rod dystrophy, Cone-rod retinal dystrophy, Leber congenital amaurosis, Leber congenital amaurosis 6 | WT | 1286 | RPGRIP1NM_020366WT | 66.9062_66.2756_66.2385_65.9022_65.8486 | 66.9062 | 8.0682_8.0689_8.0689_8.0695_8.0696 | 8.0682 |
| RPGRIP1 | 605446 | Cone-rod dystrophy, Cone-rod retinal dystrophy, Leber congenital amaurosis, Leber congenital amaurosis 6 | Mutant | 1286 | RPGRIP1NM_020366D1114G | 67.2004_67.0373_66.5074_66.0708_65.4706 | 67.2004 | 8.0684_8.0689_8.0695_8.0696_8.0696 | 8.0684 |
| RPGRIP1 | 605446 | Cone-rod dystrophy, Cone-rod retinal dystrophy, Leber congenital amaurosis, Leber congenital amaurosis 6 | Mutant | 1286 | RPGRIP1NM_020366V1211E | 67.4869_66.9473_66.0543_65.9811_65.8822 | 67.4869 | 8.0678_8.069_8.0696_8.0695_8.0696 | 8.0678 |
| RRM2B | 604712 | Progressive external ophthalmoplegia with mitochondrial DNA deletions, autosomal dominant 5 | WT | 351 | RRM2BWT | 85.1488_84.8733_83.6843_83.3501_83.2465 | 85.1488 | 8.0013_8.0013_8.0013_8.0013_8.0013 | 8.0013 |
| RRM2B | 604712 | Progressive external ophthalmoplegia with mitochondrial DNA deletions, autosomal dominant 5 | Mutant | 350 | RRM2B85MISSING | 83.9845_83.8179_82.617_82.5899_81.3373 | 83.9845 | 8.0019_8.0018_8.0018_8.0019_8.0019 | 8.0019 |
| RRM2B | 604712 | Progressive external ophthalmoplegia with mitochondrial DNA deletions, autosomal dominant 5 | Mutant | 351 | RRM2BR121H | 84.1688_83.9527_83.6688_83.5246_83.1456 | 84.1688 | 8.0013_8.0013_8.0013_8.0013_8.0013 | 8.0013 |
| RS1 | 300839 | Retinoschisis | WT | 201 | RS1WT | 72.8645_72.6447_72.0657_67.8287_62.7363 | 72.8645 | 6.9268_7.4141_7.4147_7.4273_7.4271 | 6.9268 |
| RS1 | 300839 | Retinoschisis | Mutant | 201 | RS1E72K | 71.7266_69.4583_67.642_66.8774_62.7689 | 71.7266 | 7.1347_7.4199_7.4285_7.4286_7.4299 | 7.1347 |
| RS1 | 300839 | Retinoschisis | Mutant | 201 | RS1R200C | 74.8974_73.0288_69.6463_68.4837_64.6506 | 74.8974 | 7.021_7.4291_7.433_7.4353_7.4377 | 7.021 |
| RTN4IP1 | 610502 | Optic atrophy 10 | WT | 356 | RTN4IP1WT | 96.7544_96.6777_95.9874_95.8696_95.8168 | 96.7544 | 8.0031_8.0031_8.0032_8.0032_8.0031 | 8.0031 |
| RTN4IP1 | 610502 | Optic atrophy 10 | Mutant | 356 | RTN4IP1R103H | 95.9324_95.2125_95.1728_94.9625_94.7567 | 95.9324 | 8.0029_8.0029_8.0029_8.0029_8.0029 | 8.0029 |
| SCO2 | 604272 | Myopia | WT | 225 | SCO2NM_005138WT | 89.9166_89.1912_88.7438_88.6847_85.2923 | 89.9166 | 2.8805_2.7789_2.8465_2.7206_2.7396 | 2.8805 |
| SCO2 | 604272 | Myopia | Mutant | 225 | SCO2NM_005138E140K | 90.2079_89.5032_88.1168_87.8148_85.6716 | 90.2079 | 7.7217_7.7192_7.7257_7.7263_7.7259 | 7.7217 |
| SCO2 | 604272 | Myopia | Mutant | 225 | SCO2NM_005138R120W | 90.2182_89.591_89.2751_88.098_86.1027 | 90.2182 | 7.9602_7.9616_7.9619_7.9621_7.9618 | 7.9602 |
| SDHA | 600857 | Leigh Syndrome | WT | 622 | SDHAWT | 97.4867_97.1982_95.3559_95.0826_94.6325 | 97.4867 | 8.0296_8.0297_8.0296_8.0295_8.0297 | 8.0296 |
| SDHA | 600857 | Leigh Syndrome | Mutant | 622 | SDHAC189G | 97.5163_97.2978_95.5855_95.1242_94.9006 | 97.5163 | 8.0295_8.0295_8.0296_8.0296_8.0296 | 8.0295 |
| SDHA | 600857 | Leigh Syndrome | Mutant | 622 | SDHAG555E | 97.5346_97.1275_95.383_95.1259_94.8647 | 97.5346 | 8.0296_8.0295_8.0297_8.0296_8.0296 | 8.0296 |
| SDHA | 600857 | Leigh Syndrome | Mutant | 622 | SDHAR589W | 97.3523_97.2517_94.6932_94.6092_94.4432 | 97.3523 | 8.0296_8.0296_8.0296_8.0296_8.0297 | 8.0296 |
| SEMA4A | 607292 | Cone-rod dystrophy, Retinitis pigmentosa, Cone-rod retinal dystrophy | WT | 729 | SEMA4AWT | 85.5222_85.3073_85.0909_85.0707_84.9281 | 85.5222 | 7.4187_8.0301_8.031_8.0313_8.0315 | 7.4187 |
| SEMA4A | 607292 | Cone-rod dystrophy, Retinitis pigmentosa, Cone-rod retinal dystrophy | Mutant | 729 | SEMA4AR510Q | 85.4182_85.3912_85.0509_84.9025_84.4985 | 85.4182 | 7.4769_7.9576_7.9583_7.9589_7.9591 | 7.4769 |
| SEMA4A | 607292 | Cone-rod dystrophy, Retinitis pigmentosa, Cone-rod retinal dystrophy | Mutant | 729 | SEMA4AT405M | 85.3628_85.3595_85.2683_85.2083_84.6086 | 85.3628 | 7.3554_8.0294_8.0296_8.0297_8.0308 | 7.3554 |
| SEMA4A | 607292 | Cone-rod dystrophy, Retinitis pigmentosa, Cone-rod retinal dystrophy | Mutant | 729 | SEMA4AV78M | 85.4234_85.4193_85.3877_85.3835_84.788 | 85.4234 | 7.5307_8.0291_8.0293_8.0304_8.0306 | 7.5307 |
| SEMA4A | 607292 | Cone-rod dystrophy, Retinitis pigmentosa, Cone-rod retinal dystrophy | Mutant | 729 | SEMA4AY244C | 85.2317_85.181_85.1545_84.8763_84.6877 | 85.2317 | 7.4663_8.0284_8.0286_8.0305_8.0309 | 7.4663 |
| SGCA | 600119 | Muscular dystrophy, limb-girdle | WT | 364 | SGCAWT | 81.3299_81.128_81.0572_80.2667_78.8363 | 81.3299 | 6.0209_7.7078_7.709_7.7097_7.7108 | 6.0209 |
| SGCA | 600119 | Muscular dystrophy, limb-girdle | Mutant | 364 | SGCAL173P | 81.0338_80.842_80.464_79.6515_78.1274 | 81.0338 | 6.1271_7.7036_7.7067_7.7078_7.7086 | 6.1271 |
| SGCA | 600119 | Muscular dystrophy, limb-girdle | Mutant | 364 | SGCAR77C | 81.5051_81.1867_80.9643_79.9932_79.0484 | 81.5051 | 6.019_7.7051_7.7068_7.7091_7.7087 | 6.019 |
| SGCB | 600900 | Limb-girdle muscular dystrophy | WT | 318 | SGCBWT | 77.3464_76.8564_76.4724_74.8101_74.7897 | 77.3464 | 8.1863_8.1864_8.1864_8.1864_8.1864 | 8.1863 |
| SGCB | 600900 | Limb-girdle muscular dystrophy | Mutant | 37 | SGCB38-318MISSING | 72.5627_71.8789_67.4874_67.1821_65.7395 | 72.5627 | 6.5886_7.6166_8.7255_8.3147_8.1081 | 6.5886 |
| SGCB | 600900 | Limb-girdle muscular dystrophy | Mutant | 318 | SGCBG167S | 78.0266_76.7507_76.4198_76.1062_75.9139 | 78.0266 | 8.1864_8.1865_8.1864_8.1866_8.1864 | 8.1864 |
| SGCB | 600900 | Limb-girdle muscular dystrophy | Mutant | 318 | SGCBS114F | 78.0348_77.006_75.6817_75.668_75.5983 | 78.0348 | 8.1865_8.1865_8.1865_8.1865_8.1866 | 8.1865 |
| SGCD | 601411 | Macular degeneration | WT | 289 | SGCDWT | 83.2601_82.7458_81.5532_81.2319_80.2288 | 83.2601 | 8.3_8.3075_8.3082_8.3088_8.3121 | 8.3 |
| SGCD | 601411 | Macular degeneration | Mutant | 289 | SGCDE261K | 82.0364_80.7946_80.6815_80.5795_79.295 | 82.0364 | 8.2992_8.3047_8.3012_8.3049_8.3049 | 8.2992 |
| SGCD | 601411 | Macular degeneration | Mutant | 289 | SGCDS150A | 82.7906_82.6024_81.5524_80.7686_80.0869 | 82.7906 | 8.3061_8.306_8.3087_8.3109_8.3076 | 8.3061 |
| SGCG | 608896 | Muscular dystrophy, limb-girdle | WT | 291 | SGCGWT | 80.8649_80.7825_79.9306_79.7806_78.7059 | 80.8649 | 8.249_8.2501_8.2554_8.2551_8.2587 | 8.249 |
| SGCG | 608896 | Muscular dystrophy, limb-girdle | Mutant | 291 | SGCGC283Y | 79.8176_78.9226_78.1888_77.8132_76.9977 | 79.8176 | 8.2305_8.2381_8.2418_8.2447_8.2454 | 8.2305 |
| SGCG | 608896 | Muscular dystrophy, limb-girdle | Mutant | 291 | SGCGL71S | 80.6436_80.4567_79.3592_78.5273_77.4348 | 80.6436 | 8.2449_8.2507_8.2476_8.2528_8.2515 | 8.2449 |
| SHH | 600725 | Microphthalmia, Eye deficit disorder | WT | 439 | SHHWT | 78.6082_78.5688_78.2264_78.1722_77.4067 | 78.6082 | 8.0386_8.0387_8.0387_8.0388_8.0389 | 8.0386 |
| SHH | 600725 | Microphthalmia, Eye deficit disorder | Mutant | 439 | SHHE188Q | 78.5284_78.4902_78.2232_77.9353_77.4625 | 78.5284 | 8.0388_8.0389_8.0389_8.0389_8.0388 | 8.0388 |
| SHH | 600725 | Microphthalmia, Eye deficit disorder | Mutant | 439 | SHHQ100H | 79.0264_78.7276_78.6089_78.3975_78.3489 | 79.0264 | 8.0388_8.0388_8.0389_8.039_8.0388 | 8.0388 |
| SHH | 600725 | Microphthalmia, Eye deficit disorder | Mutant | 439 | SHHW117R | 78.2354_78.1958_77.5447_77.3747_76.9838 | 78.2354 | 9.3469_9.3435_9.3445_9.3442_9.3451 | 9.3469 |
| SIL1 | 608005 | Nail - patella syndrome | WT | 430 | SIL1WT | 83.3592_83.1557_82.6249_82.5452_82.0149 | 83.3592 | 8.0325_8.0327_8.0328_8.0327_8.0327 | 8.0325 |
| SIL1 | 608005 | Nail - patella syndrome | Mutant | 430 | SIL1G312R | 83.1816_83.124_82.6195_82.5363_81.6639 | 83.1816 | 9.3582_9.3597_9.3591_9.3605_9.36 | 9.3582 |
| SIL1 | 608005 | Nail - patella syndrome | Mutant | 430 | SIL1L457P | 83.5342_82.8172_82.7521_82.4857_81.9166 | 83.5342 | 9.3596_9.3596_9.3589_9.3597_9.3596 | 9.3596 |
| SIL1 | 608005 | Nail - patella syndrome | Mutant | 428 | SIL1V231_I232DEL | 81.6961_81.3818_81.1908_81.0091_80.4191 | 81.6961 | 9.3816_9.3811_9.3815_9.381_9.3817 | 9.3816 |
| SIPA1L3 | 616655 | Cataract 45 | WT | 1781 | SIPA1L3WT | 56.0649_55.5116_54.2537_54.1272_53.2321 | 56.0649 | 7.9119_7.9201_7.9217_7.9321_7.9517 | 7.9119 |
| SIPA1L3 | 616655 | Cataract 45 | Mutant | 1496 | SIPA1L3R1497X | 58.5217_58.1585_56.0384_55.8359_54.5879 | 58.5217 | 8.0437_8.0499_8.0559_8.0595_8.0799 | 8.0437 |
| SIX5 | 600963 | Branchiootorenal syndrome | WT | 739 | SIX5WT | 50.7708_48.7057_47.2425_47.0919_46.6024 | 50.7708 | 7.9659_7.9771_7.982_7.9833_7.9865 | 7.9659 |
| SIX5 | 600963 | Branchiootorenal syndrome | Mutant | 739 | SIX5A296T | 50.7783_48.4328_46.9181_46.8388_46.249 | 50.7783 | 7.8984_7.9191_7.935_7.9367_7.9378 | 7.8984 |
| SIX5 | 600963 | Branchiootorenal syndrome | Mutant | 739 | SIX5T552M | 50.6604_48.4361_46.6976_46.5145_46.4989 | 50.6604 | 7.9091_7.9194_7.9287_7.9403_8.1346 | 7.9091 |
| SIX6 | 606326 | Oculoauricular syndrome, Macular dystrophy, Optic disc anomalies with retinal and/or macular dystrophy, Fraser syndrome | WT | 246 | SIX6WT | 80.1773_79.2072_78.7843_78.2139_78.0131 | 80.1773 | 7.9116_7.916_7.9162_7.9191_7.9194 | 7.9116 |
| SIX6 | 606326 | Oculoauricular syndrome, Macular dystrophy, Optic disc anomalies with retinal and/or macular dystrophy, Fraser syndrome | Mutant | 261 | SIX6Asn178ProfsTer142 | 69.6373_67.9617_67.7362_67.367_66.6141 | 69.6373 | 7.9024_7.9066_7.9088_7.9178_7.9174 | 7.9024 |
| SIX6 | 606326 | Oculoauricular syndrome, Macular dystrophy, Optic disc anomalies with retinal and/or macular dystrophy, Fraser syndrome | Mutant | 246 | SIX6H141N | 79.0649_78.7995_78.5805_78.1883_77.8126 | 79.0649 | 7.9922_7.9925_7.9932_7.9932_7.9934 | 7.9922 |
| SLC16A12 | 611910 | Diabetes, Corneal abnormality syndrome | WT | 516 | SLC16A12WT | 80.0246_79.4412_78.3457_78.2682_77.3019 | 80.0246 | 8.0194_7.9402_7.9404_7.9406_7.9408 | 8.0194 |
| SLC24A1 | 603617 | Congenital static night blindness, Congenital static night blindness, 1D | WT | 1099 | SLC24A1NM_004727WT | 54.2951_51.4313_50.9644_50.3782_48.0287 | 54.2951 | 10.1846_10.1847_10.1847_10.1848_10.1851 | 10.1846 |
| SLC24A1 | 603617 | Congenital static night blindness, Congenital static night blindness, 1D | Mutant | 559 | SLC24A1NM_004727F538CfsX23 | 47.4866_43.2164_42.7778_42.6595_42.437 | 47.4866 | 8.0312_8.0313_8.0313_8.0314_8.0312 | 8.0312 |
| SLC25A15 | 603861 | Hyperornithinemia-hyperammonemia-homocitrullinemia syndrome | WT | 301 | SLC25A15WT | 86.3692_85.4153_80.3104_78.7254_78.2435 | 86.3692 | 8.1865_8.1864_8.1864_8.1865_8.1865 | 8.1865 |
| SLC25A15 | 603861 | Hyperornithinemia-hyperammonemia-homocitrullinemia syndrome | Mutant | 300 | SLC25A15188MISSING | 85.4195_82.904_79.3422_75.7249_72.124 | 85.4195 | 8.1875_8.1876_8.1875_8.1876_8.1875 | 8.1875 |
| SLC25A15 | 603861 | Hyperornithinemia-hyperammonemia-homocitrullinemia syndrome | Mutant | 301 | SLC25A15G27R | 85.538_85.3263_77.0365_75.0207_74.7377 | 85.538 | 8.1865_8.1864_8.1865_8.1864_8.1864 | 8.1865 |
| SLC25A4 | 103220 | Progressive external ophthalmoplegia with mitochondrial DNA deletions, autosomal dominant 2 | WT | 297 | SLC25A4WT | 93.0389_92.5628_91.3116_89.6194_89.5452 | 93.0389 | 8.1929_8.1929_8.193_8.1931_8.193 | 8.1929 |
| SLC25A4 | 103220 | Progressive external ophthalmoplegia with mitochondrial DNA deletions, autosomal dominant 2 | Mutant | 297 | SLC25A4A114P | 92.9133_92.5003_90.007_89.8144_89.3162 | 92.9133 | 8.1929_8.1929_8.193_8.193_8.193 | 8.1929 |
| SLC25A4 | 103220 | Progressive external ophthalmoplegia with mitochondrial DNA deletions, autosomal dominant 2 | Mutant | 297 | SLC25A4V289M | 92.9743_92.8635_91.9767_91.6947_89.7772 | 92.9743 | 8.1928_8.1931_8.1929_8.193_8.193 | 8.1928 |
| SLC26A4 | 605646 | Retinitis pigmentosa | WT | 780 | SLC26A4WT | 82.1807_82.0479_81.3108_80.5177_79.987 | 82.1807 | 6.3445_7.7234_7.9784_8.061_8.0716 | 6.3445 |
| SLC26A4 | 605646 | Retinitis pigmentosa | Mutant | 780 | SLC26A4T416P | 82.2863_81.9615_81.3718_80.7603_79.7036 | 82.2863 | 6.5718_7.94_7.94_7.94_7.94 | 6.5718 |
| SLC26A4 | 605646 | Retinitis pigmentosa | Mutant | 780 | SLC26A4V138F | 82.6008_82.2764_80.9728_80.7552_80.3082 | 82.6008 | 6.516_7.7414_7.9659_8.0585_8.0717 | 6.516 |
| SLC2A1 | 138140 | Stomatin-deficient cryohydrocytosis with neurologic defects | WT | 492 | SLC2A1WT | 88.5093_87.9843_87.7617_87.3122_84.9122 | 88.5093 | 9.2343_9.2434_9.2434_9.2438_9.2434 | 9.2343 |
| SLC2A1 | 138140 | Stomatin-deficient cryohydrocytosis with neurologic defects | Mutant | 492 | SLC2A1R126H | 89.3773_88.5869_88.3251_88.0874_87.6825 | 89.3773 | 9.233_9.2332_9.2336_9.2337_9.234 | 9.233 |
| SLC2A1 | 138140 | Stomatin-deficient cryohydrocytosis with neurologic defects | Mutant | 492 | SLC2A1R333W | 89.1446_87.9691_87.4125_87.0784_84.8885 | 89.1446 | 8.0416_8.0417_8.0417_8.0416_8.0417 | 8.0416 |
| SLC33A1 | 603690 | Syndromic cataract | WT | 549 | SLC33A1WT | 82.2687_82.2274_82.1606_81.1277_81.0592 | 82.2687 | 6.3545_7.9368_7.9374_7.9374_7.9377 | 6.3545 |
| SLC33A1 | 603690 | Syndromic cataract | Mutant | 549 | SLC33A1A110P | 81.3762_81.2381_80.9342_80.6987_79.8801 | 81.3762 | 6.484_8.0334_8.0334_8.0334_8.0334 | 6.484 |
| SLC4A11 | 610206 | Corneal dystrophy, Corneal dystrophy, Fuchs endothelial, 4, Corneal endothelial dystrophy and perceptive deafness, Corneal endothelial dystrophy, Corneal endothelial dystrophy, autosomal recessive | WT | 891 | SLC4A11WT | 73.5396_72.3874_72.2864_72.0518_71.8072 | 73.5396 | 8.041_8.0432_8.0433_8.0446_8.0452 | 8.041 |
| SLC4A11 | 610206 | Corneal dystrophy, Corneal dystrophy, Fuchs endothelial, 4, Corneal endothelial dystrophy and perceptive deafness, Corneal endothelial dystrophy, Corneal endothelial dystrophy, autosomal recessive | Mutant | 891 | SLC4A11C386R | 73.195_73.0498_72.1119_72.0115_70.5104 | 73.195 | 8.0405_8.0447_8.0446_8.0446_8.0459 | 8.0405 |
| SLC4A11 | 610206 | Corneal dystrophy, Corneal dystrophy, Fuchs endothelial, 4, Corneal endothelial dystrophy and perceptive deafness, Corneal endothelial dystrophy, Corneal endothelial dystrophy, autosomal recessive | Mutant | 891 | SLC4A11R755W | 73.5641_72.9154_72.1946_71.397_70.2631 | 73.5641 | 8.043_8.0436_8.0438_8.047_8.0466 | 8.043 |
| SLC9A6 | 300231 | Intellectual developmental disorder, X-linked syndromic, Christianson type | WT | 669 | SLC9A6WT | 70.1488_70.0469_69.4251_69.1174_67.7588 | 70.1488 | 8.034_8.0342_8.0343_8.0342_8.0343 | 8.034 |
| SLC9A6 | 300231 | Intellectual developmental disorder, X-linked syndromic, Christianson type | Mutant | 669 | SLC9A6G186R | 70.2125_69.5604_69.5136_69.4379_68.424 | 70.2125 | 8.0343_8.0343_8.0343_8.0343_8.0342 | 8.0343 |
| SLITRK6 | 609681 | Myopic syndrome | WT | 815 | SLITRK6NM_032229WT | 69.6995_68.2445_68.0993_67.9266_67.9004 | 69.6995 | 8.0344_8.0346_8.0346_8.0345_8.0346 | 8.0344 |
| SLITRK6 | 609681 | Myopic syndrome | Mutant | 387 | SLITRK6NM_032229Q414X | 84.098_83.6795_82.8432_82.6967_82.5693 | 84.098 | 7.0509_8.0312_8.0312_8.0313_8.0313 | 7.0509 |
| SMOC1 | 608488 | Microphthalmia with limb anomalies | WT | 408 | SMOC1WT | 74.5779_71.1136_71.0983_71.0355_70.1057 | 74.5779 | 9.4126_9.4117_9.4134_9.4142_9.4132 | 9.4126 |
| SMOC1 | 608488 | Microphthalmia with limb anomalies | Mutant | 408 | SMOC1R278C | 73.9788_71.3551_69.7703_69.4019_69.2368 | 73.9788 | 8.043_8.0431_8.0429_8.043_8.0432 | 8.043 |
| SMOC1 | 608488 | Microphthalmia with limb anomalies | Mutant | 408 | SMOC1R286H | 73.1941_70.5855_70.4834_70.0156_69.5286 | 73.1941 | 9.4134_9.4111_9.4113_9.4122_9.4143 | 9.4134 |
| SMOC1 | 608488 | Microphthalmia with limb anomalies | Mutant | 408 | SMOC1T283N | 74.1131_70.7078_70.4317_69.9497_69.3939 | 74.1131 | 8.043_8.043_8.0431_8.0432_8.0431 | 8.043 |
| SMS | 300105 | Myopia | WT | 365 | SMSNM_004595WT | 94.5709_94.527_93.6628_93.032_92.7461 | 94.5709 | 7.1791_8.1996_8.2002_8.2002_8.2003 | 7.1791 |
| SMS | 300105 | Myopia | Mutant | 365 | SMSNM_004595G67E | 94.5816_94.5738_94.1836_93.4408_93.3708 | 94.5816 | 7.213_8.1996_8.2_8.1999_8.1995 | 7.213 |
| SMS | 300105 | Myopia | Mutant | 365 | SMSNM_004595Y328C | 94.3969_94.3248_93.4834_93.1587_92.9444 | 94.3969 | 7.1866_8.2004_8.2007_8.2007_8.2012 | 7.1866 |
| SNRNP200 | 601664 | Retinitis pigmentosa | WT | 2136 | SNRNP200WT | 83.4207_82.0134_81.0799_80.8983_80.4636 | 83.4207 | 7.9235_7.9276_7.9283_7.9287_7.9289 | 7.9235 |
| SNRNP200 | 601664 | Retinitis pigmentosa | Mutant | 2136 | SNRNP200H805P | 85.5325_84.1592_81.0483_79.9659_79.7147 | 85.5325 | 7.9288_7.9289_7.9289_7.9289_7.9289 | 7.9288 |
| SNRNP200 | 601664 | Retinitis pigmentosa | Mutant | 2136 | SNRNP200S1087L | 85.5937_84.8002_80.3557_80.3496_79.9475 | 85.5937 | 7.9277_7.9286_7.9288_7.9289_7.9288 | 7.9277 |
| SOD1 | 147450 | Macular degeneration | WT | 153 | SOD1WT | 98.1903_98.1888_97.8688_97.8087_97.7651 | 98.1903 | 7.8686_7.8635_7.8544_7.8677_7.8658 | 7.8686 |
| SOD1 | 147450 | Macular degeneration | Mutant | 153 | SOD1G86R | 98.0786_98.0114_97.7287_97.6552_97.6154 | 98.0786 | 8.0031_8.0152_8.0127_8.0094_8.0084 | 8.0031 |
| SOD1 | 147450 | Macular degeneration | Mutant | 153 | SOD1I114T | 98.2938_98.2835_97.8405_97.8107_97.7353 | 98.2938 | 7.8635_7.8704_7.8636_7.8708_7.8709 | 7.8635 |
| SOX10 | 602229 | PCWH syndrome, Waardenburg syndrome | WT | 466 | SOX10WT | 56.5789_53.5128_52.9019_51.8722_51.1468 | 56.5789 | 9.4736_9.4815_9.7094_9.7515_9.8454 | 9.4736 |
| SOX10 | 602229 | PCWH syndrome, Waardenburg syndrome | Mutant | 466 | SOX10M112I | 57.3572_54.685_51.7319_50.7538_50.3452 | 57.3572 | 8.0426_8.0427_8.0427_8.0427_8.0426 | 8.0426 |
| SOX10 | 602229 | PCWH syndrome, Waardenburg syndrome | Mutant | 466 | SOX10Q174P | 57.8197_53.8592_51.4172_51.1991_50.7715 | 57.8197 | 8.0426_8.0426_8.0427_8.0427_8.0427 | 8.0426 |
| SOX2 | 184429 | Microphthalmia | WT | 317 | SOX2WT | 59.77_55.7987_55.7913_55.7685_55.5904 | 59.77 | 8.1063_8.1068_8.1065_8.1071_8.1079 | 8.1063 |
| SOX2 | 184429 | Microphthalmia | Mutant | 317 | SOX2R74P | 59.6165_56.3934_55.9668_55.6517_55.4882 | 59.6165 | 8.1885_8.1882_8.1884_8.1883_8.1883 | 8.1885 |
| SOX2 | 184429 | Microphthalmia | Mutant | 317 | SOX2W51R | 59.5903_56.8796_56.628_55.9856_55.8894 | 59.5903 | 8.1883_8.1884_8.1884_8.1883_8.1883 | 8.1883 |
| SOX2 | 184429 | Microphthalmia | Mutant | 317 | SOX2W79S | 59.4415_56.5771_56.2468_55.8621_55.4648 | 59.4415 | 8.1884_8.1884_8.1883_8.1885_8.1884 | 8.1884 |
| SPATA7 | 609868 | Leber congenital amaurosis 3, Leber congenital amaurosis | WT | 599 | SPATA7NM_018418WT | 57.5259_53.8506_53.4273_52.5196_52.3619 | 57.5259 | 8.0286_8.0287_8.0289_8.0287_8.0288 | 8.0286 |
| SPATA7 | 609868 | Leber congenital amaurosis 3, Leber congenital amaurosis | Mutant | 599 | SPATA7NM_018418E405D | 57.3007_53.7092_53.4262_52.8562_51.943 | 57.3007 | 8.0286_8.0286_8.0286_8.0288_8.0287 | 8.0286 |
| SPATA7 | 609868 | Leber congenital amaurosis 3, Leber congenital amaurosis | Mutant | 599 | SPATA7NM_018418I371T | 56.7201_53.5593_52.6643_51.9069_51.8733 | 56.7201 | 8.0287_8.0287_8.0288_8.0289_8.0287 | 8.0287 |
| SPATA7 | 609868 | Leber congenital amaurosis 3, Leber congenital amaurosis | Mutant | 394 | SPATA7NM_018418R395X | 60.1541_57.7458_57.6167_56.9902_56.1637 | 60.1541 | 8.0344_8.0345_8.0345_8.0346_8.0345 | 8.0344 |
| SPATA7 | 609868 | Leber congenital amaurosis 3, Leber congenital amaurosis | Mutant | 599 | SPATA7NM_018418Y367C | 56.9322_53.457_52.316_52.1353_51.5925 | 56.9322 | 8.0287_8.0287_8.0288_8.0287_8.0288 | 8.0287 |
| SPG7 | 602783 | Spastic paraplegia 7, autosomal recessive, Baraitser - Winter syndrome, Spastic paraplegia, autosomal recessive | WT | 690 | SPG7WT | 79.6868_79.1944_78.565_77.9444_77.9005 | 79.6868 | 6.1921_7.9569_7.9586_7.9596_7.9604 | 6.1921 |
| SPG7 | 602783 | Spastic paraplegia 7, autosomal recessive, Baraitser - Winter syndrome, Spastic paraplegia, autosomal recessive | Mutant | 689 | SPG7518MISSING | 79.938_79.391_78.8486_77.9068_77.8092 | 79.938 | 6.4569_8.0395_8.0395_8.0396_8.0397 | 6.4569 |
| SPG7 | 602783 | Spastic paraplegia 7, autosomal recessive, Baraitser - Winter syndrome, Spastic paraplegia, autosomal recessive | Mutant | 690 | SPG7A510V | 79.8707_79.0304_78.4796_78.284_77.3327 | 79.8707 | 7.9556_7.9573_7.9576_7.96_7.2405 | 7.9556 |
| STRA6 | 610745 | Microphthalmia, Coloboma | WT | 667 | STRA6WT | 77.9457_77.1276_76.6635_75.2315_74.7027 | 77.9457 | 8.0375_8.0375_8.0375_8.0375_8.0377 | 8.0375 |
| STRA6 | 610745 | Microphthalmia, Coloboma | Mutant | 667 | STRA6P90L | 76.9985_76.3757_76.0129_74.6942_73.8957 | 76.9985 | 8.0373_8.0375_8.0375_8.0374_8.0376 | 8.0373 |
| STRA6 | 610745 | Microphthalmia, Coloboma | Mutant | 667 | STRA6T644M | 77.6373_76.9768_76.4393_74.8381_74.5992 | 77.6373 | 8.0375_8.0376_8.0374_8.0376_8.0375 | 8.0375 |
| TBC1D24 | 613577 | Hypomagnesemia 5, renal, with ocular involvement | WT | 553 | TBC1D24NM_020705WT | 86.2956_84.3837_83.494_83.092_80.4624 | 86.2956 | 5.7132_8.0322_8.0323_8.0323_8.0323 | 5.7132 |
| TBC1D24 | 613577 | Hypomagnesemia 5, renal, with ocular involvement | Mutant | 553 | TBC1D24NM_020705L159P | 84.5231_84.5118_84.0867_83.1914_83.0585 | 84.5231 | 5.6941_8.0323_8.0323_8.0323_8.0323 | 5.6941 |
| TBC1D24 | 613577 | Hypomagnesemia 5, renal, with ocular involvement | Mutant | 553 | TBC1D24NM_020705R242C | 84.4794_83.5687_82.4854_82.0157_80.6019 | 84.4794 | 5.6423_8.0322_8.0323_8.0323_8.0322 | 5.6423 |
| TBK1 | 604834 | Frontotemporal dementia and/or amyotrophic lateral sclerosis 4 | WT | 729 | TBK1WT | 89.6835_89.1937_89.0755_88.986_88.7058 | 89.6835 | 7.7237_8.0557_8.0555_8.0556_8.0556 | 7.7237 |
| TBK1 | 604834 | Frontotemporal dementia and/or amyotrophic lateral sclerosis 4 | Mutant | 729 | TBK1E696K | 89.6594_89.2319_88.7286_88.6209_88.49 | 89.6594 | 7.7166_7.9545_7.9547_7.955_7.9546 | 7.7166 |
| TBK1 | 604834 | Frontotemporal dementia and/or amyotrophic lateral sclerosis 4 | Mutant | 729 | TBK1G159A | 89.7233_89.1916_89.0599_88.8818_88.8664 | 89.7233 | 7.7556_8.0551_8.0553_8.0561_8.0561 | 7.7556 |
| TCAP | 604488 | Muscular dystrophy, limb-girdle | WT | 167 | TCAPWT | 78.5085_78.3475_76.7672_76.7054_76.2179 | 78.5085 | 8.1581_8.1499_8.1612_8.1533_8.1559 | 8.1581 |
| TCAP | 604488 | Muscular dystrophy, limb-girdle | Mutant | 167 | TCAPP90L | 78.7472_78.4014_75.9707_74.5258_73.1261 | 78.7472 | 8.112_8.1242_8.1343_8.124_8.1221 | 8.112 |
| TCAP | 604488 | Muscular dystrophy, limb-girdle | Mutant | 167 | TCAPR70W | 78.5944_78.3724_76.0508_75.9355_75.0642 | 78.5944 | 8.1411_8.1574_8.1438_8.1591_8.1612 | 8.1411 |
| TCF4 | 602272 | Corneal dystrophy, Fuchs endothelial, 3, Corneal endothelial dystrophy | WT | 671 | TCF4WT | 51.027_48.2365_46.8254_46.8183_46.6955 | 51.027 | 8.0189_8.019_8.019_8.0191_8.0191 | 8.0189 |
| TCF4 | 602272 | Corneal dystrophy, Fuchs endothelial, 3, Corneal endothelial dystrophy | Mutant | 671 | TCF4R576Q | 50.6736_48.4072_47.0932_46.9418_46.7591 | 50.6736 | 7.8918_7.895_7.9079_7.9173_7.9182 | 7.8918 |
| TCF4 | 602272 | Corneal dystrophy, Fuchs endothelial, 3, Corneal endothelial dystrophy | Mutant | 671 | TCF4R576W | 50.8513_48.2724_46.9192_46.5168_46.5146 | 50.8513 | 8.0191_8.019_8.0192_8.019_8.0191 | 8.0191 |
| TCOF1 | 606847 | Treacher Collins syndrome 1 | WT | 1411 | TCOF1WT | 42.494_40.0469_39.879_38.8724_37.6009 | 42.494 | 8.049_8.0491_8.0491_8.049_8.0491 | 8.049 |
| TCOF1 | 606847 | Treacher Collins syndrome 1 | Mutant | 1411 | TCOF1W53R | 43.1655_41.2108_40.1944_38.714_37.6653 | 43.1655 | 8.1811_8.1812_8.1812_8.1812_8.1813 | 8.1811 |
| TCTN3 | 613847 | Joubert syndrome | WT | 585 | TCTN3WT | 74.154_72.25_72.2264_72.2053_71.842 | 74.154 | 6.0722_7.9404_7.9471_7.9483_7.9501 | 6.0722 |
| TCTN3 | 613847 | Joubert syndrome | Mutant | 585 | TCTN3G314R | 75.5024_75.2444_74.4227_73.1468_72.6313 | 75.5024 | 5.6863_8.033_8.0341_8.0342_8.0341 | 5.6863 |
| TDRD7 | 611258 | Congenital cataract and developmental cataract, Cataract | WT | 1098 | TDRD7WT | 73.2535_73.1415_71.8996_71.8064_71.0369 | 73.2535 | 10.2127_10.2128_10.2128_10.2127_10.2128 | 10.2127 |
| TDRD7 | 611258 | Congenital cataract and developmental cataract, Cataract | Mutant | 1097 | TDRD7618missing | 72.8962_72.6735_71.9278_71.4689_71.2313 | 72.8962 | 9.4466_9.4467_9.4467_9.447_9.4469 | 9.4466 |
| TEAD1 | 189967 | Chorioretinopathy, Sveinsson chorioretinal atrophy | WT | 426 | TEAD1WT | 77.4899_75.6999_75.3859_75.3095_74.668 | 77.4899 | 8.81_8.81_8.81_8.81_8.81 | 8.81 |
| TEAD1 | 189967 | Chorioretinopathy, Sveinsson chorioretinal atrophy | Mutant | 426 | TEAD1Y421H | 77.3095_76.1706_76.0026_75.3314_74.3062 | 77.3095 | 8.0338_8.0338_8.0337_8.0339_8.0339 | 8.0338 |
| TEK | 600221 | Congenital primary glaucoma | WT | 1102 | TEKWT | 84.0153_82.6383_79.5469_78.3452_73.0609 | 84.0153 | 8.915_8.9151_8.9151_8.9153_8.9153 | 8.915 |
| TEK | 600221 | Congenital primary glaucoma | Mutant | 914 | TEK19-210MISSING | 84.9991_82.6711_77.3326_77.3037_76.5048 | 84.9991 | 6.3622_8.3318_8.336_8.338_8.338 | 6.3622 |
| TEK | 600221 | Congenital primary glaucoma | Mutant | 1102 | TEKC233Y | 83.3881_82.0869_75.9485_75.3588_72.6468 | 83.3881 | 7.8902_7.8902_7.8902_7.8902_7.8902 | 7.8902 |
| TEK | 600221 | Congenital primary glaucoma | Mutant | 1102 | TEKK294N | 83.1669_82.6499_75.853_75.1053_71.8877 | 83.1669 | 8.9574_8.9578_8.9578_8.9576_8.9578 | 8.9574 |
| TFAP2A | 107580 | Branchiooculofacial syndrome | WT | 433 | TFAP2AWT | 68.171_66.3259_65.1531_64.7905_64.5814 | 68.171 | 8.0387_8.0386_8.0385_8.0386_8.0387 | 8.0387 |
| TFAP2A | 107580 | Branchiooculofacial syndrome | Mutant | 433 | TFAP2AL249P | 66.4902_66.1926_65.1182_64.4714_64.0271 | 66.4902 | 8.0386_8.0385_8.0387_8.0386_8.0387 | 8.0386 |
| TGFBI | 601692 | Corneal dystrophy, lattice, Corneal dystrophy, Reis-Bucklers type, Corneal dystrophy, Corneal dystrophy, Thiel-Behnke type, Corneal dystrophy, Groenouw | WT | 660 | TGFBIWT | 91.4934_91.1866_90.4335_90.0538_89.6746 | 91.4934 | 8.047_8.0472_8.0471_8.0471_8.0471 | 8.047 |
| TGFBI | 601692 | Corneal dystrophy, lattice, Corneal dystrophy, Reis-Bucklers type, Corneal dystrophy, Corneal dystrophy, Thiel-Behnke type, Corneal dystrophy, Groenouw | Mutant | 660 | TGFBIR124C | 92.3495_91.3703_91.0159_90.683_90.1055 | 92.3495 | 8.0471_8.0471_8.0472_8.0472_8.0472 | 8.0471 |
| TGFBI | 601692 | Corneal dystrophy, lattice, Corneal dystrophy, Reis-Bucklers type, Corneal dystrophy, Corneal dystrophy, Thiel-Behnke type, Corneal dystrophy, Groenouw | Mutant | 660 | TGFBIR555W | 91.367_90.8124_90.076_89.684_89.4157 | 91.367 | 8.0471_8.0472_8.0471_8.0472_8.0471 | 8.0471 |
| TIMM8A | 300356 | Mohr-Tranebjaerg syndrome | WT | 97 | TIMM8AWT | 85.3074_85.1503_84.8947_84.0701_83.9968 | 85.3074 | 7.1643_8.1407_8.1502_8.1588_8.1493 | 7.1643 |
| TIMM8A | 300356 | Mohr-Tranebjaerg syndrome | Mutant | 97 | TIMM8AC66W | 84.9091_84.6829_84.3635_83.9313_83.6919 | 84.9091 | 6.7987_8.1665_8.1624_8.1855_8.2025 | 6.7987 |
| TIMP3 | 188826 | Sorsby fundus dystrophy | WT | 188 | TIMP3WT | 91.7054_91.2851_90.3075_90.0326_89.1983 | 91.7054 | 7.3801_7.8763_7.9135_7.9076_7.9143 | 7.3801 |
| TIMP3 | 188826 | Sorsby fundus dystrophy | Mutant | 188 | TIMP3G189C | 92.4629_90.8016_90.5582_89.7371_89.3277 | 92.4629 | 7.3291_8.1899_8.1987_8.1992_8.205 | 7.3291 |
| TIMP3 | 188826 | Sorsby fundus dystrophy | Mutant | 188 | TIMP3Y191C | 92.4414_90.7188_90.5939_89.9298_89.4003 | 92.4414 | 7.3476_7.8856_7.9179_7.9165_7.9092 | 7.3476 |
| TINF2 | 604319 | Revesz syndrome | WT | 450 | TINF2WT | 59.7429_58.5643_57.7642_57.1881_56.998 | 59.7429 | 8.81_8.81_8.81_8.81_8.81 | 8.81 |
| TINF2 | 604319 | Revesz syndrome | Mutant | 450 | TINF2K280E | 60.1568_59.4719_58.7195_58.5899_57.4744 | 60.1568 | 8.0404_8.0405_8.0404_8.0405_8.0405 | 8.0404 |
| TINF2 | 604319 | Revesz syndrome | Mutant | 450 | TINF2R282H | 59.0053_58.9477_58.2007_57.487_56.808 | 59.0053 | 8.0405_8.0405_8.0406_8.0405_8.0405 | 8.0405 |
| TK2 | 188250 | Progressive external ophthalmoplegia，autosomal recessive 3 | WT | 232 | TK2WT | 91.1121_91.0743_90.9822_90.7137_90.4694 | 91.1121 | 7.9705_7.9708_7.9711_7.9711_7.9709 | 7.9705 |
| TK2 | 188250 | Progressive external ophthalmoplegia，autosomal recessive 3 | Mutant | 232 | TK2R183W | 90.9398_90.854_90.5306_90.323_90.2959 | 90.9398 | 7.97_7.9701_7.9706_7.972_7.9722 | 7.97 |
| TK2 | 188250 | Progressive external ophthalmoplegia，autosomal recessive 3 | Mutant | 232 | TK2T108M | 91.1545_90.8663_90.8271_90.7067_90.5965 | 91.1545 | 7.5869_7.6013_7.6017_7.6041_7.6023 | 7.5869 |
| TLR3 | 603029 | Macular degeneration, age-related | WT | 881 | TLR3WT | 91.0854_90.8685_89.9532_88.7383_88.0355 | 91.0854 | 8.1761_8.1763_8.1762_8.1762_8.1762 | 8.1761 |
| TLR3 | 603029 | Macular degeneration, age-related | Mutant | 881 | TLR3P554S | 91.5197_90.8342_90.1854_88.0566_87.5534 | 91.5197 | 8.1762_8.1763_8.1762_8.1763_8.1763 | 8.1762 |
| TLR4 | 603030 | Macular degeneration, age-related | WT | 816 | TLR4WT | 88.2169_88.1061_87.583_87.4947_87.4262 | 88.2169 | 8.0713_8.0718_8.0716_8.0725_8.0723 | 8.0713 |
| TLR4 | 603030 | Macular degeneration, age-related | Mutant | 816 | TLR4P714H | 88.5488_88.2763_87.3511_87.2746_86.8439 | 88.5488 | 8.0449_8.045_8.0449_8.045_8.0449 | 8.0449 |
| TMEM114 | 611579 | Cataract | WT | 223 | TMEM114WT | 81.2085_80.9828_76.4936_76.3112_75.1259 | 81.2085 | 7.6978_7.7019_7.6984_7.707_7.7103 | 7.6978 |
| TMEM114 | 611579 | Cataract | Mutant | 223 | TMEM114F106L | 79.739_79.0095_74.3428_74.2313_74.2172 | 79.739 | 7.7085_7.7119_7.7214_7.7225_7.9634 | 7.7085 |
| TMEM114 | 611579 | Cataract | Mutant | 223 | TMEM114I35T | 81.0357_80.8927_76.7483_74.2827_73.2543 | 81.0357 | 7.6905_7.6906_7.7128_7.7502_7.9657 | 7.6905 |
| TMEM126A | 612988 | Optic atrophy 7, Optic atrophy | WT | 195 | TMEM126AWT | 90.1374_90.0876_89.9858_89.0068_85.3447 | 90.1374 | 7.7705_7.8366_7.9377_7.9806_7.9909 | 7.7705 |
| TMEM126A | 612988 | Optic atrophy 7, Optic atrophy | Mutant | 54 | TMEM126AR55X | 83.4514_77.8747_77.4791_77.286_74.8076 | 83.4514 | 8.0879_8.0758_8.1136_8.1016_8.1268 | 8.0879 |
| TMEM138 | 614459 | Joubert syndrome | WT | 162 | TMEM138WT | 87.2708_86.1037_81.4774_80.5436_78.0983 | 87.2708 | 7.8593_7.9205_7.9118_7.9252_7.9291 | 7.8593 |
| TMEM138 | 614459 | Joubert syndrome | Mutant | 162 | TMEM138A127V | 87.7737_86.7288_80.9327_80.5486_78.6218 | 87.7737 | 7.9247_7.9351_7.9353_8.0854_8.0799 | 7.9247 |
| TMEM138 | 614459 | Joubert syndrome | Mutant | 162 | TMEM138Y130C | 85.5708_84.812_78.4959_78.2474_74.8465 | 85.5708 | 7.926_7.9386_7.9273_8.0915_8.095 | 7.926 |
| TMEM216 | 613277 | Meckel syndrome, Joubert syndrome | WT | 145 | TMEM216WT | 90.1019_89.1101_88.7872_88.2983_79.1432 | 90.1019 | 7.8879_7.8943_7.9093_7.9173_7.9055 | 7.8879 |
| TMEM216 | 613277 | Meckel syndrome, Joubert syndrome | Mutant | 145 | TMEM216R73H | 88.0309_84.9065_83.6644_83.597_76.1296 | 88.0309 | 8.0104_8.0136_8.0195_8.0253_8.0292 | 8.0104 |
| TMEM231 | 614949 | Joubert syndrome, Meckel syndrome | WT | 316 | TMEM231WT | 88.5288_88.0494_87.6488_87.5113_84.2071 | 88.5288 | 8.1873_8.1873_8.1873_8.1874_8.1874 | 8.1873 |
| TMEM231 | 614949 | Joubert syndrome, Meckel syndrome | Mutant | 316 | TMEM231D209N | 88.944_88.3124_87.6499_87.5663_84.3767 | 88.944 | 8.1873_8.1873_8.1872_8.1873_8.1874 | 8.1873 |
| TMEM231 | 614949 | Joubert syndrome, Meckel syndrome | Mutant | 316 | TMEM231Q272P | 88.3769_88.0087_87.836_87.1363_83.9671 | 88.3769 | 8.1873_8.1874_8.1874_8.1872_8.1874 | 8.1873 |
| TMEM67 | 609884 | Meckel syndrome, Joubert syndrome, Nephronophthisis, COACH syndrome | WT | 960 | TMEM67WT | 85.5901_85.2727_85.2128_84.9387_84.4678 | 85.5901 | 8.0513_8.0513_8.0513_8.0513_8.0513 | 8.0513 |
| TMEM67 | 609884 | Meckel syndrome, Joubert syndrome, Nephronophthisis, COACH syndrome | Mutant | 960 | TMEM67I833T | 85.7552_85.408_85.2866_85.2099_85.1855 | 85.7552 | 8.0512_8.0513_8.0513_8.0513_8.0514 | 8.0512 |
| TMEM67 | 609884 | Meckel syndrome, Joubert syndrome, Nephronophthisis, COACH syndrome | Mutant | 960 | TMEM67Y513C | 86.0255_85.562_85.3115_84.9468_84.7538 | 86.0255 | 8.0512_8.0512_8.0513_8.0513_8.0514 | 8.0512 |
| TOPORS | 609507 | Retinitis pigmentosa | WT | 1045 | TOPORSWT | 49.2976_45.5018_44.6492_44.5123_44.3005 | 49.2976 | 5.2595_8.3354_8.3571_8.3618_8.3655 | 5.2595 |
| TOPORS | 609507 | Retinitis pigmentosa | Mutant | 1045 | TOPORSR126C | 49.8293_45.6592_45.1717_44.5425_44.4877 | 49.8293 | 5.2537_8.3045_8.338_8.353_8.3529 | 5.2537 |
| TPM3 | 191030 | Myopathy, congenital, with fiber-type disproportion | WT | 284 | TPM3WT | 91.3516_89.7941_89.385_89.2799_89.0514 | 91.3516 | 7.9726_7.9735_7.9723_7.9748_7.977 | 7.9726 |
| TPM3 | 191030 | Myopathy, congenital, with fiber-type disproportion | Mutant | 284 | TPM3R168C | 91.4463_89.7723_89.2933_89.254_88.7805 | 91.4463 | 7.9748_7.9753_7.9756_7.9755_7.9766 | 7.9748 |
| TPM3 | 191030 | Myopathy, congenital, with fiber-type disproportion | Mutant | 284 | TPM3R168H | 91.421_90.1082_89.4845_89.1958_89.039 | 91.421 | 7.9654_7.9727_7.9733_7.975_7.9729 | 7.9654 |
| TPP1 | 607998 | Ceroid lipofuscinosis, neuronal | WT | 368 | TPP1WT | 95.9976_95.1945_93.8499_93.6706_92.837 | 95.9976 | 7.2521_7.5224_7.5229_7.5228_7.5237 | 7.2521 |
| TPP1 | 607998 | Ceroid lipofuscinosis, neuronal | Mutant | 368 | TPP1C365N | 95.8856_94.7209_93.3451_93.2013_92.2049 | 95.8856 | 7.3708_8.0079_8.0158_8.0164_8.0189 | 7.3708 |
| TPP1 | 607998 | Ceroid lipofuscinosis, neuronal | Mutant | 368 | TPP1R447H | 95.0754_94.72_94.2548_93.2116_93.1199 | 95.0754 | 7.2462_7.5228_7.5228_7.5234_7.5239 | 7.2462 |
| TRIM32 | 602290 | Bardet-Biedl syndrome | WT | 652 | TRIM32WT | 79.9152_78.1001_77.1563_76.5753_75.9844 | 79.9152 | 7.968_7.9757_7.9757_7.9764_7.9764 | 7.968 |
| TRIM32 | 602290 | Bardet-Biedl syndrome | Mutant | 651 | TRIM32588Missing | 79.3182_78.1476_77.4547_76.759_76.6849 | 79.3182 | 7.8198_8.0343_8.0342_8.0343_8.0344 | 7.8198 |
| TRIM32 | 602290 | Bardet-Biedl syndrome | Mutant | 652 | TRIM32P130S | 79.0615_77.8753_76.7689_76.002_74.0992 | 79.0615 | 8.0416_8.0416_8.0417_8.0417_8.0416 | 8.0416 |
| TRIM44 | 612298 | Aniridia 3 | WT | 344 | TRIM44WT | 73.2706_72.261_72.1471_71.9253_70.7522 | 73.2706 | 8.1894_8.1894_8.1896_8.1893_8.1896 | 8.1894 |
| TRIM44 | 612298 | Aniridia 3 | Mutant | 344 | TRIM44G155R | 73.9397_72.5373_72.3389_72.0125_70.8578 | 73.9397 | 8.1893_8.1894_8.1895_8.1896_8.1894 | 8.1893 |
| TRPM1 | 603576 | Congenital static night blindness, Night blindness, congenital stationary (complete), 1C, autosomal recessive | WT | 1603 | TRPM1NM_002420WT | 66.5761_65.9115_64.1911_64.0001_63.5894 | 66.5761 | 6.2857_6.4498_7.15_6.8656_7.0197 | 6.2857 |
| TRPM1 | 603576 | Congenital static night blindness, Night blindness, congenital stationary (complete), 1C, autosomal recessive | Mutant | 1603 | TRPM1NM_002420L99P | 66.3626_65.8489_64.4009_64.3577_64.0451 | 66.3626 | 7.947_7.947_7.947_7.947_7.947 | 7.947 |
| TRPM1 | 603576 | Congenital static night blindness, Night blindness, congenital stationary (complete), 1C, autosomal recessive | Mutant | 1603 | TRPM1NM_002420R74C | 66.8581_66.2896_65.4407_64.6688_64.4806 | 66.8581 | 7.947_7.947_7.947_7.947_7.947 | 7.947 |
| TSPAN12 | 613138 | Spondylo-megaepiphyseal-metaphyseal dysplasia, Familial exudative vitreoretinopathy | WT | 306 | TSPAN12WT | 78.3575_76.6001_76.4377_76.319_76.0815 | 78.3575 | 8.1942_8.1943_8.1942_8.1942_8.1941 | 8.1942 |
| TSPAN12 | 613138 | Spondylo-megaepiphyseal-metaphyseal dysplasia, Familial exudative vitreoretinopathy | Mutant | 306 | TSPAN12A237P | 78.7092_77.1014_76.8858_76.6354_76.5722 | 78.7092 | 8.1942_8.1942_8.1943_8.1943_8.1942 | 8.1942 |
| TSPAN12 | 613138 | Spondylo-megaepiphyseal-metaphyseal dysplasia, Familial exudative vitreoretinopathy | Mutant | 306 | TSPAN12Y138C | 78.172_76.5848_75.9249_75.6248_74.6161 | 78.172 | 8.1942_8.1942_8.1941_8.1943_8.1942 | 8.1942 |
| TTC21B | 612014 | Nephronophthisis, jeune syndrome | WT | 1316 | TTC21BWT | 83.0961_82.674_82.2391_81.3883_80.6913 | 83.0961 | 6.2846_8.1835_8.1835_8.1835_8.1835 | 6.2846 |
| TTC21B | 612014 | Nephronophthisis, jeune syndrome | Mutant | 1316 | TTC21BD755Y | 82.7505_82.6675_82.2512_81.4815_80.2856 | 82.7505 | 6.2817_7.9518_7.9518_7.9541_7.9555 | 6.2817 |
| TTC21B | 612014 | Nephronophthisis, jeune syndrome | Mutant | 1316 | TTC21BS591N | 83.4638_82.3681_81.5276_80.9963_80.6801 | 83.4638 | 6.2845_8.1756_8.1773_8.1779_8.1805 | 6.2845 |
| TTC21B | 612014 | Nephronophthisis, jeune syndrome | Mutant | 230 | TTC21BT231X | 92.226_91.4738_90.5455_90.1355_89.283 | 92.226 | 7.9732_7.975_7.9761_7.9752_7.9754 | 7.9732 |
| TTC8 | 608132 | Retinitis pigmentosa, Bardet-Biedl syndrome | WT | 515 | TTC8WT | 87.747_87.1971_76.1375_75.6033_75.1294 | 87.747 | 7.9452_7.9452_7.9454_7.9455_7.9456 | 7.9452 |
| TTC8 | 608132 | Retinitis pigmentosa, Bardet-Biedl syndrome | Mutant | 495 | TTC838-57MISSING | 86.7618_86.2612_77.3548_76.9477_75.6973 | 86.7618 | 8.0344_8.0343_8.0344_8.0343_8.0343 | 8.0344 |
| TTR | 176300 | Other etiologies of vitreous lesions | WT | 127 | TTRWT | 93.0373_93.011_92.9655_92.7624_92.1582 | 93.0373 | 7.7073_8.1138_8.1141_8.1141_8.1141 | 7.7073 |
| TTR | 176300 | Other etiologies of vitreous lesions | Mutant | 127 | TTRF53L | 93.398_93.3297_93.1937_92.7673_92.6835 | 93.398 | 7.6621_8.1141_8.1141_8.1141_8.1141 | 7.6621 |
| TTR | 176300 | Other etiologies of vitreous lesions | Mutant | 127 | TTRV50M | 93.4015_93.1784_92.9025_92.6695_92.6465 | 93.4015 | 7.7303_8.1141_8.1141_8.1141_8.1141 | 7.7303 |
| TTR | 176300 | Other etiologies of vitreous lesions | Mutant | 127 | TTRV91A | 93.2062_93.175_93.0767_92.716_92.633 | 93.2062 | 7.6701_8.1141_8.1141_8.1141_8.1141 | 7.6701 |
| TUBB3 | 602661 | Fibrosis of extraocular muscles, congenital, Congenital fibrosis of the extraocular muscles | WT | 450 | TUBB3WT | 91.8437_90.6581_90.131_89.6444_88.6483 | 91.8437 | 8.0405_8.0407_8.0406_8.0407_8.0407 | 8.0405 |
| TUBB3 | 602661 | Fibrosis of extraocular muscles, congenital, Congenital fibrosis of the extraocular muscles | Mutant | 450 | TUBB3D417H | 91.6276_90.2056_89.5948_89.5561_88.0006 | 91.6276 | 9.2051_9.2054_9.2054_9.2056_9.206 | 9.2051 |
| TUBB3 | 602661 | Fibrosis of extraocular muscles, congenital, Congenital fibrosis of the extraocular muscles | Mutant | 450 | TUBB3R62Q | 91.5535_90.1808_89.6813_89.0491_87.9302 | 91.5535 | 8.0405_8.0406_8.0406_8.0406_8.0407 | 8.0405 |
| TUBGCP6 | 610053 | Chorioretinopathy | WT | 1819 | TUBGCP6WT | 60.9422_60.4505_58.7064_58.2701_57.1501 | 60.9422 | 7.976_7.9761_7.9761_7.9761_7.9761 | 7.976 |
| TUBGCP6 | 610053 | Chorioretinopathy | Mutant | 1834 | TUBGCP6c.T5458G | 59.9885_59.7331_59.0155_56.9998_56.2463 | 59.9885 | 7.9699_7.9727_7.9891_8.0035_8.0567 | 7.9699 |
| TULP1 | 602280 | Retinitis pigmentosa, Leber congenital amaurosis 15, Leber congenital amaurosis | WT | 542 | TULP1NM_003322WT | 65.8301_63.5364_61.0309_60.8945_59.682 | 65.8301 | 8.0318_8.0319_8.0319_8.032_8.032 | 8.0318 |
| TULP1 | 602280 | Retinitis pigmentosa, Leber congenital amaurosis 15, Leber congenital amaurosis | Mutant | 542 | TULP1NM_003322K489R | 64.9378_64.0787_61.1362_60.8145_59.9287 | 64.9378 | 7.961_7.9621_7.9638_7.9673_7.9676 | 7.961 |
| TULP1 | 602280 | Retinitis pigmentosa, Leber congenital amaurosis 15, Leber congenital amaurosis | Mutant | 542 | TULP1NM_003322R420P | 65.7234_63.5596_61.0758_60.4844_60.0736 | 65.7234 | 8.0319_8.0319_8.0319_8.0318_8.032 | 8.0319 |
| TYR | 606933 | Albinism, oculocutaneous, Waardenburg syndrome | WT | 511 | TYRWT | 91.9667_91.864_91.7068_91.6385_91.452 | 91.9667 | 8.2262_8.2263_8.2262_8.2277_8.2278 | 8.2262 |
| TYR | 606933 | Albinism, oculocutaneous, Waardenburg syndrome | Mutant | 511 | TYRE294K | 91.8205_91.8136_91.7123_91.5878_91.5848 | 91.8205 | 8.2264_8.2266_8.2268_8.2272_8.2272 | 8.2264 |
| TYR | 606933 | Albinism, oculocutaneous, Waardenburg syndrome | Mutant | 511 | TYRP406L | 91.8191_91.5865_91.2564_91.1417_91.1184 | 91.8191 | 8.2262_8.2264_8.2266_8.2266_8.2269 | 8.2262 |
| TYRP1 | 115501 | Cataract, Albinism, oculocutaneous, Albinism, oculocutaneous | WT | 513 | TYRP1WT | 93.9888_93.8168_92.3696_92.2958_91.8579 | 93.9888 | 7.9439_7.9441_7.9445_7.9451_7.9448 | 7.9439 |
| TYRP1 | 115501 | Cataract, Albinism, oculocutaneous, Albinism, oculocutaneous | Mutant | 513 | TYRP1R356Q | 93.9359_93.7011_92.4251_92.3509_91.8835 | 93.9359 | 7.9585_7.9581_7.9586_7.9586_7.9589 | 7.9585 |
| UBIAD1 | 611632 | Corneal dystrophy, Schnyder type, Corneal dystrophy | WT | 337 | UBIAD1WT | 89.631_89.4891_89.4011_89.2596_89.1547 | 89.631 | 8.1978_8.1977_8.1978_8.1978_8.1979 | 8.1978 |
| UBIAD1 | 611632 | Corneal dystrophy, Schnyder type, Corneal dystrophy | Mutant | 337 | UBIAD1G177R | 89.7531_89.5039_89.1864_89.0374_89.0217 | 89.7531 | 8.1975_8.1975_8.1974_8.1975_8.1975 | 8.1975 |
| UBIAD1 | 611632 | Corneal dystrophy, Schnyder type, Corneal dystrophy | Mutant | 337 | UBIAD1N102S | 89.5605_89.1436_89.028_89.0153_88.7972 | 89.5605 | 8.1973_8.1973_8.1973_8.1972_8.1973 | 8.1973 |
| UCHL1 | 191342 | Spastic paraplegia 79, autosomal recessive, Optic atrophy | WT | 220 | UCHL1WT | 93.8686_93.7704_93.4718_93.3616_92.7663 | 93.8686 | 6.6371_7.4634_7.4644_7.469_7.4695 | 6.6371 |
| UCHL1 | 191342 | Spastic paraplegia 79, autosomal recessive, Optic atrophy | Mutant | 220 | UCHL1I93M | 93.739_93.3985_93.3962_93.0651_92.9662 | 93.739 | 6.6571_7.4688_7.4763_7.478_7.4789 | 6.6571 |
| UCHL1 | 191342 | Spastic paraplegia 79, autosomal recessive, Optic atrophy | Mutant | 220 | UCHL1R178Q | 93.3559_93.2572_93.0993_93.0643_92.7351 | 93.3559 | 6.5635_7.9441_7.9468_7.9468_7.9474 | 6.5635 |
| UNC119 | 604011 | Cone-rod retinal dystrophy | WT | 240 | UNC119WT | 77.932_77.056_76.8001_76.7205_76.4538 | 77.932 | 7.7513_7.771_7.7842_7.7924_7.8172 | 7.7513 |
| UNC119 | 604011 | Cone-rod retinal dystrophy | Mutant | 240 | UNC119G22V | 77.5641_76.7468_76.6329_76.5977_75.9806 | 77.5641 | 7.9742_7.974_7.975_7.9757_7.9764 | 7.9742 |
| UNC45B | 611220 | Cataract 43 | WT | 931 | UNC45BWT | 88.5599_87.8514_87.2618_86.8084_84.6295 | 88.5599 | 6.0604_8.0409_8.0409_8.0409_8.0409 | 6.0604 |
| UNC45B | 611220 | Cataract 43 | Mutant | 931 | UNC45BR805W | 88.6348_87.4981_87.4216_86.4135_85.2555 | 88.6348 | 6.1026_8.0409_8.0409_8.0409_8.0409 | 6.1026 |
| USH1C | 605242 | Usher syndrome | WT | 552 | USH1CWT | 78.1038_77.2239_76.8819_76.3776_75.8319 | 78.1038 | 6.7346_8.0415_8.0416_8.0415_8.0416 | 6.7346 |
| USH1C | 605242 | Usher syndrome | Mutant | 463 | USH1Cc.1220delG | 76.9718_76.599_75.9833_73.8955_73.6636 | 76.9718 | 8.0289_8.0287_8.0287_8.0287_8.0287 | 8.0289 |
| USH1C | 605242 | Usher syndrome | Mutant | 552 | USH1CR103H | 77.7354_76.9183_76.8322_76.2014_74.6563 | 77.7354 | 6.6669_8.0416_8.0416_8.0417_8.0417 | 6.6669 |
| USH1G | 607696 | Usher syndrome | WT | 461 | USH1GWT | 66.7901_64.81_64.0564_64.0142_62.2232 | 66.7901 | 8.0297_8.0297_8.0297_8.0297_8.0298 | 8.0297 |
| USH1G | 607696 | Usher syndrome | Mutant | 461 | USH1GD458V | 66.7002_65.2241_64.1179_63.5869_62.0954 | 66.7002 | 8.0297_8.0297_8.0297_8.0297_8.0298 | 8.0297 |
| USH1G | 607696 | Usher syndrome | Mutant | 461 | USH1GL48P | 67.3923_65.4762_63.7675_63.7312_63.3713 | 67.3923 | 8.0294_8.0297_8.0297_8.0298_8.0298 | 8.0294 |
| USH2A | 608400 | Usher syndrome, Retinitis pigmentosa | WT | 1515 | USH2ANM_007123WT | 82.9619_82.4117_81.5841_81.0653_79.5685 | 82.9619 | 3.9587_4.0182_4.0218_4.1023_4.1595 | 3.9587 |
| USH2A | 608400 | Usher syndrome, Retinitis pigmentosa | Mutant | 1515 | USH2ANM_007123C419F | 83.402_81.4591_80.9258_79.5928_77.4383 | 83.402 | 7.9893_7.9916_8.0434_8.0633_8.0765 | 7.9893 |
| USH2A | 608400 | Usher syndrome, Retinitis pigmentosa | Mutant | 697 | USH2ANM_007123C729X | 93.5801_93.5189_92.3231_92.1831_84.6761 | 93.5801 | 8.0329_8.033_8.0329_8.033_8.0331 | 8.0329 |
| USH2A | 608400 | Usher syndrome, Retinitis pigmentosa | Mutant | 1515 | USH2ANM_007123C759F | 82.8933_82.3708_80.8497_78.7178_77.2355 | 82.8933 | 8.4205_8.4806_8.4827_8.4866_8.5223 | 8.4205 |
| USH2A | 608400 | Usher syndrome, Retinitis pigmentosa | Mutant | 1515 | USH2ANM_007123C934W | 82.7771_82.4219_81.3387_80.5843_78.6162 | 82.7771 | 7.9755_7.9807_8.0016_8.009_8.0205 | 7.9755 |
| USH2A | 608400 | Usher syndrome, Retinitis pigmentosa | Mutant | 1515 | USH2ANM_007123G1526R | 82.6023_80.6676_80.2548_79.0449_78.7614 | 82.6023 | 8.4019_8.4471_8.4605_8.4843_8.5135 | 8.4019 |
| USH2A | 608400 | Usher syndrome, Retinitis pigmentosa | Mutant | 1515 | USH2ANM_007123N346H | 81.8253_81.5762_81.32_80.4242_76.8668 | 81.8253 | 7.978_7.9915_7.9955_8.0407_8.0635 | 7.978 |
| USH2A | 608400 | Usher syndrome, Retinitis pigmentosa | Mutant | 1515 | USH2ANM_007123R334W | 81.6104_81.2322_79.3043_78.9354_78.0407 | 81.6104 | 7.9604_7.9896_7.9864_8.0184_8.0421 | 7.9604 |
| USH2A | 608400 | Usher syndrome, Retinitis pigmentosa | Mutant | 31 | USH2ANM_007123R63X | 87.1225_87.06_84.5524_83.9089_82.8084 | 87.1225 | 8.1128_7.9849_8.0021_8.0254_8.0983 | 8.1128 |
| USH2A | 608400 | Usher syndrome, Retinitis pigmentosa | Mutant | 1515 | USH2ANM_007123S69I | 83.3036_82.0443_82.0008_80.1974_78.3455 | 83.3036 | 7.9539_7.9726_7.9898_7.9888_8.004 | 7.9539 |
| VAX1 | 604294 | Microphthalmia | WT | 334 | VAX1WT | 62.7752_59.0723_58.2663_57.8304_56.4562 | 62.7752 | 8.1827_8.1828_8.1829_8.1829_8.1828 | 8.1827 |
| VAX1 | 604294 | Microphthalmia | Mutant | 334 | VAX1R152S | 63.0881_58.7999_58.6762_57.3685_56.9993 | 63.0881 | 8.1828_8.1827_8.1829_8.1829_8.1826 | 8.1828 |
| VHL | 608537 | Pheochromocytoma, von Hippel-Lindau syndrome | WT | 213 | VHLWT | 84.5248_82.0989_81.1189_80.686_79.3889 | 84.5248 | 7.9107_7.9116_7.9121_7.9131_7.9124 | 7.9107 |
| VHL | 608537 | Pheochromocytoma, von Hippel-Lindau syndrome | Mutant | 213 | VHLC162F | 84.4069_81.9744_81.5564_80.6604_80.2676 | 84.4069 | 7.911_7.9112_7.9123_7.9117_7.9118 | 7.911 |
| VHL | 608537 | Pheochromocytoma, von Hippel-Lindau syndrome | Mutant | 213 | VHLR200W | 84.1504_81.6773_81.406_79.8632_79.4719 | 84.1504 | 7.9094_7.9103_7.9111_7.911_7.9117 | 7.9094 |
| VIM | 193060 | Congenital cataract and developmental cataract | WT | 465 | VIMWT | 77.1105_75.4749_75.0664_73.5802_71.9509 | 77.1105 | 8.0432_8.0432_8.0432_8.0434_8.0432 | 8.0432 |
| VIM | 193060 | Congenital cataract and developmental cataract | Mutant | 465 | VIME151K | 76.6466_75.5098_74.741_73.0145_71.2271 | 76.6466 | 8.0432_8.0433_8.0433_8.0433_8.0433 | 8.0432 |
| VIM | 193060 | Congenital cataract and developmental cataract | Mutant | 465 | VIMQ208R | 76.7838_75.7589_75.111_73.2462_72.2495 | 76.7838 | 8.0432_8.0432_8.0432_8.0432_8.0433 | 8.0432 |
| VSX2 | 142993 | Eye deficit disorder, Microphthalmia, Microphthalmia | WT | 361 | VSX2WT | 61.6771_57.7726_57.663_57.5618_57.5441 | 61.6771 | 8.1938_8.1938_8.1938_8.1939_8.1938 | 8.1938 |
| VSX2 | 142993 | Eye deficit disorder, Microphthalmia, Microphthalmia | Mutant | 361 | VSX2G223R | 60.7846_58.8845_58.2276_58.1633_57.5666 | 60.7846 | 8.1937_8.1937_8.1938_8.1938_8.1939 | 8.1937 |
| VSX2 | 142993 | Eye deficit disorder, Microphthalmia, Microphthalmia | Mutant | 361 | VSX2R200Q | 61.7474_58.4931_58.0334_57.1972_56.8093 | 61.7474 | 8.1936_8.1938_8.1937_8.1938_8.1938 | 8.1936 |
| VSX2 | 142993 | Eye deficit disorder, Microphthalmia, Microphthalmia | Mutant | 361 | VSX2R227W | 60.8283_57.3407_57.0526_56.53_56.1534 | 60.8283 | 8.1937_8.1938_8.1937_8.1938_8.1938 | 8.1937 |
| WDPCP | 613580 | Bardet-Biedl syndrome | WT | 746 | WDPCPWT | 75.7268_75.7124_75.4737_75.1107_74.129 | 75.7268 | 6.6971_7.9575_7.959_7.9588_7.9594 | 6.6971 |
| WDPCP | 613580 | Bardet-Biedl syndrome | Mutant | 746 | WDPCPD54N | 76.0199_75.1637_74.9517_74.8368_74.672 | 76.0199 | 6.5552_7.7538_7.93_7.9305_7.9319 | 6.5552 |
| WDR19 | 608151 | jeune syndrome, Senior-Loken syndrome, Nephronophthisis | WT | 1342 | WDR19WT | 86.4311_85.9616_84.3311_84.2263_83.2593 | 86.4311 | 7.9256_7.93_7.93_7.93_7.93 | 7.9256 |
| WDR19 | 608151 | jeune syndrome, Senior-Loken syndrome, Nephronophthisis | Mutant | 1342 | WDR19D493H | 86.7433_86.1057_84.5403_84.0969_83.6637 | 86.7433 | 7.9254_7.93_7.93_7.93_7.93 | 7.9254 |
| WDR19 | 608151 | jeune syndrome, Senior-Loken syndrome, Nephronophthisis | Mutant | 1342 | WDR19L710S | 86.3106_85.5956_84.6821_83.8829_83.1015 | 86.3106 | 7.9254_7.93_7.93_7.93_7.93 | 7.9254 |
| WDR36 | 609669 | Glaucoma, Primary open angle glaucoma | WT | 951 | WDR36WT | 83.1326_82.9083_80.4102_80.2887_77.8442 | 83.1326 | 8.048_8.048_8.048_8.0481_8.048 | 8.048 |
| WDR36 | 609669 | Glaucoma, Primary open angle glaucoma | Mutant | 951 | WDR36H411Y | 82.9524_82.3299_80.4879_80.1679_78.1029 | 82.9524 | 8.0479_8.048_8.048_8.048_8.048 | 8.0479 |
| WDR36 | 609669 | Glaucoma, Primary open angle glaucoma | Mutant | 951 | WDR36Y216P | 82.4883_82.1237_79.6899_78.7686_78.5712 | 82.4883 | 7.2451_8.0206_8.0214_8.0216_8.0215 | 7.2451 |
| WFS1 | 606201 | Wolfram syndrome, Diabetes, Congenital cataract and developmental cataract | WT | 890 | WFS1WT | 72.9667_72.6283_72.4691_71.5125_71.3438 | 72.9667 | 8.038_8.0384_8.0404_8.041_8.0407 | 8.038 |
| WFS1 | 606201 | Wolfram syndrome, Diabetes, Congenital cataract and developmental cataract | Mutant | 890 | WFS1E462G | 72.8559_72.8259_72.0607_71.0882_70.1051 | 72.8559 | 8.0385_8.0391_8.0389_8.0405_8.0423 | 8.0385 |
| WRN | 277700 | Werner syndrome | WT | 1431 | WRNWT | 67.5982_66.178_65.5575_65.5549_64.6323 | 67.5982 | 7.4809_8.0564_8.0565_8.0566_8.0566 | 7.4809 |
| WRN | 277700 | Werner syndrome | Mutant | 1431 | WRNK125N | 67.7643_67.1869_65.4987_64.6132_63.8691 | 67.7643 | 7.4835_8.0565_8.0565_8.0565_8.0566 | 7.4835 |
| WRN | 277700 | Werner syndrome | Mutant | 1431 | WRNK135E | 67.5585_67.2499_65.5814_64.9627_64.8661 | 67.5585 | 7.5023_8.0564_8.0564_8.0564_8.0565 | 7.5023 |
| YME1L1 | 607472 | Optic atrophy 11 | WT | 773 | YME1L1WT | 70.6189_70.2566_69.1947_68.3663_68.0642 | 70.6189 | 7.2567_8.0349_8.035_8.035_8.0351 | 7.2567 |
| YME1L1 | 607472 | Optic atrophy 11 | Mutant | 773 | YME1L1R206W | 70.1279_70.0902_68.4924_67.6288_67.2434 | 70.1279 | 6.6347_7.8465_7.9218_8.0455_8.053 | 6.6347 |
| ZEB1 | 189909 | Corneal dystrophy, Fuchs endothelial, 6, Corneal dystrophy, Dystrophia endothelialis corneae | WT | 1124 | ZEB1WT | 48.4396_46.3063_45.5888_45.559_43.215 | 48.4396 | 4.6329_4.6728_4.7268_4.7838_4.8508 | 4.6329 |
| ZEB1 | 189909 | Corneal dystrophy, Fuchs endothelial, 6, Corneal dystrophy, Dystrophia endothelialis corneae | Mutant | 1124 | ZEB1Q810P | 49.1233_47.0414_45.7756_45.0301_43.624 | 49.1233 | 7.6246_7.93_7.93_7.93_7.93 | 7.6246 |
| ZNF408 | 616454 | Retinitis pigmentosa, Exudative vitreoretinopathy 6 | WT | 720 | ZNF408WT | 61.2523_57.8731_56.9499_56.2271_55.9511 | 61.2523 | 7.0005_8.2798_8.2798_8.2799_8.2798 | 7.0005 |
| ZNF408 | 616454 | Retinitis pigmentosa, Exudative vitreoretinopathy 6 | Mutant | 720 | ZNF408H455Y | 59.8629_57.4815_56.3934_54.0308_51.0477 | 59.8629 | 7.1066_8.2799_8.2796_8.2798_8.2799 | 7.1066 |
| ZNF408 | 616454 | Retinitis pigmentosa, Exudative vitreoretinopathy 6 | Mutant | 720 | ZNF408R541C | 59.2093_58.2338_57.1939_55.5201_54.7016 | 59.2093 | 7.0539_8.2797_8.2797_8.2798_8.2799 | 7.0539 |
| ZNF423 | 604557 | Joubert syndrome, Nephronophthisis | WT | 1284 | ZNF423WT | 59.0338_57.569_54.9925_54.1542_54.0496 | 59.0338 | 8.0665_8.0699_8.07_8.07_8.07 | 8.0665 |
| ZNF423 | 604557 | Joubert syndrome, Nephronophthisis | Mutant | 1284 | ZNF423H1277Y | 58.1176_57.7557_55.0502_54.7858_53.4673 | 58.1176 | 7.916_7.9213_7.9274_7.9283_7.9283 | 7.916 |
| ZNF423 | 604557 | Joubert syndrome, Nephronophthisis | Mutant | 1284 | ZNF423P913L | 58.5283_58.4517_54.0085_53.6742_52.9929 | 58.5283 | 8.0669_8.0699_8.07_8.07_8.07 | 8.0669 |
| ZNF513 | 613598 | Retinitis pigmentosa | WT | 541 | ZNF513WT | 60.6955_58.9385_57.7825_56.514_55.7043 | 60.6955 | 8.0329_8.0329_8.0331_8.033_8.0332 | 8.0329 |
| ZNF513 | 613598 | Retinitis pigmentosa | Mutant | 541 | ZNF513C339R | 59.5474_57.0736_55.6493_55.3049_53.8548 | 59.5474 | 8.0318_8.033_8.033_8.033_8.0332 | 8.0318 |
| ZNF644 | 614159 | Myopia | WT | 1327 | ZNF644NM_201269WT | 46.2264_43.3895_43.2588_42.8191_42.6127 | 46.2264 | 7.93_7.93_7.93_7.93_7.93 | 7.93 |
| ZNF644 | 614159 | Myopia | Mutant | 1327 | ZNF644NM_201269I587V | 46.718_43.4764_43.3486_42.5824_42.2569 | 46.718 | 7.93_7.93_7.93_7.93_7.93 | 7.93 |
| ZNF644 | 614159 | Myopia | Mutant | 1327 | ZNF644NM_201269S672G | 46.3148_44.7825_43.4474_43.2505_42.4884 | 46.3148 | 8.0466_8.0468_8.0467_8.0468_8.0468 | 8.0466 |
