# Supplementary figures and images for: Artificial Intelligence-based database for prediction of protein structure and their alterations in ocular diseases
Source: Database (Oxford). 2023 Dec 18;2023:baad083. doi: 10.1093/database/baad083 (PMC10727695; doi:10.1093/database/baad083)

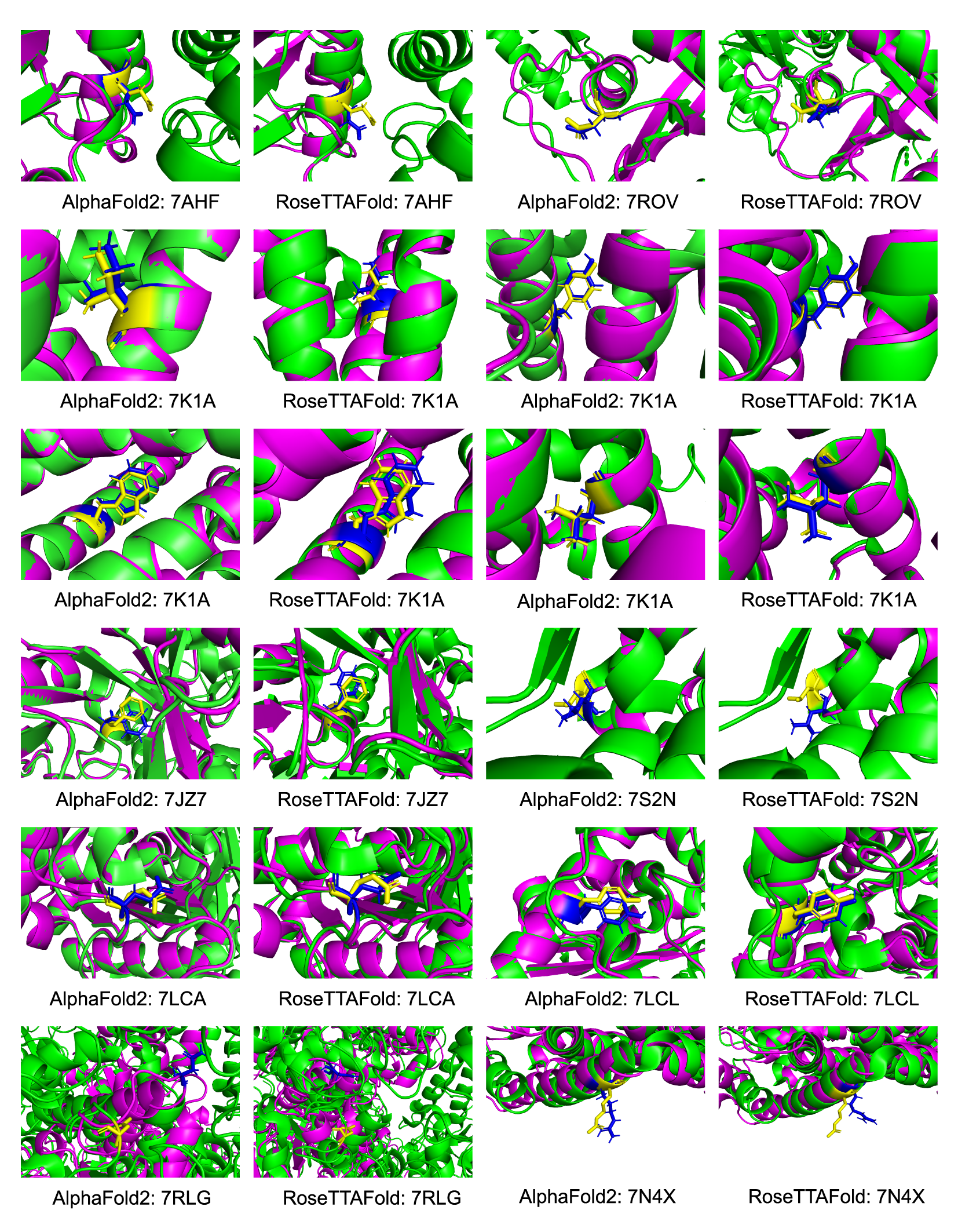

Supplement: baad083_Supp [file baad083_supp.zip › suppl_data/Suppl Figure 1.tif]

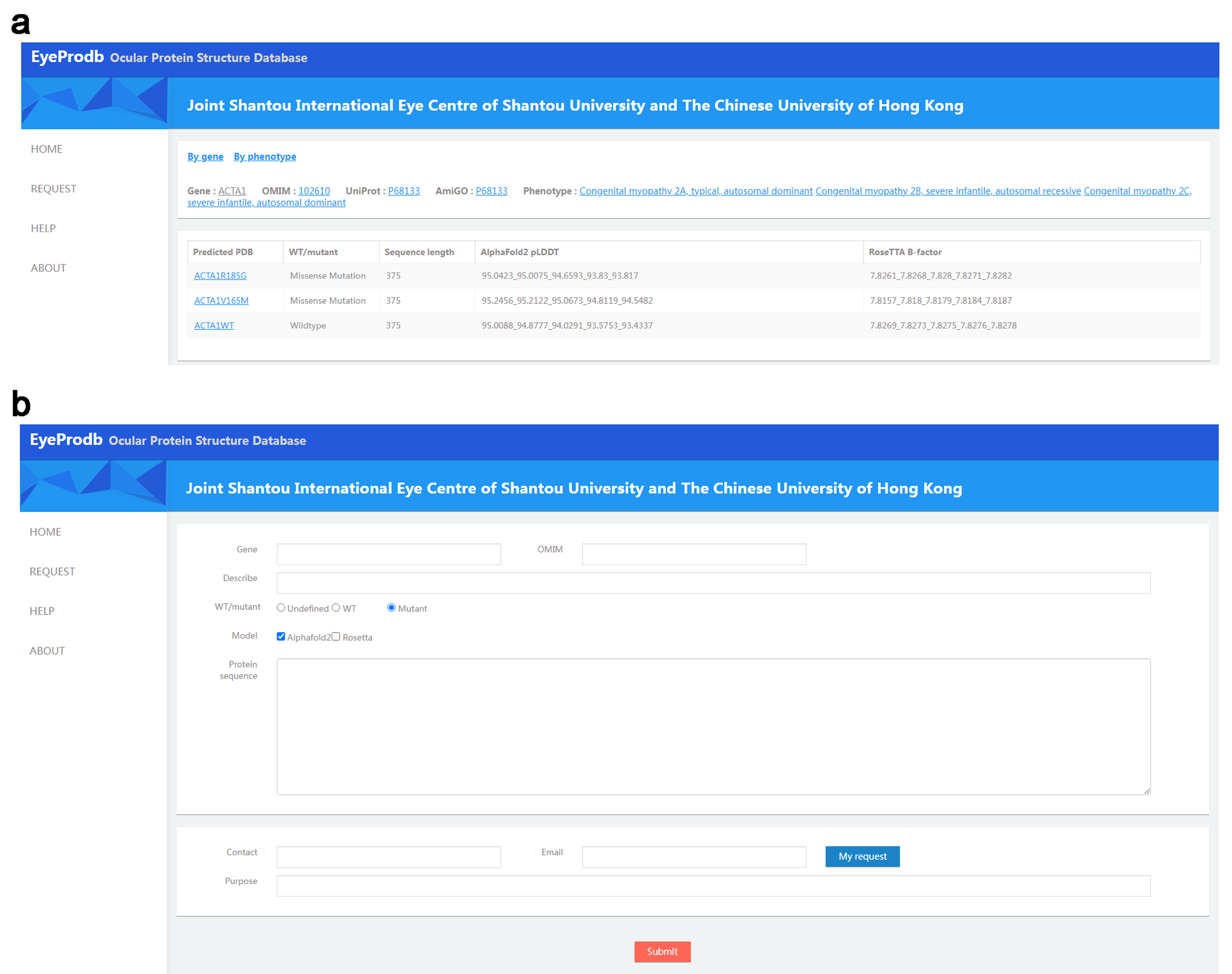

Supplement: baad083_Supp [file baad083_supp.zip › suppl_data/Suppl Figure 2.tif]
